# National and subnational burden of female and male breast cancer and risk factors in Iran from 1990 to 2019: results from the Global Burden of Disease study 2019

**DOI:** 10.1186/s13058-023-01633-4

**Published:** 2023-04-26

**Authors:** Armin Aryannejad, Sahar Saeedi Moghaddam, Baharnaz Mashinchi, Mohammadreza Tabary, Negar Rezaei, Sarvenaz Shahin, Nazila Rezaei, GBD 2019 Iran Breast Cancer Collaborators, Mohsen Abbasi-Kangevari, Zeinab Abbasi-Kangevari, Hedayat Abbastabar, Hassan Abidi, Hassan Abolhassani, Mohammad Aghaali, Bahman Ahadinezhad, Ali Ahmadi, Sepideh Ahmadi, Marjan Ajami, Mohammad Esmaeil Akbari, Yousef Alimohamadi, Sadaf Alipour, Vahid Alipour, Saeed Amini, Ali Arash Anoushirvani, Jalal Arabloo, Morteza Arab-Zozani, Bahar Ataeinia, Seyyed Shamsadin Athari, Abbas Azadmehr, Sina Azadnajafabad, Mohammadreza Azangou-Khyavy, Amirhossein Azari Jafari, Nader Bagheri, Sara Bagherieh, Saeed Bahadory, Sima Besharat, Somayeh Bohlouli, Natália Cruz-Martins, Mostafa Dianatinasab, Mojtaba Didehdar, Shirin Djalalinia, Fariba Dorostkar, Sharareh Eskandarieh, Bita Eslami, Shahab Falahi, Mohammad Farahmand, Ali Fatehizadeh, Masood Fereidoonnezhad, Nasrin Galehdar, Seyyed-Hadi Ghamari, Ahmad Ghashghaee, Maryam Gholamalizadeh, Ali Gholami, Pouya Goleij, Mohamad Golitaleb, Nima Hafezi-Nejad, Arvin Haj-Mirzaian, Aram Halimi, Soheil Hassanipour, Mohammad Heidari, Zahra Heidarymeybodi, Keyvan Heydari, Mohammad-Salar Hosseini, Elham Jamshidi, Roksana Janghorban, Ali Kabir, Leila R. Kalankesh, Taras Kavetskyy, Leila Keikavoosi-Arani, Mohammad Keykhaei, Rovshan Khalilov, Javad Khanali, Mahmoud Khodadost, Ali-Asghar Kolahi, Farzad Kompani, Hamid Reza Koohestani, Mozhgan Letafat-nezhad, Somayeh Livani, Amirhosein Maali, Farzan Madadizadeh, Soleiman Mahjoub, Ata Mahmoodpoor, Mohammad-Reza Malekpour, Reza Malekzadeh, Mohammad Ali Mansournia, Sahar Masoudi, Seyedeh Zahra Masoumi, Entezar Mehrabi Nasab, Seyyedmohammadsadeq Mirmoeeni, Esmaeil Mohammadi, Abdollah Mohammadian-Hafshejani, Mohammad Mohseni, Sara Momtazmanesh, Abdolvahab Moradi, Maryam Moradi, Yousef Moradi, Farhad Moradpour, Rahmatollah Moradzadeh, Abbas Mosapour, Mozhgan Moshtagh, Haleh MousaviIsfahani, Christopher J. L. Murray, Javad Nazari, Seyed Aria Nejadghaderi, Maryam Noori, Hassan Okati-Aliabad, Morteza Oladnabi, Babak Pakbin, Fatemeh PashazadehKan, Hamidreza Pazoki Toroudi, Naeimeh Pourtaheri, Navid Rabiee, Sima Rafiei, Fakher Rahim, Vahid Rahmanian, Samira Raoofi, Mahsa Rashidi, Mohammad-Mahdi Rashidi, Mohammad Sadegh Razeghinia, Nima Rezaei, Saeid Rezaei, Aziz Rezapour, Gholamreza Roshandel, Siamak Sabour, Maryam Sahebazzamani, Amirhossein Sahebkar, Soraya Sajadimajd, Sadaf G. Sepanlou, Saeed Shahabi, Fariba Shahraki-Sanavi, Javad Sharifi-Rad, Reza Shirkoohi, Parnian Shobeiri, Mohammad Sadegh Soltani-Zangbar, Elnaz Tabibian, Majid Taheri, Yasaman Taheri Abkenar, Ahmad Tavakoli, Amir Tiyuri, Seyed Abolfazl Tohidast, Sahel Valadan Tahbaz, Rohollah Valizadeh, Seyed Hossein YahyazadehJabbari, Leila Zaki, Maryam Zamanian, Iman Zare, Mohammad Zoladl, Mohsen Naghavi, Bagher Larijani, Farshad Farzadfar

**Affiliations:** 1 Non-Communicable Diseases Research Center, Endocrinology and Metabolism Population Sciences Institute, Tehran University of Medical Sciences, Second Floor, No.10, Jalal Al-E-Ahmad Highway, Tehran, 1411713137 Iran; 2 Division of Pulmonary, Allergy, and Critical Care Medicine, University of Pittsburgh, Pittsburgh, PA USA; 3 Endocrinology and Metabolism Research Center, Endocrinology and Metabolism Clinical Sciences Institute, Tehran University of Medical Sciences, Tehran, Iran; 4 Institute for Health Metrics and Evaluation, University of Washington, Seattle, WA USA

**Keywords:** Breast cancer, Burden, Risk factor, Global Burden of Disease, Iran

## Abstract

**Background:**

Breast cancer (BC) is one of the most burdensome cancers worldwide. Despite advancements in diagnostic and treatment modalities, developing countries are still dealing with increasing burdens and existing disparities. This study provides estimates of BC burden and associated risk factors in Iran at the national and subnational levels over 30 years (1990–2019).

**Methods:**

Data on BC burden for Iran were retrieved from the Global Burden of Disease (GBD) study from 1990 to 2019. GBD estimation methods were applied to explore BC incidence, prevalence, deaths, disability-adjusted life years (DALYs), and attributable burden to risk factors based on the GBD risk factors hierarchy. Moreover, decomposition analysis was performed to find the contribution of population growth, aging, and cause-specific incidence in the total incidence change. Age-standardized rates (per 100,000 population) and 95% uncertainty intervals (UI) were reported based on sex, age, and socio-demographic index (SDI).

**Results:**

Age-standardized incidence rate (ASIR) increased from 18.8 (95% UI 15.3–24.1)/100,000 in 2019 to 34.0 (30.7–37.9)/100,000 in 2019 among females and from 0.2/100,000 (0.2–0.3) to 0.3/100,000 (0.3–0.4) among males. Age-standardized deaths rate (ASDR) increased slightly among females from 10.3 (8.2–13.6)/100,000 in 1990 to 11.9 (10.8–13.1)/100,000 in 2019 and remained almost the same among males—0.2/100,000 (0.1–0.2). Age-standardized DALYs rate also increased from 320.2 (265.4–405.4) to 368.7 (336.7–404.3) among females but decreased slightly in males from 4.5 (3.5–5.8) to 4.0 (3.5–4.5). Of the 417.6% increase in total incident cases from 1990–2019, 240.7% was related to cause-specific incidence. In both genders, the BC burden increased by age, including age groups under 50 before routine screening programs, and by SDI levels; the high and high-middle SDI regions had the highest BC burden in Iran. Based on the GBD risk factors hierarchy, high fasting plasma glucose (FPG) and alcohol were estimated to have the most and the least attributed DALYs for BC among females, respectively.

**Conclusions:**

BC burden increased from 1990 to 2019 in both genders, and considerable discrepancies were found among different provinces and SDI quintiles in Iran. These increasing trends appeared to be associated with social and economic developments and changes in demographic factors. Improvements in registry systems and diagnostic capacities were also probably responsible for these growing trends. Raising general awareness and improving screening programs, early detection measures, and equitable access to healthcare systems might be the initial steps to tackle the increasing trends.

**Supplementary Information:**

The online version contains supplementary material available at 10.1186/s13058-023-01633-4.

## Background

Cancer is responsible for a majority of deaths due to non-communicable diseases (NCDs) and is among the main barriers to increasing life expectancy and declining mortality rates in numerous nations worldwide [1, 2]. Breast cancer (BC) is one of the most burdensome cancers globally, responsible for most cancer deaths globally. BC is estimated to be the most frequently diagnosed cancer, considering both sexes, and it is the first leading cause of cancer death among females [3]. Although BC in males accounts for approximately 1% of all BCs globally (diagnosed in 1:1000 of males), its incidence seems to be increasing [4, 5].

Among all malignancies diagnosed, BC is ranked first in Iranian females, with an estimated age-standardized incidence rate (ASIR) of 44.0 (95% uncertainty interval [UI]: 36.4–52.0) 100,000 population in 2016, based on the National and Subnational Burden of Diseases (NASBOD) project report on BC [6]. Estimates from prior studies have revealed increasing trends in BC incidence and mortality rates in Iran and the globe. Recent studies predicted an increase of about 63% in the new BC cases in Iran in the year 2025 compared with 2016, as the leading cancer among Iranian females [7]. Based on previous reports, BC seems to involve Iranian males with a higher frequency than the world’s average (approximately 2.8% of primary BC cases in the country) [8]. Besides, the 5-year survival rate of BC was estimated to be relatively lower than in developed countries [9]. As a developing country, Iran has been in a transitional period and has faced numerous challenges due to modernization over recent decades [10]. The World Bank categorized Iran as a lower-middle-income and upper-middle-income country in 1990 and 2019, respectively [11]. Economic developments and demographic changes over recent years are believed to be the major causes of the growing trends in the burden of cancers [2, 12–14]. Geographic and climate diversities, cultural variations, genetic factors, lifestyles, socioeconomic status, and population aging and growth might also be potential factors leading to increasing trends and disparities in burden among provinces in Iran [2, 15].

Accordingly, it is worthwhile to estimate and analyze BC trends in Iran at national and subnational levels to have a clearer vision of the burden of BC on the Iranian population and public health. The most updated estimates around BC in Iran were limited to a recent study by Ataenia et al. up to 2016. The authors reported incidence, mortality, and years of life lost (YLLs) for females at the national and subnational levels [16]. In the current study, we aimed to report the most up-to-date estimates of BC burden in Iran according to the Global Burden of Disease (GBD) study 2019 by estimating all measures related to the burden of disease, including incidence, prevalence, mortality, YLLs, years lived with disability (YLDs), and disability-adjusted life year (DALYs), at the national and subnational level for both females and males, based on age groups and socio-demographic index (SDI) and to report the burden attributable to known risk factors over thirty years from 1990 to 2019. This analysis will also provide policymakers and national authorities with a broader vision and a whole picture, highlighting possible disparities in different regions of Iran as an upper-middle income country in the middle-east region, which helps them tailor up-to-date strategies and allocate resources more efficiently.

## Methods

### Overview of the GBD study

The GBD study provides comprehensive and comparable health estimates around the burden of diseases and injuries worldwide. The GBD study 2019 (the most up-to-date iteration of the GBD study) estimates the burden of 369 causes of death and disability and 87 risk factors and groups of risk factors at the global and regional levels for 204 countries and territories among females and males [17]. We obtained publicly available data from the Global Health Data Exchange (GHDx) query online tool (http://ghdx.healthdata.org/gbd-results-tool) regarding the burden of BC, including annual all-ages numbers and ASIR, age-standardized deaths rate (ASDR) and prevalence rate, YLLs, YLDs, and DALYs over 30 years, from 1990 to 2019, in Iran at the national and subnational levels. (All the provinces of Iran are shown on the national map in the Additional file 1: Fig. 1.) The source of data from scientific papers and disease registry systems that have been used by the GBD study 2019 to estimate the burden of BC in Iran and subnational districts is provided in Additional file 2: Table 1. International Classification of Diseases 10 (ICD-10) codes (C50-C50.629, C50.7, C50.8-C50.929, D05-D05.92, D24.0-D24.9, D48.6-D48.62, D49.3, Z12.3-Z12.39, Z80.3, Z85.3, and Z86.000) were utilized to identify BC, which is defined as the code B.1.14 in the GBD 2019 list of causes of death and disability. This study follows the Guidelines for Accurate and Transparent Health Estimates Reporting (GATHER) [18].

**Fig. 1:**
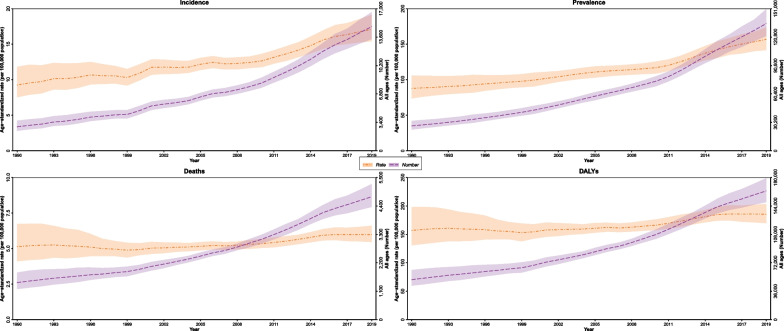
Time trends of breast cancer age-standardized incidence, prevalence, deaths and disability-adjusted life years (DALYs), rates (per 100,000 population) and numbers and 95% UIs in Iran, from 1990 to 2019 for both sexes

**Table 1:** Breast cancer incidence, prevalence, deaths, disability-adjusted life years (DALYs), years of life lost (YLLs), and years lived with disability (YLDs) by rates (per 100,000 population) and numbers with percent changes in Iran between 1990 and 2019, for both sexes, females, and males

Measure	Metric	1990			2019			% Change (1990 to 2019)		
		Both	Female	Male	Both	Female	Male	Both	Female	Male
Incidence	Rate^*^	9.3 (7.5 to 11.8)	18.8 (15.3 to 24.1)	0.2 (0.2 to 0.3)	17.1 (15.4 to 19.1)	34.0 (30.7 to 37.9)	0.3 (0.3 to 0.4)	84.8 (37.7 to 135)	81.2 (34.6 to 130.5)	28.6 (-13.2 to 80.4)
	Number^†^	2871 (2389 to 3608)	2835 (2352 to 3575)	36 (27 to 48)	14,863 (13,372 to 16,590)	14,743 (13,248 to 16,469)	120 (98 to 143)	417.6 (292.7 to 542.4)	420 (292.9 to 546)	234.2 (123.5 to 368.6)
Prevalence	Rate	87.9 (73.7 to 106.0)	178.6 (149.7 to 216.4)	2.0 (1.6 to 2.6)	156.9 (141.7 to 173.7)	312.1 (281.5 to 345.4)	2.8 (2.3 to 3.3)	78.5 (44.4 to 113.1)	74.7 (40.9 to 108.9)	37.3 (1.7 to 79.8)
	Number	26,458 (22,494 to 31,775)	26,160 (22,174 to 31,486)	298 (233 to 383)	135,257 (122,011 to 150,481)	134,187 (120,955 to 149,206)	1070 (886 to 1280)	411.2 (311.9 to 511.2)	412.9 (312.1 to 514.6)	259.2 (161.1 to 377.8)
Deaths	Rate	5.1 (4.1 to 6.7)	10.3 (8.2 to 13.6)	0.2 (0.1 to 0.2)	6.0 (5.5 to 6.6)	11.9 (10.8 to 13.1)	0.2 (0.1 to 0.2)	16.5 (-14.2 to 49.9)	14.9 (-15.1 to 47.7)	-11.4 (-37 to 22.6)
	Number	1436 (1186 to 1823)	1413 (1160 to 1796)	23 (18 to 30)	4760 (4362 to 5250)	4704 (4306 to 5192)	56 (48 to 63)	231.5 (151.4 to 314.3)	232.9 (151.2 to 318)	142.8 (72.5 to 232.2)
DALYs	Rate	157.0 (130.7 to 198.1)	320.2 (265.4 to 405.4)	4.5 (3.5 to 5.8)	185.6 (169.7 to 203.4)	368.7 (336.7 to 404.3)	4 (3.5 to 4.5)	18.2 (-9 to 45.9)	15.2 (-11.7 to 42.5)	-10.7 (-36.1 to 20.7)
	Number	50,647 (43,014 to 62,892)	49,919 (42,208 to 62,170)	728 (574 to 926)	163,091 (148,806 to 179,221)	161,486 (147,227 to 177,500)	1605 (1379 to 1790)	222 (150.1 to 290.7)	223.5 (150.8 to 293.2)	120.4 (58.2 to 197.1)
YLLs	Rate	150.8 (125.9 to 188.8)	307.6 (255.7 to 386.4)	4.3 (3.4 to 5.6)	174.1 (159.6 to 191.6)	345.9 (316.9 to 380.9)	3.8 (3.2 to 4.2)	15.4 (-12 to 43.2)	12.5 (-14.6 to 40)	-12.7 (-37.9 to 18.4)
	Number	48,718 (41,357 to 60,770)	48,016 (40,680 to 59,999)	702 (550 to 895)	153,082 (140,075 to 168,502)	151,570 (138,503 to 166,910)	1512 (1296 to 1689)	214.2 (144.5 to 283.2)	215.7 (144.8 to 285.4)	115.3 (54.6 to 189.9)
YLDs	Rate	6.2 (4.2 to 9.0)	12.6 (8.5 to 18.4)	0.2 (0.1 to 0.3)	11.5 (8.0 to 15.8)	22.8 (15.9 to 31.4)	0.2 (0.2 to 0.3)	84.7 (42.6 to 129.7)	81 (39.4 to 125.5)	38.2 (-3.2 to 90)
	Number	1929 (1304 to 2771)	1903 (1285 to 2739)	26 (17 to 38)	10,009 (6956 to 13,776)	9916 (6887 to 13,657)	93 (62 to 133)	419 (305 to 536.8)	421.2 (304.4 to 541.9)	257.2 (146.6 to 391.9)

Data in parentheses are 95% uncertainty intervals

^*^Age-standardized rate (per 100,000)

^**†**^All ages

### Cancer burden estimation

The GBD study employs various estimation and modeling tools to provide comparable results around the global burden of diseases and injuries. The detailed process of burden estimations regarding primary values, including incidence, prevalence, mortality, YLLs, YLDs, and DALYs in the GBD 2019 study, has been described in detail elsewhere [17]. Here, we briefly describe the estimation methods of these primary values by the GBD study.

In some regions, cancer mortality data are scarce, while cancer incidence data are more accessible through population-based cancer registries. So, to maximize data informing mortality models, the GBD study transforms incidence data into cancer mortality estimates using modeled mortality-to-incidence ratios (MIRs). First, incidence and mortality data obtained from cancer registries (inputs) were processed to generate crude MIRs. Final cancer cause-specific MIRs were estimated using a spatiotemporal Gaussian process regression (ST-GPR) by utilizing Healthcare Access and Quality (HAQ) index, age, and sex as covariates. The MIR estimates using the ST-GPR model were then multiplied with incidence data to generate crude mortality estimates. Subsequently, MIR-transformed mortality data were pooled with mortality data obtained from vital registries and verbal autopsies and were used as inputs in cancer-specific Cause of Death Ensemble models (CODEm) [19–21]. CODEm produced models of deaths for each cause, sex and age group, and time point within the GBD study by selecting models based on out-of-sample predictive validity. Eventually, the predicted mortality estimates were adjusted to align with independently modeled estimates for each sex-age group-location-year [17].

Incidence estimates were generated from modeled cancer mortality estimates using BC MIRs for each sex, age group, location, and year. Prevalence of BC was modeled using incidence, mortality background, and estimated relative survival curves and their correlation with modeled MIRs. For the estimation of YLDs, the estimated prevalence was multiplied by the corresponding BC-specific disability weight. Disability weight for each cause describes the severity of health loss with that specific cause, ranging from 0 (full health) to 1 (death) [17]. Accordingly, YLLs were calculated by multiplying the difference between the number of deaths due to BC in a specific age group by the remaining standard life expectancy at the age of death. DALYs also represent the sum of YLDs and YLLs [17, 22].

### Socio-demographic index (SDI)

We categorized Iranian subnational districts by SDI into five quintiles (low, low-middle, middle, high-middle, and high SDI). We calculated this index according to the previously described methods based on the lag distributed income per capita, average educational attainment (schooling years) for individuals over 15 years, and the fertility rate in females under 25 years, which could be found elsewhere [23].

### Decomposition analysis

We performed decomposition analysis in this study to find the attribution of population growth, population aging, and BC incidence rate in the total change of BC incident cases between 1990 and 2019. We performed this analysis based on the previously described method, which could be found elsewhere [24]. Briefly, two steps were taken to analyze this attribution: in the first step, the age structure, sex structure, and BC rates of 1990 were applied to the total population of 2019. The difference between this step and the total number of new cases in 1990 was attributed to population growth. In the second step, the BC incidence rate from 1990 was applied to the age structure, sex structure, and population size of 2019. The difference between these two steps was attributed to population aging. Eventually, differences between the total number of incident cases in 2019 and the second step were attributed to true changes in the BC-specific incidence rate.

### Mortality-to-incidence ratio (MIR)

To report the age-standardized MIRs in this study, we divided ASDR by ASIR, obtained from the GBD results for BC, for each sex-age group-location-year combination as follows [25]:

$$\mathrm{MIR} \, (\mathrm{Mortality\!-\!to\!-\!Incidence\,Ratio})=\frac{\mathrm{Age\!-\!standardized\,deaths\,rate} \, (\mathrm{ASDR})}{\mathrm{Age\!-\!standardized\,incidence\,rate} \, (\mathrm{ASIR})}$$

To calculate the MIRs, we used the final estimated values for incidence and mortality (outputs) after the modeling systems utilized by the original GBD estimation processes described earlier in the methods section. The MIR calculation was validated previously for BC in the country using local data as part of the NASBOD project and at the global scale using GBD 2019 data [6, 26].

### Estimation of the breast cancer burden attributable to risk factors

The GBD study has incorporated a comparative risk assessment framework comprising a risk factor hierarchy categorized into three main groups (environmental and occupational, behavioral, and metabolic risks) and four levels to assess the attributable burden to different risk factors. Counting all specific risk factors and aggregates, the GBD study 2019 includes 87 risks or clusters of risks [27]. Risk factors for BC were included in this study based on the GBD risk factor hierarchy at the 4th level, as follows: alcohol use, smoking, secondhand smoke, high fasting plasma glucose (FPG) [28], diet high in red meat, high body-mass index (BMI), and low physical activity. These risk factors have been included in the last iteration of the GBD study (2019) as the risk factors known to be associated with BC; for more details on the inclusion process of the risk factors, refer to the original GBD 2019 risk factors summary paper [27].

Detailed methods of estimating risk factors attributed burden could be found elsewhere [27]. Briefly, the GBD comparative risk assessment framework followed a six-step approach to compute the fraction of cancer-specific burden attributable to risk factors in the GBD study 2019. In the first step, risk factors with convincing or probable evidence for a causal association were identified using the World Cancer Research Fund criteria [29]. In the next step, relative risks (RRs) were estimated for each risk-outcome pair as a function of exposure. The meta-analytic approach was updated for a selected set of continuous risk factors in the GBD study 2019, using the meta-regression-Bayesian, regularized, trimmed (MR-BRT) mixed-effects model. In the third step, exposure levels and distributions of risk factors were modeled for each sex-age-location-year combination using existing data from published studies, household surveys, censuses, administrative data, ground monitor data, or remote sensing data. The GBD study modeled risk factor exposure levels using either a Bayesian meta-regression modeling tool (DisMod-MR 2.1), a flexible approach incorporating age- and sex-specific data, or ST-GPR, which was the preferred approach when exposure was stable across different age groups. In the fourth step, the theoretical minimum risk exposure level (TMREL) was identified for each risk factor. In the next step, the population attributable fraction (PAF) was computed for each risk-outcome pair for each sex-age-location-year based on the RRs, exposure levels, and the TMREL. Then, correction for PAF overestimation, which might occur if the independence of certain risk factors was assumed, was performed using a mediation matrix. In the last step, to estimate the BC burden attributable to each risk factor, YLLs, YLDs, and deaths were multiplied by the corresponding PAF of each risk factor for each sex-age-location-year combination. The sum of YLLs and YLDs was used to estimate the DALYs attributable to risk factors [27, 30, 31].

### Statistical analysis

Age groups for BC were defined from 15 to + 80 years on a 5-year basis. Crude all age numbers for the primary values have been reported in this study, and the rates have been reported per 100,000 population. Age-standardized rates were estimated using the direct method of standardization and the GBD world population standard and were reported per 100,000 population [22]. We calculated and reported percent changes (PCs) for all the measures between 1990 and 2019. Annual percent changes (APC) were also generated to measure the trend of the age-standardized rates changes over the study period. To this end, we applied a regression line to the natural logarithm of the age-standardized rates, and 95% confidence intervals were also reported. Besides, we performed Pearson’s correlation tests to evaluate the correlation between different burden measures and SDI and between females and males in different provinces regarding age-standardized rates for each measure. We performed all analyses and data visualizations using Stata version 13 (StataCorp LLC, TX, USA) and R version 3.5.0; statistical codes are publicly available elsewhere [17]. For all estimates, the 95% UI is reported by obtaining 1000 samples of the posterior distribution and marking the 25th and 975th ranked values across all 1000 draws, which covers the proportion of data point mean values that fall between the 2.5th and 97.5th percentile of the draws of the fit values [17].

## Results

### Breast cancer incidence and prevalence in Iran

Overall, BC incidence case number among females increased by 5.2-fold from 2,835 (95% UI: 2352–3575) in 1990 to 14,743 (13,248–16,469) in 2019 in Iran. Similarly, ASIR for females increased from 18.8 (15.3–24.1)/100,000 to 34.0 (30.7–37.9)/100,000, with 81.2% (34.6–130.5) PC and 1.87 (1.69–2.05) APC in the same period (Table 1 and Additional file 3: Table 2). Among males, the number of incident cases also increased from 36 (27–48) to 120 (98–143) over this period, with 28.6% (− 13.2–80.4) PC and 0.39 (0.05–0.74) APC in their ASIR. Males comprised approximately 1.2% of the total incidence in 1990 (36 [27–48]/2871 [2389–3608]), while approximately 0.8% of all incident cases in 2019 (120 [98–143]/14,863 [13,372–16,590]) were male BC. Annual trends illustrated in Fig. 1 show ASIR trends and the all-ages number of incidence from 1990 to 2019. The overall age-standardized prevalence rate of BC in Iran was estimated as 312.e1 (281.5–345.4)/100,000 in 2019 for females, which was remarkably higher than its rate in 1990, estimated as 178.6 (149.7–216.4)/100,000. For males, this rate increased from 2.0 (1.6–2.6)/100,000 to 2.8 (2.3–3.3)/100,000 over this period.

**Table 2:** Decomposition analysis on changes in breast cancer incident cases in Iran and its 31 provinces, for both sexes, females, and males, between 1990 and 2019 (provinces have been sorted alphabetically)

Location		Sex	New cases		Expected new cases in 2019		% 1990—2019 new cases change cause			% 1990—2019 new cases overall change
			1990	2019	Population growth	Population growth + Aging	Population growth	Age structure change	Incidence rate change	
Iran (Islamic Republic of)		Both	2871	14,863	4135	7952	44%	133%	240.7%	417.6%
		Female	2835	14,743	4110	8050	44.9%	139%	236%	420%
		Male	36	120	51	93	43.1%	117.3%	73.8%	234.2%
Subnational	Alborz	Both	90	644	177	384	95.4%	229.8%	287.3%	612.5%
		Female	89	637	177	398	99%	247.5%	268.1%	614.6%
		Male	1	7	3	5	92%	218.5%	157.2%	467.7%
	Ardebil	Both	48	189	53	111	10.9%	120.9%	162.6%	294.4%
		Female	47	187	52	115	10.9%	131.3%	153.9%	296%
		Male	1	1	1	1	10.8%	94.9%	51.7%	157.5%
	Bushehr	Both	31	196	52	99	71.4%	154%	317.2%	542.7%
		Female	30	195	50	96	64.7%	152.6%	327.4%	544.8%
		Male	0	1	0	1	78%	123.3%	106.8%	308%
	Chahar Mahaal and Bakhtiari	Both	27	116	37	77	35.3%	143.3%	143%	321.6%
		Female	27	115	37	79	36%	153.7%	133.5%	323.2%
		Male	0	1	0	1	34.8%	109.1%	38%	181.8%
	East Azarbayejan	Both	159	798	188	365	18.2%	110.4%	272%	400.6%
		Female	157	791	186	369	18.1%	116.2%	268.1%	402.5%
		Male	2	7	2	4	18.2%	97.8%	138.9%	254.9%
	Fars	Both	158	1002	215	440	35.9%	142.4%	355.3%	533.6%
		Female	156	994	213	443	36.5%	146.8%	353.1%	536.4%
		Male	2	8	3	5	35.4%	124.7%	148.1%	308.2%
	Gilan	Both	137	646	153	315	11.6%	118.9%	241.9%	372.4%
		Female	135	640	151	311	11.7%	119.2%	244.1%	375%
		Male	2	6	2	5	11.4%	122.4%	68.6%	202.5%
	Golestan	Both	48	308	69	134	43.9%	136.1%	362.3%	542.3%
		Female	47	306	68	137	44.3%	145.4%	355.8%	545.5%
		Male	1	2	1	1	43.5%	109.4%	117.6%	270.6%
	Hamadan	Both	78	284	80	161	3.4%	104.5%	158.3%	266.3%
		Female	77	283	81	164	5.1%	107.9%	154.6%	267.7%
		Male	1	2	1	2	1.8%	92.4%	36.5%	130.8%
	Hormozgan	Both	27	196	55	90	105%	131.4%	398.8%	635.2%
		Female	26	193	53	91	105.1%	147.6%	396.3%	649%
		Male	1	3	2	2	104.9%	78%	28.1%	210.9%
	Ilam	Both	12	94	16	37	32%	170%	472.3%	674.3%
		Female	12	93	16	40	33.7%	199.2%	448%	680.8%
		Male	0	1	0	1	30.4%	119.7%	173.7%	323.8%
	Isfahan	Both	193	1069	264	536	37.1%	140.7%	276.7%	454.4%
		Female	191	1059	266	535	39.7%	140.9%	275.2%	455.9%
		Male	2	10	3	6	34.6%	137.5%	158.9%	331%
	Kerman	Both	72	407	128	225	78.3%	135.5%	254.5%	468.2%
		Female	71	404	124	224	76%	140.5%	254.7%	471.2%
		Male	1	4	2	3	80.5%	103.6%	76.4%	260.6%
	Kermanshah	Both	66	330	77	161	16.3%	129.2%	255.8%	401.4%
		Female	65	327	77	171	18.7%	144.7%	241.3%	404.7%
		Male	1	3	1	2	14.1%	106%	63.4%	183.5%
	Khorasan-e-Razavi	Both	190	988	269	478	41.4%	109.8%	267.7%	418.9%
		Female	188	982	267	488	41.7%	117.4%	262.4%	421.5%
		Male	2	6	3	5	41.1%	85.2%	61.2%	187.5%
	Khuzestan	Both	120	791	183	355	52.2%	143.4%	362.5%	558.1%
		Female	118	786	181	356	53.2%	147.4%	363%	563.6%
		Male	2	5	3	5	51.3%	121.2%	27.1%	199.6%
	Kohgiluyeh and Boyer-Ahmad	Both	10	78	15	31	51.9%	159.6%	457.1%	668.6%
		Female	10	77	15	31	51.9%	171.9%	464.6%	688.4%
		Male	0	1	1	1	52%	118.9%	16.7%	187.7%
	Kurdistan	Both	48	230	64	125	33.8%	127.4%	221.8%	383.1%
		Female	47	228	63	130	34.2%	141.6%	210.2%	386%
		Male	1	2	1	2	33.5%	100.1%	39.9%	173.4%
	Lorestan	Both	53	239	60	128	14.3%	127.7%	212.1%	354.1%
		Female	52	237	60	135	15.5%	145.2%	197.5%	358.2%
		Male	1	2	1	2	13.2%	97.1%	27.9%	138.1%
	Markazi	Both	62	255	74	146	19.2%	117.7%	176.2%	313.1%
		Female	61	253	72	143	18.5%	116%	180.1%	314.6%
		Male	1	2	1	2	19.9%	105.1%	64%	188.9%
	Mazandaran	Both	146	881	192	410	31.5%	150.1%	322.7%	504.3%
		Female	144	875	189	409	31.3%	152.5%	323%	506.8%
		Male	2	5	2	4	31.6%	147.3%	85.3%	264.2%
	North Khorasan	Both	23	115	31	56	37.5%	109%	260.8%	407.3%
		Female	22	114	30	57	37.6%	118.8%	258.2%	414.6%
		Male	1	1	1	1	37.3%	89.6%	-0.4%	126.6%
	Qazvin	Both	34	221	48	98	39.9%	143.6%	358.2%	541.6%
		Female	34	220	48	97	40.4%	145.3%	361.5%	547.2%
		Male	1	2	1	1	39.3%	113.4%	57.8%	210.5%
	Qom	Both	36	240	67	128	84.1%	168.7%	307.8%	560.5%
		Female	36	238	66	126	85.8%	169.8%	313.4%	569%
		Male	1	3	2	3	82.4%	150.5%	-14.6%	218.3%
	Semnan	Both	28	148	43	72	54.9%	106.9%	272.1%	433.9%
		Female	27	145	43	71	57.1%	103.1%	275.8%	436%
		Male	1	2	1	1	52.9%	88.5%	186.4%	327.8%
	Sistan and Baluchistan	Both	35	183	71	94	101.9%	65.7%	250.9%	418.5%
		Female	35	181	70	101	102%	92%	230.7%	424.7%
		Male	1	1	1	2	101.9%	27%	-15.3%	113.6%
	South Khorasan	Both	32	121	39	62	24.2%	73.3%	185.4%	282.9%
		Female	31	120	38	65	24.1%	85.2%	177%	286.3%
		Male	1	1	1	1	24.4%	52.7%	3%	80.1%
	Tehran	Both	750	3206	1228	2351	63.8%	149.8%	114.1%	327.7%
		Female	743	3185	1243	2396	67.3%	155.1%	106.3%	328.7%
		Male	7	21	11	21	60.5%	159.8%	-8.1%	212.2%
	West Azarbayejan	Both	96	514	140	262	46.6%	126.4%	263.2%	436.3%
		Female	95	511	139	268	46.8%	135.9%	255.9%	438.5%
		Male	1	3	1	2	46.5%	100.7%	60.4%	207.6%
	Yazd	Both	38	234	65	112	69.2%	121.2%	319.7%	510.1%
		Female	38	230	64	106	70.3%	112.6%	328.9%	511.8%
		Male	1	4	1	2	68.1%	107.5%	254.2%	429.8%
	Zanjan	Both	25	138	31	63	21.6%	126%	294.6%	442.1%
		Female	25	137	31	64	22.1%	131.2%	291.7%	445%
		Male	0	1	0	1	21%	95.5%	111.8%	228.3%

**Fig. 2:**
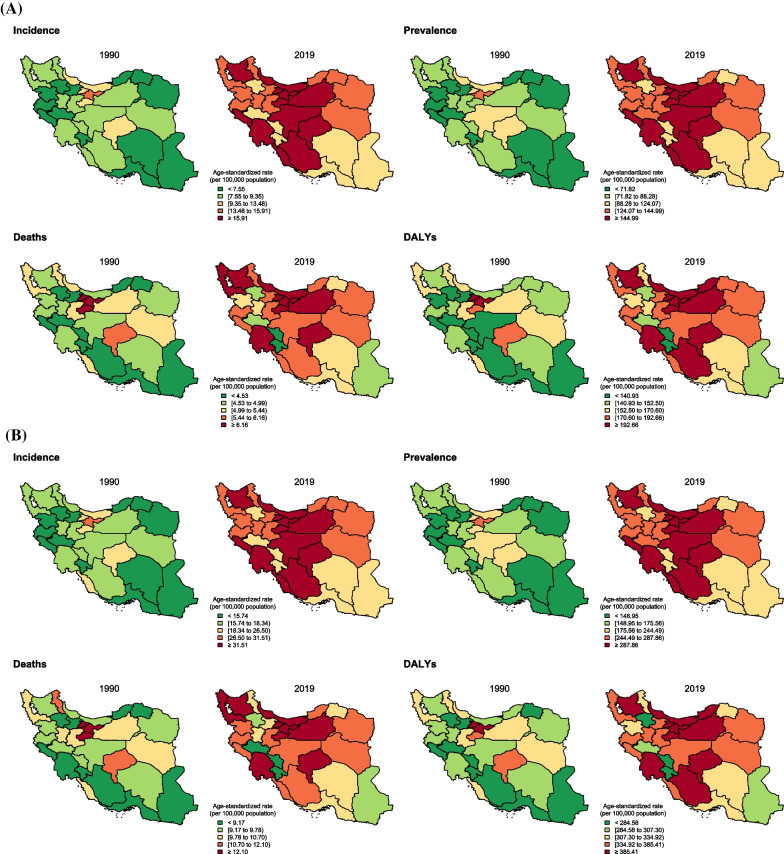

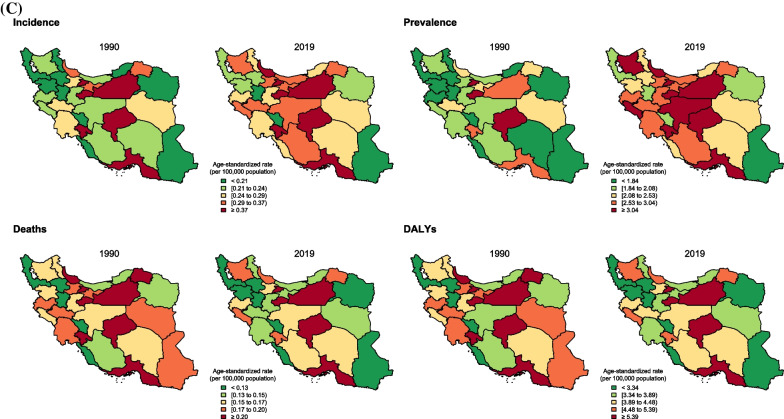
Breast cancer age-standardized incidence, prevalence, deaths, and disability-adjusted life years (DALYs) rates (per 100,000) and their changes between 1990 and 2019 in Iran at the subnational level for **A** both sexes, **B** females, and **C** males

At the subnational level, all provinces in Iran experienced a remarkable increase in ASIR for both females and males (Fig. 2A, B, and C), and the average ASIR, considering both sexes, almost doubled over the study period—Iran’s provinces are illustrated on the national map in Additional file 1: Fig. 1. In 1990, the highest and lowest ASIR for females among all provinces were 30.3 (21.0–43.0)/100,000 (Tehran) and 10.4 (6.6–16.2)/100,000 (Kohgiluyeh and Boyer-Ahmad), respectively, while in 2019, the highest and lowest rates were estimated as 41.8 (33.3–52.6)/100,000 (Alborz) and 20.0 (15.5–25.7)/100,000 (Sistan and Baluchistan), respectively. Taking both sexes into consideration, the maximum and minimum ASIR were 14.9 (10.4–21.2)/100,000 (Tehran) and 5.1 (3.3–7.9)/100,000 (Kohgiluyeh and Boyer-Ahmad) in 1990 and 20.8 (16.5–25.8)/100,000 (Mazandaran) and 10.1 (7.8–12.9)/100,000 (Sistan and Baluchistan) in 2019, respectively, see Additional file 4: Fig. 2A, Additional file 5: Fig. 2B, and Additional file 6: Fig. 2C. Almost all provinces experienced this remarkable increase in ASIR for females and males (Additional file 7: Table 3).

**Fig. 3:**
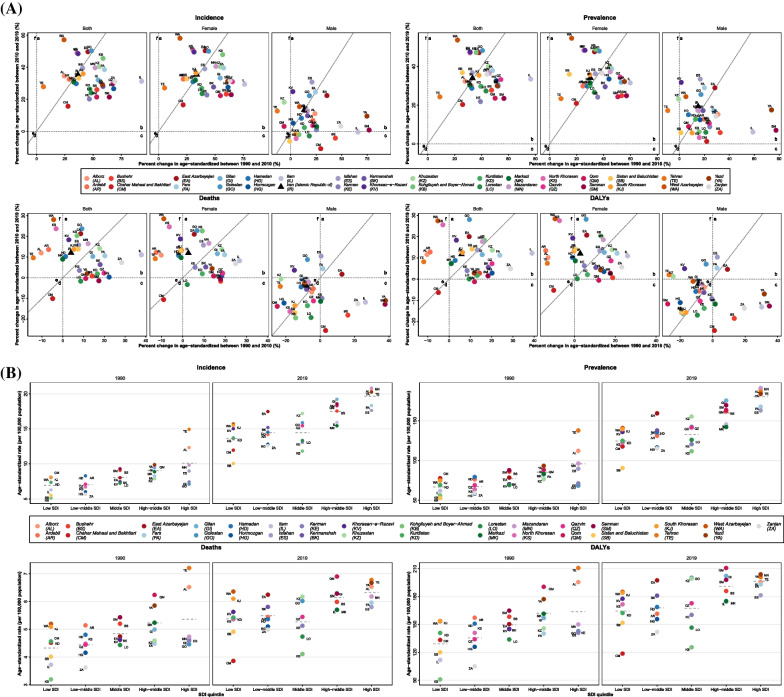
**A** Breast cancer age-standardized incidence, prevalence, deaths, and disability-adjusted life years (DALYs) rates percent changes (%) over two major periods (*X*-axis: the first period, from 1990 to 2010; *Y*-axis: the second period, from 2010 to 2019) in Iran and its 31 provinces for both sexes, females, and males. The oblique line in each graph shows the equal percent change in the second period as the first period. **B** Breast cancer age-standardized incidence, prevalence, deaths, and disability-adjusted life years (DALYs) rates (per 100,000 population) based on the subnational socio-demographic index (SDI) quintiles in 1990 and 2019 for both sexes. The dotted line shows the mean rate within each SDI quintile

In order to evaluate changes in ASIR from 1990 to 2019, PCs were assessed in two major periods, based on the two main periods over the original GBD study 2019 [17]: 1) between 1990 and 2010; 2) between 2010 and 2019 (Fig. 3A and Additional file 8: Table 4). In Tehran, the capital of Iran, the total (both sexes) PC of ASIR in the second period was almost sixfold higher than the first period, with the overall ASIR PC of 35.5% (− 12.9–111.0) from 1990 to 2019—not statistically significant increase. Regarding sexes, no province at the subnational level had a negative ASIR PC in females in both periods, while some provinces had negative ASIR PCs in males during the second period, illustrated in Fig. 3A. The highest negative ASIR PC for males was − 7.1% (− 47.9–75.2; Sistan and Baluchistan). On the other hand, the highest positive ASIR PC for females was 131.8% (30.5–310.2; Kohgiluyeh and Boyer-Ahmad), and Ilam had the highest APC in incidence rate of 3.31 (3.11–3.51) among all provinces between 1990 and 2019 (Additional file 3: Table 2).

Moreover, the highest age-standardized prevalence rates at the subnational level in 2019 were estimated at 383.7 (311.1–470.1)/100,000 (Alborz) for females and 6.9 (5.0–10.0)/100,000 (Yazd) for males, while the lowest age-standardized prevalence rates for females and males were 179.6 (146.6–223.7)/100,000 and 1.8 (1.3–2.4)/100,000, respectively (both in Sistan and Baluchistan); see Additional file 7: Table 3.

**Table 3:** Breast cancer incidence, prevalence, deaths, disability-adjusted life years (DALYs), years of life lost (YLLs), and years lived with disability (YLDs) by rates (per 100,000 population) and numbers with percent changes in Iran between 1990 and 2019, for both sexes, females, and males, based on the age groups < 50 (15–49), 50–69, and + 70

Measure	Metric	Age group (years)	1990			2019			% Change (1990 to 2019)		
			Both	Female	Male	Both	Female	Male	Both	Female	Male
Incidence	Rate (per 100,000)	15 to 49	4.9 (4.2 to 6)	9.9 (8.4 to 12.1)	0.1 (0.1 to 0.1)	14.9 (13.1 to 16.9)	30.2 (26.5 to 34.2)	0.1 (0.1 to 0.2)	202.8 (135.7 to 274.6)	204.9 (136.9 to 277.1)	81.7 (20.8 to 166.1)
		50 to 69	24.7 (19.8 to 32.1)	52.3 (41.8 to 68.4)	0.7 (0.5 to 1)	45.4 (40.2 to 51.7)	89.5 (79.2 to 102.2)	0.9 (0.7 to 1.2)	83.9 (34.4 to 138.8)	71.1 (24.3 to 122.8)	26.9 (− 22.1 to 93.5)
		70 +	29.5 (20.1 to 45.6)	58.5 (39.3 to 91)	1.2 (0.9 to 1.7)	53.8 (46.6 to 62.1)	105.4 (91.3 to 121.5)	1.7 (1.3 to 2.1)	82.3 (11.1 to 173.4)	80.2 (9 to 174.8)	33.6 (− 13 to 101.4)
	Number	15 to 49	1298 (1104 to 1591)	1289 (1095 to 1582)	9 (7 to 12)	7029 (6175 to 7967)	6999 (6144 to 7942)	30 (23 to 39)	441.4 (321.4 to 569.8)	442.9 (321.8 to 571.5)	226.3 (117 to 377.8)
		50 to 69	1289 (1038 to 1678)	1269 (1014 to 1659)	21 (15 to 29)	5966 (5285 to 6805)	5904 (5227 to 6741)	62 (47 to 81)	362.7 (238.1 to 500.7)	365.4 (238 to 505.8)	196.9 (82.1 to 352.5)
		70 +	284 (193 to 438)	278 (186 to 431)	6 (4 to 8)	1868 (1616 to 2156)	1839 (1592 to 2118)	29 (22 to 36)	558.6 (301.6 to 888.1)	562.6 (301.1 to 910.6)	374.5 (208.8 to 615.2)
Prevalence	Rate (per 100,000)	15 to 49	40.7 (34.7 to 50)	82 (69.7 to 100.7)	0.6 (0.4 to 0.8)	129 (112.9 to 146.7)	261.6 (228.9 to 297.9)	1.1 (0.9 to 1.5)	216.6 (150.1 to 291.2)	218.9 (151.5 to 295.1)	87.9 (29.8 to 170.5)
		50 to 69	239.1 (196.9 to 295.2)	508.7 (417.6 to 630.1)	5.8 (4.3 to 8.1)	431.7 (383 to 487.5)	852.6 (755.6 to 963.4)	8.1 (6.2 to 10.8)	80.5 (41.4 to 120.9)	67.6 (31.2 to 105.6)	38.8 (− 9.1 to 104.6)
		70 +	331 (244.7 to 445.8)	659.5 (487.6 to 890.9)	10.9 (7.8 to 14.9)	503.8 (434 to 587.7)	987.7 (850.2 to 1152.6)	15.3 (11.8 to 19.6)	52.2 (16.6 to 94.9)	49.8 (14.3 to 92.6)	40.6 (5.3 to 88.8)
	Number	15 to 49	10,772 (9164 to 13,217)	10,690 (9084 to 13,116)	82 (60 to 107)	60,977 (53,389 to 69,342)	60,701 (53,111 to 69,111)	276 (209 to 367)	466.1 (347.3 to 599.6)	467.8 (347.9 to 603.5)	237.3 (133.1 to 385.6)
		50 to 69	12,506 (10,296 to 15,435)	12,342 (10,132 to 15,289)	163 (121 to 228)	56,790 (50,377 to 64,124)	56,260 (49,856 to 63,567)	530 (407 to 705)	354.1 (255.6 to 455.6)	355.8 (256.8 to 459.1)	224.8 (112.6 to 378.5)
		70 +	3180 (2351 to 4283)	3127 (2312 to 4224)	53 (38 to 73)	17,491 (15,068 to 20,406)	17,226 (14,828 to 20,101)	264 (203 to 339)	450 (321.3 to 604.4)	450.8 (320.6 to 608.2)	399.1 (273.8 to 570.4)
Deaths	Rate (per 100,000)	15 to 49	1.8 (1.6 to 2.1)	3.6 (3.1 to 4.3)	0 (0 to 0)	3.1 (2.8 to 3.4)	6.3 (5.7 to 6.9)	0 (0 to 0)	74.6 (43.4 to 105.3)	75.9 (44.1 to 106.9)	15.6 (− 17.4 to 53)
		50 to 69	14 (11.3 to 18)	29.6 (23.9 to 38.3)	0.5 (0.4 to 0.6)	16.7 (15.2 to 18.3)	32.8 (30 to 36.1)	0.4 (0.3 to 0.5)	19 (− 10.8 to 50.6)	10.8 (− 17.3 to 40.5)	− 13.9 (− 41.7 to 21.6)
		70 +	24.2 (16.8 to 37.1)	48 (33.1 to 73.8)	1.1 (0.8 to 1.4)	31.6 (27.3 to 35.8)	61.7 (53.4 to 69.7)	1.1 (0.9 to 1.3)	30.4 (− 19.9 to 91.8)	28.7 (− 21.5 to 90.5)	7.3 (− 26.9 to 56)
	Number	15 to 49	472 (413 to 562)	467 (409 to 558)	5 (4 to 6)	1473 (1337 to 1616)	1464 (1329 to 1608)	9 (8 to 11)	212.2 (156.4 to 267.1)	213.2 (156.7 to 268.5)	107.6 (48.3 to 174.7)
		50 to 69	731 (593 to 944)	718 (579 to 929)	13 (10 to 18)	2191 (2000 to 2409)	2164 (1976 to 2380)	27 (23 to 31)	199.5 (124.3 to 278.7)	201.3 (124.9 to 282.1)	101.5 (36.4 to 184.5)
		70 +	233 (161 to 356)	227 (157 to 350)	5 (4 to 7)	1096 (949 to 1243)	1077 (932 to 1215)	20 (16 to 23)	371.2 (189.6 to 593.1)	373.2 (188.6 to 600.7)	281 (159.6 to 453.8)
DALYs	Rate (per 100,000)	15 to 49	89.7 (78.2 to 107.8)	180.1 (156.8 to 216.8)	1.8 (1.5 to 2.2)	159.6 (144.7 to 175)	323.1 (292.8 to 354.4)	2 (1.7 to 2.3)	77.9 (45.8 to 109)	79.3 (46.9 to 110.9)	13.5 (− 17 to 46.9)
		50 to 69	441.8 (362.7 to 567.4)	935.4 (764.3 to 1207.1)	14.5 (10.8 to 19.1)	537.9 (489 to 592.6)	1059.6 (963.5 to 1168.6)	12.8 (10.8 to 15)	21.7 (− 7.5 to 52.5)	13.3 (− 14.3 to 41.8)	− 11.6 (− 40.1 to 24.8)
		70 +	398.8 (280.4 to 603.8)	790.4 (550.4 to 1206.5)	17.3 (12.4 to 22.7)	486.5 (427.3 to 547.9)	952.4 (836.3 to 1068.6)	16.2 (13 to 18.8)	22 (− 23.1 to 76.5)	20.5 (− 24.6 to 75.7)	− 6.4 (− 35.9 to 36.1)
	Number	15 to 49	23,711 (20,665 to 28,501)	23,474 (20,437 to 28,245)	238 (195 to 295)	75,447 (68,396 to 82,743)	74,963 (67,935 to 82,218)	484 (413 to 560)	218.2 (160.8 to 273.6)	219.3 (161.6 to 275.6)	103.8 (49 to 163.8)
		50 to 69	23,104 (18,966 to 29,669)	22,697 (18,544 to 29,290)	407 (302 to 536)	70,754 (64,321 to 77,957)	69,913 (63,572 to 77,109)	841 (708 to 980)	206.2 (132.7 to 283.6)	208 (133 to 285.7)	106.8 (40.1 to 191.9)
		70 +	3832 (2694 to 5802)	3748 (2610 to 5721)	84 (61 to 110)	16,890 (14,835 to 19,024)	16,611 (14,585 to 18,636)	280 (225 to 325)	340.8 (178.1 to 537.9)	343.2 (177.4 to 546.3)	232.5 (127.8 to 383.2)
YLLs	Rate (per 100,000)	15 to 49	86.4 (75.4 to 103.3)	173.5 (151.1 to 207.6)	1.7 (1.4 to 2.1)	149.5 (135.3 to 163.9)	302.5 (273.9 to 331.8)	1.9 (1.6 to 2.2)	73 (41.2 to 103.7)	74.4 (42.4 to 105.5)	11.2 (− 18.9 to 44.1)
		50 to 69	425.4 (348.6 to 545.3)	900.6 (733.9 to 1157.9)	14 (10.4 to 18.4)	507.6 (462.8 to 559.4)	999.8 (911.5 to 1099.6)	12.1 (10.1 to 14.1)	19.3 (− 9.7 to 49.4)	11 (− 16.3 to 39.5)	− 13.4 (− 41.4 to 22.1)
		70 +	378.3 (265 to 574.1)	749.8 (520.1 to 1146.2)	16.4 (11.8 to 21.5)	451 (397.6 to 509.4)	883.2 (779.2 to 993.6)	14.8 (12 to 17.4)	19.2 (− 25.4 to 74)	17.8 (− 26.8 to 73.4)	− 9.2 (− 38.9 to 32.2)
	Number	15 to 49	22,840 (19,942 to 27,302)	22,610 (19,695 to 27,053)	230 (188 to 287)	70,658 (63,982 to 77,471)	70,197 (63,544 to 76,982)	460 (394 to 533)	209.4 (152.5 to 264.2)	210.5 (153.6 to 266)	99.7 (45.7 to 158.8)
		50 to 69	22,243 (18,228 to 28,515)	21,851 (17,807 to 28,096)	392 (291 to 515)	66,764 (60,880 to 73,580)	65,970 (60,141 to 72,553)	795 (665 to 922)	200.2 (127.2 to 275.9)	201.9 (127.6 to 279.5)	102.7 (37 to 185.7)
		70 +	3635 (2546 to 5516)	3555 (2466 to 5435)	80 (57 to 105)	15,660 (13,805 to 17,687)	15,403 (13,589 to 17,329)	257 (207 to 300)	330.8 (169.5 to 528.8)	333.2 (169.3 to 537.7)	222.4 (116.9 to 369.3)
YLDs	Rate (per 100,000)	15 to 49	3.3 (2.3 to 4.7)	6.6 (4.5 to 9.5)	0.1 (0 to 0.1)	10.1 (6.9 to 14.1)	20.5 (13.9 to 28.5)	0.1 (0.1 to 0.2)	207.5 (138.6 to 282.5)	209.7 (139.9 to 285.9)	88.6 (25.5 to 175.5)
		50 to 69	16.5 (10.9 to 24.6)	34.9 (22.9 to 52.2)	0.5 (0.3 to 0.8)	30.3 (20.7 to 42)	59.8 (40.9 to 82.7)	0.7 (0.5 to 1.1)	84.3 (36.3 to 135.4)	71.4 (26.2 to 119.5)	36 (− 15.5 to 104.1)
		70 +	20.5 (12.3 to 33)	40.6 (24.2 to 65.7)	0.9 (0.6 to 1.4)	35.4 (25.2 to 47.7)	69.2 (49.2 to 93)	1.3 (0.8 to 2)	72.9 (17 to 144.8)	70.6 (14.7 to 144.2)	44.5 (− 3.7 to 110.9)
	Number	15 to 49	871 (598 to 1244)	864 (593 to 1236)	7 (4 to 10)	4789 (3252 to 6655)	4766 (3237 to 6618)	24 (15 to 36)	449.8 (326.6 to 584)	451.5 (327.2 to 587.2)	238.6 (125.4 to 394.7)
		50 to 69	861 (568 to 1284)	846 (556 to 1267)	15 (9 to 22)	3990 (2726 to 5523)	3943 (2700 to 5455)	46 (30 to 69)	363.5 (242.9 to 492.1)	366 (243.2 to 497)	218.2 (97.6 to 377.5)
		70 +	197 (118 to 317)	192 (115 to 312)	4 (3 to 7)	1230 (874 to 1655)	1207 (858 to 1622)	23 (14 to 34)	525 (322.9 to 784.6)	527.6 (322 to 798.2)	413.2 (242 to 649)

Data in parentheses are 95% uncertainty intervals; DALYs = Disability-adjusted life years; YLLs = Years of life lost; YLDs = Years lived with disability

Regarding SDI quintiles at the subnational level, ASIR increased with SDI in both 1990 and 2019. The mean ASIR for high and high-middle SDI was approximately doubled from 1990 to 2019. Low, low-middle, and middle SDI quintile regions also experienced this remarkable increase, while mean ASIRs in these regions were overall lower than that of high-middle and high-SDI regions in 1990 and 2019 (Fig. 3B).

Decomposition analysis revealed that 133.0% and 44.0% of the total 417.6% change in incident cases from 1990 to 2019 were attributable to age structure change and population growth, respectively. Thus, it was estimated that 240.7% of the total increase in new cases was related to the true increase in the BC-specific incidence over this period. All the decomposition analyses for both females and males at the national and subnational levels are presented in Table 2.

### The breast cancer burden of deaths and DALYs in Iran

BC led to 4704 (4306–5192) deaths among females in 2019, almost 3.3-fold more than 1990 with 1413 (1160–1796) deaths at the national level. ASDR for females also increased slightly over these 30 years from 10.3 (8.2–13.6)/100,000 to 11.9 (10.8–13.1)/100,000, which had a PC of 14.9% (− 15.1–47.7) and 0.50 (0.34–0.66) APC. Moreover, the age-standardized DALYs rate of females was 368.7 (336.7–404.3)/100,000 in 2019, 15.2% (− 11.7–42.5) higher than of 1990 (320.2 [265.4–405.4]/100,000), 0.51 (0.36–0.65) APC; however, the overall PCs in age-standardized DALYs and ASDR in males decreased over this period (-10.7% [− 36.1–20.7] and − 11.4% [− 37.0–22.6], respectively). It is worth mentioning that 93.8% of the overall age-standardized DALYs rate in both sexes was related to YLLs in 2019, while this proportion was 96.0% in 1990 (Table 1).

At the subnational level, ASDR and age-standardized DALYs rate increased remarkably in most provinces of Iran; these increasing rates were mostly estimated for central and northern parts of Iran, as illustrated in Figs. 2A, B, and C. Females had positive ASDR PCs in most provinces, while males mostly had negative ASDR PCs in Iran (Fig. 3A). Changes in age-standardized DALYs rate also revealed almost a similar pattern to ASDR in most provinces for both sexes (Fig. 3A). Based on Fig. 3A, two provinces (Ilam and Zanjan) had the most remarkable deviation from the others and the overall PCs in age-standardized DALYs and ASDRs in the country and had the highest positive PCs in the first period; however, they had PCs lower than average in the second period. Ilam had the highest APCs in age-standardized DALYs and ASDR between 1990 and 2019, 1.75 (1.63–1.87) and 1.80 (1.63–1.96), respectively (Additional file 3: Table 2).

**Fig. 4:**
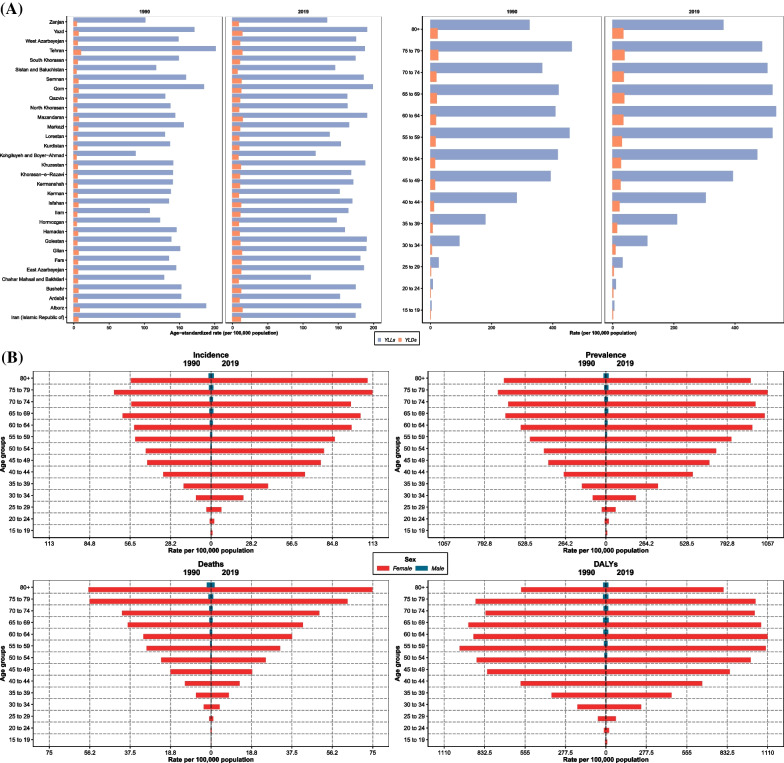
**A** Breast cancer age-standardized years of life lost (YLLs) and years lived with disability (YLDs) rates (per 100,000 population), in 1990 and 2019 for both sexes. (left: for Iran and its 31 provinces; right: based on age groups). **B** Breast cancer incidence, prevalence, deaths, and disability-adjusted life years (DALYs) rates (per 100,000 population) in 1990 and 2019 based on age groups by sex (red: females; blue: males) in Iran

All the provinces’ rankings in terms of ASDR for both sexes and females and males separately are presented in Additional file 4: Fig. 2A, Additional file 5: Fig. 2B, and Additional file 6: Fig. 2C. Among females, the highest estimated ASDR in 1990 was 14.3 (9.9–20.8)/100,000 (Tehran). The first-ranked province in 2019 had, similarly, an ASDR of 14.3 (11.6–17.2)/100,000 (Qom); however, the lowest ASDR for females was estimated at 7.5 (5.7–9.7)/100,000 (Chahar Mahaal and Bakhtiari) in 2019 compared to 6.4 (4–10.2)/100,000 (Kohgiluyeh and Boyer-Ahmad) in 1990 (Additional file 5: Fig. 2B). Males had ASDRs of approximately 0.5/100,000 or lower in 1990 and 2019 (Additional file 6: Fig. 2C). Considering YLLs and YLDs, both rates increased remarkably in Iran and almost all provinces; however, only limited provinces experienced decreasing YLLs while having increasing YLDs from 1990 to 2019, such as Tehran and Chahar Mahaal and Bakhtiari (Fig. 4A).

Based on SDI quintiles, the ASDR and the age-standardized DALYs rate in all quintiles were higher in 2019 compared to 1990. Overall, high and high-middle SDI quintiles had slightly higher mean ASDRs and age-standardized DALYs rate than the others (Fig. 3B).

### Age-related breast cancer burden

Based on age groups, both sexes’ incidence and deaths rates were higher in almost all age groups in 2019 than in 1990 at the national level. Overall, the incidence rate increased by age in both 1990 and 2019, and the highest incidence rate was estimated in the 75–79 age group in both 1990 and 2019. Females were diagnosed with BC at younger age groups than males who were diagnosed with BC, mostly over 40–45 years. Females over 80 also experienced the highest increase in the incidence rate over this period (Fig. 4B). Although ASDR among males did not change remarkably in different age groups, it increased considerably in females of all age groups over this period (Fig. 4B). DALYs rate also increased among Iranian females of all ages from 1990 to 2019, while it had a reverse U-pattern association with age, reaching a peak at around 1,144/100,000 in the 55–59 age group in 2019. The highest age-standardized YLDs rate was estimated for the 75–79 age group, followed by 65–69 years (Fig. 4A). Of note, men had a negative change regarding DALYs rate in most age groups from 1990 to 2019. We found that the + 70-age group had highest ASIR and ASDR compared to < 50 (15–49) and 50–69 age groups, while the 50–69 age group had the highest age-standardized DALYs rate in both 1990 and 2019. It is worth noting that the most remarkable increases in the age-standardized rates were found < 50 years (15–49 age group) compared to age groups 50–69 and + 70 years since the 15–49 age group had highest PCs between 1990 and 2019 despite the relatively lower rates in this age group (Table 3).

At the subnational level, almost all 31 provinces had a similar increasing pattern to the national trend of Iran for ASIR and ASDR by age groups, with the highest rates estimated for age groups over 70 years. Besides, the highest ASDR was estimated for the + 80-age group in all provinces. In almost all provinces, DALYs rate followed a similar pattern to Iran, with the highest rates estimated for middle-aged groups (Additional file 9: Fig. 3, Additional file 10: Fig. 4, Additional file 11: Fig. 5 and Additional file 12: Fig. 6).

### Correlation between measures of burden and SDI

We found a high positive correlation between age-standardized rates of all burden measures and SDI in females (Additional file 13: Table 5), and the highest Pearson’s correlation coefficients (*r*) were calculated for incidence, prevalence, and YLDs (*r* > 0.80). On the contrary, males had low correlations between the measures and SDI, and the lowest negative correlation in males was found for death rates (*r* = − 0.05).

### Correlation between females and males in different provinces regarding BC burden

**Fig. 5:**
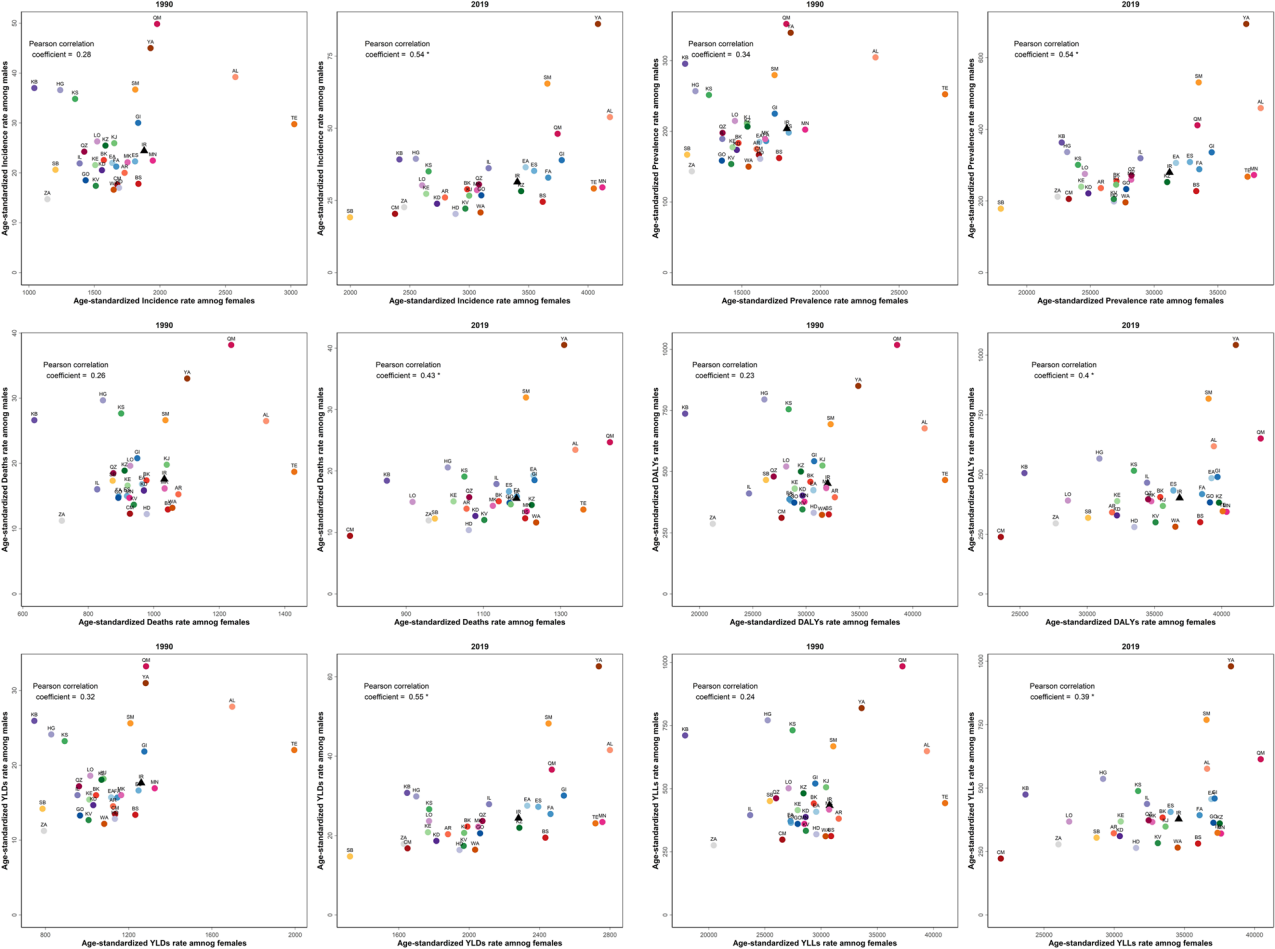
Pearson’s correlation coefficient (*r*) between males and females of all provinces of Iran regarding measures of BC burden

We found positive correlations between females and males with reference to ASIR, ASDR, and age-standardized DALYs rate in different provinces in the year 2019 with *r* of 0.54, 0.43, and 0.40 respectively. However, there were no meaningful positive correlations between females and males for the age-standardized rates in 1990 (Fig. 5).

### Mortality-to-incidence ratio (MIR)

**Fig. 6:**
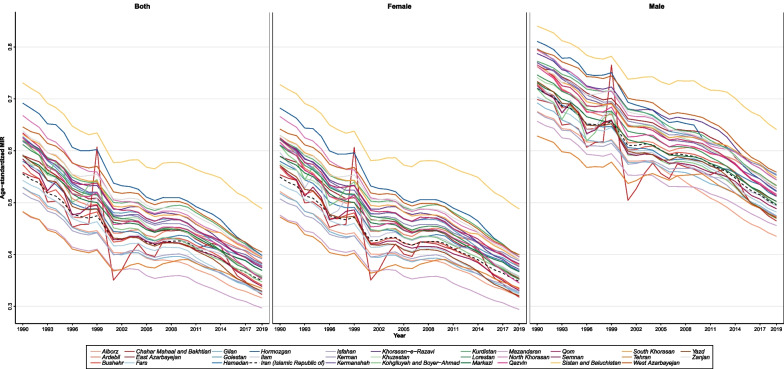
Age-standardized mortality-to-incidence ratio (MIR) trend in Iran and all provinces from 1990 to 2019

Overall, age-standardized MIR for BC revealed a downward trend in Iran and all provinces for both females and males (Fig. 6). Generally, males had a higher MIR than females in Iran, but they followed almost a similar pattern in all provinces. The overall age-standardized MIR for BC in Iran was 0.35 in 2019 compared to 0.55 in 1990. At the subnational level, the highest and the lowest MIR in 2019 were estimated as 0.48 and 0.30, respectively (Sistan and Baluchestan and Mazandaran). In 1990, the highest and the lowest MIR were also recorded in the same provinces (0.73 and 0.48, respectively).

### The breast cancer burden attributable to risk factors

**Fig. 7:**
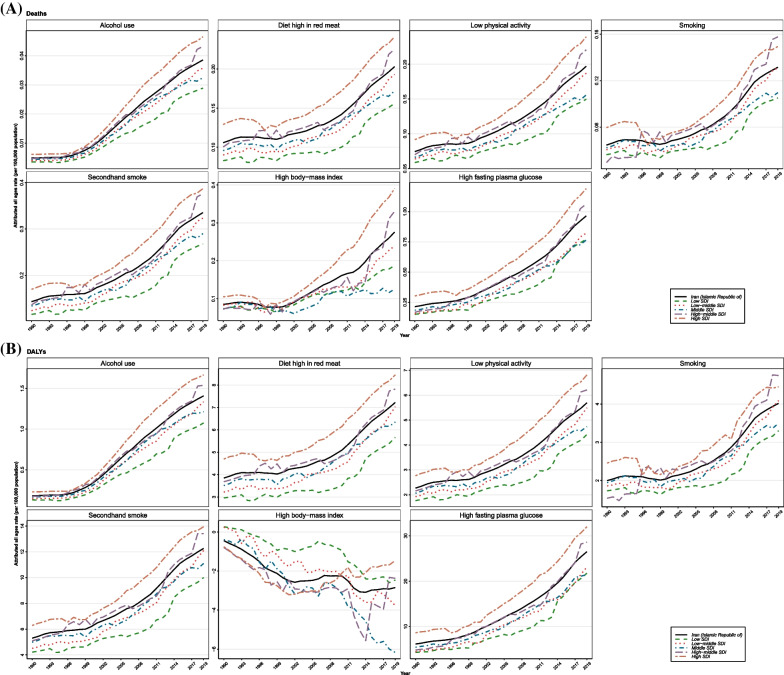
Time trend of all-ages breast cancer **A** deaths and **B** disability-adjusted life years (DALYs) rates (per 100,000) attributable to risk factors are shown for Iran and its subnational socio-demographic index (SDI) quintiles from 1990 to 2019 in females

Due to the scarce data around risk factors attributed burden in males, we have only provided the attributable burden to risk factors in females. Overall, deaths and DALYs rate attributable to each risk factor had upward trends in Iran and all SDI quintiles from 1990 to 2019; high and high-middle SDI quintiles generally had the highest deaths and DALYs rate attributable to risk factors, except for high BMI (Fig. 7A and B). High FPG exerted the highest burden among risk factors, followed by secondhand smoke, low physical activity, diet high in red meat, smoking, and alcohol use (Table 4). High FPG-related ASDR for females was 1.1 (0.2–2.4)/100,000 in 2019 with 117.1% (52.0–210.1) increment compared to 1990, and its age-standardized DALYs rate was 28.1 (5.5–61.0)/100,000 in 2019 (PC of 117.1% [60.6–194.2] from 1990 to 2019). Alcohol use, on the other hand, had the least attributable burden, with the age-standardized DALYs rate of 1.3 (0.9–1.8)/100,000 among Iranian females, followed by high-BMI with the attributed age-standardized DALYs rate of 3.2 (-11.3–16.5)/100,000 (Table 4). BC burden attributable to each risk factor at the subnational level can be found in Additional file 14: Table 6.

**Table 4:** Breast cancer deaths and disability-adjusted life years (DALYs) by rates (per 100,000 population) and numbers with percent changes attributable to risk factors in Iran between 1990 and 2019 for females and both sexes

Measure	Metric	Risk factors	1990		2019		% Change (1990 to 2019)	
			Both	Female	Both	Female	Both	Female
Deaths	Rate^*^	Alcohol use	0.01 (0 to 0.01)	0.01 (0.01 to 0.02)	0.02 (0.01 to 0.03)	0.04 (0.03 to 0.05)	305.81 (154.71 to 589.77)	307.69 (151.07 to 603.52)
		Diet high in red meat	0.11 (0.02 to 0.17)	0.21 (0.05 to 0.34)	0.1 (0.02 to 0.15)	0.21 (0.05 to 0.29)	-0.92 (-30.42 to 30.96)	-2.81 (-31.93 to 29.07)
		Low physical activity	0.09 (0.03 to 0.17)	0.18 (0.06 to 0.35)	0.11 (0.04 to 0.2)	0.23 (0.09 to 0.4)	25.25 (-15.01 to 71.1)	25.48 (-14.44 to 71.22)
		Smoking	0.06 (0.03 to 0.11)	0.13 (0.07 to 0.22)	0.07 (0.05 to 0.1)	0.14 (0.09 to 0.2)	12.52 (-26.93 to 79.53)	5.77 (-31.18 to 70.05)
		Secondhand smoke	0.14 (0.03 to 0.26)	0.29 (0.07 to 0.53)	0.17 (0.04 to 0.29)	0.34 (0.08 to 0.59)	20.12 (-11.6 to 55.72)	17.79 (-13.82 to 52.6)
		High body-mass index	0.13 (-0.02 to 0.34)	0.28 (-0.04 to 0.72)	0.24 (-0.01 to 0.53)	0.47 (-0.03 to 1.05)	83.75 (-105.9 to 406.19)	69.14 (-72.49 to 323.7)
		High fasting plasma glucose	0.25 (0.04 to 0.66)	0.51 (0.09 to 1.33)	0.56 (0.11 to 1.22)	1.11 (0.22 to 2.44)	119.99 (53.4 to 214.37)	117.1 (51.97 to 210.06)
	Number^†^	Alcohol use	2 (1 to 2)	1 (1 to 2)	17 (12 to 23)	16 (11 to 22)	1005.23 (616.95 to 1763.54)	1046.3 (625.58 to 1834.68)
		Diet high in red meat	31 (7 to 48)	30 (7 to 47)	85 (19 to 120)	84 (19 to 119)	176.96 (98.44 to 256.87)	178.05 (98.49 to 261.79)
		Low physical activity	21 (8 to 41)	21 (8 to 41)	82 (32 to 148)	82 (32 to 148)	283.56 (172.64 to 409.08)	283.56 (172.64 to 409.08)
		Smoking	18 (10 to 30)	18 (10 to 30)	55 (35 to 76)	55 (35 to 76)	195.42 (92.8 to 366.65)	195.42 (92.8 to 366.65)
		Secondhand smoke	42 (10 to 74)	41 (10 to 73)	140 (33 to 244)	139 (33 to 243)	237.69 (154.71 to 327.92)	238.75 (155.15 to 331.08)
		High body-mass index	24 (-18 to 76)	24 (-18 to 76)	114 (-75 to 314)	114 (-75 to 314)	370.42 (-807.73 to 1678.25)	370.42 (-807.73 to 1678.25)
		High fasting plasma glucose	61 (10 to 153)	61 (10 to 153)	400 (80 to 875)	400 (80 to 875)	555.92 (379.83 to 808.21)	555.92 (379.83 to 808.21)
DALYs	Rate	Alcohol use	0.17 (0.1 to 0.26)	0.32 (0.19 to 0.5)	0.68 (0.48 to 0.95)	1.3 (0.91 to 1.83)	301.94 (163.97 to 568.12)	302.43 (156.36 to 583.19)
		Diet high in red meat	3.4 (0.79 to 5.15)	6.94 (1.62 to 10.57)	3.37 (0.76 to 4.76)	6.71 (1.51 to 9.46)	-0.65 (-28.56 to 26.8)	-3.25 (-30.78 to 24.15)
		Low physical activity	2.24 (0.85 to 4.32)	4.62 (1.74 to 8.83)	2.9 (1.14 to 5.35)	5.81 (2.29 to 10.72)	29.32 (-5.96 to 68.02)	25.78 (-8.46 to 63.07)
		Smoking	1.85 (1 to 2.96)	3.91 (2.1 to 6.3)	2.07 (1.31 to 2.9)	4.11 (2.59 to 5.77)	12.01 (-26.04 to 75.52)	5.14 (-30.62 to 65.2)
		Secondhand smoke	4.64 (1.13 to 8.25)	9.52 (2.31 to 16.95)	5.65 (1.33 to 9.83)	11.3 (2.65 to 19.69)	21.85 (-7.06 to 52.61)	18.71 (-9.69 to 48.58)
		High body-mass index	0.85 (-3.63 to 5.6)	2.32 (-7.07 to 12.34)	1.83 (-5.31 to 8.55)	3.19 (-11.27 to 16.54)	115.85 (-853.14 to 1069.54)	37.6 (-625.36 to 938.45)
		High fasting plasma glucose	6.23 (1.07 to 15.5)	12.93 (2.22 to 32.15)	14.08 (2.78 to 30.59)	28.06 (5.54 to 61)	126.01 (67.03 to 207.34)	117.06 (60.57 to 194.24)
	Number	Alcohol use	57 (34 to 87)	52 (31 to 81)	613 (432 to 858)	584 (409 to 818)	977.72 (611.8 to 1674.21)	1015.69 (625.97 to 1768.42)
		Diet high in red meat	1116 (267 to 1663)	1101 (263 to 1645)	3023 (688 to 4257)	2995 (682 to 4224)	171.01 (96.11 to 244.28)	172.06 (96.43 to 246.35)
		Low physical activity	649 (249 to 1259)	649 (249 to 1259)	2362 (955 to 4434)	2362 (955 to 4434)	264.04 (169.57 to 362.85)	264.04 (169.57 to 362.85)
		Smoking	572 (307 to 922)	572 (307 to 922)	1667 (1031 to 2371)	1667 (1031 to 2371)	191.52 (92.31 to 353.93)	191.52 (92.31 to 353.93)
		Secondhand smoke	1530 (379 to 2698)	1517 (374 to 2679)	5115 (1197 to 8915)	5087 (1190 to 8868)	234.42 (158.11 to 313.87)	235.38 (158.12 to 314.99)
		High body-mass index	-127 (-1609 to 1316)	-127 (-1609 to 1316)	-1181 (-7517 to 4078)	-1181 (-7517 to 4078)	828.25 (-2861.43 to 2850.23)	828.25 (-2861.43 to 2850.23)
		High fasting plasma glucose	1766 (307 to 4321)	1766 (307 to 4321)	10,994 (2155 to 24,037)	10,994 (2155 to 24,037)	522.62 (368.61 to 725.7)	522.62 (368.61 to 725.7)

Data in parentheses are 95% uncertainty intervals; DALYs = Disability-Adjusted Life Years

*Age-standardized rate (per 100,000)

**†**All ages

A total DALYs (all-ages numbers) of 5563 were attributable to all the risk factors mentioned above in 1990, with a fraction of about 11.0% of the total DALYs. The fraction of attributable burden to all the included risk factors was 13.8% in 2019 (22,593/163,091); see Tables 1 and 4.

## Discussion

This study revealed that BC incidence, deaths, and DALYs generally had upward trends in Iran from 1990 to 2019. Although ASIR for males was increasing almost constantly over the study period, their ASDR and age-standardized DALYs had negative PCs from 1990 to 2019 in most age groups. In contrast, these trends increased for females of all ages during this period. Decomposition analyses revealed that 133.0% and 44.0% of the total 417.6% change in incident cases from 1990 to 2019 were attributable to age structure change and population growth, respectively, and 240.7% of the total increase in new cases was related to the true increase in the BC-specific incidence over this period. Besides, females faced BC at a younger age than males; however, the incidence of BC increased remarkably with aging in both sexes. Age-standardized MIRs had a downward trend from 1990 to 2019 in Iran and all provinces, while men had higher MIRs than females over the study period. Regarding associated risk factors, high FPG was found to exert the most attributed burden in Iran, contrary to alcohol as the least.

Concerning age and sexes, our study found that the incidence rate in females increased by age, and the highest incidence rate was estimated for the 75–79 age group. Despite the less significant estimates on the burden of BC in younger age groups, previous studies estimated that BC still affects 4–6% of females under 40 years [32]. We also found that the chance of BC accelerates with aging. Also, we found that the younger population under 50 years experienced the sharpest increase in the incidence rate of BC compared to older age groups, specifically women under 50 years had the highest magnitude in ASIR increase over the study period. This finding was in line with previous studies on female and male BC at the global level. Z Chen et al. found in a recent study that the magnitude of the increase in ASIR decrease by age, and females under 50 years had the highest increase in their rates despite the relatively lower ASIR among the population [33]. So, special attention should be paid to younger age groups focusing on improving general awareness, developing screening programs and sticking to them, and modulating preventable risk factors, which might help control the increasing burden of BC among younger population as well as advanced ages. Concerning the remarkable increase in BC incidence among younger age groups, special attention should also be paid to other possible risk factors in the nation, such as stress, socioeconomic situation, and lifestyle and environmental changes. It is worth investigating by future studies to explore the possible effect of such factors on the increasing burden of BC among younger age groups more in depth. Besides, male BC has been mostly underrepresented in studies and clinical trials due to the meager incidence rate of BC among men. Limited studies on male BC revealed that men were diagnosed with BC mostly aged > 40 years, and the median age at diagnosis in males was approximately five years higher than females, 67 and 62 years, respectively, and presented at more advanced stages with a lower survival rate [8, 34, 35], concordant with our results showing that BC was mostly diagnosed in men over 40–44 years. Our study showed that although the estimated percentage of new male BC cases out of all diagnosed cases decreased from 1.2% in 1990 to 0.8% in 2019, ASIR still increased in males over this period. This study also found that MIR was higher in males than in females. This might be due to the evidence that males are mostly diagnosed at more advanced ages and stages than females. Previous studies also reported a lower survival rate in males than females, which could explain the higher MIRs in males based on our findings [35]. So, males should not be neglected, and effective strategies should be tailored to diagnose male BC at earlier ages and stages and manage them appropriately. Further studies are needed focusing on the early detection measures and treatment modalities to tackle high MIRs in males. Identifying males at higher risk for BC and developing screening programs for such individuals might be beneficial based on previous studies’ evidence [36]. MIR is associated with the clinical outcome of cancers and could be utilized as an indicator of the quality of care provided by the healthcare system [37, 38]. Based on previous studies, the global MIR for female BC decreased from 0.4 in 1990 to 0.3 in 2016 [25]. In Iran, we also found a similar downward pattern for the MIR of BC for both females and males. We inferred that these decreasing trends might be due to improvements in early diagnosis and treatment capacities over these 30 years, leading to a better survival rate.

Regarding SDI quintiles, high and high-middle SDI regions in Iran were found to have greater incidence, deaths, and DALYs rate than the others. In contrast, previous studies revealed that ASDR and DALYs rate of BC decreased globally and in high and high-middle SDI regions of the world over time, and the low-middle SDI regions surpassed the high SDI over the 2010–2017 period [39]. Improvements in therapeutic approaches, including up-to-date surgical technologies, radiation therapies, and the use of novel systemic agents based on BC subtypes [40] in developed countries, might have led to the decreased ASDR and DALYs rate in such regions. Iran is still a middle-income developing country facing limitations in diagnostic and screening capacities and access to novel therapeutic modalities. Still, better diagnostic capacities, access to healthcare facilities, and higher levels of awareness in high-SDI regions of Iran seem to be responsible for higher estimates of BC burden in these regions than in lower SDIs. Besides, lifestyle-associated and environmental and occupational risk factors might be more significant in high-SDI regions due to modernization, such as diet high in red meat and low in vegetables and organic products, and toxic environmental pollutants. Studies proposed that industrial development has led to the soil, surface water, and food, as a consequence, pollution by heavy metal salts, which have been found to stimulate BC progression and reduce its sensitivity to treatment [41]. Results of the current study also found that the impact of PCs in ASIR and ASDR is declining over time (from 2010–2019 compared to the 1990–2010 period) in wealthier provinces of Iran, while PCs in more deprived provinces in recent years were still as high as previous decades. These findings were in line with the results of a recent investigation by Rahimzadeh et al [42], on the impact of geographical and socioeconomic inequalities in female BC incidence and mortality in Iran, suggesting that a possible reversal might occur in the upcoming years, with the most deprived provinces possessing the highest ASDRs in the country. So, more attention should be paid to low SDI regions in Iran to resolve possible inequalities and control the impact of increasing mortality rates and the burden of BC in these regions. Iran is a vast country, longitudinally and latitudinally, consisting of a wide range of ethnicities. In addition to the possible factors mentioned above, disparities in lifestyles, individuals’ behaviors, cultures, genetic backgrounds, the age structure of the population in each region, and geographical differences might correspond to the divergence in rates in different country regions [43–46]. It necessitates future studies aiming at close observations and evaluations to find out possible underlying causes of disparities and any possible inequalities in access to healthcare services and the quality of care.

According to the GBD risk factor hierarchy related to BC, we found that high-FPG exerted the most attributed deaths and DALYs among Iranian females, and its attributed burden had an upward trend over the study period. Globally, alcohol consumption seems to be the most important contributing factor to the BC burden [12, 47]. However, we found that alcohol use was a minor attributable factor among the Iranian population. It is worth noting that alcohol consumption is illegal in Iran, and the data regarding this risk factor might not be accurate. Previous investigations also reported that in countries with religious-based policies to keep the rate of alcohol consumption low, the lowest PAFs were recorded regarding this factor [48].

Regarding tobacco exposure, some studies found that secondhand (passive) smoking puts individuals at a higher risk of BC than active smoking [49, 50]. Accordingly, in the present study, we found that secondhand smoking was associated with deaths and DALYs rate more than active smoking. Besides, the association between BC and BMI is controversial and still under investigation [51]. Some studies showed a strong association between increased BMI and higher BC incidence, especially in postmenopausal women who are not taking exogenous hormones, and obesity has been found as a potential risk factor for BC. By contrast, in premenopausal women, high BMI is negatively associated with BC risk [52]. In the present study, we found that high-BMI-related deaths rate had an increasing trend over the study period, while a downward trend was observed regarding DALYs. This trend could be explained by the diverse role of obesity in BC among different age groups.

Nevertheless, lifestyle-associated risk factors should be regulated in the population through preventing obesity and not having a high-fat diet. Studies also revealed that daily and routine exercise and vegetable consumption could lower the risk of BC development [50]. In a recent meta-analysis concerning the association between nutrition and the risk of BC, the authors found that fruit, vegetables, and soy consumption had an inverse association with BC. However, red meat and processed meat both had a positive association with BC risk, while processed meat consumption showed a higher impact than red meat [53]. Therefore, adopting a healthy lifestyle, including a healthy diet, physical activity, and non-smoking behavior, might help prevent BC in the community setting [54, 55].

Estimates from the GBD study provide a good vision of the burden of diseases worldwide for clinicians and policymakers. Accordingly, the present study results could raise the health information, which can be helpful in adapting appropriate strategies to control the increasing burden of BC in Iran and similar developing countries worldwide. Previous studies suggested that developing a National Cancer Control Program (NCCP) might immensely improve the management of cancers in each country by mapping the existing problems and considering possible implementation solutions, especially in developing and low-income countries, which have been reported to have no effective NCCP [56]. Besides, recent studies in Iran revealed that around 61% of Iranian women know about BC and its screening programs, while only 17% stick to these programs [57]. So, mass education of the population, especially for individuals at a higher risk of BC, based on the geographical, genetic factors, and findings from burden studies could raise general awareness, leading to better prevention and early detection of cancers.

### Limitations

Despite providing high-quality estimates of the burden associated with a wide range of diseases and injuries by the GBD study, some limitations might still exist [17]. The GBD study primarily relies on data acquired from vital and cancer registries and other epidemiologic studies worldwide, and the results mainly depend on the out-of-sample predictive validity of modeling efforts [17]. In many regions of the world, especially in low-income regions, cancer registries might not be fully developed and thus reliable enough, which could influence the final results [58, 59]. Early detection of new cases requires updated systems and advanced modalities that might not be effectively accessible in low-income regions and provinces. Based on previous reports, cancer registries began in Iran in 1999, while it did not cover all cancer data from pathology laboratories and departments all over the country during the first years, and it took years to be developed to a higher standard level [60]. So, data from that period in the GBD study mostly rely on epidemiological studies and systematic reviews. The GBD study adopted advanced estimation models and methods to tackle the scarcity of data in such regions as much as possible, as described briefly in the methods of the present study. Recent investigations by the NASBOD project also attempted to estimate and resolve possible incompleteness of cancer registry systems in Iran by adapting data from the Social Security Organization Cancer Registry (SSOCR), since before receiving cancer medications, all cancer patients should be registered in the SSOCR database [16]. Results from the NASBOD project estimating the burden of cancers could be accessed using online data visualization tools at www.vizit.report website.

There were also limitations regarding males, especially concerning the burden attributable to risk factors. There were very few BC cases among males in Iran over the study period. So, the interpretations of results for males in this study should be made and applied cautiously. We were also not able to investigate the burden of BC attributable to risk factors in males due to the limited number of cases in each category. So, we could report attributable burden to risk factors only for females. It is highly recommended to investigate the male BC burden and the potential risk factors in future studies more precisely.

Concerning existing disparities among different subnational regions of Iran, it is also vital to investigate access to routine screening programs and explore some key points, such as which regions have or do not have access to screening programs, to what extent the national screening programs for BC are performed in each region, and what percentage of the population among younger and older age groups are screened. Future studies could provide valuable information and help better policy making in the nation regarding BC burden control by answering such questions require, which requires precise investigations at the national and subnational level.

Some limitations also arise from the Iranian population, culture, and genetic background. For example, it is worth noting that alcohol consumption is illegal in Iran based on culture and religion. So, the reported data might be underestimated regarding alcohol consumption. This might influence the real burden attributable to alcohol use among the Iranian population. Possible genetic diversities among different ethnicities might influence the existing disparities regarding BC burden in different provinces, which could not be covered and investigated based on the GBD study results. Further studies are needed to focus on this aspect and investigate the role of genetic diversity in the future. We could investigate the BC burden attributable to risk factors based on the GBD study 2019 risk factors hierarchy. In this study, we found that only 11.0% of the total DALYs in 1990 and 13.8% in 2019 were attributable to the included risk factors, and about 86.2% of the total DALYs in 2019 were associated with other factors. There are still some potential risk factors known to be associated with BC, such as genetic background and gene mutations, hormonal patterns, and hazardous environmental pollutants, to name but a few. The GBD study constantly investigates the burden attributable to more risk factors in each updated iteration. So, we suggest that future iterations of the GBD study incorporate more known risk factors of BC to investigate their attributed burden.

Besides, the quality of care provided by healthcare systems is another vital public health concern. It is suggested that future studies investigate the quality of care provided in different regions of Iran to BC patients. The novel quality of care index (QCI) has been recently introduced in the literature, which seems to be a reliable tool for the quality-of-care assessment for a specific cause in each sex-age group-location-year [61–63].

Although economic sanctions against Iran in recent decades did not directly target healthcare systems and access to medication, it seems that they led to severe issues for the healthcare system and patients with cancer, exerting a severe economic burden on the whole system and limiting access to quality care. Future studies might investigate the possible burden associated with sanctions as a public health concern in Iran in recent years more in-depth.

## Conclusions

In this study, we reported for the first time the most up-to-date estimates regarding BC burden in Iran for females and males at the national and subnational levels, based on age and SDI over thirty years from 1990 to 2019. We also performed decomposition analysis to find the attribution of population growth, population aging, and BC incidence rate in the total change of BC incident cases between 1990 and 2019 and reported MIRs—as an indicator of the quality of care—to provide a more comprehensive viewpoint on the current status of BC burden in Iran for the policymakers and health authorities. Besides, we assessed and reported the burden of BC attributable to known risk factors based on the GBD 2019 risk factor hierarchy. This study also will provide insights for other similar regions of the world to Iran to adopt the methodology of this study, which will help them reach comparable results and a broad vision concerning the burden of this lethal and prevalent cancer.

Still, more attention must be paid to Iran, at national and subnational levels, especially in low SDI regions. The policy-making should focus on systematic screening programs, especially for high-risk individuals, mass education about BC risk factors, self-examination, screening measures, healthy diet, lifestyle modifications, improving diagnostic and early detection tools, increasing access to the healthcare system and diagnostic facilities, developing comprehensive cancer registries, and cost-effective therapies to control the increasing trends. Developing an efficient NCCP could be a crucial step toward controlling the BC burden in Iran and other developing regions of the world. No doubt, further studies are needed to investigate the reasons behind the existing disparities in BC burden in different parts of Iran, explore more risk factors that might affect this burden, investigate the role of sex, hormones, genetic, and ethnic factors, assess the burden of BC in males more accurately, and investigate possible effective strategies to tackle the increasing burden of BC in the future more in-depth.

## Supplementary Information

**Additional file 1**. Fig. 1 The geographical location of Iran in the globe (shown in red color) and its subnational administrative divisions.

**Additional file 2**. Table 1 Scientific papers and data registry systems that were used by the GBD study 2019 to model and estimate the burden of breast cancer in Iran at national and subnational levels.

**Additional file 3**. Table 2 Annual percent changes of breast cancer age-standardized incidence, prevalence, deaths, disability-adjusted life years (DALYs), years of life lost (YLLs) and years lived with disability (YLDs) rates from 1990 to 2019 at the national and subnational levels.

**Additional file 4**. Fig. 2A Breast cancer age-standardized incidence and deaths rates (per 100,000 population) rankings in Iran and its 31 provinces in 1990 and 2019, for A) both sexes, B) females, and C) males.

**Additional file 5**. Fig. 2B Breast cancer age-standardized incidence and deaths rates (per 100,000 population) rankings in Iran and its 31 provinces in 1990 and 2019, for A) both sexes, B) females, and C) males.

**Additional file 6**. Fig. 2C Breast cancer age-standardized incidence and deaths rates (per 100,000 population) rankings in Iran and its 31 provinces in 1990 and 2019, for A) both sexes, B) females, and C) males.

**Additional file 7**. Table 3 Breast cancer age-standardized incidence, prevalence, deaths, disability-adjusted life years (DALYs), years of life lost (YLLs) and years lived with disability (YLDs) rates (per 100,000 population), numbers and percent changes in all 31 provinces of Iran between 1990 and 2019, for both sexes, females, and males (provinces have been sorted alphabetically).

**Additional file 8**. Table 4 Percent changes (%) in breast cancer age-standardized rates and all-ages numbers of incidence, prevalence, deaths, disability-adjusted life years (DALYs), years of life lost (YLLs), and years lived with disability (YLDs) over two major time periods, between 1990 and 2010 and between 2010 and 2019, in Iran and all 31 provinces for both sexes, females, and males.

**Additional file 9**. Fig. 3 Breast cancer incidence rates (per 100,000 population) in 1990 and 2019 based on age groups by sex (red: female; blue: males) in Iran and its 31 provinces.

**Additional file 10**. Fig. 4 Breast cancer prevalence rates (per 100,000 population) in 1990 and 2019 based on age groups by sex (red: female; blue: males) in Iran and its 31 provinces.

**Additional file 11**. Fig. 5 Breast cancer deaths rates (per 100,000 population) in 1990 and 2019 based on age groups by sex (red: female; blue: males) in Iran and its 31 provinces.

**Additional file 12**. Fig. 6 Breast cancer disability-adjusted life years (DALYs) rates (per 100,000 population) in 1990 and 2019 based on age groups by sex (red: female; blue: males) in Iran and its 31 provinces.

**Additional file 13**. Table 5 Pearson’s correlation coefficient (r) between measures of burden and socio-demographic index (SDI)

**Additional file 14**. Table 6 Breast cancer deaths and disability-adjusted life years (DALYs) rate (per 100,000 population), numbers and percent changes attributable to risk factors in all 31 provinces of Iran between 1990 and 2019 for both sexes, females, and males (provinces have been sorted alphabetically).

**Additional file 15**. Contributions by the GBD Iran Breast Cancer Collaborators.

## Data Availability

Data regarding the burden of BC for Iran, at the national and subnational levels, were obtained from the Global Health Data Exchange (GHDx) query online tool, over 30 years from 1990 to 2019, which is publicly available at http://ghdx.healthdata.org/gbd-results-tool.
