# Correction to: National and subnational burden of female and male breast cancer and risk factors in Iran from 1990 to 2019: results from the Global Burden of Disease study 2019

**DOI:** 10.1186/s13058-023-01666-9

**Published:** 2023-06-15

**Authors:** Armin Aryannejad, Sahar Saeedi Moghaddam, Baharnaz Mashinchi, Mohammadreza Tabary, Negar Rezaei, Sarvenaz Shahin, Nazila Rezaei, Mohsen Naghavi, Bagher Larijani, Farshad Farzadfar, GBD 2019 Iran Breast Cancer Collaborators, Mohsen Abbasi-Kangevari, Zeinab Abbasi-Kangevari, Hedayat Abbastabar, Hassan Abidi, Hassan Abolhassani, Mohammad Aghaali, Bahman Ahadinezhad, Ali Ahmadi, Sepideh Ahmadi, Marjan Ajami, Mohammad Esmaeil Akbari, Yousef Alimohamadi, Sadaf Alipour, Vahid Alipour, Saeed Amini, Ali Arash Anoushirvani, Jalal Arabloo, Morteza Arab-Zozani, Bahar Ataeinia, Seyyed Shamsadin Athari, Abbas Azadmehr, Sina Azadnajafabad, Mohammadreza Azangou-Khyavy, Amirhossein AzariJafari, Nader Bagheri, Sara Bagherieh, Saeed Bahadory, Sima Besharat, Somayeh Bohlouli, Natália Cruz-Martins, Mostafa Dianatinasab, Mojtaba Didehdar, Shirin Djalalinia, Fariba Dorostkar, Sharareh Eskandarieh, Bita Eslami, Shahab Falahi, Mohammad Farahmand, Ali Fatehizadeh, Masood Fereidoonnezhad, Nasrin Galehdar, Seyyed-Hadi Ghamari, Ahmad Ghashghaee, Maryam Gholamalizadeh, Ali Gholami, Pouya Goleij, Mohamad Golitaleb, Nima Hafezi-Nejad, Arvin Haj-Mirzaian, Aram Halimi, Soheil Hassanipour, Mohammad Heidari, Zahra Heidarymeybodi, Keyvan Heydari, Mohammad-Salar Hosseini, Elham Jamshidi, Roksana Janghorban, Ali Kabir, Leila R. Kalankesh, Taras Kavetskyy, Leila Keikavoosi-Arani, Mohammad Keykhaei, Rovshan Khalilov, Javad Khanali, Mahmoud Khodadost, Ali-Asghar Kolahi, Farzad Kompani, Hamid Reza Koohestani, Mozhgan Letafat-nezhad, Somayeh Livani, Amirhosein Maali, Farzan Madadizadeh, Soleiman Mahjoub, Ata Mahmoodpoor, Mohammad-Reza Malekpour, Reza Malekzadeh, Mohammad Ali Mansournia, Sahar Masoudi, Seyedeh Zahra Masoumi, Entezar MehrabiNasab, Seyyedmohammadsadeq Mirmoeeni, Esmaeil Mohammadi, Abdollah Mohammadian-Hafshejani, Mohammad Mohseni, Sara Momtazmanesh, Abdolvahab Moradi, Maryam Moradi, Yousef Moradi, Farhad Moradpour, Rahmatollah Moradzadeh, Abbas Mosapour, Mozhgan Moshtagh, Haleh MousaviIsfahani, Christopher J. L. Murray, Javad Nazari, Seyed Aria Nejadghaderi, Maryam Noori, Hassan Okati-Aliabad, Morteza Oladnabi, Babak Pakbin, Fatemeh PashazadehKan, Hamidreza PazokiToroudi, Naeimeh Pourtaheri, Navid Rabiee, Sima Rafiei, Fakher Rahim, Vahid Rahmanian, Samira Raoofi, Mahsa Rashidi, Mohammad-Mahdi Rashidi, Mohammad Sadegh Razeghinia, Nima Rezaei, Saeid Rezaei, Aziz Rezapour, Gholamreza Roshandel, Siamak Sabour, Maryam Sahebazzamani, Amirhossein Sahebkar, Soraya Sajadimajd, Sadaf G. Sepanlou, Saeed Shahabi, Fariba Shahraki-Sanavi, Javad Sharifi-Rad, Reza Shirkoohi, Parnian Shobeiri, Mohammad Sadegh Soltani-Zangbar, Elnaz Tabibian, Majid Taheri, Yasaman TaheriAbkenar, Ahmad Tavakoli, Amir Tiyuri, Seyed Abolfazl Tohidast, Sahel ValadanTahbaz, Rohollah Valizadeh, Seyed Hossein YahyazadehJabbari, Leila Zaki, Maryam Zamanian, Iman Zare, Mohammad Zoladl

**Affiliations:** 1 Non-Communicable Diseases Research Center, Endocrinology and Metabolism Population Sciences Institute, Tehran University of Medical Sciences, Second Floor, No.10, Jalal Al-E-Ahmad Highway, Tehran, 1411713137 Iran; 2 Division of Pulmonary, Allergy, and Critical Care Medicine, University of Pittsburgh, Pittsburgh, PA USA; 3 Endocrinology and Metabolism Research Center, Endocrinology and Metabolism Clinical Sciences Institute, Tehran University of Medical Sciences, Tehran, Iran; 4 Institute for Health Metrics and Evaluation, University of Washington, Seattle, WA USA

**Correction to: Breast Cancer Research (2023) 25:47** 10.1186/s13058-023-01633-4

In the original publication of this article the contributions of the GBD 2019 Iran Breast Cancer Collaborators were not included. The full list of collaborators and their contributions are listed in the supplementary file and the individual collaborators are now listed in the original article.

