# Supplementary material for: National and subnational burden of female and male breast cancer and risk factors in Iran from 1990 to 2019: results from the Global Burden of Disease study 2019
Source: Breast Cancer Res. 2023 Apr 26;25:47. doi: 10.1186/s13058-023-01633-4 (PMC10131337; doi:10.1186/s13058-023-01633-4)

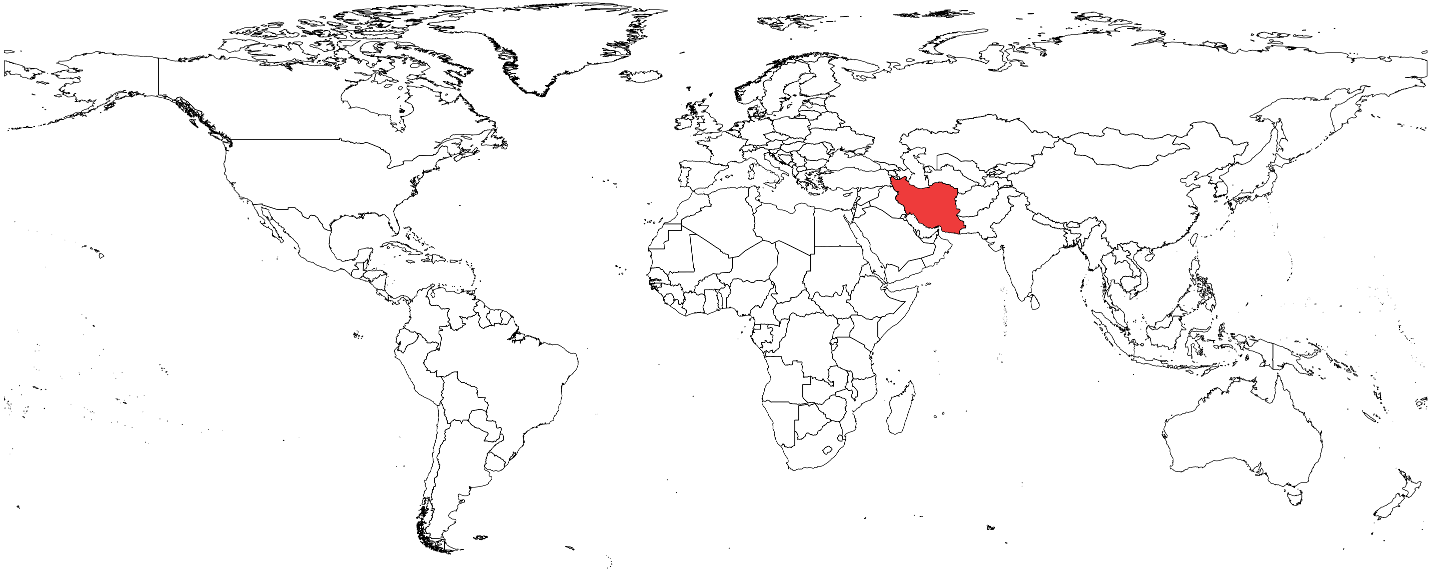

Alborz (AL)
Ardebil (AR)
Bushehr (BS)
Chahar Mahaal and Bakhtiari (CM)
East Azarbayejan (EA)
Fars (FA)
Gilan (GI)
Golestan (GO)
Hamadan (HD)
Hormozgan (HG)
Ilam (IL)
Isfahan (ES)
Kerman (KE)
Kermanshah (BK)
Khorasan-e-Razavi (KV)
Khuzestan (KZ)
Kohgiluyeh and Boyer-Ahmad (KB)
Kurdistan (KD)
Lorestan (LO)
Markazi (MK)
Mazandaran (MN)
North Khorasan (KS)
Qazvin (QZ)
Qom (QM)
Semnan (SM)
Sistan and Baluchistan (SB)
South Khorasan (KJ)
Tehran (TE)
West Azarbayejan (WA)
Yazd (YA)
Zanjan (ZA)

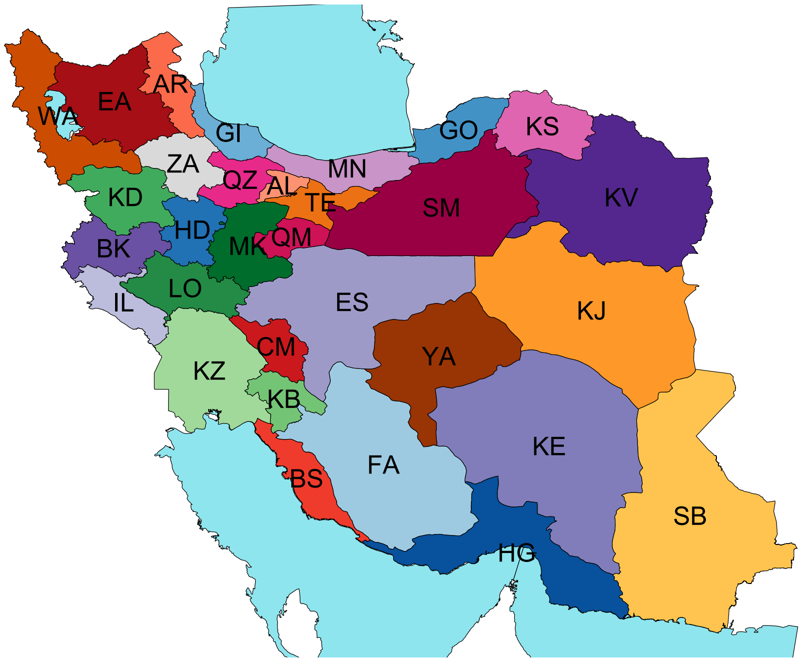

Supplement: Supplementary file 1 — Additional file 1. Fig. 1 The geographical location of Iran in the globe (shown in red color) and its subnational administrative divisions. [file 13058_2023_1633_MOESM1_ESM.pdf]
