# Supplementary material for: National and subnational burden of female and male breast cancer and risk factors in Iran from 1990 to 2019: results from the Global Burden of Disease study 2019
Source: Breast Cancer Res. 2023 Apr 26;25:47. doi: 10.1186/s13058-023-01633-4 (PMC10131337; doi:10.1186/s13058-023-01633-4)
Supplement: Supplementary file 3 — Additional file 3. Table 2 Annual percent changes of breast cancer age-standardized incidence, prevalence, deaths, disability-adjusted life years (DALYs), years of life lost (YLLs) and years lived with disability (YLDs) rates from 1990 to 2019 at the national and subnational levels. [file 13058_2023_1633_MOESM3_ESM.pdf]

Location	Measure	APC (1990 to 2019)		
		Both	Female	Male
Iran	Incidence	1.98 (1.80 to 2.15)	1.87 (1.69 to 2.05)	0.39 (0.05 to 0.74)
	Prevalence	2.02 (1.86 to 2.17)	1.89 (1.74 to 2.05)	0.87 (0.68 to 1.05)
	Deaths	0.58 (0.41 to 0.75)	0.50 (0.34 to 0.66)	-0.76 (-1.07 to -0.44)
	DALYs	0.63 (0.48 to 0.78)	0.51 (0.36 to 0.65)	-0.70 (-0.99 to -0.42)
	YLLs	0.56 (0.41 to 0.70)	0.43 (0.29 to 0.58)	-0.77 (-1.06 to -0.49)
	YLDs	2.04 (1.89 to 2.20)	1.93 (1.77 to 2.09)	0.73 (0.45 to 1.01)
Alborz	Incidence	1.36 (1.14 to 1.58)	1.20 (0.97 to 1.44)	0.54 (0.04 to 1.04)
	Prevalence	1.62 (1.46 to 1.77)	1.43 (1.27 to 1.60)	1.09 (0.76 to 1.42)
	Deaths	-0.28 (-0.45 to -0.11)	-0.33 (-0.49 to -0.16)	-0.94 (-1.49 to -0.39)
	DALYs	-0.31 (-0.47 to -0.15)	-0.52 (-0.69 to -0.34)	-0.76 (-1.18 to -0.34)
	YLLs	-0.42 (-0.58 to -0.26)	-0.62 (-0.79 to -0.45)	-0.85 (-1.27 to -0.43)
	YLDs	1.52 (1.34 to 1.70)	1.35 (1.15 to 1.55)	0.91 (0.47 to 1.34)
Ardebil	Incidence	1.39 (1.08 to 1.70)	1.19 (0.88 to 1.49)	0.19 (-0.46 to 0.83)
	Prevalence	1.62 (1.40 to 1.85)	1.40 (1.18 to 1.62)	0.64 (0.22 to 1.06)
	Deaths	0.05 (-0.14 to 0.23)	-0.23 (-0.42 to -0.03)	-0.88 (-1.41 to -0.34)
	DALYs	-0.20 (-0.40 to 0.01)	-0.38 (-0.59 to -0.17)	-1.09 (-1.63 to -0.54)
	YLLs	-0.28 (-0.48 to -0.08)	-0.46 (-0.68 to -0.25)	-1.17 (-1.71 to -0.62)
	YLDs	1.58 (1.32 to 1.85)	1.39 (1.13 to 1.65)	0.56 (0.00 to 1.12)

Province	Measure	APC (1990 to 2019)		
		Both	Female	Male
Bushehr	Incidence	2.14 (1.98 to 2.30)	2.32 (2.18 to 2.47)	0.70 (0.03 to 1.38)
	Prevalence	2.22 (2.13 to 2.31)	2.38 (2.30 to 2.46)	1.13 (0.73 to 1.53)
	Deaths	0.67 (0.56 to 0.78)	0.77 (0.64 to 0.91)	-0.39 (-1.03 to 0.26)
	DALYs	0.63 (0.55 to 0.72)	0.81 (0.70 to 0.92)	-0.48 (-1.13 to 0.17)
	YLLs	0.55 (0.46 to 0.64)	0.73 (0.61 to 0.84)	-0.56 (-1.22 to 0.09)
	YLDs	2.22 (2.11 to 2.33)	2.40 (2.31 to 2.50)	1.01 (0.44 to 1.58)
Chahar Mahaal and Bakhtiari	Incidence	1.19 (0.63 to 1.76)	1.01 (0.45 to 1.58)	0.00 (-0.82 to 0.83)
	Prevalence	1.46 (1.26 to 1.67)	1.26 (1.06 to 1.47)	0.58 (0.13 to 1.03)
	Deaths	-0.24 (-0.49 to 0.01)	-0.45 (-0.70 to -0.20)	-1.13 (-1.80 to -0.46)
	DALYs	-0.27 (-0.50 to -0.03)	-0.45 (-0.67 to -0.22)	-1.15 (-1.81 to -0.48)
	YLLs	-0.36 (-0.59 to -0.13)	-0.54 (-0.76 to -0.31)	-1.24 (-1.91 to -0.57)
	YLDs	1.38 (1.11 to 1.65)	1.20 (0.93 to 1.47)	0.39 (-0.25 to 1.04)
East Azarbayejan	Incidence	2.62 (2.37 to 2.86)	2.50 (2.24 to 2.75)	1.81 (1.61 to 2.02)
	Prevalence	2.53 (2.29 to 2.77)	2.38 (2.14 to 2.63)	1.88 (1.71 to 2.04)
	Deaths	1.00 (0.78 to 1.21)	0.84 (0.63 to 1.05)	0.65 (0.45 to 0.84)
	DALYs	1.00 (0.80 to 1.21)	0.89 (0.68 to 1.10)	0.64 (0.44 to 0.85)
	YLLs	0.93 (0.73 to 1.13)	0.81 (0.60 to 1.02)	0.58 (0.38 to 0.78)
	YLDs	2.62 (2.39 to 2.86)	2.50 (2.26 to 2.74)	2.01 (1.84 to 2.18)

Province	Measure	APC (1990 to 2019)		
		Both	Female	Male
Fars	Incidence	2.84 (2.67 to 3.02)	2.76 (2.59 to 2.94)	1.39 (1.20 to 1.59)
	Prevalence	2.68 (2.48 to 2.88)	2.59 (2.40 to 2.78)	1.57 (1.41 to 1.74)
	Deaths	1.23 (1.04 to 1.42)	1.19 (1.02 to 1.36)	0.20 (0.03 to 0.37)
	DALYs	1.37 (1.19 to 1.55)	1.27 (1.10 to 1.44)	0.33 (0.15 to 0.51)
	YLLs	1.29 (1.11 to 1.47)	1.19 (1.02 to 1.36)	0.26 (0.08 to 0.45)
	YLDs	2.82 (2.63 to 3.01)	2.73 (2.55 to 2.92)	1.59 (1.42 to 1.76)
Gilan	Incidence	2.42 (2.32 to 2.52)	2.42 (2.32 to 2.53)	0.43 (0.10 to 0.76)
	Prevalence	2.39 (2.31 to 2.46)	2.40 (2.31 to 2.48)	0.96 (0.71 to 1.20)
	Deaths	0.94 (0.81 to 1.07)	1.01 (0.89 to 1.12)	-0.75 (-1.04 to -0.46)
	DALYs	1.02 (0.90 to 1.13)	0.99 (0.88 to 1.10)	-0.64 (-0.89 to -0.38)
	YLLs	0.94 (0.82 to 1.06)	0.91 (0.80 to 1.03)	-0.71 (-0.97 to -0.45)
	YLDs	2.43 (2.35 to 2.52)	2.44 (2.34 to 2.54)	0.79 (0.50 to 1.09)
Golestan	Incidence	2.48 (2.20 to 2.77)	2.38 (2.09 to 2.67)	0.63 (0.11 to 1.14)
	Prevalence	2.53 (2.29 to 2.76)	2.41 (2.17 to 2.65)	1.13 (0.85 to 1.40)
	Deaths	0.92 (0.69 to 1.14)	0.82 (0.59 to 1.05)	-0.56 (-1.04 to -0.07)
	DALYs	1.02 (0.76 to 1.29)	0.91 (0.62 to 1.19)	-0.39 (-0.84 to 0.07)
	YLLs	0.96 (0.69 to 1.23)	0.84 (0.55 to 1.12)	-0.45 (-0.91 to 0.01)
	YLDs	2.54 (2.29 to 2.79)	2.43 (2.17 to 2.69)	1.02 (0.60 to 1.44)

Province	Measure	APC (1990 to 2019)		
		Both	Female	Male
Hamadan	Incidence	1.74 (1.61 to 1.88)	1.63 (1.47 to 1.78)	0.12 (-0.22 to 0.45)
	Prevalence	1.81 (1.71 to 1.92)	1.67 (1.54 to 1.79)	0.55 (0.39 to 0.72)
	Deaths	0.36 (0.24 to 0.49)	0.23 (0.09 to 0.36)	-0.93 (-1.20 to -0.66)
	DALYs	0.37 (0.28 to 0.46)	0.26 (0.14 to 0.37)	-0.93 (-1.20 to -0.66)
	YLLs	0.30 (0.21 to 0.39)	0.19 (0.07 to 0.30)	-0.99 (-1.26 to -0.72)
	YLDs	1.82 (1.71 to 1.93)	1.70 (1.57 to 1.83)	0.46 (0.20 to 0.72)
Hormozgan	Incidence	2.69 (2.58 to 2.80)	2.53 (2.41 to 2.65)	-0.44 (-1.07 to 0.19)
	Prevalence	2.80 (2.64 to 2.96)	2.60 (2.43 to 2.76)	0.49 (0.07 to 0.92)
	Deaths	0.93 (0.81 to 1.04)	0.80 (0.69 to 0.91)	-1.88 (-2.52 to -1.24)
	DALYs	0.84 (0.76 to 0.93)	0.66 (0.58 to 0.74)	-1.75 (-2.31 to -1.19)
	YLLs	0.76 (0.67 to 0.85)	0.58 (0.49 to 0.66)	-1.83 (-2.39 to -1.27)
	YLDs	2.81 (2.72 to 2.90)	2.64 (2.54 to 2.74)	0.12 (-0.43 to 0.67)
Ilam	Incidence	3.31 (3.11 to 3.51)	2.93 (2.73 to 3.14)	1.60 (0.87 to 2.33)
	Prevalence	3.34 (3.22 to 3.46)	2.92 (2.80 to 3.05)	1.95 (1.44 to 2.46)
	Deaths	1.80 (1.63 to 1.96)	1.41 (1.25 to 1.58)	0.33 (-0.34 to 1.01)
	DALYs	1.75 (1.63 to 1.87)	1.37 (1.24 to 1.50)	0.47 (-0.19 to 1.14)
	YLLs	1.67 (1.55 to 1.80)	1.29 (1.16 to 1.42)	0.40 (-0.26 to 1.07)
	YLDs	3.35 (3.24 to 3.46)	2.97 (2.86 to 3.09)	1.89 (1.24 to 2.54)

Province	Measure	APC (1990 to 2019)		
		Both	Female	Male
Isfahan	Incidence	2.19 (2.00 to 2.37)	2.15 (1.97 to 2.34)	1.38 (1.14 to 1.61)
	Prevalence	2.09 (1.92 to 2.25)	2.06 (1.90 to 2.21)	1.52 (1.33 to 1.72)
	Deaths	0.81 (0.65 to 0.97)	0.85 (0.71 to 0.99)	0.31 (0.11 to 0.51)
	DALYs	0.96 (0.84 to 1.07)	0.89 (0.79 to 1.00)	0.47 (0.26 to 0.67)
	YLLs	0.89 (0.77 to 1.00)	0.82 (0.72 to 0.92)	0.41 (0.20 to 0.61)
	YLDs	2.19 (2.02 to 2.36)	2.15 (1.99 to 2.32)	1.56 (1.36 to 1.77)
Kerman	Incidence	1.85 (1.72 to 1.97)	1.75 (1.62 to 1.89)	0.60 (0.21 to 0.99)
	Prevalence	1.96 (1.87 to 2.04)	1.85 (1.76 to 1.95)	1.07 (0.85 to 1.30)
	Deaths	0.37 (0.29 to 0.45)	0.29 (0.21 to 0.37)	-0.46 (-0.81 to -0.12)
	DALYs	0.39 (0.33 to 0.46)	0.28 (0.21 to 0.36)	-0.45 (-0.79 to -0.11)
	YLLs	0.32 (0.26 to 0.39)	0.21 (0.14 to 0.29)	-0.51 (-0.85 to -0.17)
	YLDs	1.91 (1.80 to 2.02)	1.82 (1.69 to 1.94)	0.88 (0.54 to 1.23)
Kermanshah	Incidence	2.37 (2.27 to 2.46)	2.08 (1.97 to 2.18)	0.51 (0.15 to 0.88)
	Prevalence	2.48 (2.41 to 2.55)	2.15 (2.06 to 2.24)	1.10 (0.88 to 1.32)
	Deaths	0.84 (0.76 to 0.92)	0.54 (0.46 to 0.61)	-0.72 (-1.06 to -0.38)
	DALYs	0.82 (0.77 to 0.88)	0.54 (0.48 to 0.60)	-0.56 (-0.89 to -0.22)
	YLLs	0.75 (0.69 to 0.81)	0.47 (0.40 to 0.53)	-0.62 (-0.96 to -0.29)
	YLDs	2.47 (2.40 to 2.54)	2.17 (2.08 to 2.26)	0.89 (0.58 to 1.21)

Province	Measure	APC (1990 to 2019)		
		Both	Female	Male
Khorasan-e-Razavi	Incidence	2.23 (1.95 to 2.50)	2.08 (1.78 to 2.37)	0.45 (0.20 to 0.70)
	Prevalence	2.32 (2.06 to 2.57)	2.15 (1.88 to 2.42)	0.96 (0.73 to 1.18)
	Deaths	0.65 (0.44 to 0.86)	0.47 (0.26 to 0.68)	-0.68 (-0.87 to -0.49)
	DALYs	0.67 (0.46 to 0.88)	0.52 (0.30 to 0.74)	-0.71 (-0.90 to -0.52)
	YLLs	0.60 (0.39 to 0.80)	0.45 (0.23 to 0.67)	-0.78 (-0.97 to -0.59)
	YLDs	2.32 (2.07 to 2.57)	2.17 (1.90 to 2.43)	0.81 (0.59 to 1.03)
Khuzestan	Incidence	2.72 (2.53 to 2.91)	2.65 (2.48 to 2.83)	-0.47 (-0.88 to -0.06)
	Prevalence	2.68 (2.47 to 2.89)	2.60 (2.41 to 2.78)	0.25 (0.02 to 0.49)
	Deaths	1.15 (0.99 to 1.30)	1.11 (0.98 to 1.24)	-1.60 (-1.98 to -1.23)
	DALYs	1.25 (1.09 to 1.40)	1.16 (1.02 to 1.30)	-1.59 (-1.93 to -1.24)
	YLLs	1.18 (1.02 to 1.33)	1.09 (0.95 to 1.23)	-1.66 (-2.01 to -1.32)
	YLDs	2.74 (2.56 to 2.92)	2.67 (2.50 to 2.83)	-0.02 (-0.35 to 0.31)
Kohgiluyeh and Boyer-Ahmad	Incidence	2.72 (2.45 to 2.99)	2.69 (2.41 to 2.97)	-0.73 (-1.46 to 0.00)
	Prevalence	2.68 (2.44 to 2.93)	2.62 (2.36 to 2.87)	0.15 (-0.35 to 0.66)
	Deaths	0.82 (0.64 to 1.00)	0.95 (0.75 to 1.14)	-2.00 (-2.69 to -1.30)
	DALYs	1.11 (0.97 to 1.25)	1.02 (0.86 to 1.18)	-2.05 (-2.70 to -1.39)
	YLLs	1.02 (0.88 to 1.16)	0.93 (0.77 to 1.09)	-2.14 (-2.80 to -1.48)
	YLDs	2.82 (2.63 to 3.00)	2.78 (2.57 to 2.98)	-0.19 (-0.82 to 0.45)

Province	Measure	APC (1990 to 2019)		
		Both	Female	Male
Kurdistan	**Incidence**	2.21 (2.12 to 2.30)	1.96 (1.85 to 2.07)	0.26 (-0.14 to 0.67)
	**Prevalence**	2.26 (2.14 to 2.38)	1.97 (1.84 to 2.11)	0.86 (0.64 to 1.08)
	**Deaths**	0.89 (0.77 to 1.01)	0.62 (0.51 to 0.72)	-0.94 (-1.36 to -0.52)
	**DALYs**	0.68 (0.61 to 0.76)	0.44 (0.37 to 0.51)	-0.78 (-1.19 to -0.37)
	**YLLs**	0.61 (0.53 to 0.69)	0.37 (0.30 to 0.44)	-0.85 (-1.26 to -0.43)
	**YLDs**	2.27 (2.18 to 2.37)	2.02 (1.91 to 2.13)	0.67 (0.34 to 1.00)
Lorestan	**Incidence**	1.90 (1.75 to 2.06)	1.59 (1.43 to 1.76)	-0.11 (-0.72 to 0.50)
	**Prevalence**	2.09 (1.99 to 2.18)	1.75 (1.63 to 1.86)	0.48 (0.10 to 0.87)
	**Deaths**	0.21 (0.13 to 0.29)	-0.11 (-0.20 to -0.03)	-1.38 (-1.98 to -0.77)
	**DALYs**	0.24 (0.15 to 0.34)	-0.07 (-0.15 to 0.01)	-1.47 (-2.03 to -0.91)
	**YLLs**	0.16 (0.06 to 0.25)	-0.16 (-0.24 to -0.08)	-1.55 (-2.11 to -0.99)
	**YLDs**	2.04 (1.92 to 2.16)	1.73 (1.60 to 1.86)	0.31 (-0.21 to 0.83)
Markazi	**Incidence**	1.78 (1.64 to 1.91)	1.78 (1.64 to 1.93)	0.75 (0.26 to 1.25)
	**Prevalence**	1.90 (1.77 to 2.02)	1.88 (1.74 to 2.01)	1.20 (0.89 to 1.50)
	**Deaths**	0.31 (0.19 to 0.42)	0.28 (0.17 to 0.39)	-0.43 (-0.90 to 0.04)
	**DALYs**	0.30 (0.22 to 0.38)	0.31 (0.22 to 0.40)	-0.35 (-0.83 to 0.14)
	**YLLs**	0.22 (0.15 to 0.30)	0.23 (0.15 to 0.32)	-0.42 (-0.90 to 0.07)
	**YLDs**	1.88 (1.75 to 2.01)	1.88 (1.74 to 2.02)	1.09 (0.68 to 1.50)

Province	Measure	APC (1990 to 2019)		
		Both	Female	Male
Mazandaran	Incidence	2.54 (2.37 to 2.71)	2.52 (2.35 to 2.69)	0.29 (-0.16 to 0.74)
	Prevalence	2.45 (2.31 to 2.59)	2.42 (2.28 to 2.57)	0.67 (0.41 to 0.93)
	Deaths	0.99 (0.82 to 1.16)	0.99 (0.82 to 1.15)	-0.86 (-1.27 to -0.46)
	DALYs	1.16 (0.99 to 1.33)	1.13 (0.95 to 1.31)	-0.82 (-1.20 to -0.44)
	YLLs	1.08 (0.91 to 1.25)	1.05 (0.87 to 1.23)	-0.90 (-1.28 to -0.52)
	YLDs	2.53 (2.39 to 2.68)	2.52 (2.37 to 2.67)	0.58 (0.21 to 0.95)
North Khorasan	Incidence	2.09 (1.81 to 2.37)	1.94 (1.65 to 2.25)	-0.65 (-1.19 to -0.10)
	Prevalence	2.28 (2.05 to 2.50)	2.09 (1.84 to 2.34)	0.22 (-0.15 to 0.60)
	Deaths	0.40 (0.15 to 0.64)	0.26 (-0.01 to 0.52)	-1.85 (-2.36 to -1.34)
	DALYs	0.44 (0.18 to 0.70)	0.29 (0.00 to 0.59)	-1.87 (-2.34 to -1.40)
	YLLs	0.36 (0.10 to 0.62)	0.22 (-0.08 to 0.51)	-1.94 (-2.42 to -1.47)
	YLDs	2.26 (2.03 to 2.49)	2.11 (1.86 to 2.37)	-0.10 (-0.58 to 0.39)
Qazvin	Incidence	2.82 (2.69 to 2.95)	2.77 (2.64 to 2.90)	0.02 (-0.60 to 0.65)
	Prevalence	2.81 (2.65 to 2.97)	2.74 (2.59 to 2.89)	0.63 (0.25 to 1.02)
	Deaths	1.15 (0.95 to 1.35)	1.07 (0.89 to 1.26)	-1.14 (-1.70 to -0.57)
	DALYs	1.20 (1.03 to 1.37)	1.14 (0.97 to 1.31)	-1.26 (-1.80 to -0.72)
	YLLs	1.12 (0.95 to 1.29)	1.06 (0.89 to 1.23)	-1.34 (-1.89 to -0.79)
	YLDs	2.84 (2.72 to 2.95)	2.78 (2.67 to 2.90)	0.43 (-0.10 to 0.96)

Province	Measure	APC (1990 to 2019)		
		Both	Female	Male
Qom	Incidence	2.00 (1.87 to 2.12)	2.04 (1.91 to 2.17)	-1.39 (-2.19 to -0.58)
Qom	Prevalence	2.18 (2.12 to 2.25)	2.18 (2.12 to 2.25)	-0.36 (-0.95 to 0.23)
Qom	Deaths	0.37 (0.27 to 0.48)	0.52 (0.42 to 0.63)	-2.58 (-3.35 to -1.81)
Qom	DALYs	0.39 (0.29 to 0.49)	0.40 (0.30 to 0.49)	-2.62 (-3.36 to -1.87)
Qom	YLLs	0.31 (0.20 to 0.41)	0.32 (0.22 to 0.42)	-2.70 (-3.44 to -1.94)
Qom	YLDs	2.16 (2.08 to 2.24)	2.19 (2.10 to 2.27)	-0.81 (-1.53 to -0.08)
Semnan	Incidence	2.50 (2.35 to 2.66)	2.49 (2.34 to 2.65)	2.09 (1.51 to 2.68)
Semnan	Prevalence	2.59 (2.50 to 2.68)	2.58 (2.50 to 2.66)	2.44 (1.99 to 2.89)
Semnan	Deaths	0.88 (0.71 to 1.04)	0.87 (0.72 to 1.01)	0.85 (0.30 to 1.41)
Semnan	DALYs	0.90 (0.78 to 1.02)	0.88 (0.77 to 0.99)	0.79 (0.24 to 1.35)
Semnan	YLLs	0.81 (0.69 to 0.94)	0.79 (0.68 to 0.91)	0.72 (0.17 to 1.27)
Semnan	YLDs	2.62 (2.53 to 2.71)	2.61 (2.53 to 2.69)	2.33 (1.79 to 2.87)
Sistan and Baluchistan	Incidence	1.83 (1.67 to 2.00)	1.40 (1.20 to 1.60)	-0.89 (-1.44 to -0.34)
Sistan and Baluchistan	Prevalence	2.00 (1.81 to 2.19)	1.56 (1.35 to 1.76)	-0.01 (-0.29 to 0.27)
Sistan and Baluchistan	Deaths	0.63 (0.51 to 0.75)	0.21 (0.08 to 0.34)	-1.66 (-2.18 to -1.15)
Sistan and Baluchistan	DALYs	0.73 (0.62 to 0.83)	0.31 (0.18 to 0.44)	-1.81 (-2.29 to -1.32)
Sistan and Baluchistan	YLLs	0.68 (0.58 to 0.79)	0.26 (0.14 to 0.39)	-1.86 (-2.35 to -1.37)
Sistan and Baluchistan	YLDs	2.03 (1.88 to 2.18)	1.61 (1.42 to 1.79)	-0.37 (-0.80 to 0.07)

Province	Measure	APC (1990 to 2019)		
		Both	Female	Male
South Khorasan	Incidence	2.08 (1.92 to 2.25)	1.87 (1.69 to 2.05)	-0.67 (-1.33 to 0.00)
	Prevalence	2.17 (2.04 to 2.30)	1.93 (1.78 to 2.09)	0.06 (-0.40 to 0.51)
	Deaths	0.58 (0.43 to 0.73)	0.34 (0.18 to 0.50)	-1.65 (-2.25 to -1.05)
	DALYs	0.59 (0.47 to 0.72)	0.38 (0.22 to 0.54)	-1.83 (-2.45 to -1.20)
	YLLs	0.52 (0.40 to 0.65)	0.31 (0.15 to 0.47)	-1.90 (-2.53 to -1.27)
	YLDs	2.18 (2.05 to 2.31)	1.97 (1.81 to 2.13)	-0.22 (-0.80 to 0.37)
Tehran	Incidence	0.84 (0.60 to 1.09)	0.75 (0.53 to 0.98)	-0.76 (-1.03 to -0.48)
	Prevalence	0.91 (0.74 to 1.09)	0.82 (0.66 to 0.97)	-0.27 (-0.47 to -0.07)
	Deaths	-0.12 (-0.45 to 0.21)	-0.11 (-0.40 to 0.18)	-1.51 (-1.68 to -1.34)
	DALYs	-0.05 (-0.35 to 0.24)	-0.18 (-0.44 to 0.09)	-1.47 (-1.64 to -1.30)
	YLLs	-0.11 (-0.41 to 0.19)	-0.24 (-0.51 to 0.03)	-1.54 (-1.70 to -1.37)
	YLDs	0.91 (0.71 to 1.12)	0.82 (0.63 to 1.01)	-0.46 (-0.70 to -0.22)
West Azarbayejan	Incidence	2.02 (1.67 to 2.37)	1.83 (1.47 to 2.20)	0.36 (0.06 to 0.67)
	Prevalence	2.09 (1.79 to 2.40)	1.89 (1.56 to 2.22)	0.85 (0.68 to 1.01)
	Deaths	0.52 (0.23 to 0.81)	0.30 (0.00 to 0.61)	-0.77 (-1.06 to -0.48)
	DALYs	0.45 (0.17 to 0.74)	0.28 (-0.03 to 0.59)	-0.79 (-1.06 to -0.51)
	YLLs	0.38 (0.09 to 0.66)	0.21 (-0.10 to 0.51)	-0.86 (-1.13 to -0.58)
	YLDs	2.12 (1.81 to 2.43)	1.93 (1.60 to 2.26)	0.74 (0.51 to 0.97)

Province	Measure	APC (1990 to 2019)		
		Both	Female	Male
Yazd	Incidence	2.45 (2.35 to 2.56)	2.52 (2.41 to 2.64)	2.42 (1.91 to 2.92)
	Prevalence	2.49 (2.41 to 2.57)	2.58 (2.50 to 2.65)	2.66 (2.29 to 3.03)
	Deaths	0.64 (0.52 to 0.77)	0.71 (0.62 to 0.81)	1.05 (0.54 to 1.57)
	DALYs	0.63 (0.54 to 0.71)	0.67 (0.60 to 0.73)	1.02 (0.52 to 1.51)
	YLLs	0.53 (0.44 to 0.62)	0.56 (0.49 to 0.64)	0.94 (0.44 to 1.44)
	YLDs	2.56 (2.48 to 2.63)	2.63 (2.55 to 2.71)	2.57 (2.12 to 3.02)
Zanjan	Incidence	3.00 (2.83 to 3.16)	2.85 (2.69 to 3.00)	1.34 (0.76 to 1.92)
	Prevalence	2.81 (2.56 to 3.05)	2.64 (2.40 to 2.88)	1.56 (1.26 to 1.86)
	Deaths	1.52 (1.33 to 1.71)	1.36 (1.18 to 1.53)	0.16 (-0.44 to 0.76)
	DALYs	1.36 (1.22 to 1.50)	1.20 (1.07 to 1.33)	0.08 (-0.55 to 0.70)
	YLLs	1.28 (1.14 to 1.43)	1.13 (0.99 to 1.26)	0.00 (-0.63 to 0.64)
	YLDs	2.95 (2.77 to 3.12)	2.79 (2.62 to 2.97)	1.60 (1.14 to 2.06)

Data in parentheses are 95% Confidence Intervals (95% CIs)

APC= Annual Percent Change; DALYs=Disability-Adjusted Life Years; YLLs=Years of Life Lost; YLDs=Years Lived with Disability
