# Supplementary figures and images for: National and subnational burden of female and male breast cancer and risk factors in Iran from 1990 to 2019: results from the Global Burden of Disease study 2019
Source: Breast Cancer Res. 2023 Apr 26;25:47. doi: 10.1186/s13058-023-01633-4 (PMC10131337; doi:10.1186/s13058-023-01633-4)

Incidence

Deaths

1990

2019

1990

2019

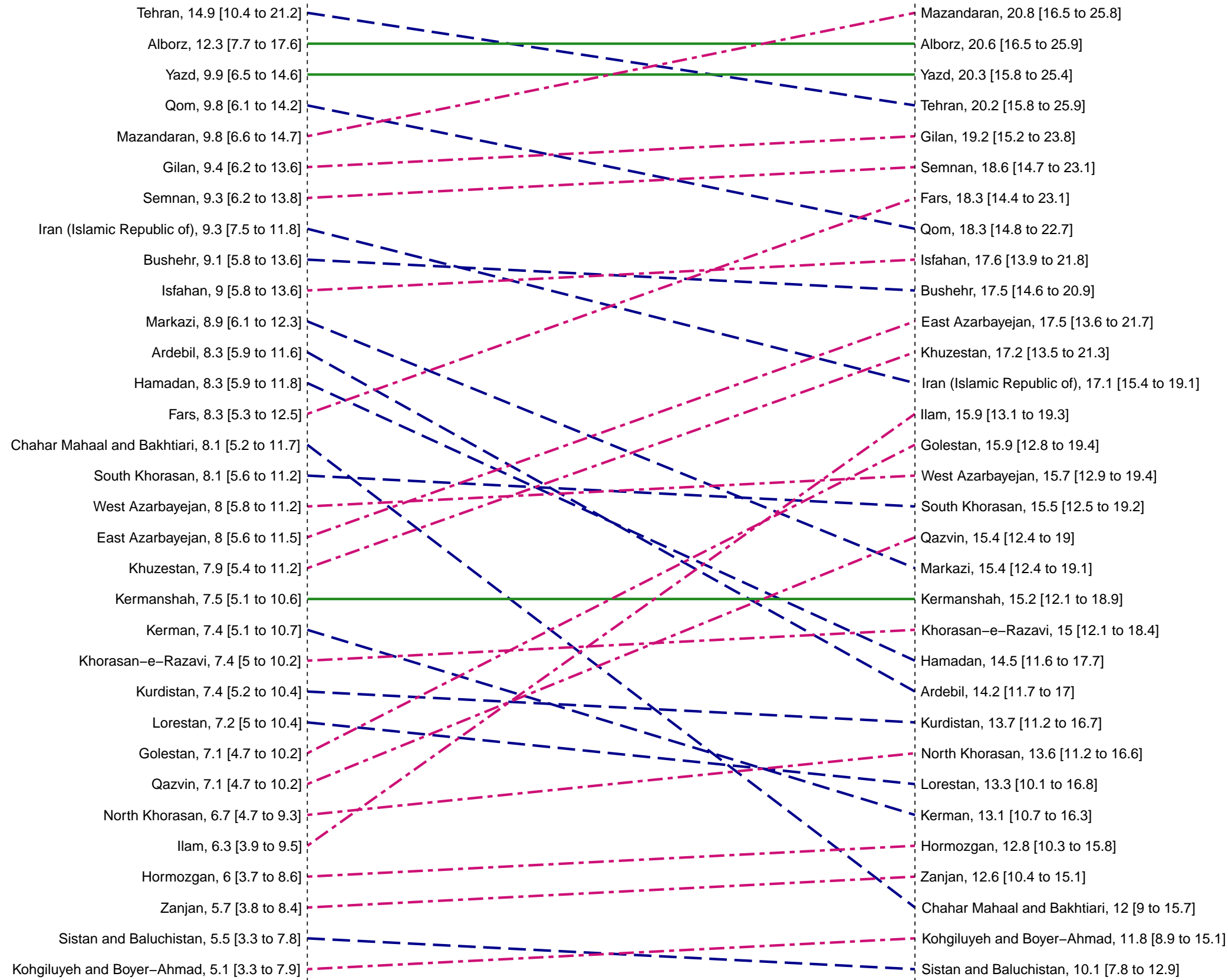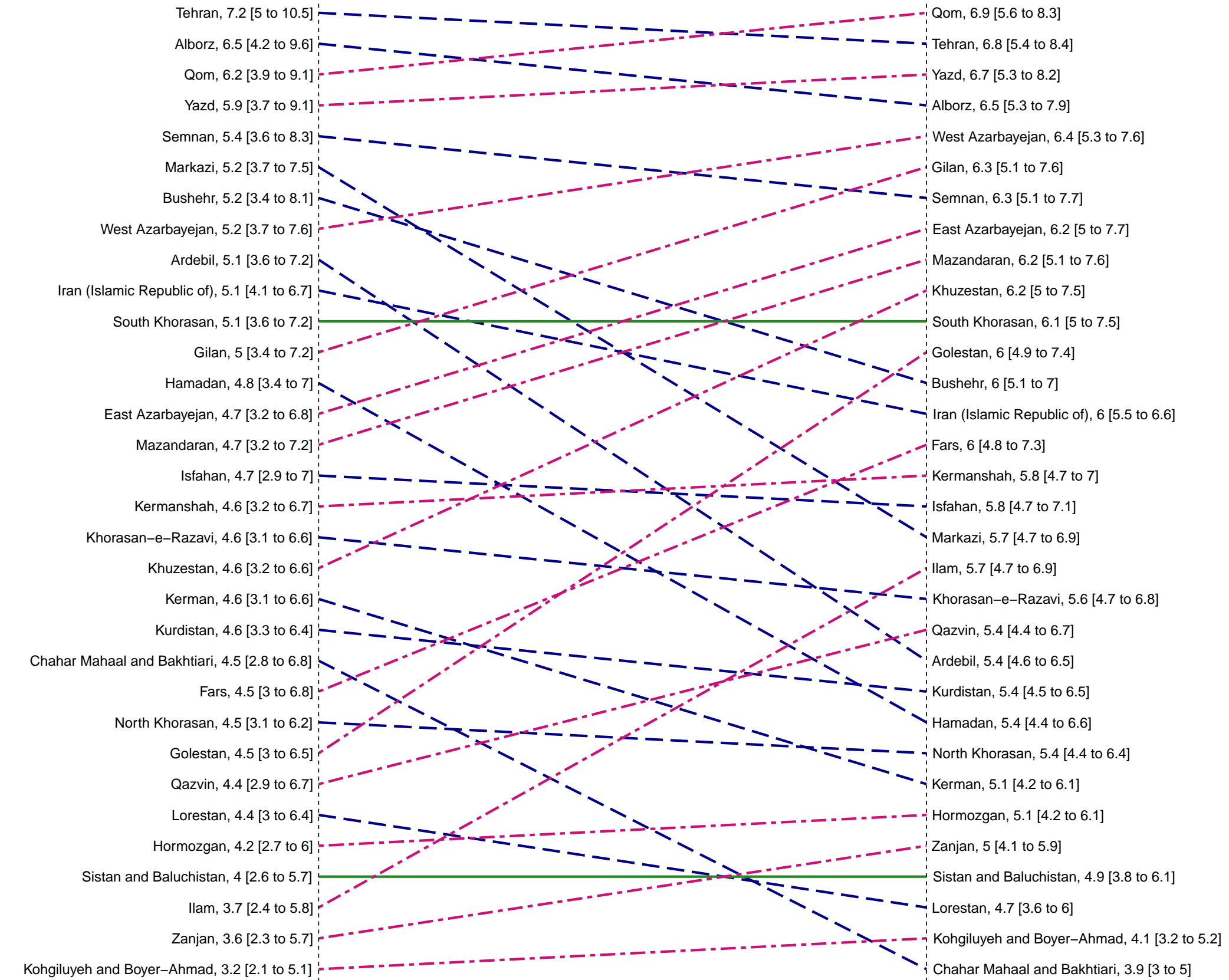

Ranking

Upward

Monotone

Downward

Supplement: Supplementary file 4 — Additional file 4. Fig. 2A Breast cancer age-standardized incidence and deaths rates (per 100,000 population) rankings in Iran and its 31 provinces in 1990 and 2019, for A) both sexes, B) females, and C) males. [file 13058_2023_1633_MOESM4_ESM.pdf]

Incidence

Deaths

1990

2019

1990

2019

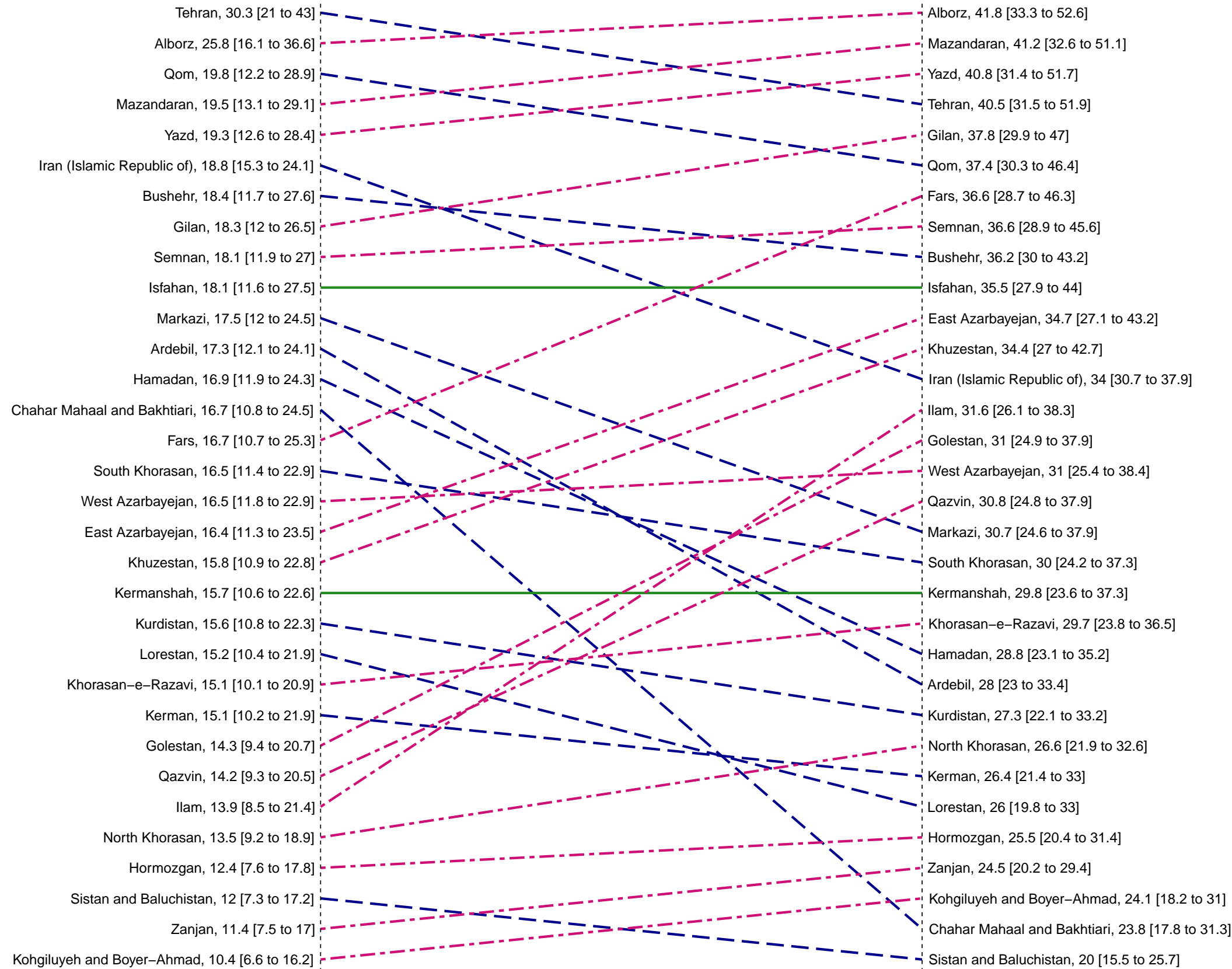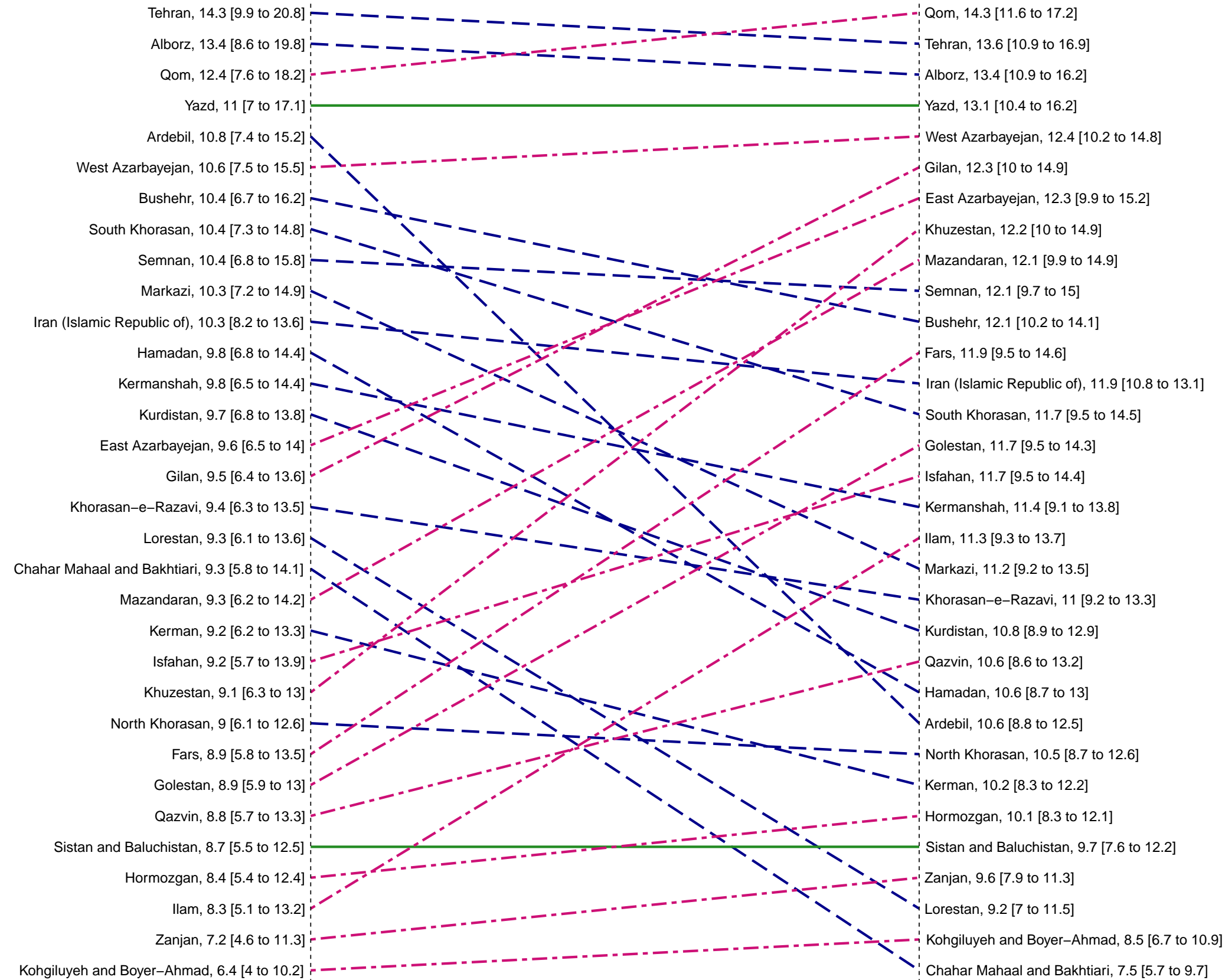

Ranking

Upward

Monotone

Downward

Supplement: Supplementary file 5 — Additional file 5. Fig. 2B Breast cancer age-standardized incidence and deaths rates (per 100,000 population) rankings in Iran and its 31 provinces in 1990 and 2019, for A) both sexes, B) females, and C) males. [file 13058_2023_1633_MOESM5_ESM.pdf]

Incidence

Deaths

1990

2019

1990

2019

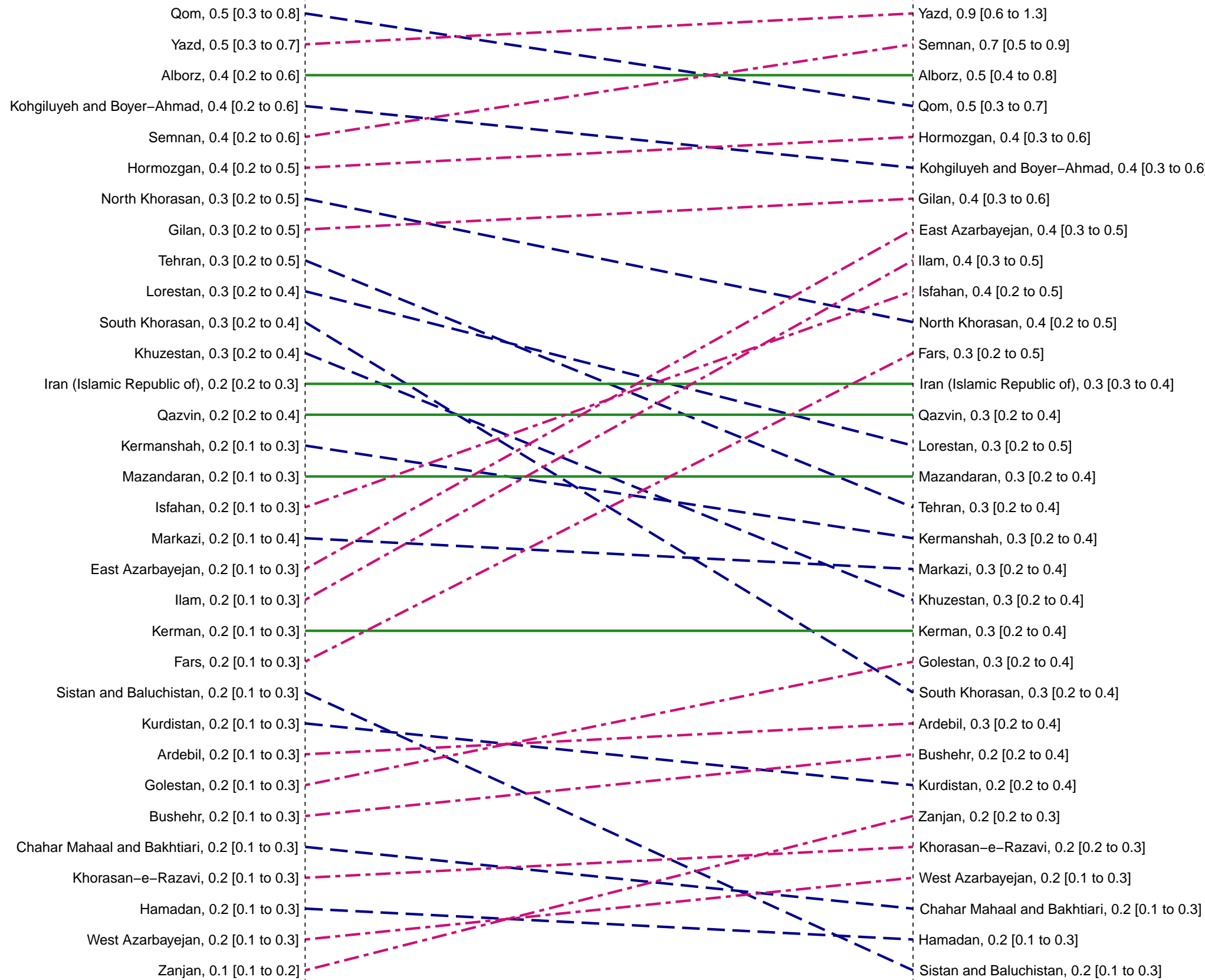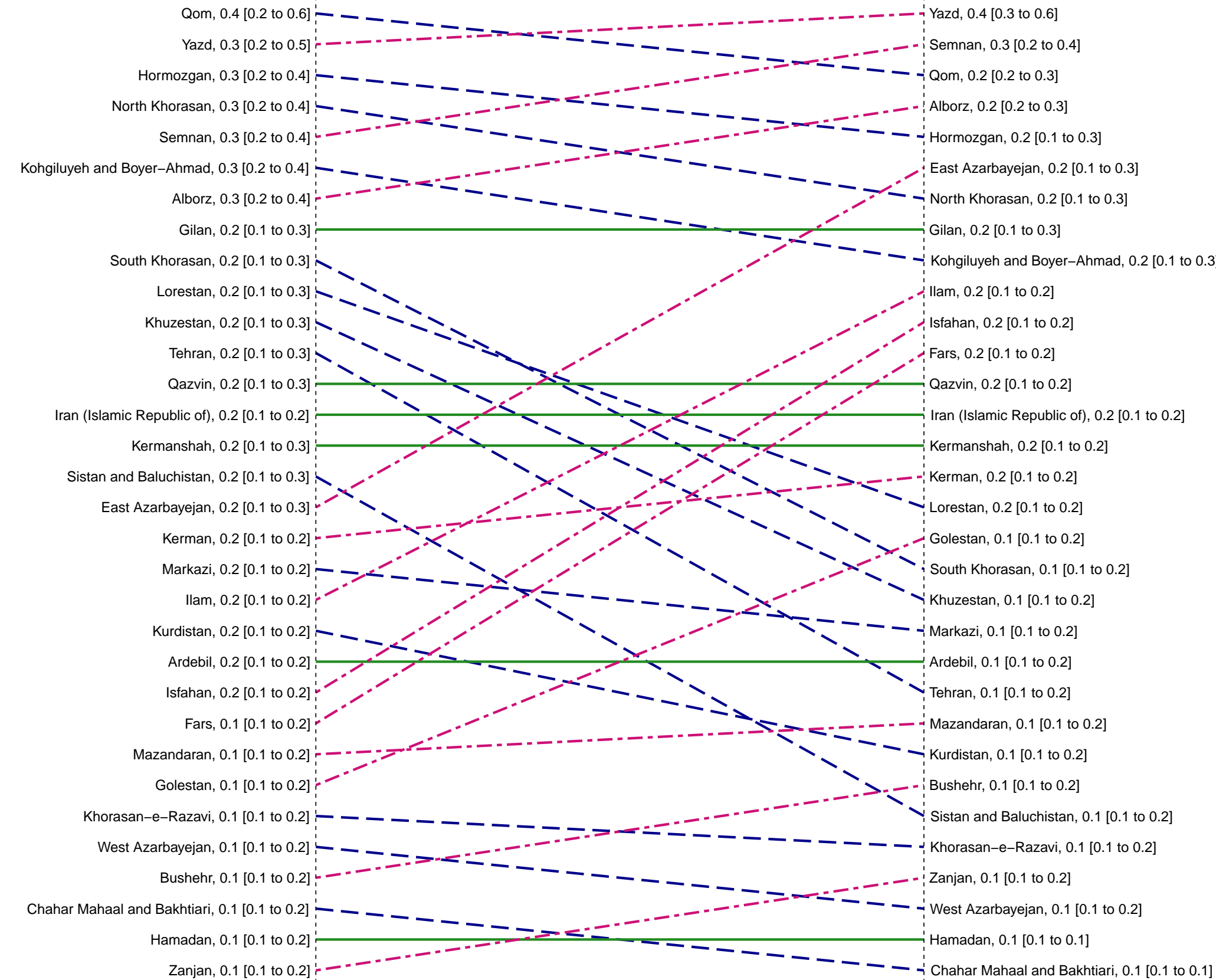

Ranking

Upward

Monotone

Downward

Supplement: Supplementary file 6 — Additional file 6. Fig. 2C Breast cancer age-standardized incidence and deaths rates (per 100,000 population) rankings in Iran and its 31 provinces in 1990 and 2019, for A) both sexes, B) females, and C) males. [file 13058_2023_1633_MOESM6_ESM.pdf]

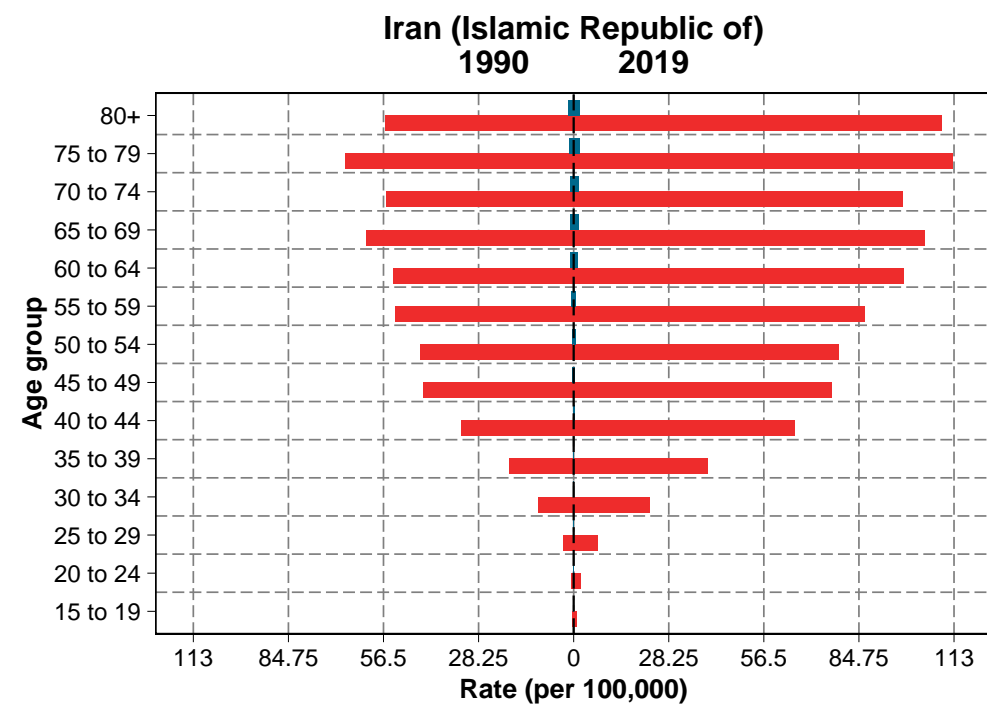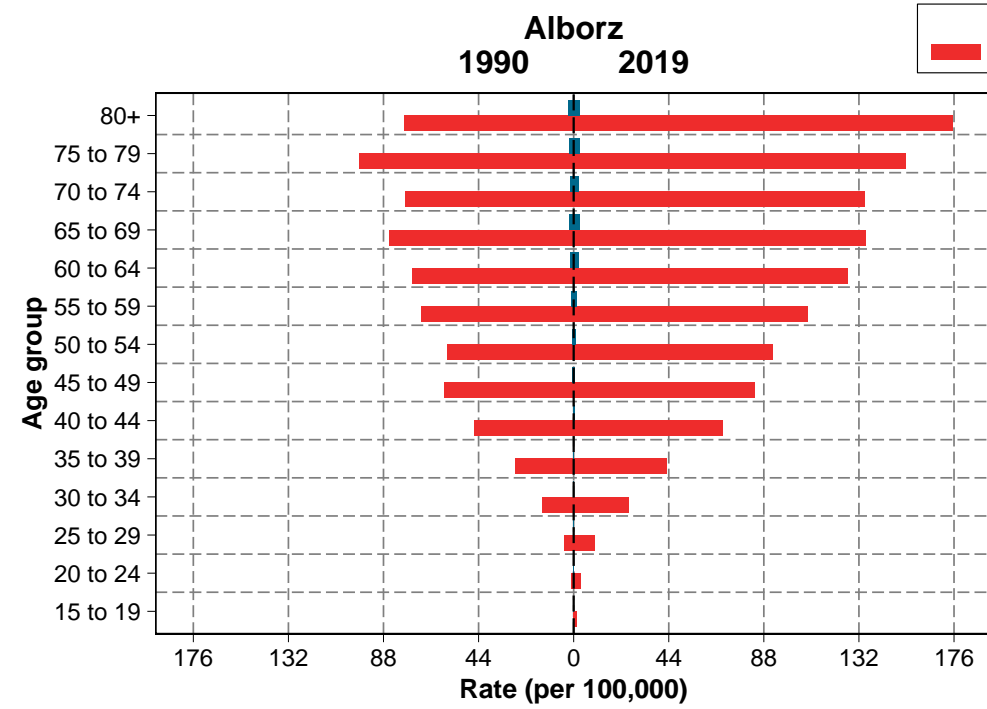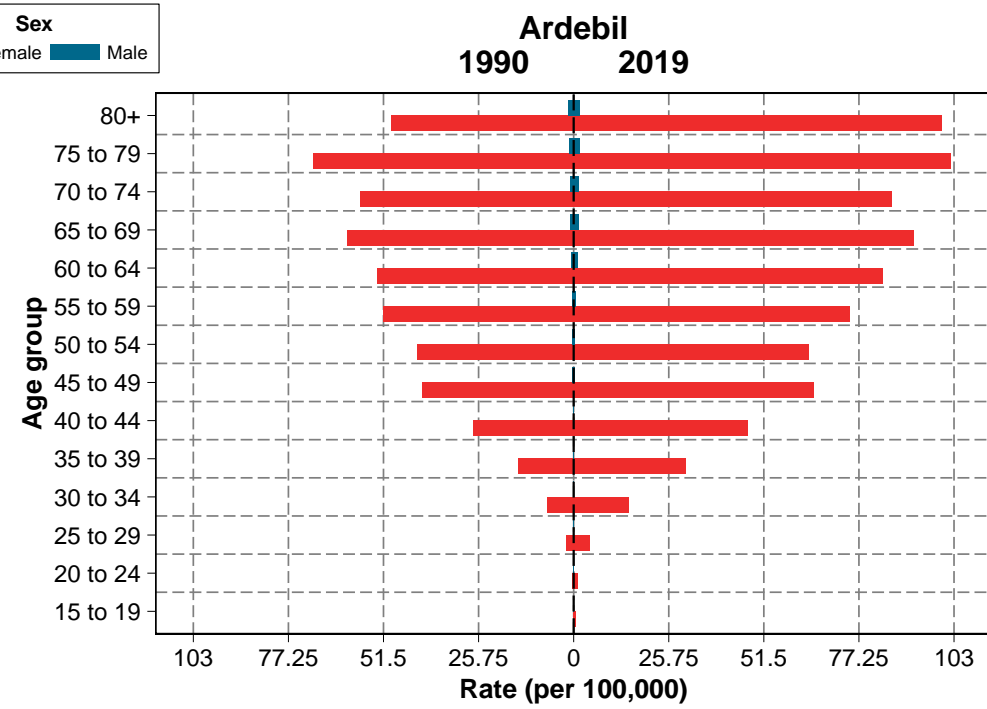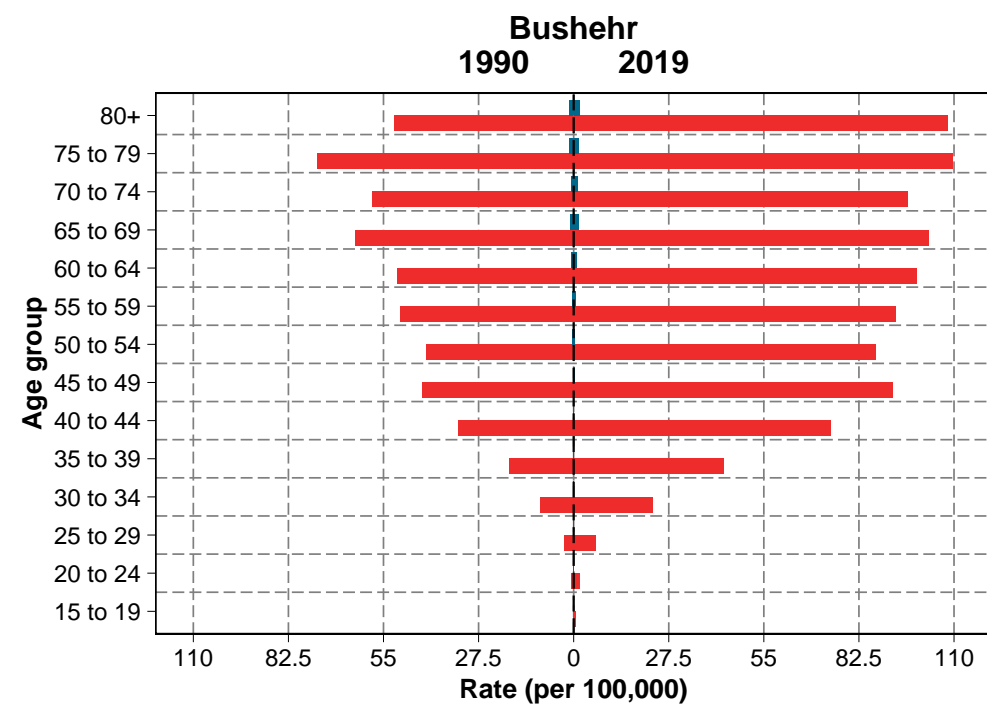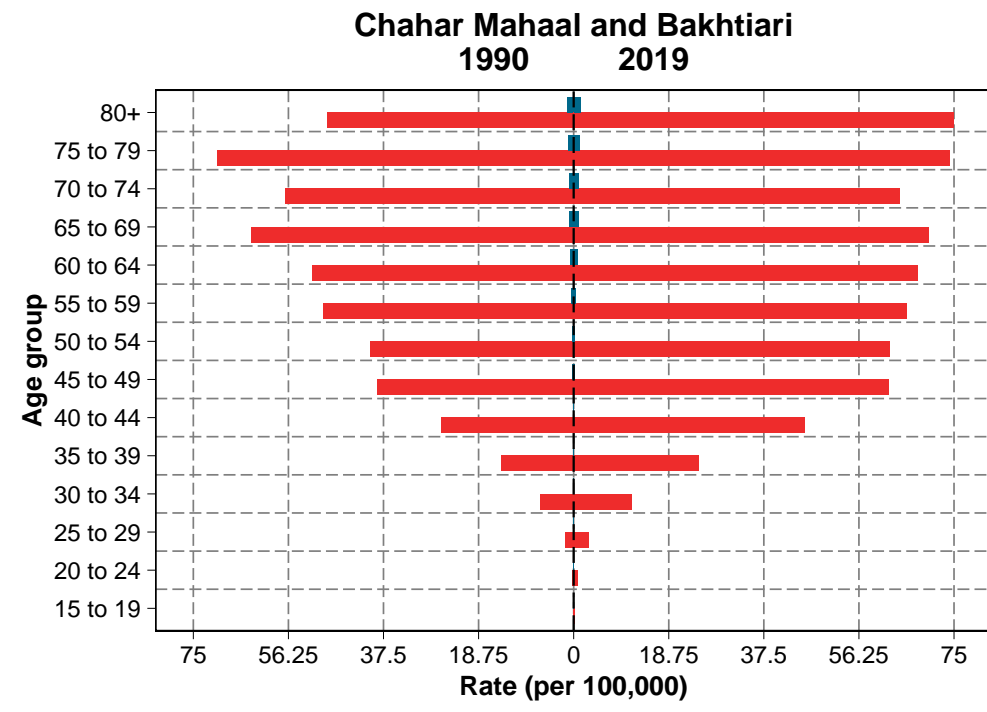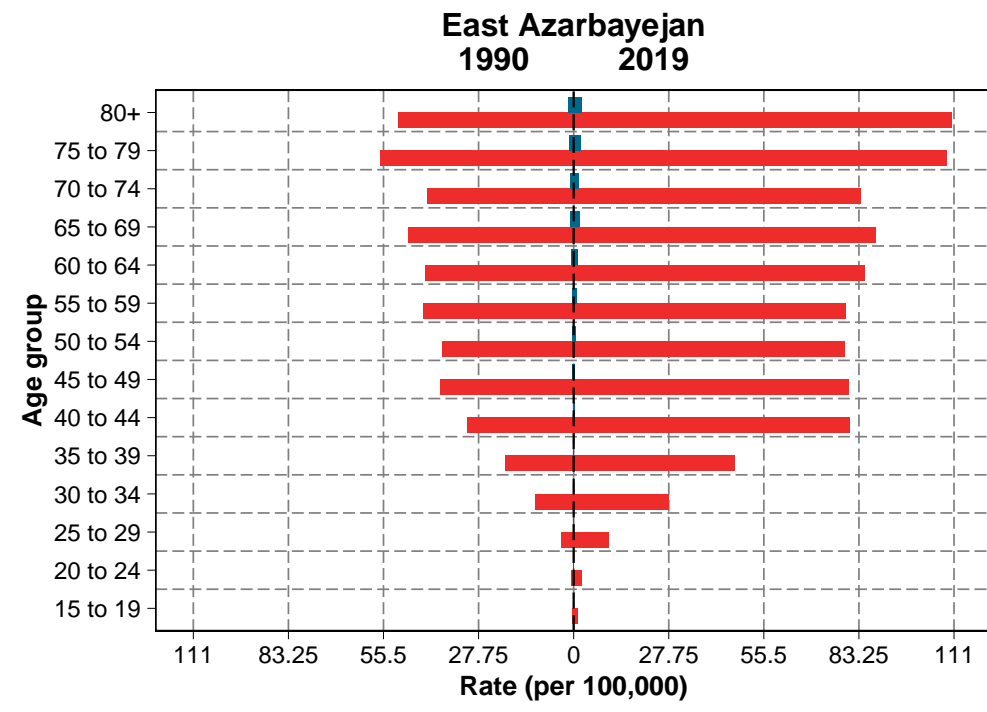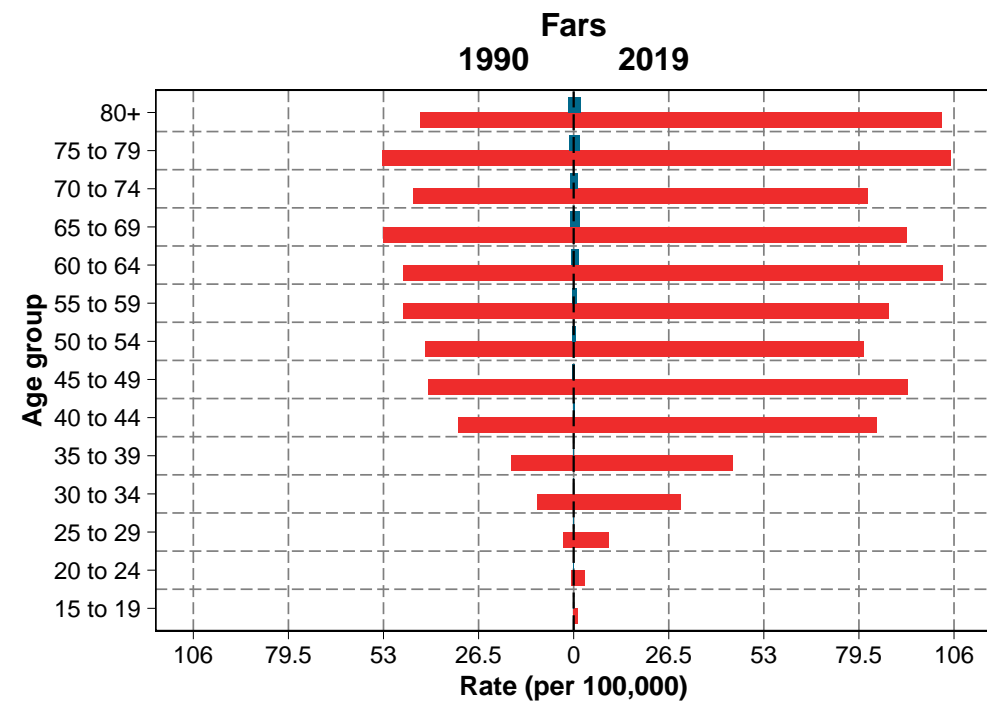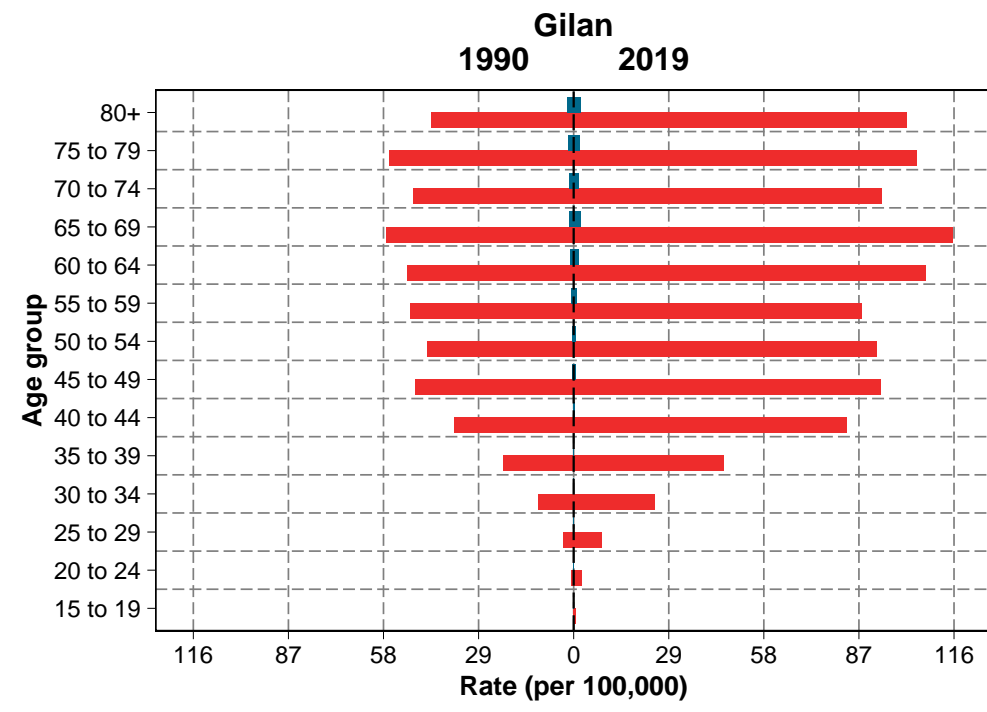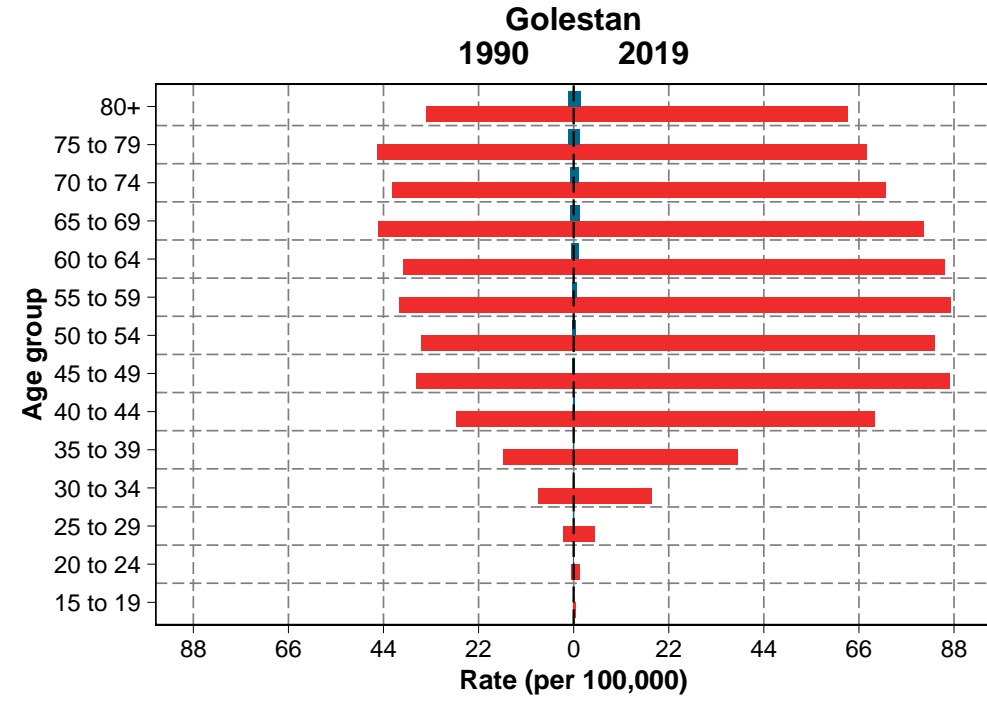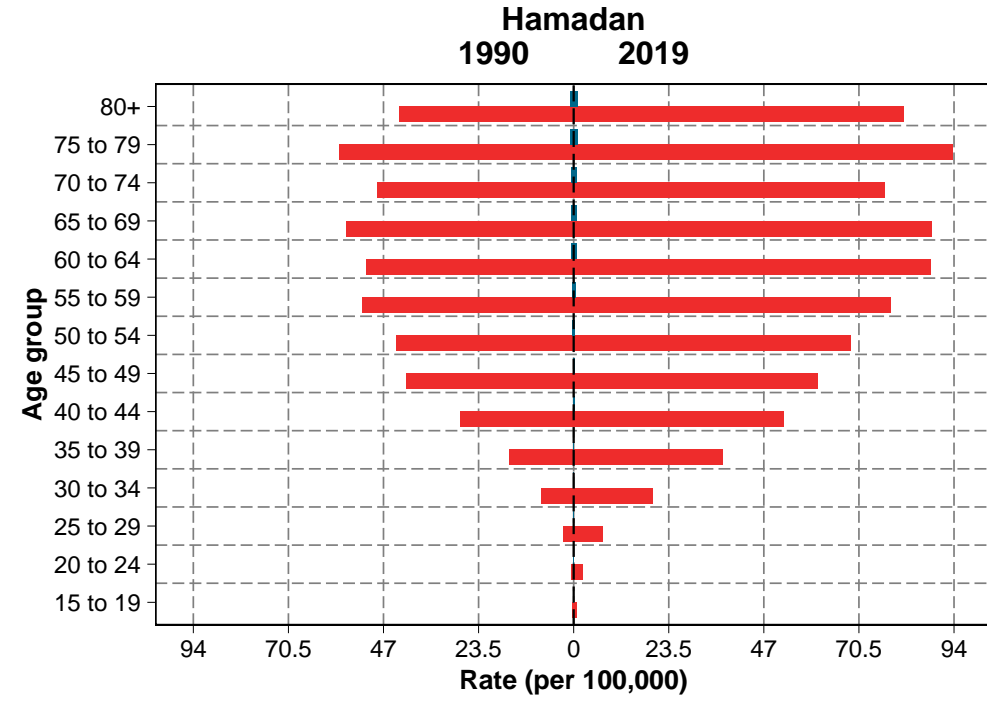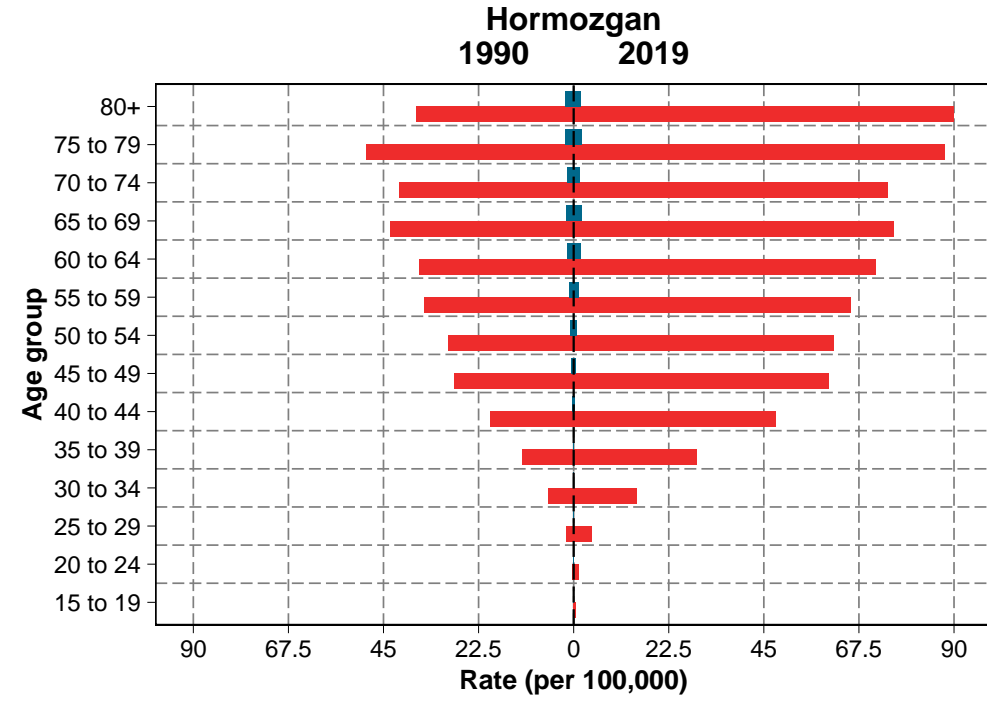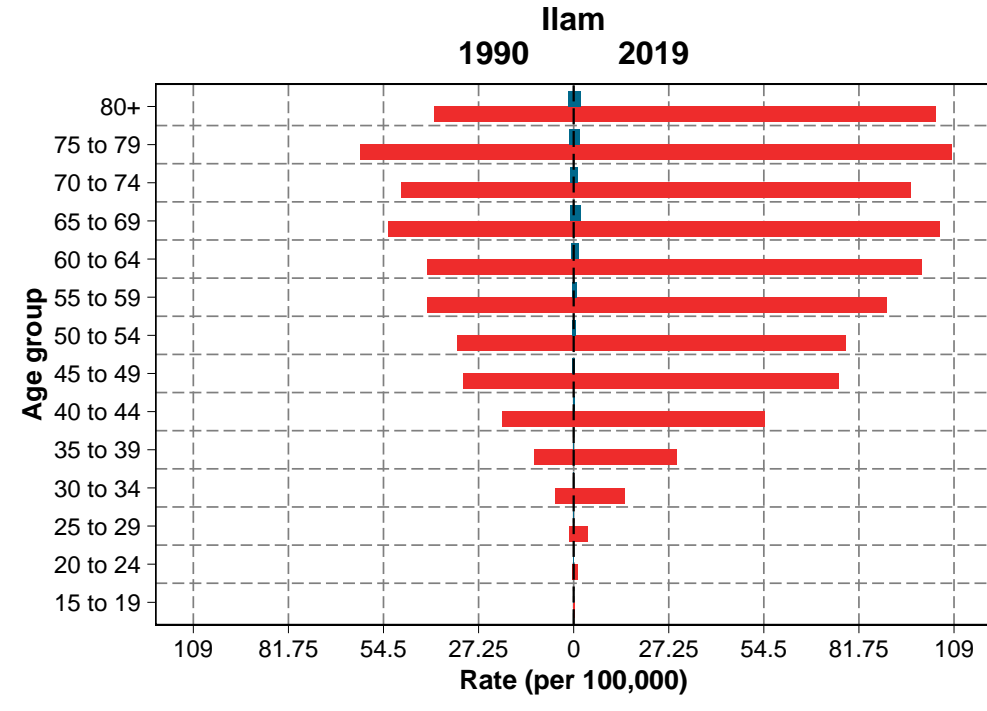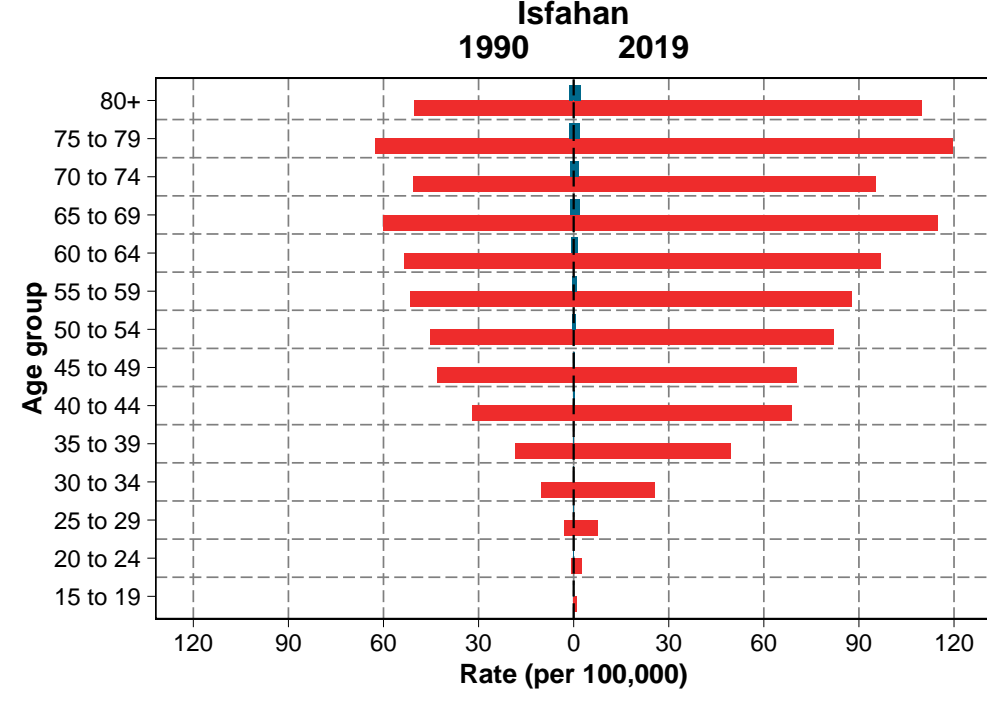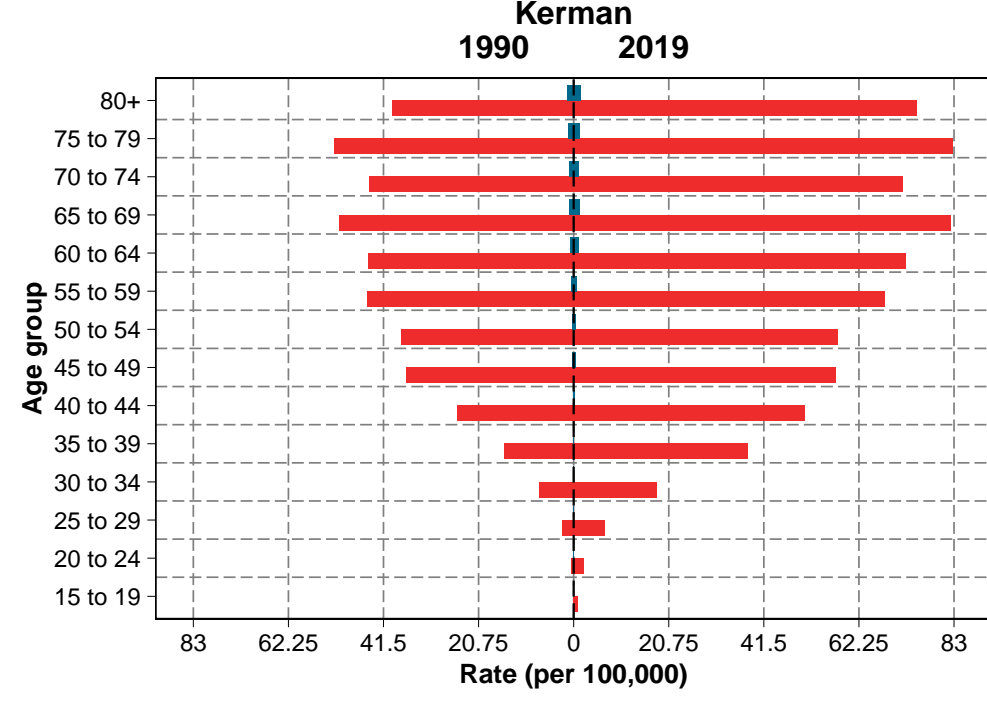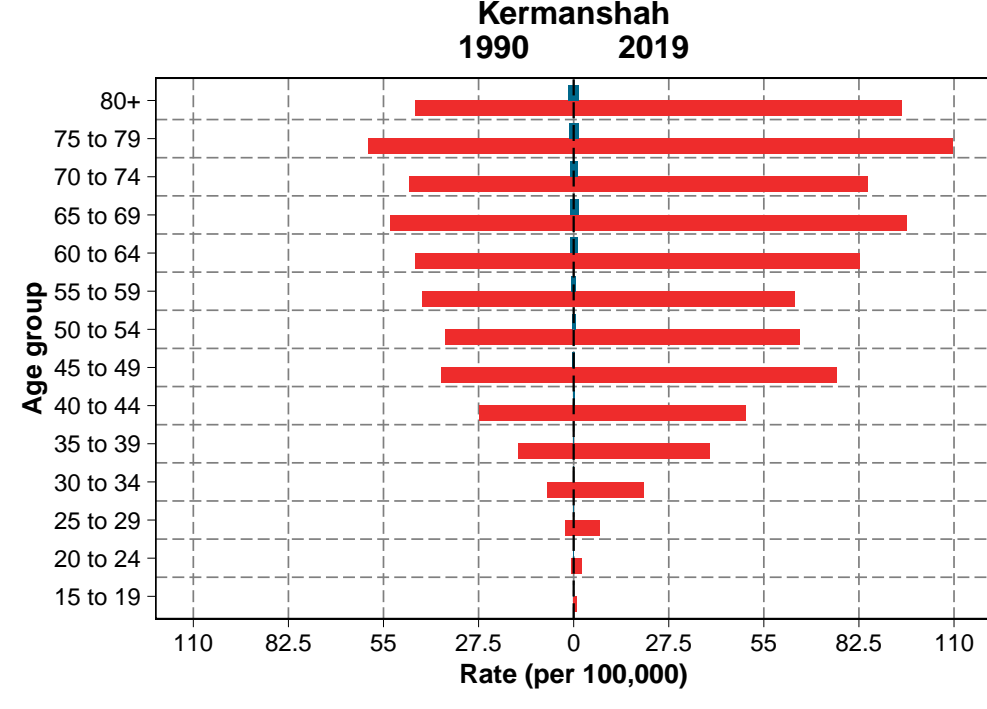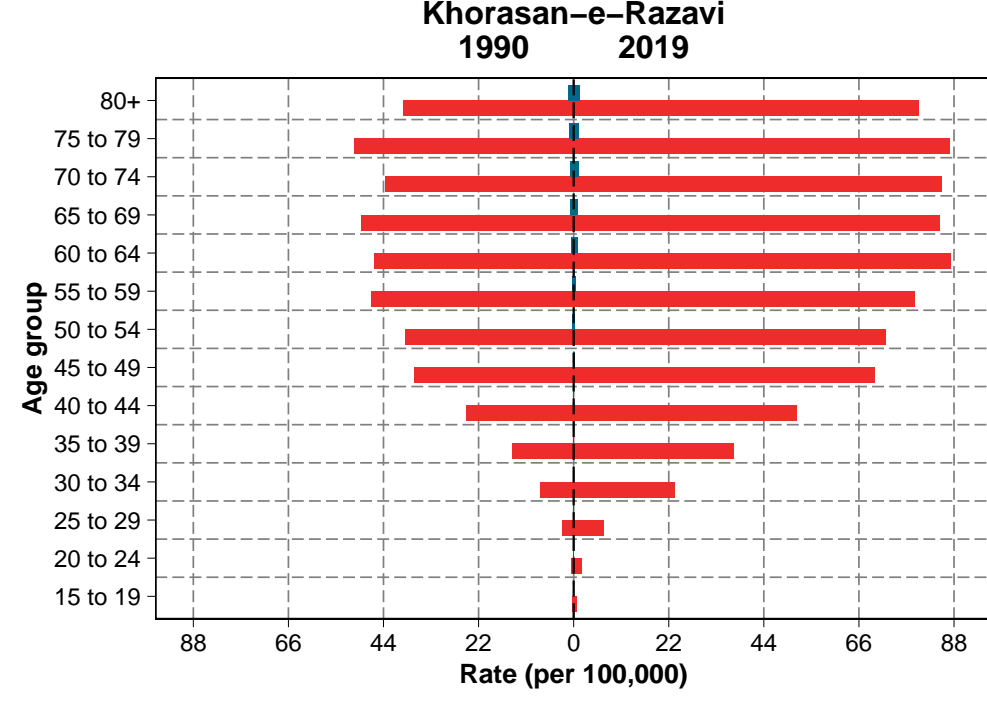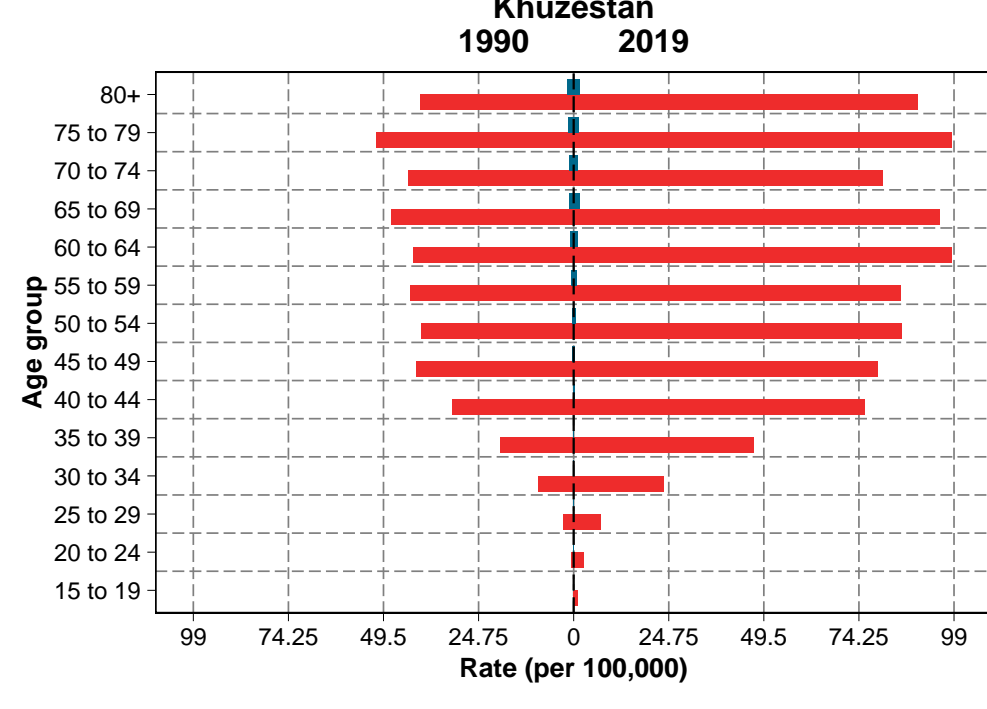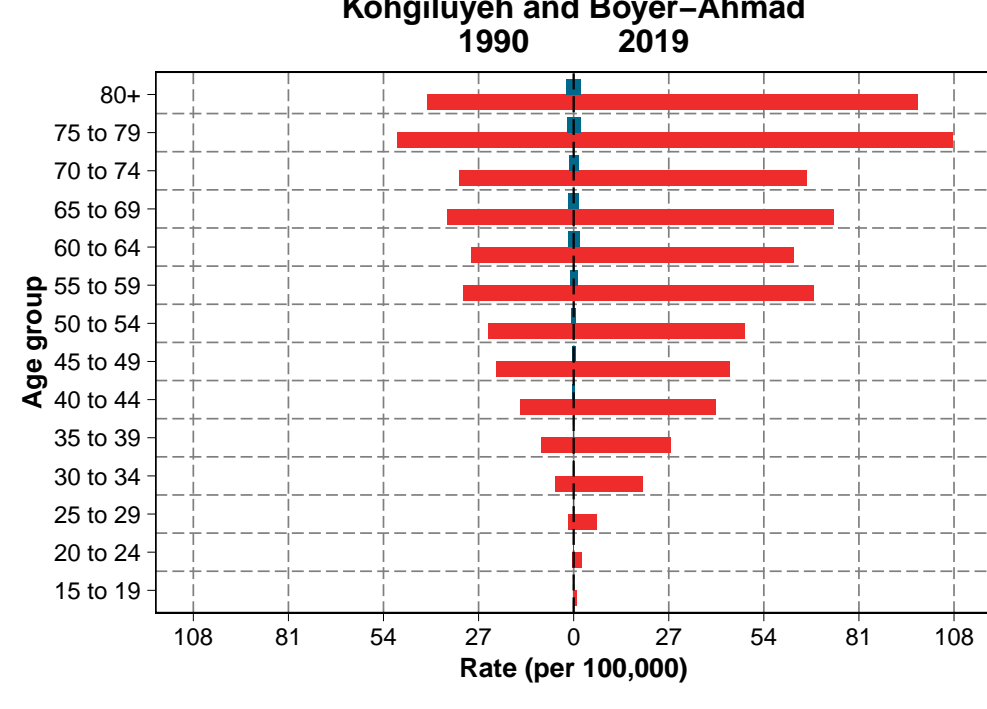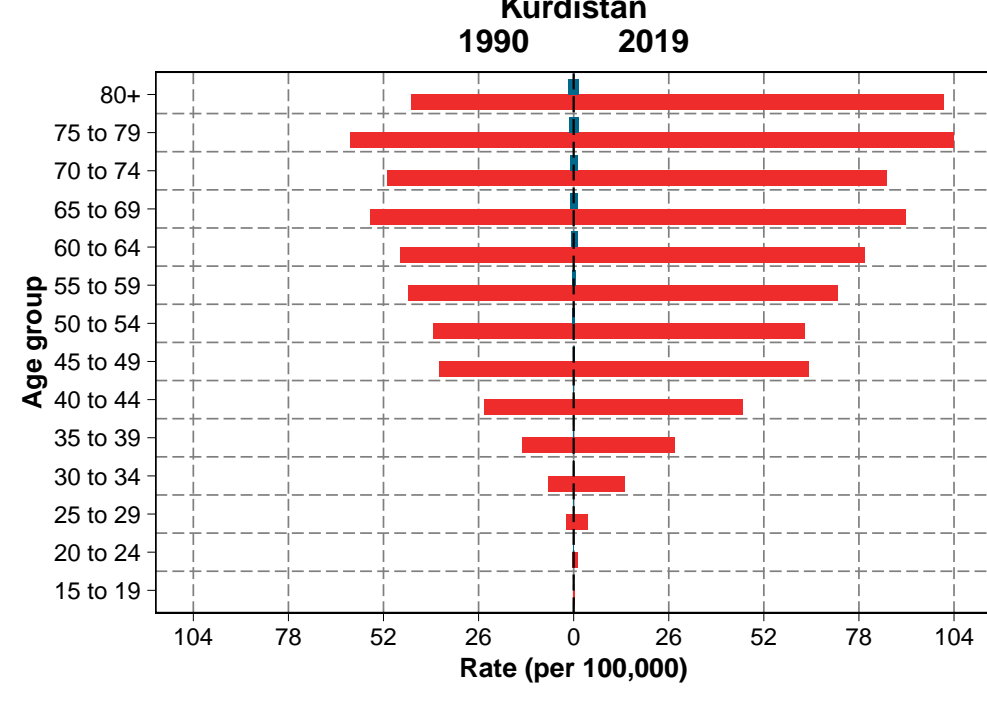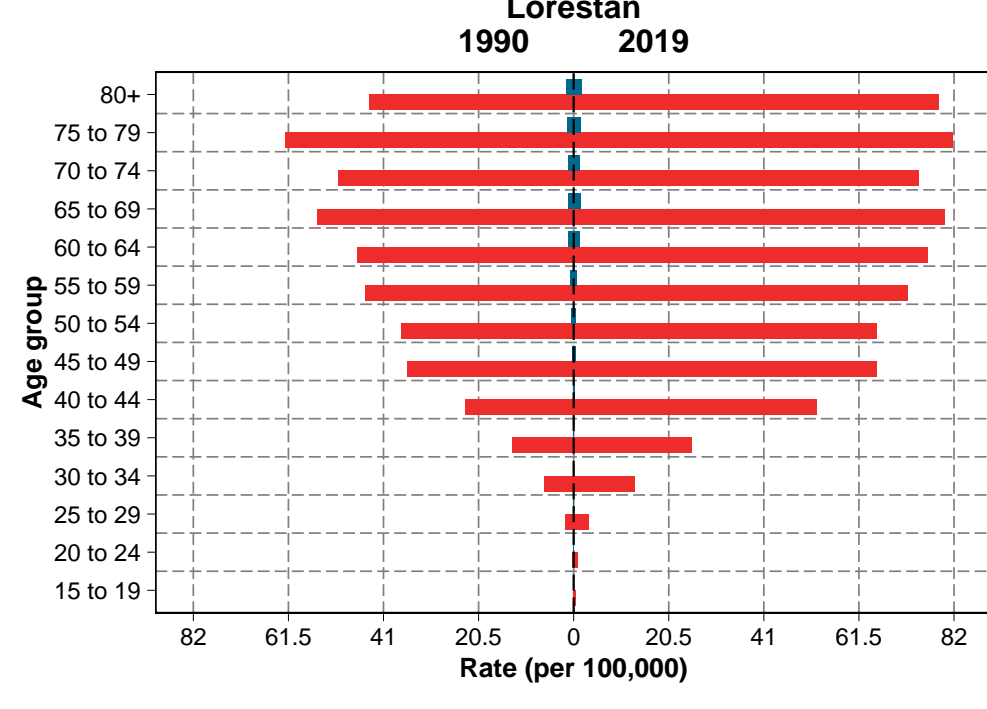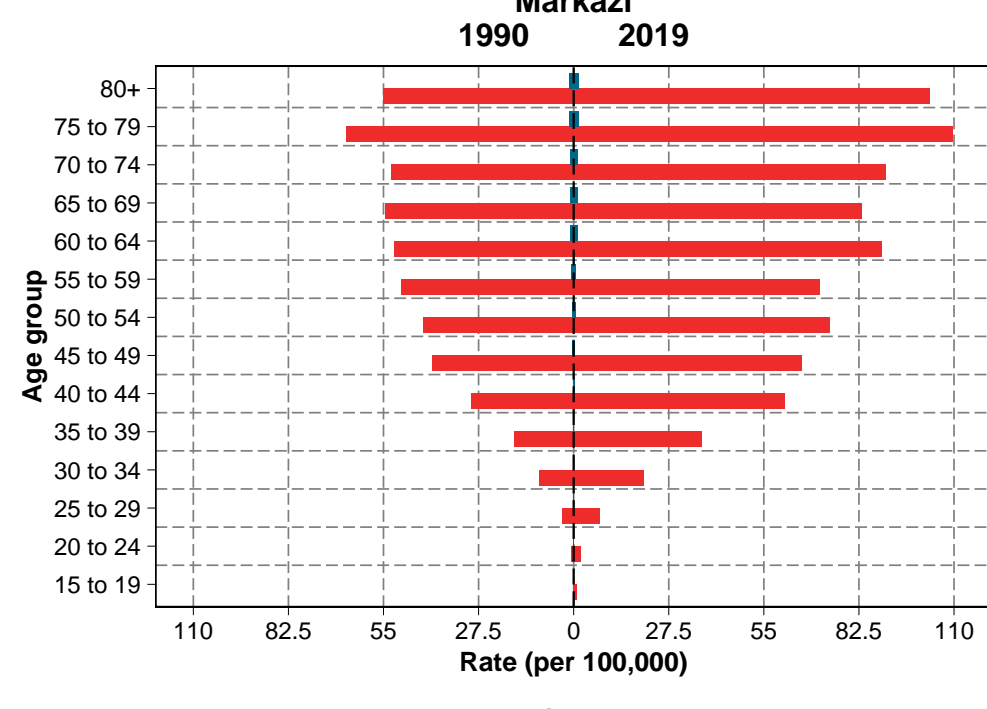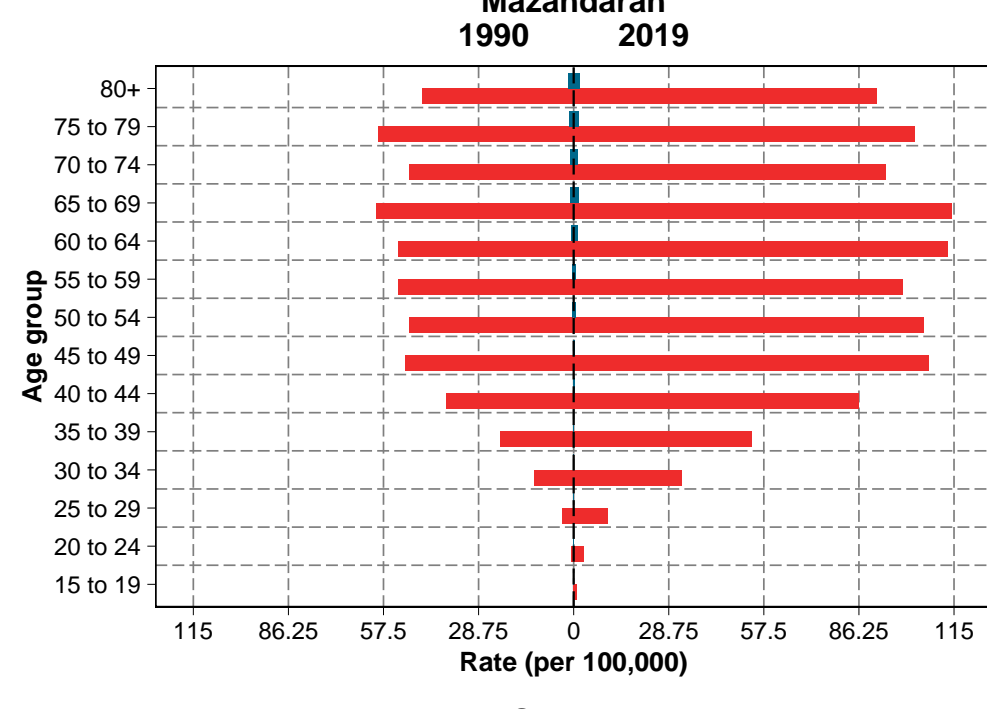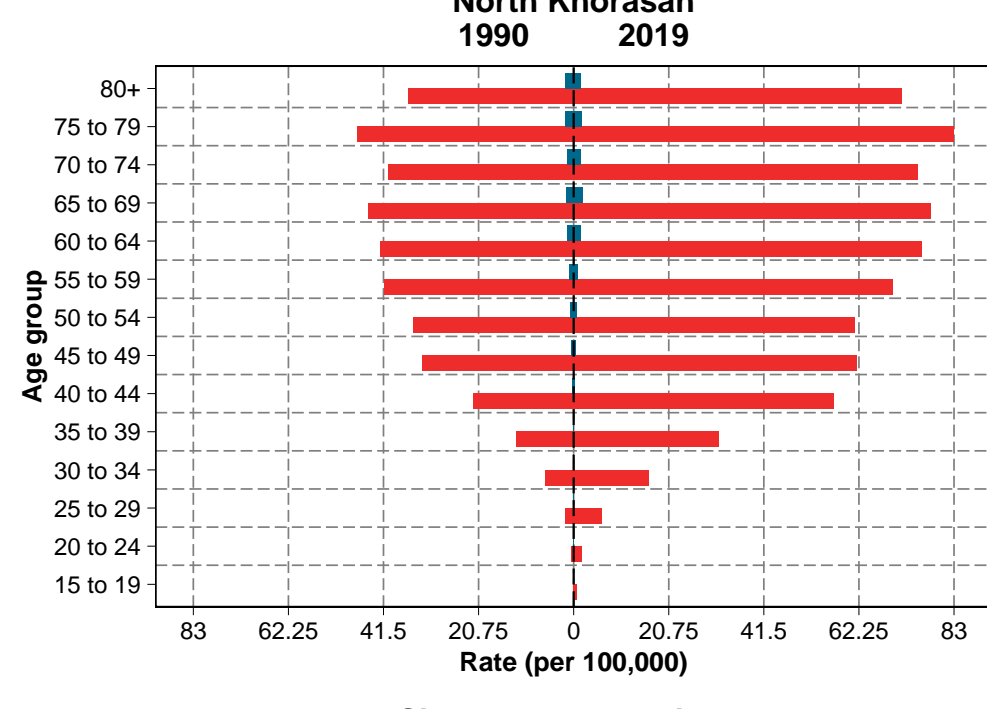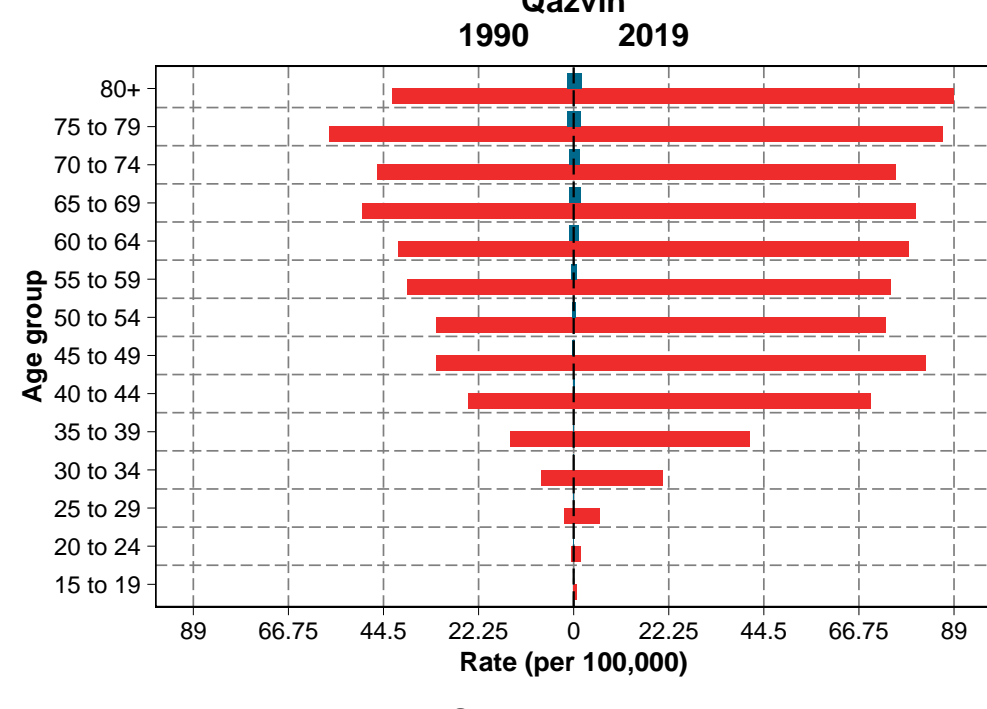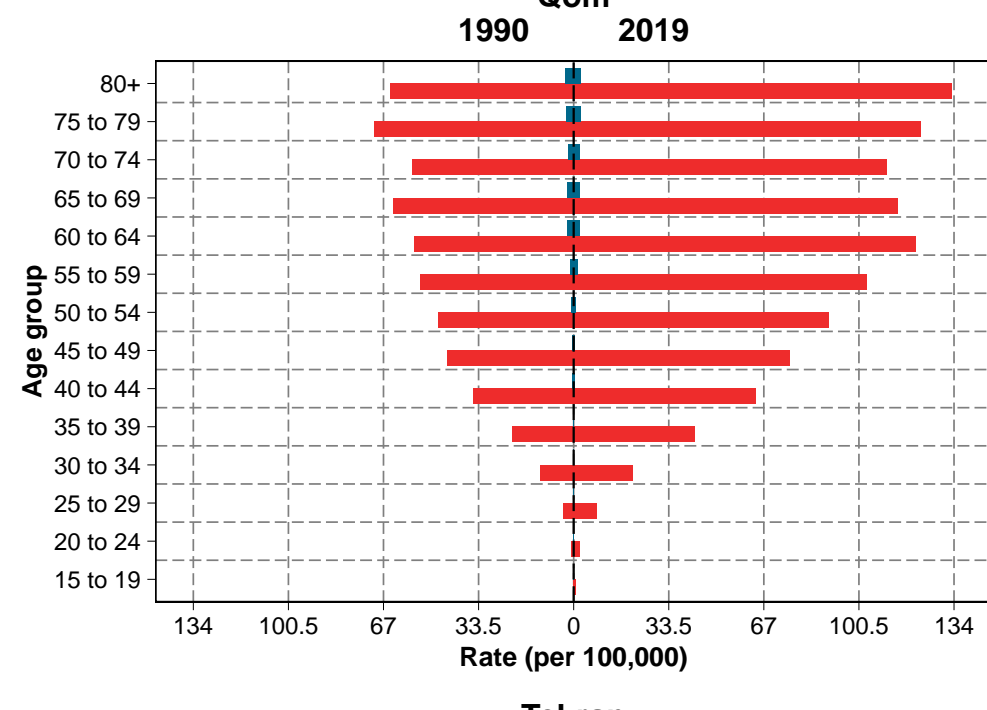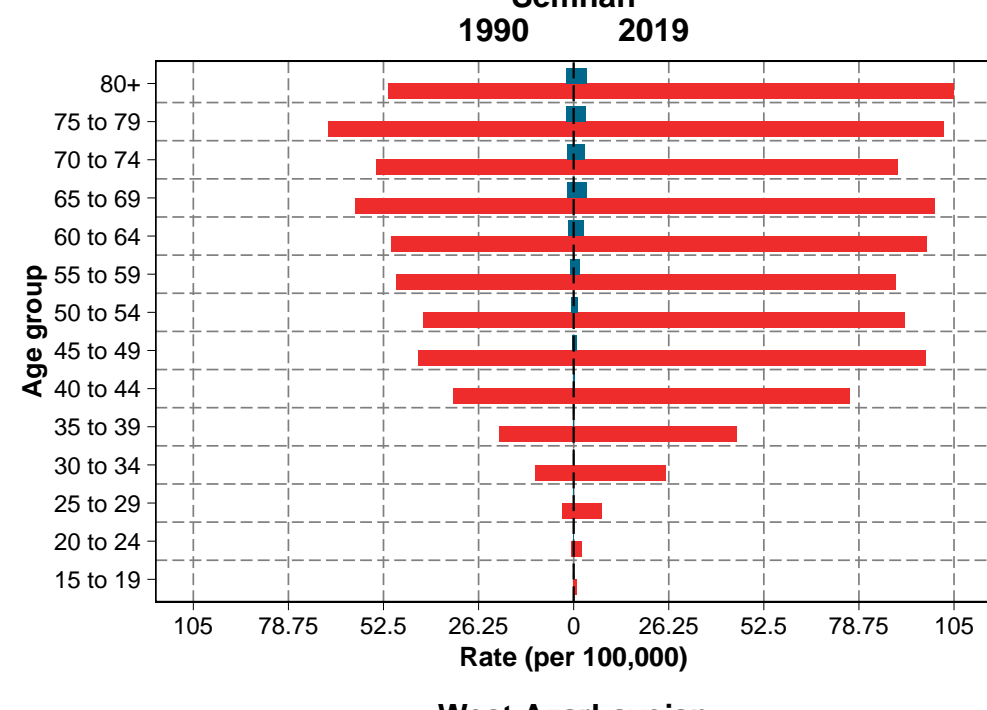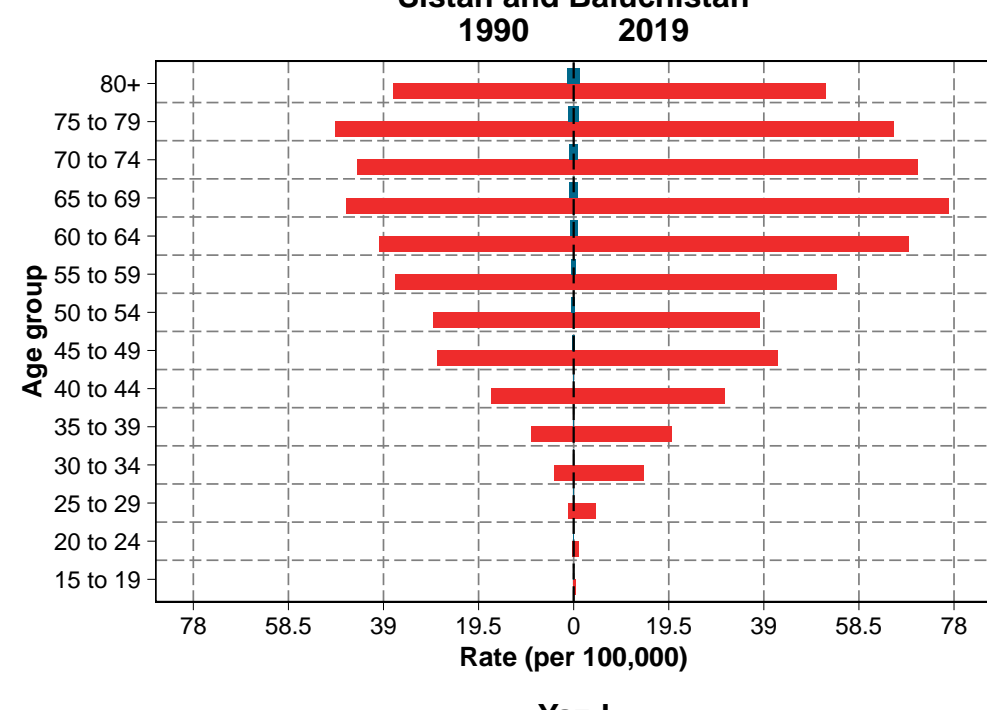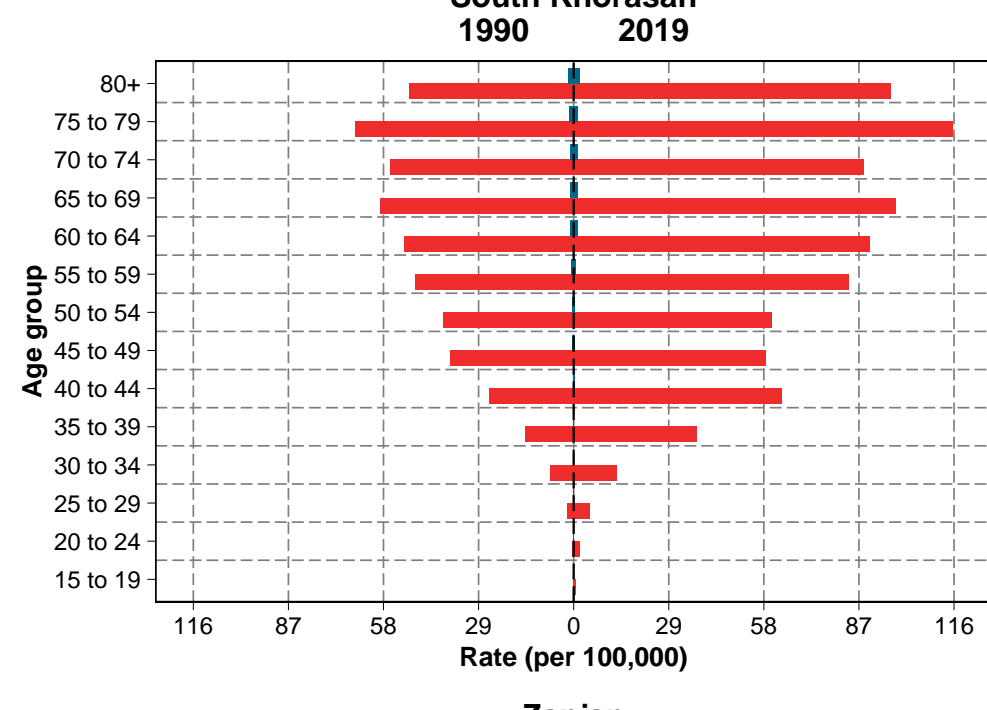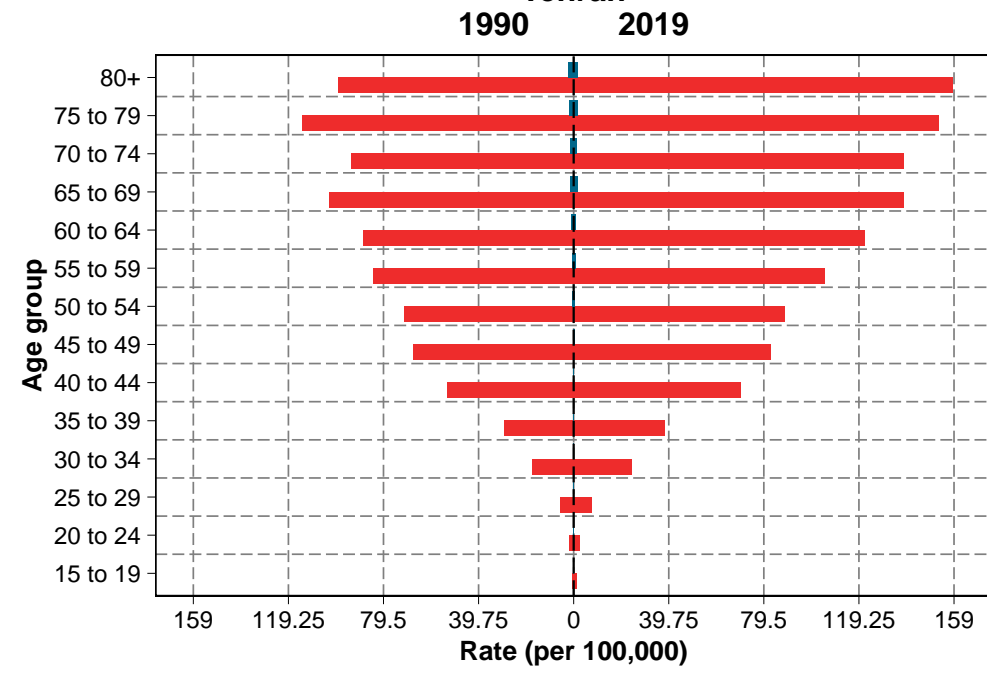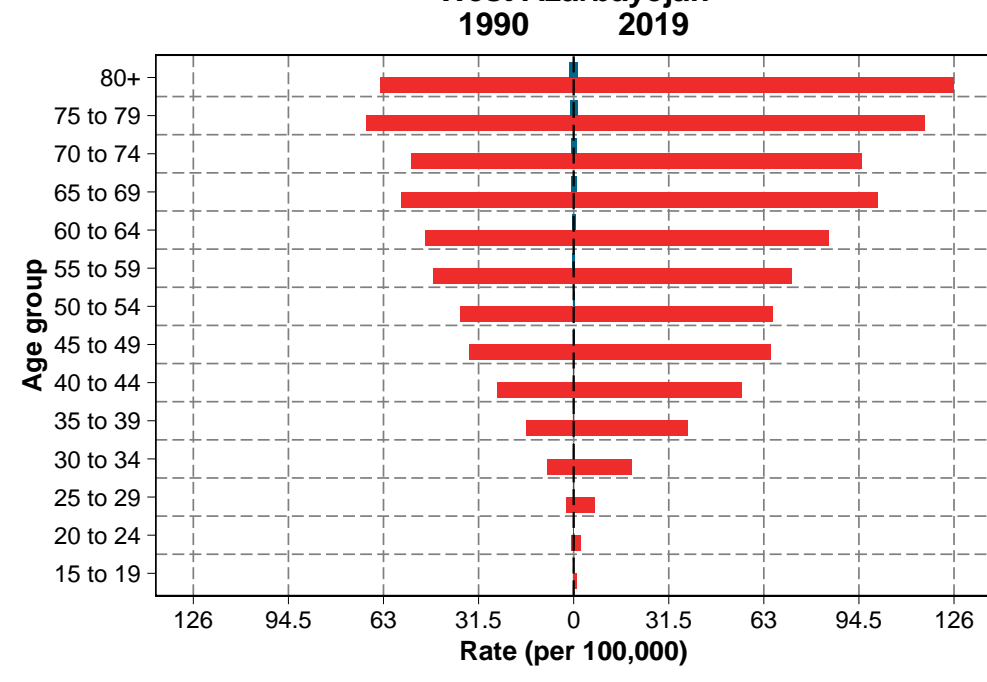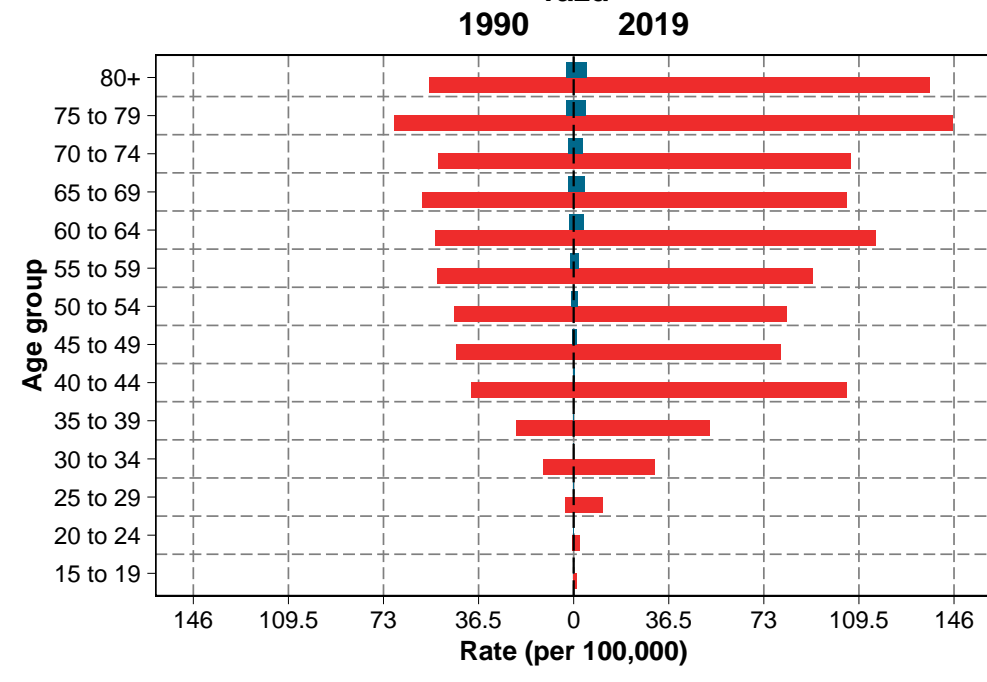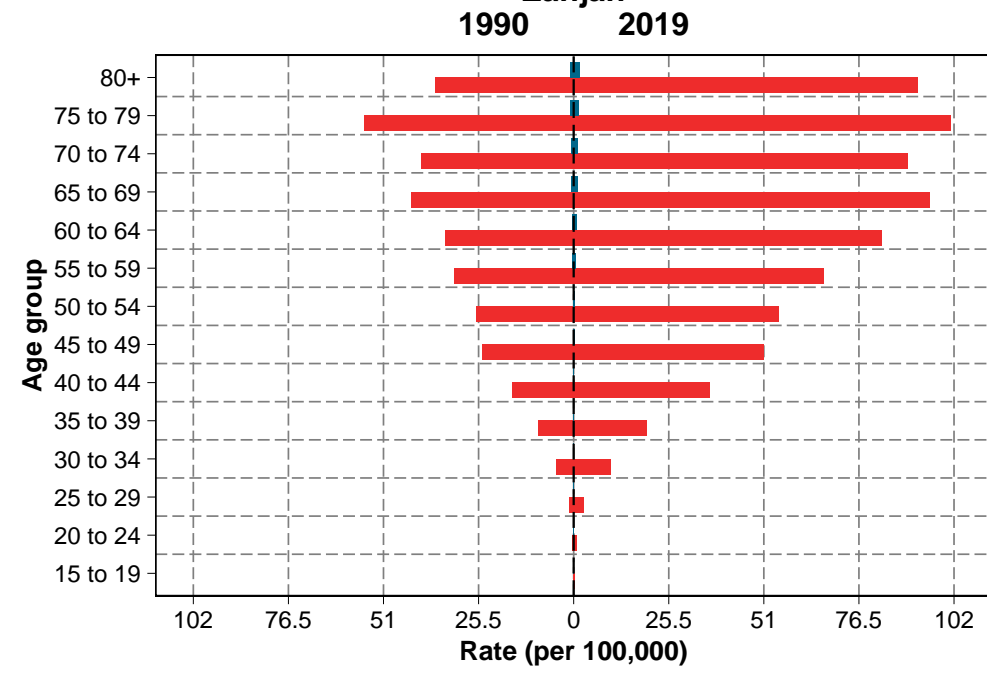

Supplement: Supplementary file 9 — Additional file 9. Fig. 3 Breast cancer incidence rates (per 100,000 population) in 1990 and 2019 based on age groups by sex (red: female; blue: males) in Iran and its 31 provinces. [file 13058_2023_1633_MOESM9_ESM.pdf]

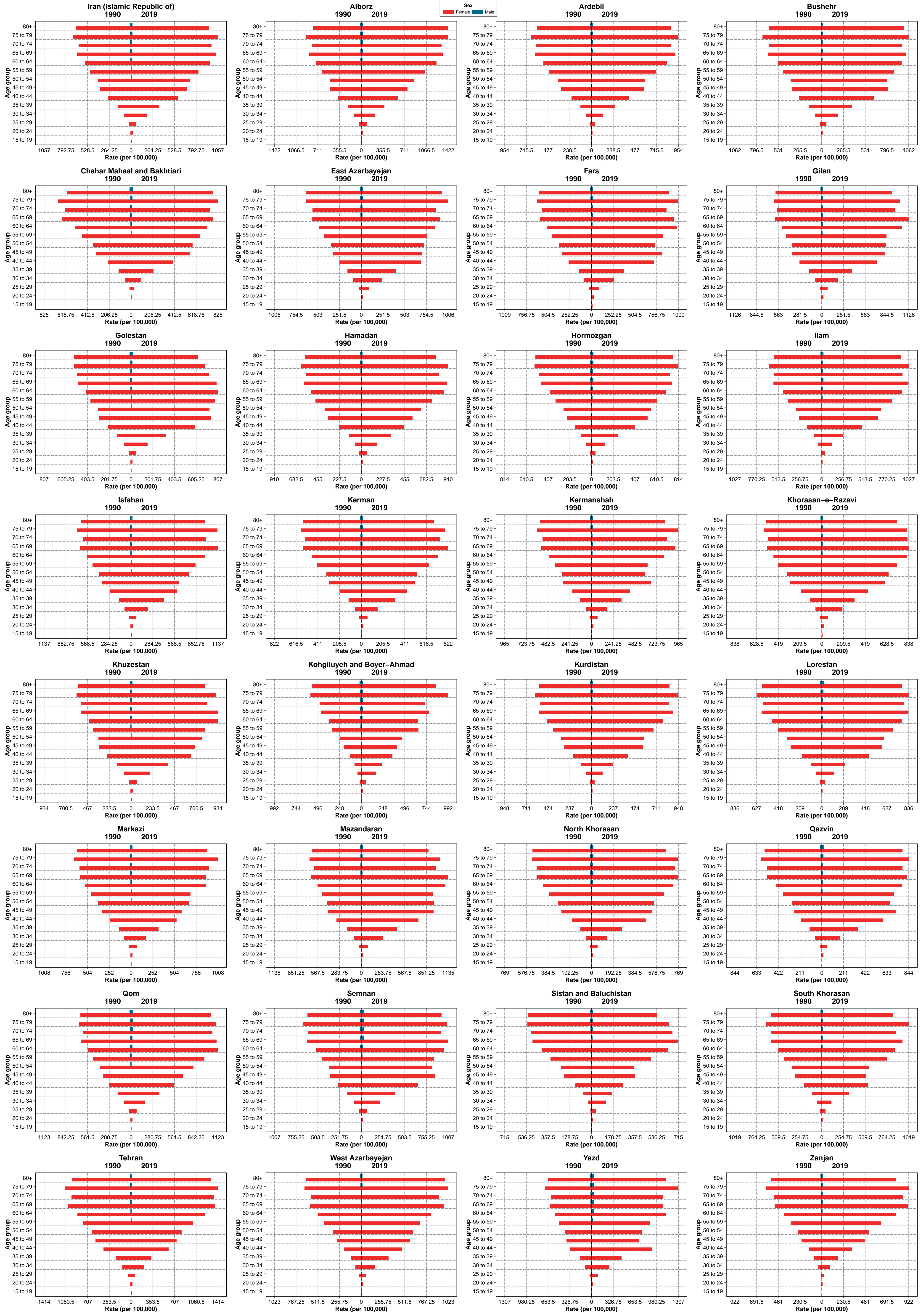

Supplement: Supplementary file 10 — Additional file 10. Fig. 4 Breast cancer prevalence rates (per 100,000 population) in 1990 and 2019 based on age groups by sex (red: female; blue: males) in Iran and its 31 provinces. [file 13058_2023_1633_MOESM10_ESM.pdf]

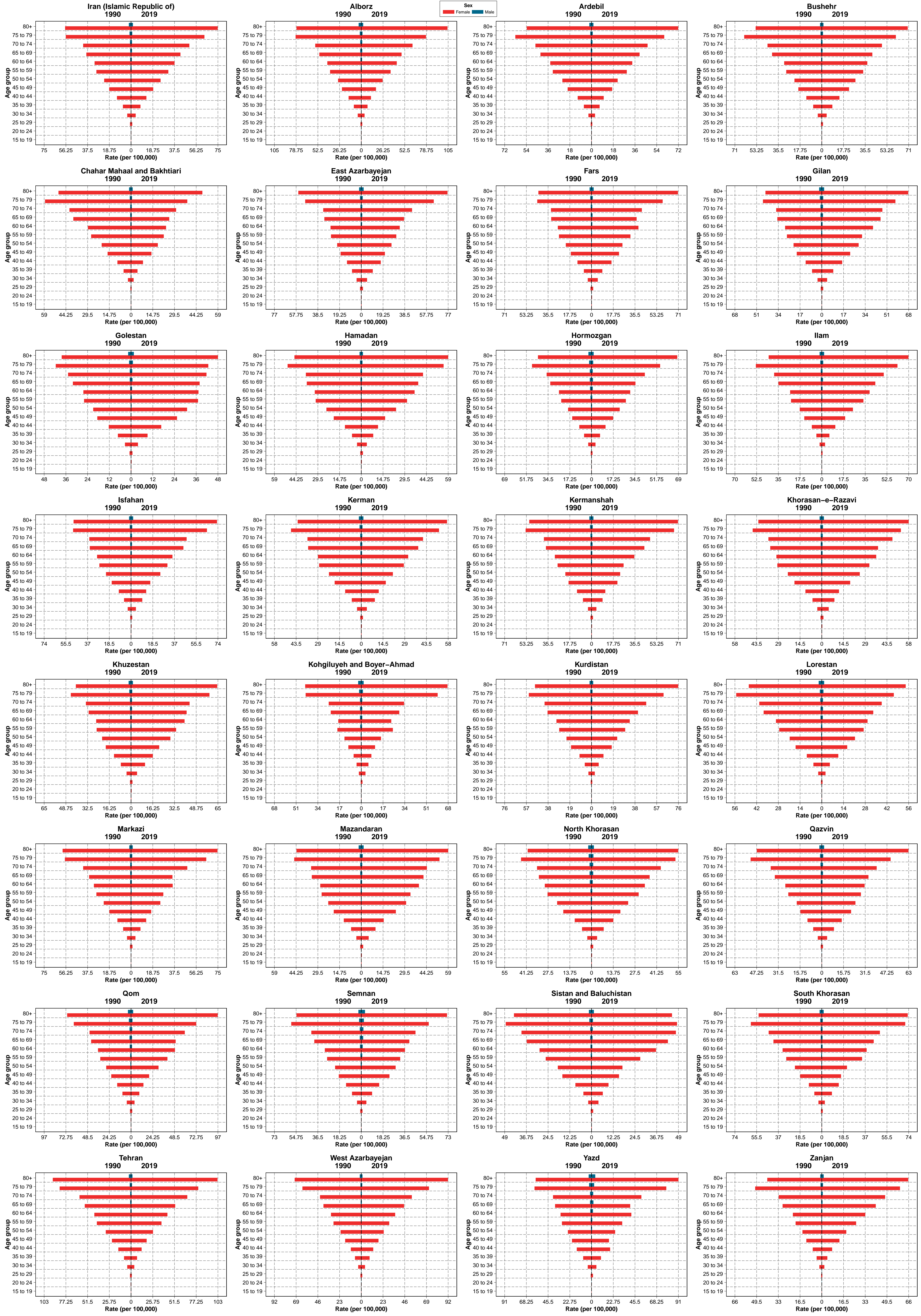

Supplement: Supplementary file 11 — Additional file 11. Fig. 5 Breast cancer deaths rates (per 100,000 population) in 1990 and 2019 based on age groups by sex (red: female; blue: males) in Iran and its 31 provinces. [file 13058_2023_1633_MOESM11_ESM.pdf]

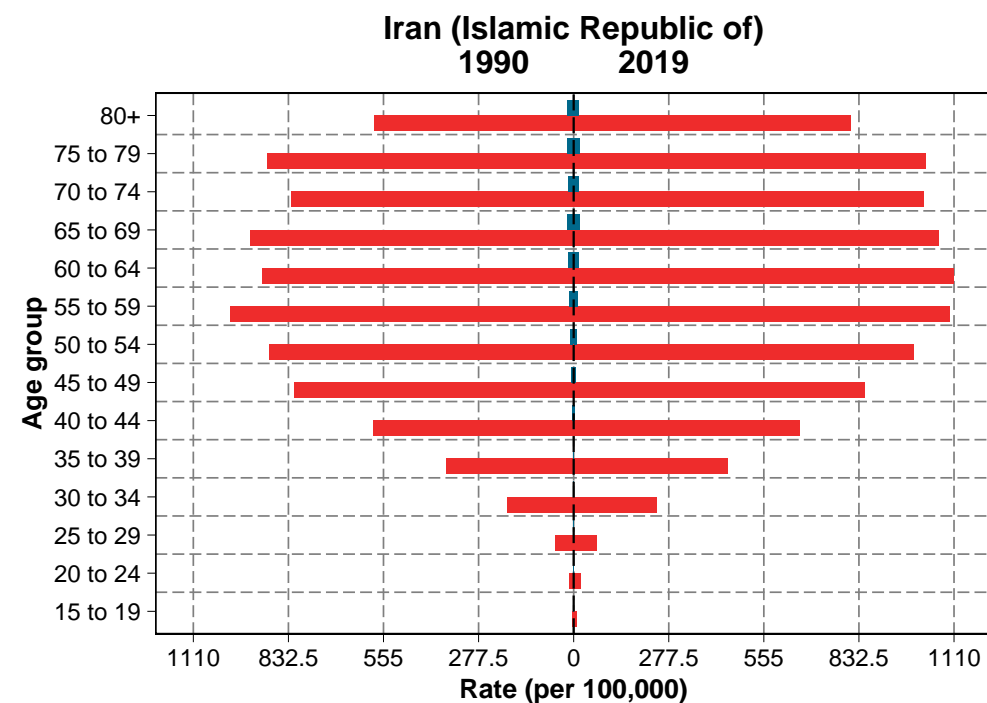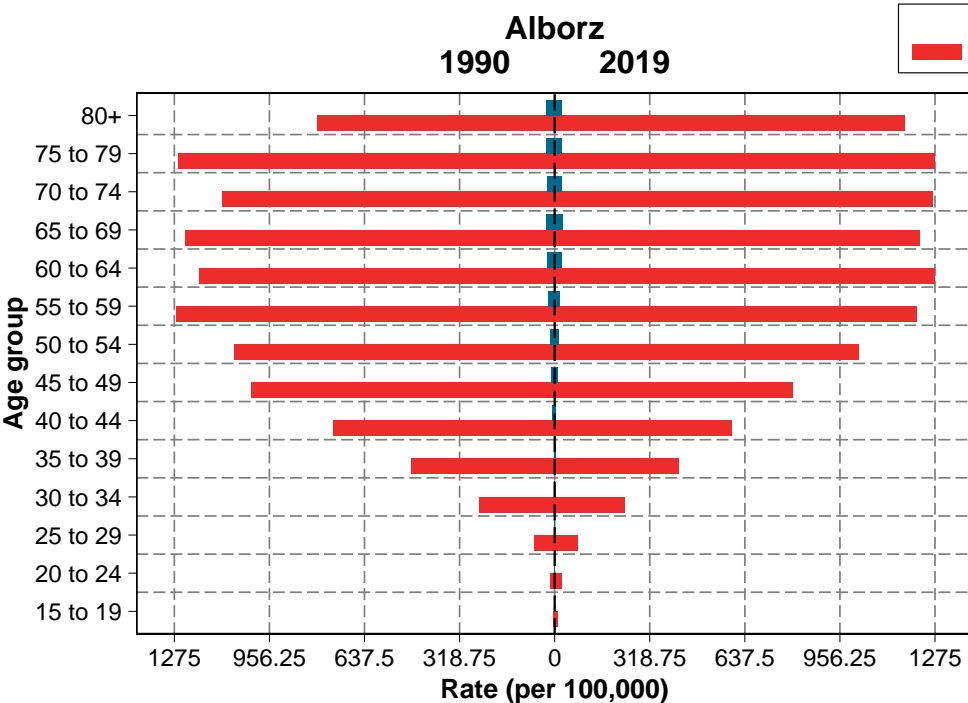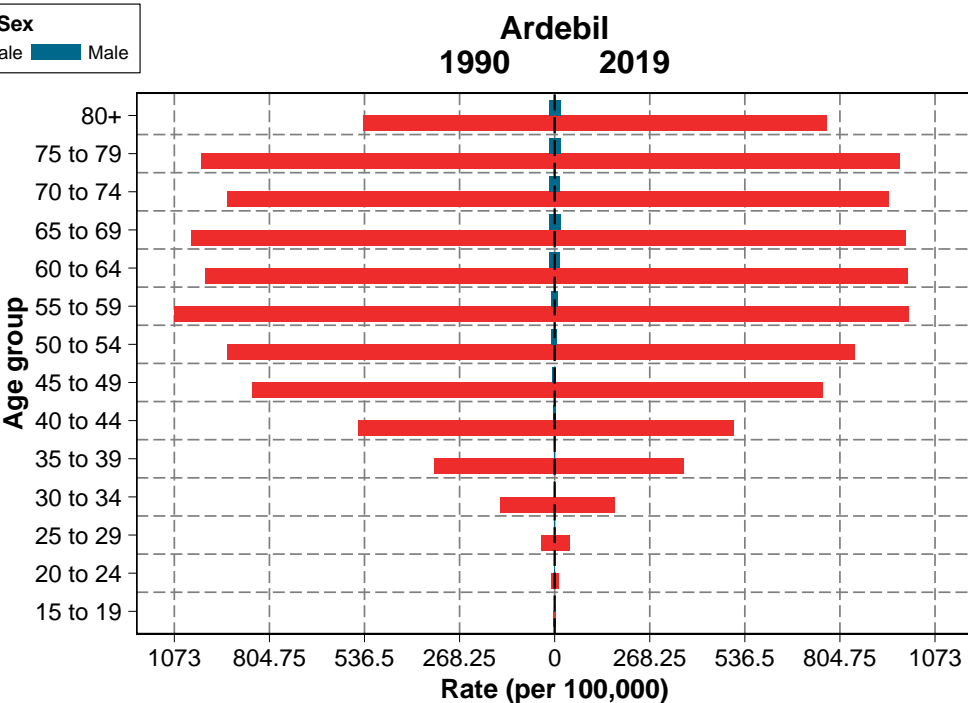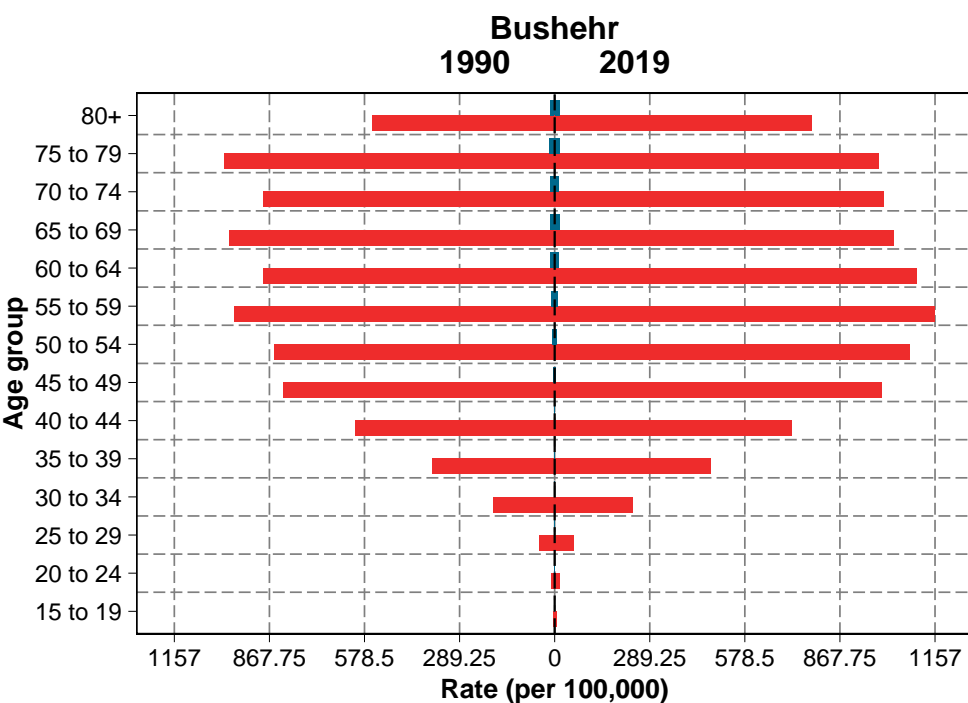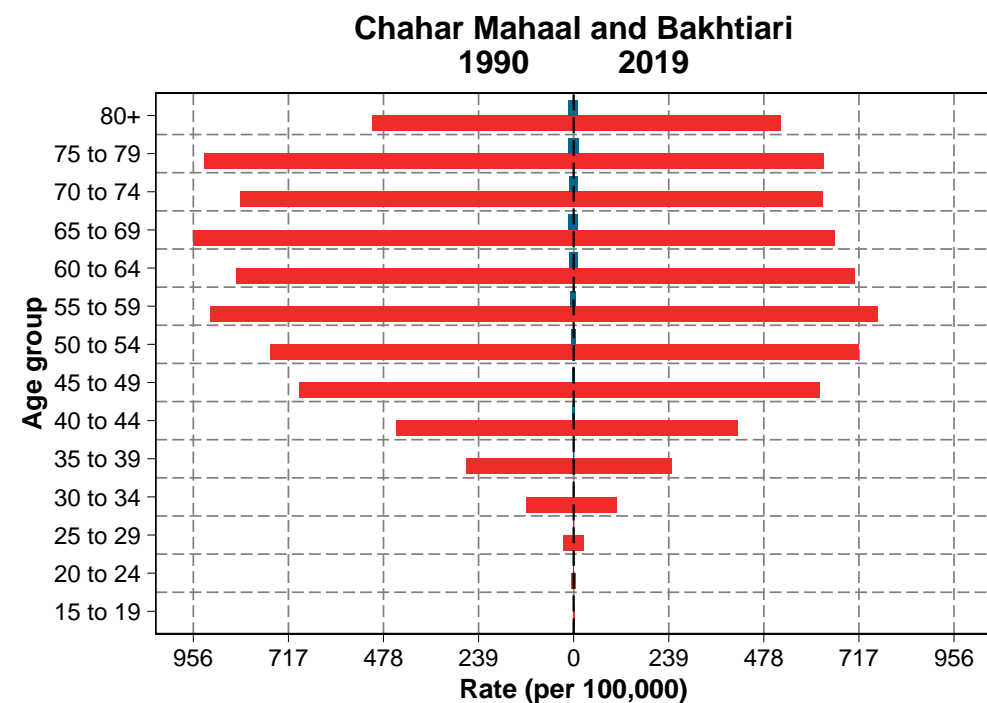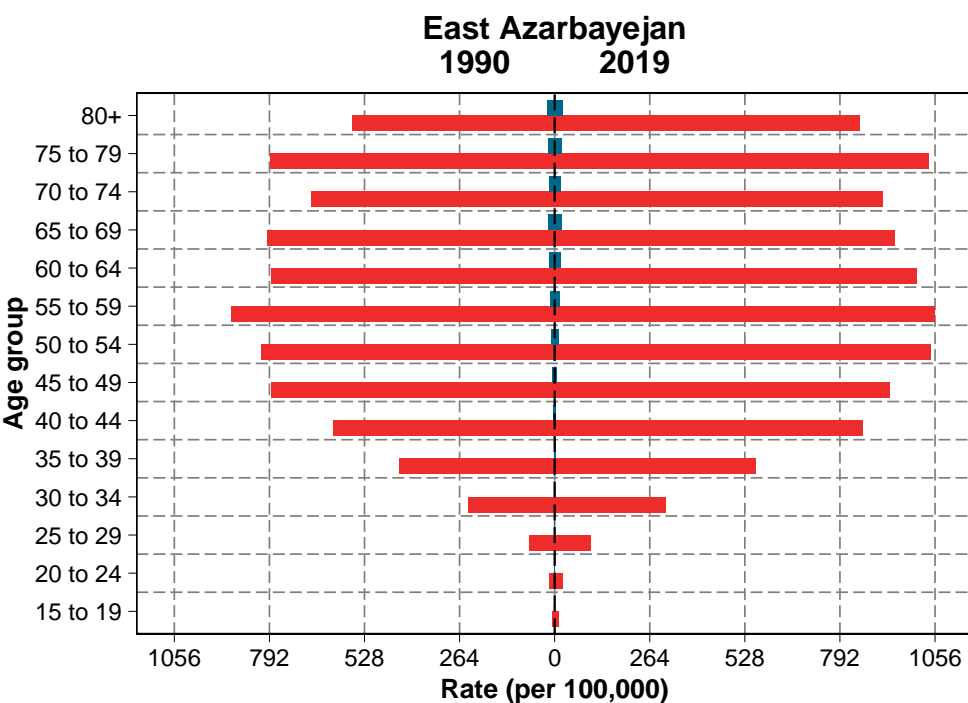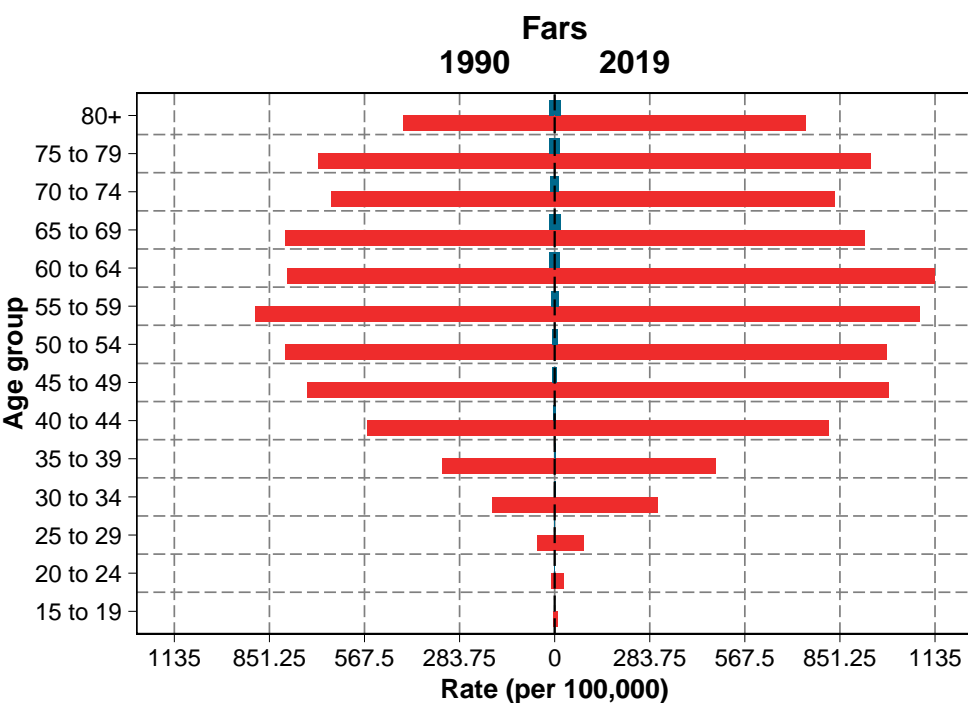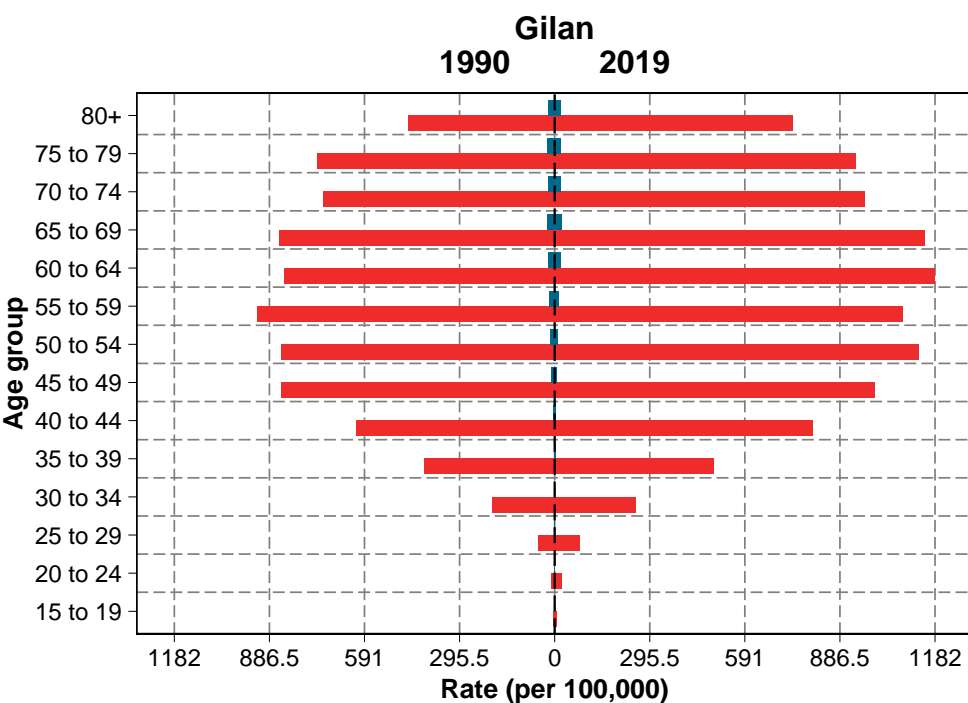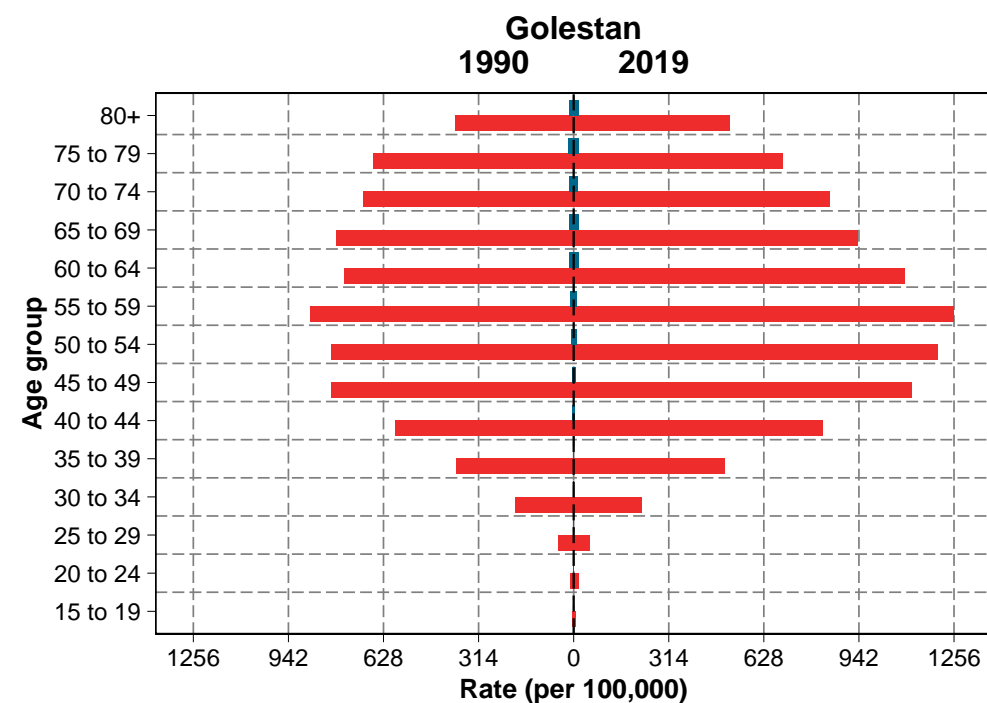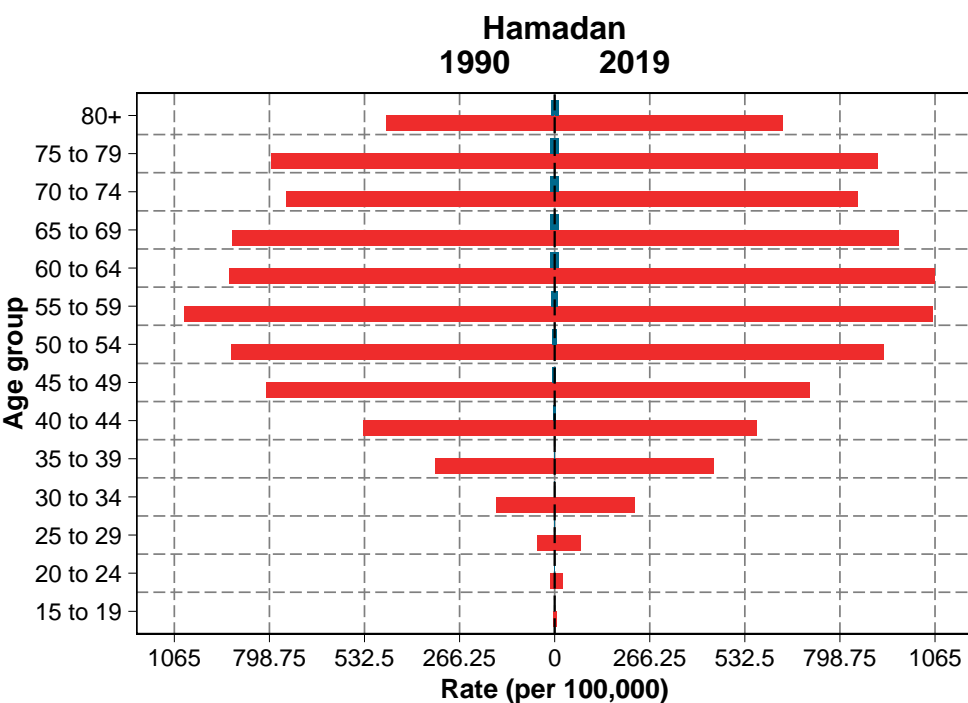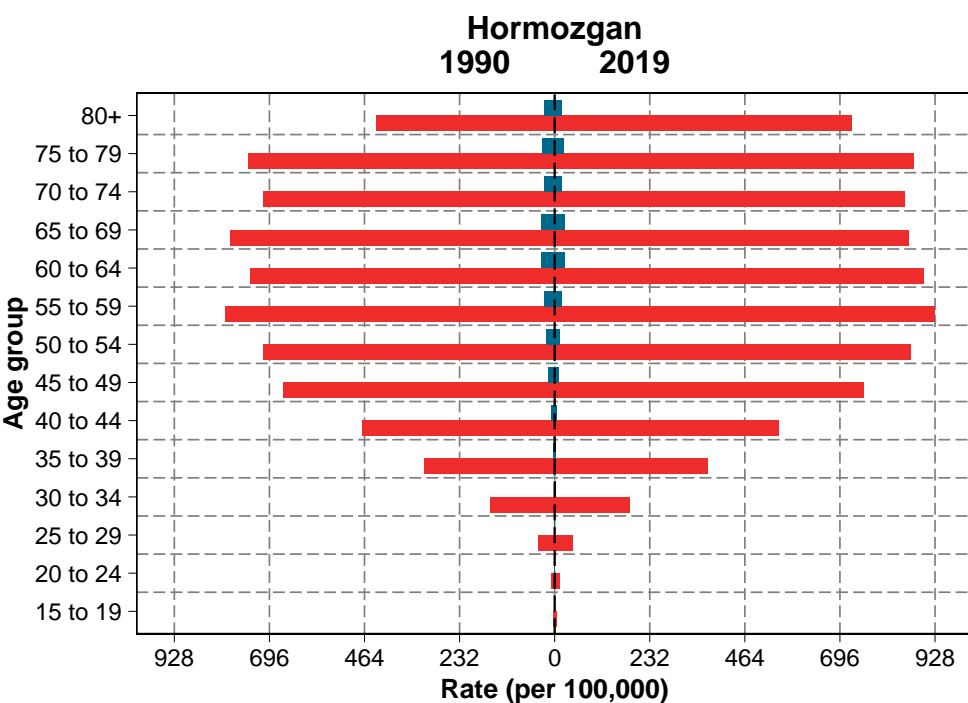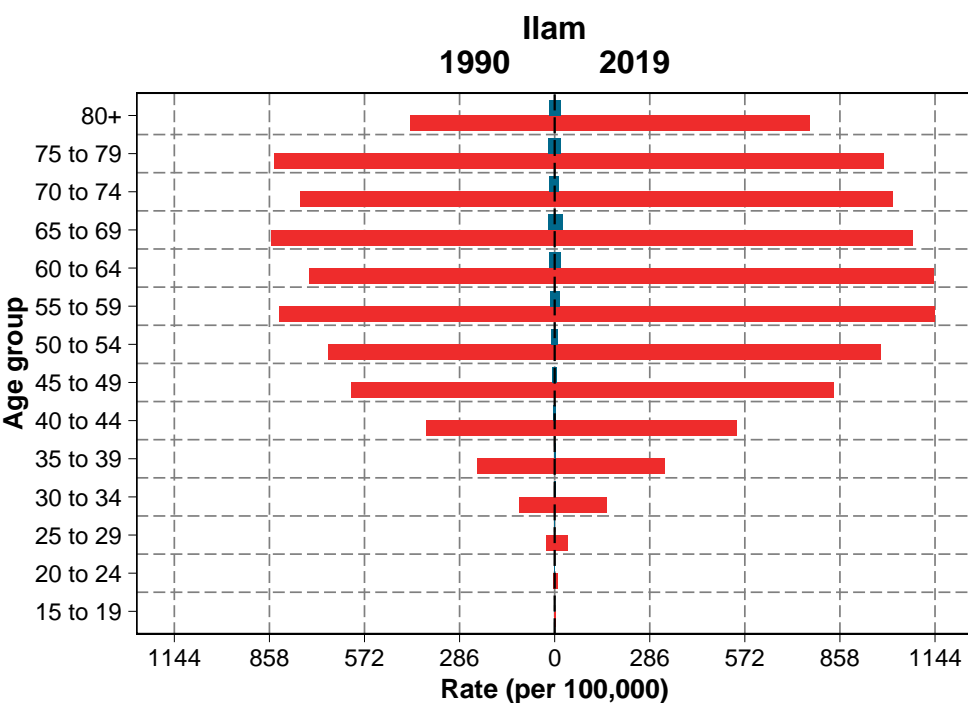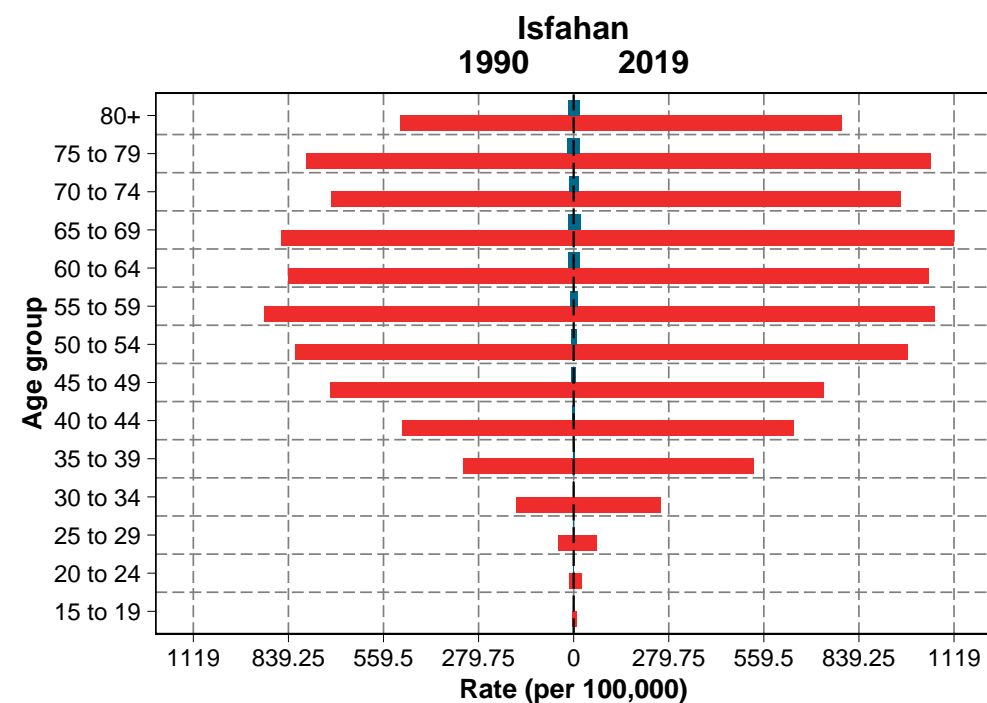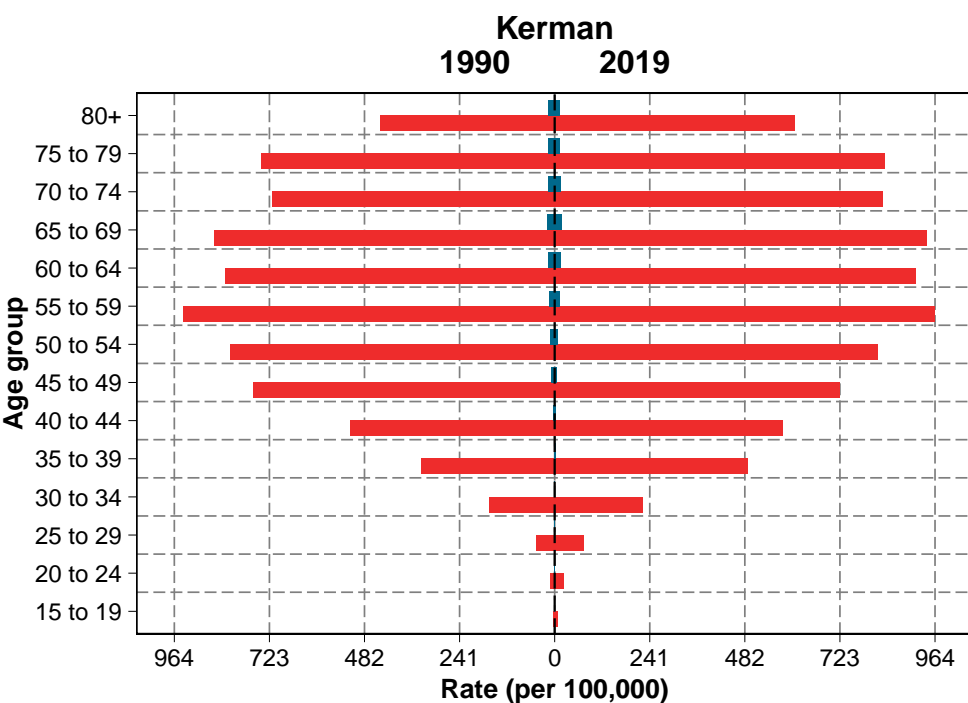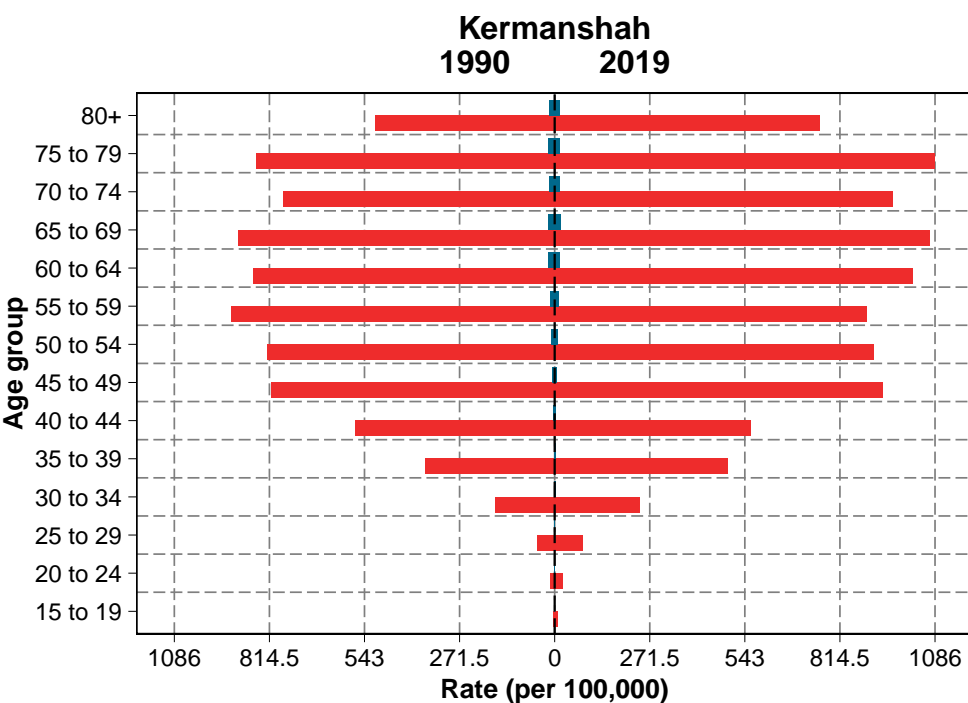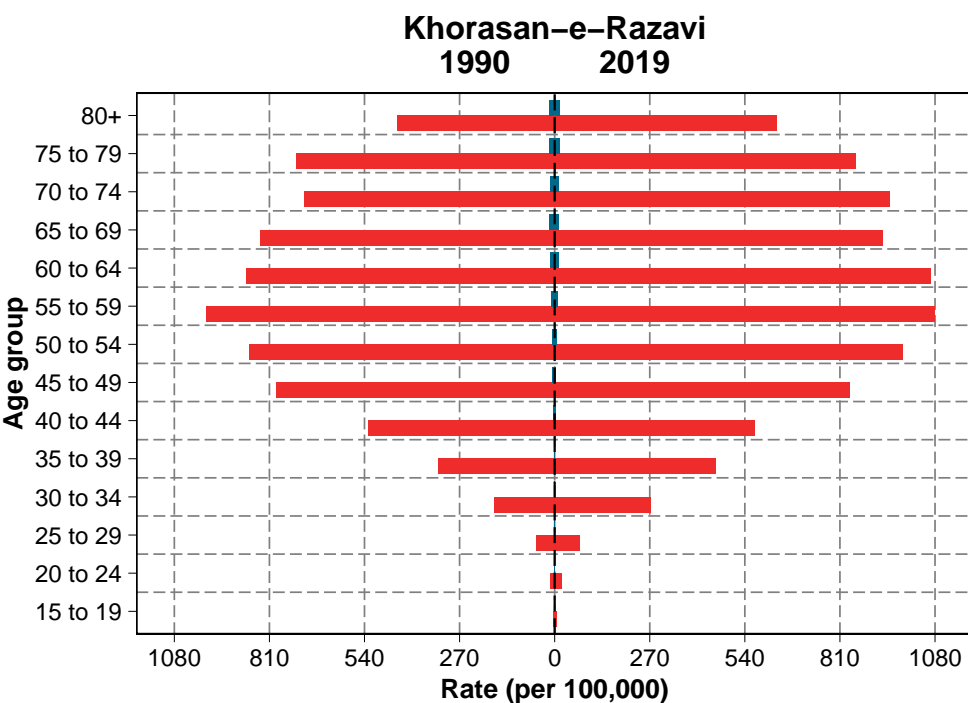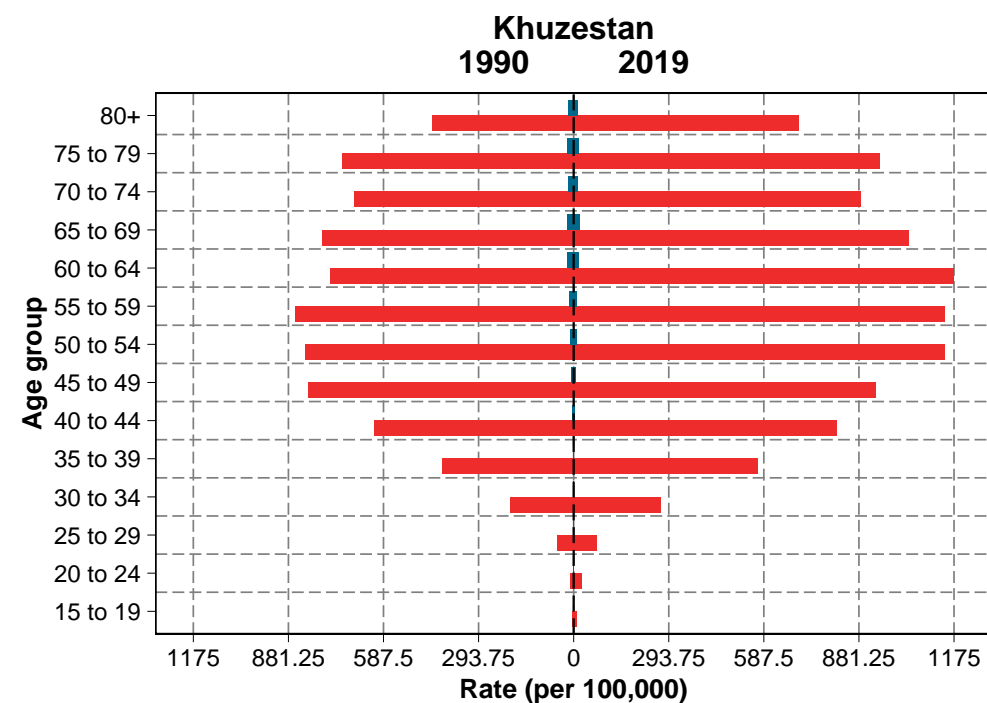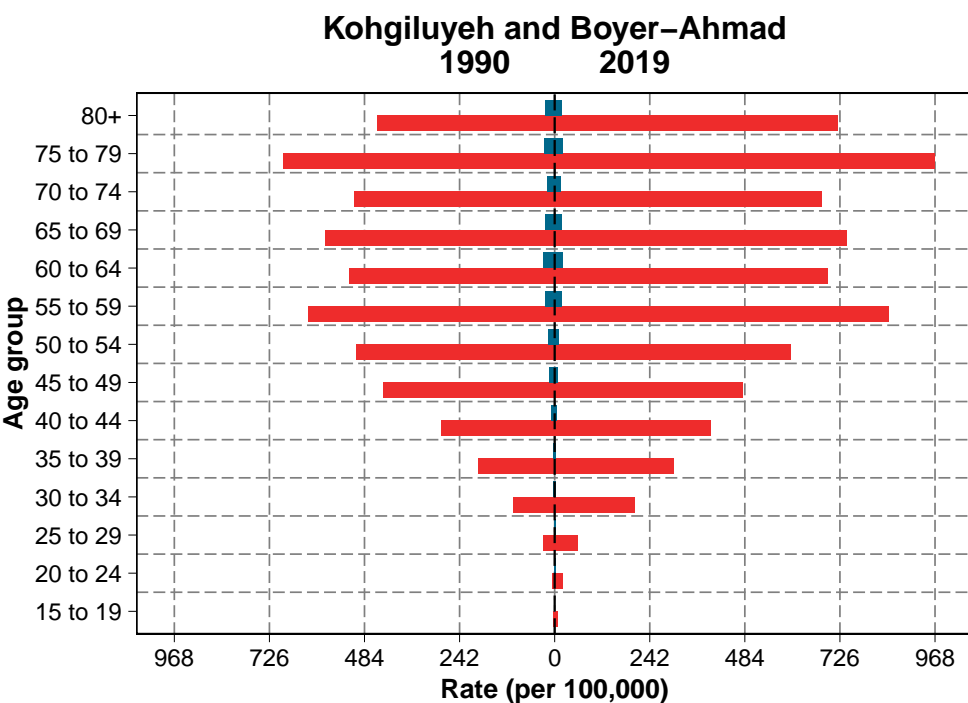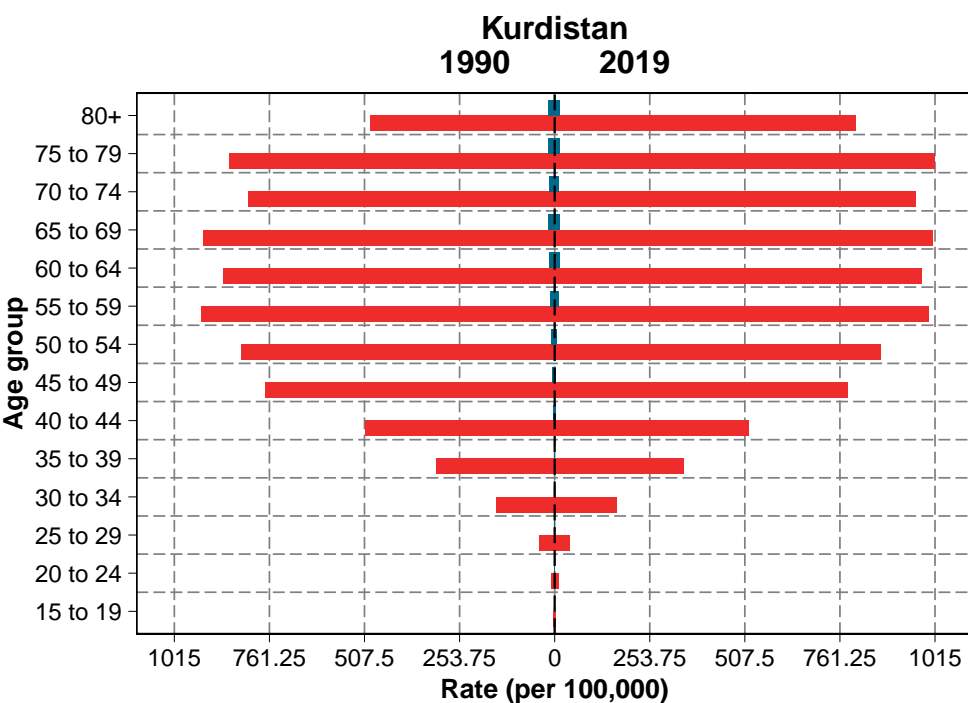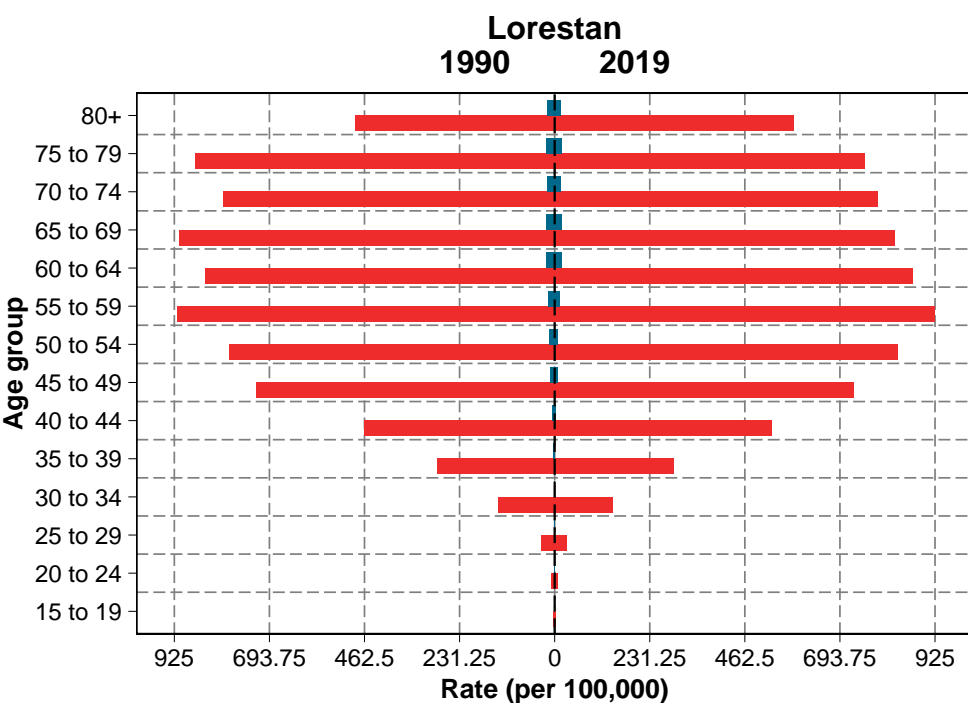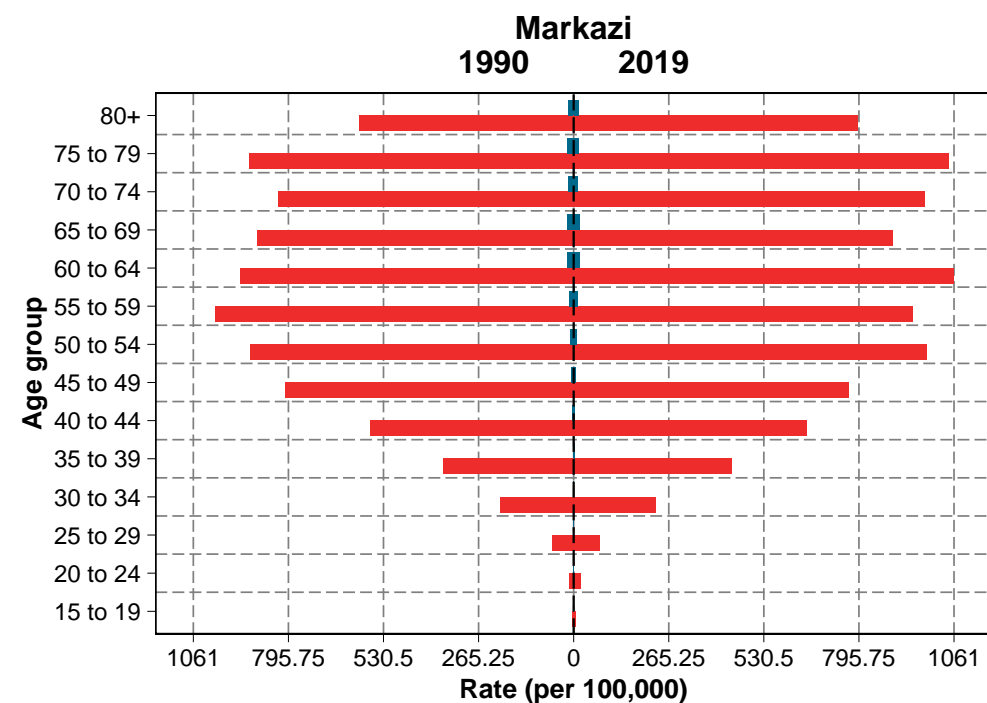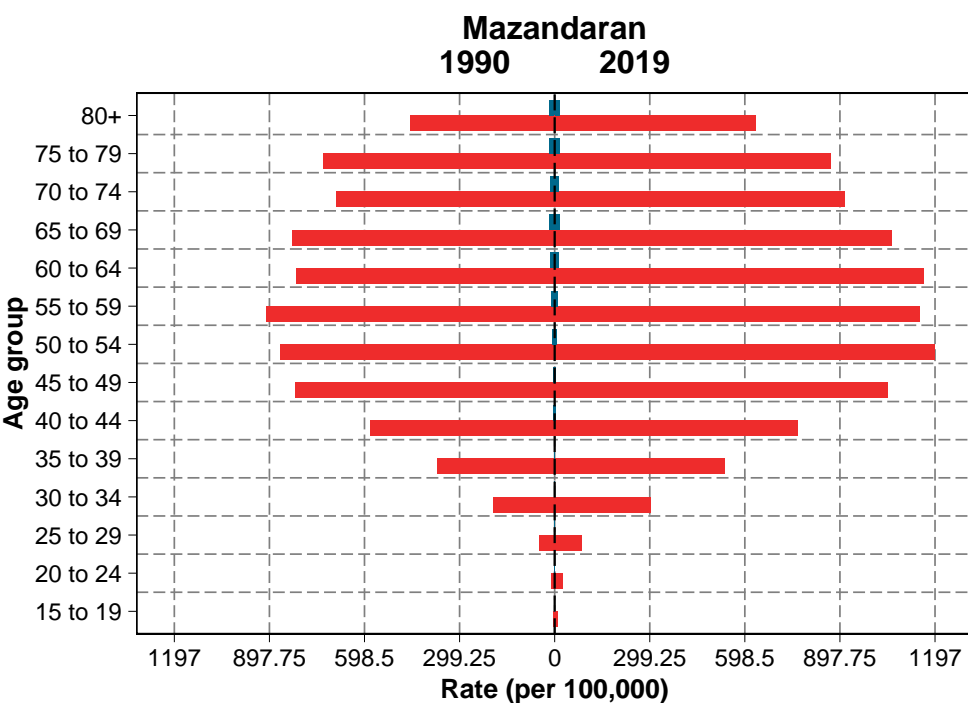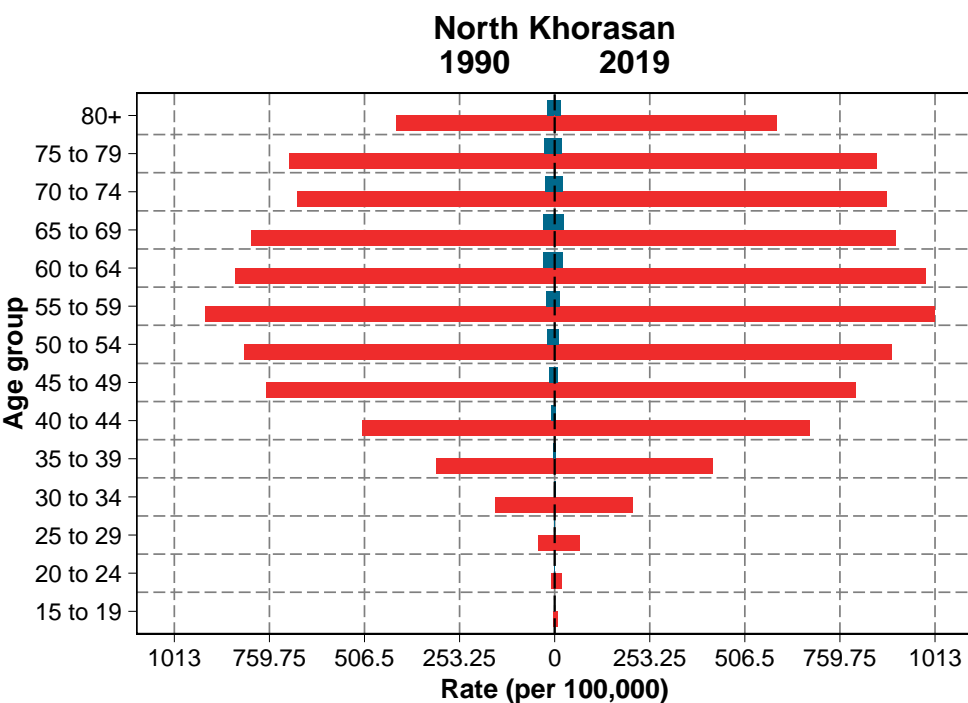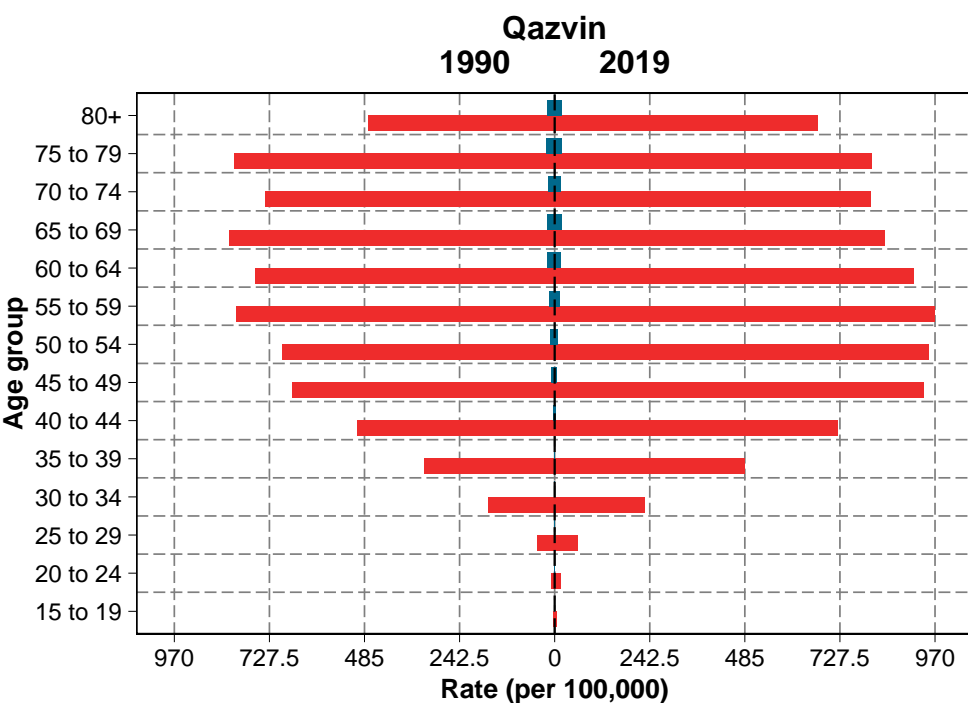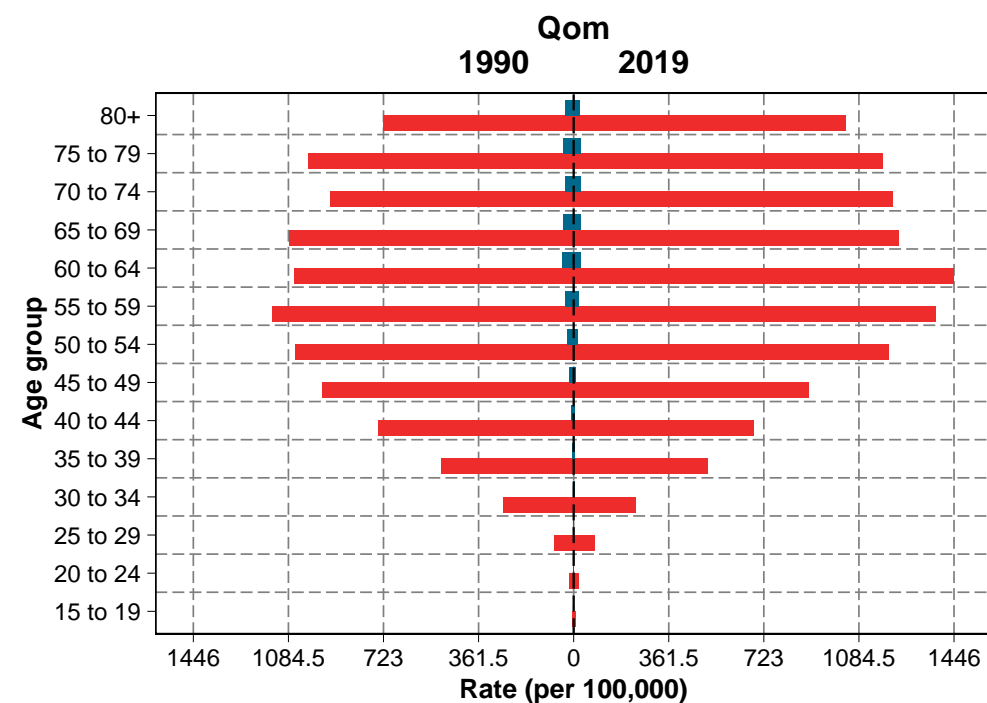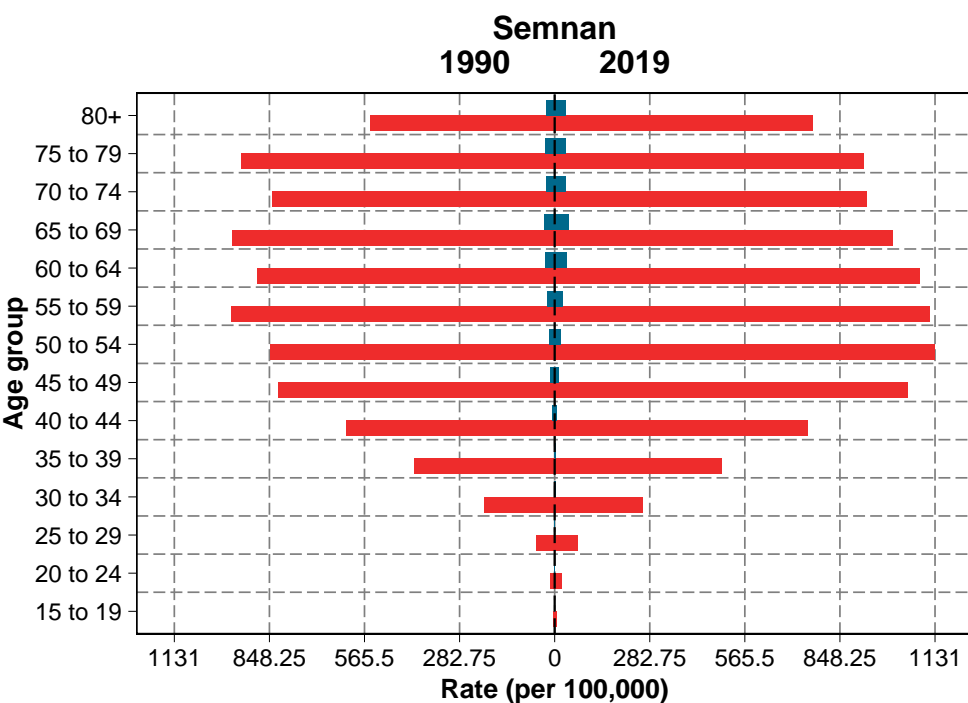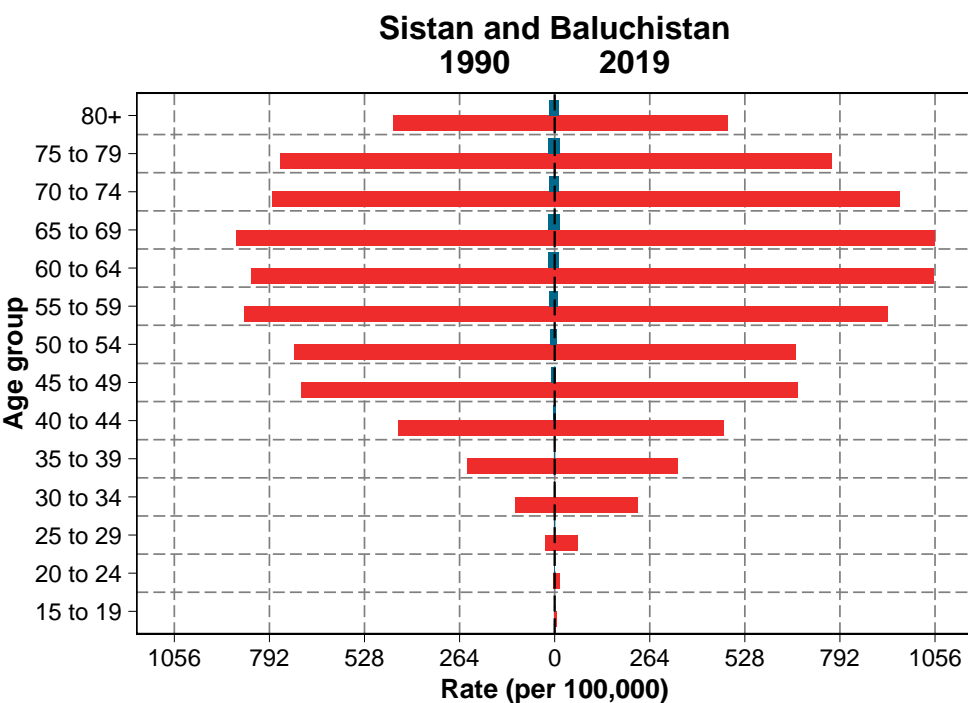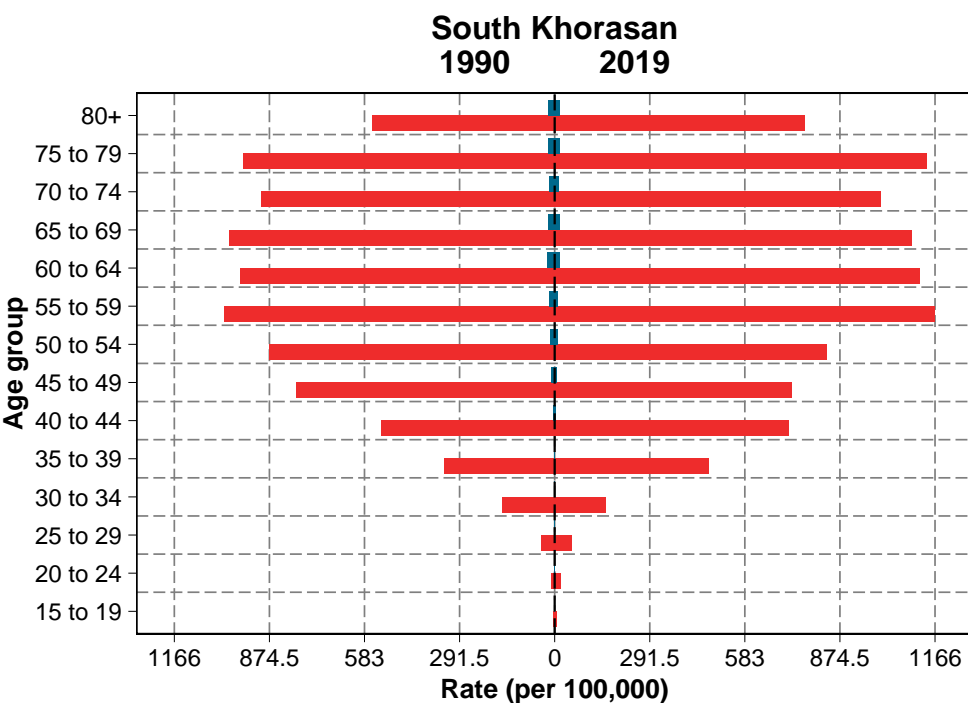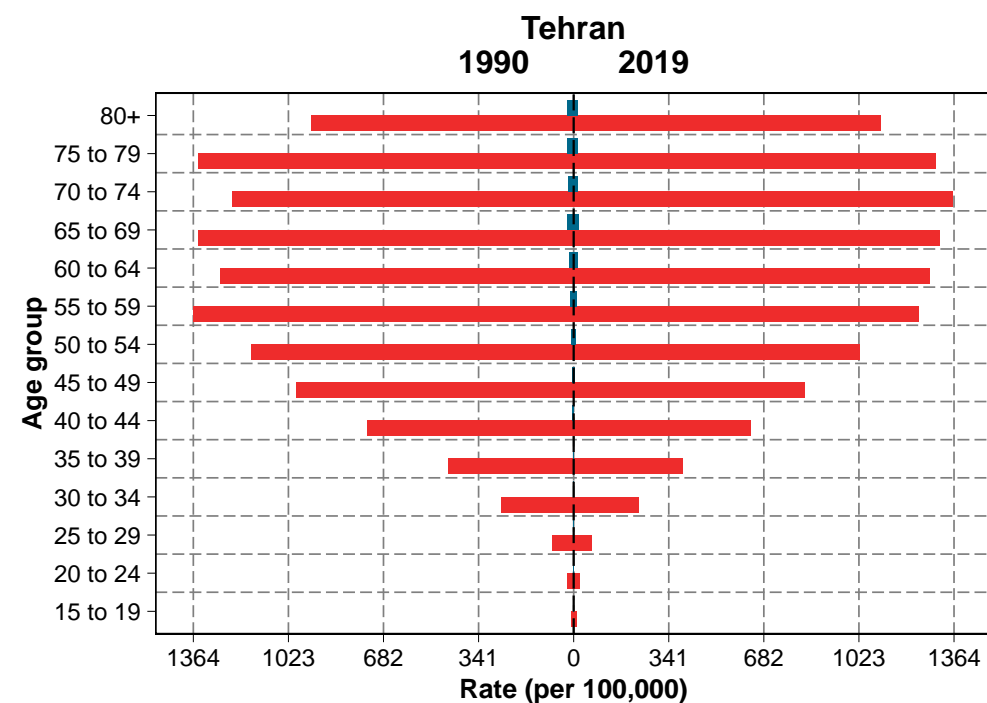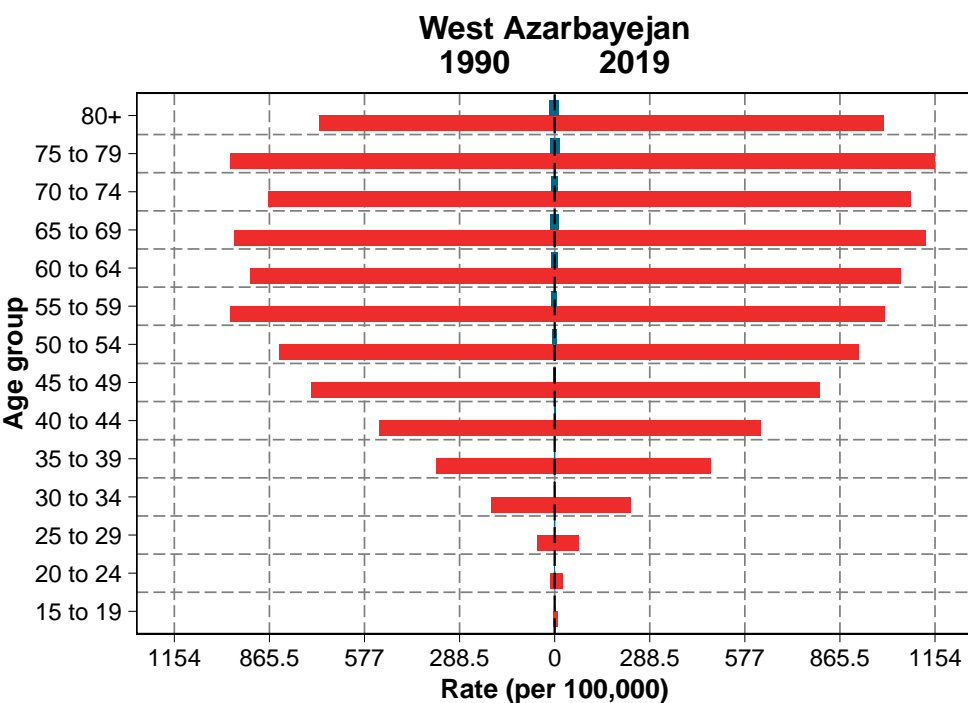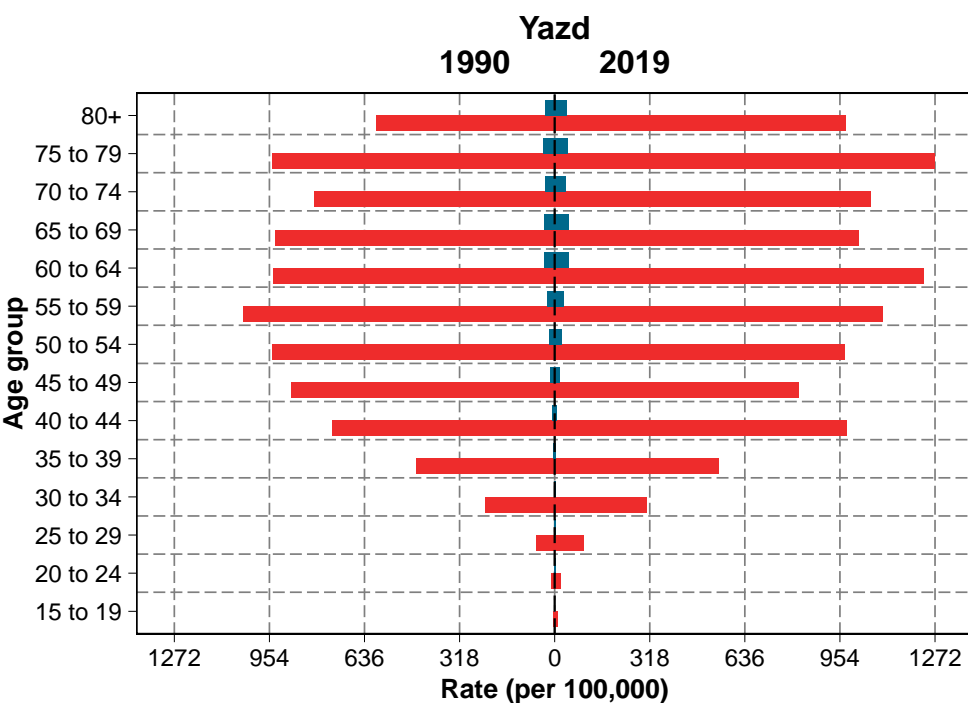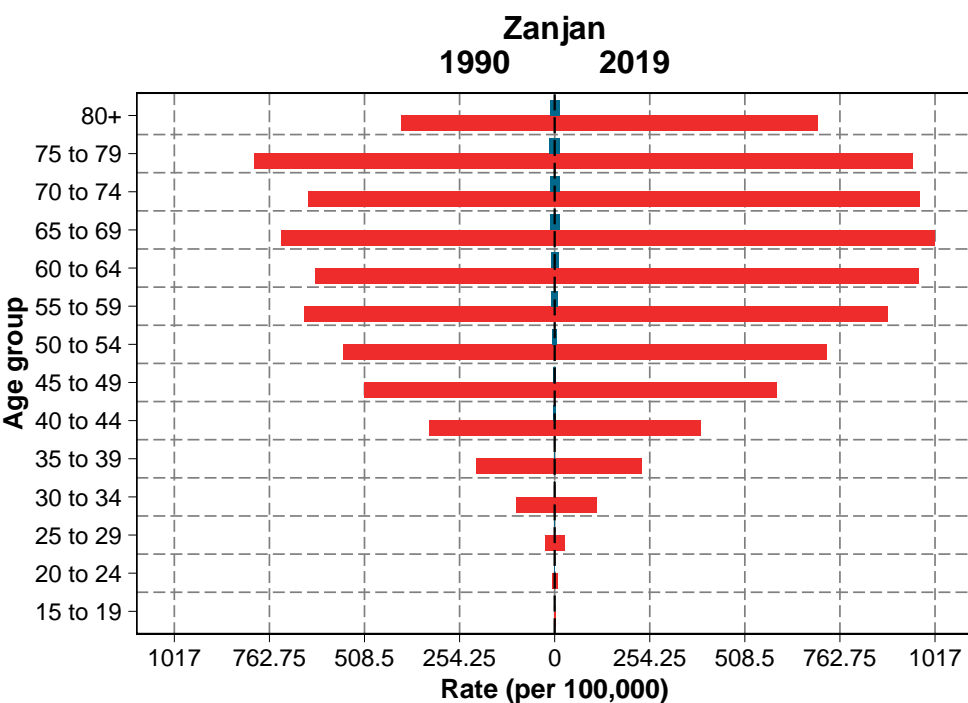

Supplement: Supplementary file 12 — Additional file 12. Fig. 6 Breast cancer disability-adjusted life years (DALYs) rates (per 100,000 population) in 1990 and 2019 based on age groups by sex (red: female; blue: males) in Iran and its 31 provinces. [file 13058_2023_1633_MOESM12_ESM.pdf]
