# Supplementary material for: National and subnational burden of female and male breast cancer and risk factors in Iran from 1990 to 2019: results from the Global Burden of Disease study 2019
Source: Breast Cancer Res. 2023 Apr 26;25:47. doi: 10.1186/s13058-023-01633-4 (PMC10131337; doi:10.1186/s13058-023-01633-4)
Supplement: Supplementary file 7 — Additional file 7. Table 3 Breast cancer age-standardized incidence, prevalence, deaths, disability-adjusted life years (DALYs), years of life lost (YLLs) and years lived with disability (YLDs) rates (per 100,000 population), numbers and percent changes in all 31 provinces of Iran between 1990 and 2019, for both sexes, females, and males (provinces have been sorted alphabetically). [file 13058_2023_1633_MOESM7_ESM.pdf]

Province	Measure	Metric	1990			2019			% Change (1990 to 2019)		
			Both	Female	Male	Both	Female	Male	Both	Female	Male
Alborz	Incidence	Rate*	12.3 (7.7 to 17.6)	25.8 (16.1 to 36.6)	0.4 (0.2 to 0.6)	20.6 (16.5 to 25.9)	41.8 (33.3 to 52.6)	0.5 (0.4 to 0.8)	67.6 (10.8 to 188.2)	62.4 (7.2 to 182)	37.4 (-27 to 172.5)
		Number†	90 (54 to 128)	89 (53 to 127)	1 (1 to 2)	644 (506 to 815)	637 (499 to 807)	7 (5 to 11)	612.5 (368 to 1179)	614.6 (365.9 to 1194.6)	467.7 (195.9 to 1051.5)
	Prevalence	Rate	112.2 (79.1 to 147.4)	235 (164 to 308.2)	3 (2 to 4.6)	189.7 (154.2 to 232.7)	383.7 (311.1 to 470.1)	4.6 (3.3 to 6.5)	69.1 (23.3 to 150.5)	63.3 (18.3 to 144.9)	50.8 (-10.6 to 157.1)
		Number	805 (532 to 1082)	795 (526 to 1071)	10 (7 to 16)	5901 (4751 to 7310)	5837 (4686 to 7246)	64 (45 to 91)	633 (406.4 to 1075.4)	634.6 (406 to 1085.5)	512.9 (245 to 1001.9)
	Deaths	Rate	6.5 (4.2 to 9.6)	13.4 (8.6 to 19.8)	0.3 (0.2 to 0.4)	6.5 (5.3 to 7.9)	13.4 (10.9 to 16.2)	0.2 (0.2 to 0.3)	0.2 (-36.4 to 66.8)	-0.4 (-36.9 to 65.9)	-11.5 (-49.6 to 62.8)
		Number	41 (27 to 60)	41 (26 to 59)	1 (0 to 1)	180 (146 to 220)	177 (143 to 217)	3 (2 to 4)	335.4 (180.2 to 654.5)	336.5 (179.8 to 675.9)	280 (114.3 to 615.9)
	DALYs	Rate	195.6 (127.9 to 280.4)	411.2 (260.3 to 594)	6.8 (3.9 to 10.2)	195.7 (159 to 238.1)	394.2 (319.8 to 480.4)	6.2 (4.4 to 8.3)	0 (-35 to 68.8)	-4.1 (-38 to 65.1)	-8.6 (-47.2 to 72.9)
		Number	1498 (943 to 2154)	1472 (912 to 2124)	25 (14 to 39)	6187 (5024 to 7598)	6097 (4942 to 7508)	90 (64 to 121)	313.1 (173.4 to 584.2)	314.1 (173.7 to 597.6)	255.4 (101.4 to 583.6)
	YLLs	Rate	187.5 (119.1 to 271.2)	394.2 (248 to 570.9)	6.5 (3.7 to 9.7)	181.8 (146.8 to 223.4)	366.2 (295.1 to 450.7)	5.8 (4.1 to 7.7)	-3 (-37 to 67.1)	-7.1 (-40.3 to 63.1)	-11.1 (-49.5 to 68.1)
		Number	1438 (892 to 2073)	1413 (862 to 2044)	24 (14 to 38)	5751 (4604 to 7115)	5667 (4529 to 7031)	85 (60 to 114)	300 (159.5 to 575.7)	300.9 (158.8 to 586.3)	245.6 (94.7 to 574.7)
	YLDs	Rate	8.1 (4.6 to 12.4)	17 (9.5 to 25.9)	0.3 (0.2 to 0.5)	13.9 (9.3 to 19.7)	28 (18.8 to 39.8)	0.4 (0.2 to 0.7)	70.6 (16.8 to 178.4)	64.9 (12.2 to 172.4)	49.3 (-19 to 182.1)
		Number	60 (31 to 92)	59 (30 to 91)	1 (0 to 2)	436 (288 to 621)	430 (282 to 615)	6 (3 to 9)	625.5 (377.8 to 1216.3)	627.4 (374.7 to 1228.5)	509.2 (227.2 to 1074.3)

Province	Measure	Metric	1990			2019			% Change (1990 to 2019)		
			Both	Female	Male	Both	Female	Male	Both	Female	Male
Ardebil	Incidence	Rate	8.3 (5.9 to 11.6)	17.3 (12.1 to 24.1)	0.2 (0.1 to 0.3)	14.2 (11.7 to 17)	28 (23 to 33.4)	0.3 (0.2 to 0.4)	70.9 (15.3 to 157.8)	61.6 (8.9 to 145.7)	30.1 (-25.2 to 137.1)
		Number	48 (34 to 66)	47 (34 to 66)	1 (0 to 1)	189 (155 to 227)	187 (153 to 224)	1 (1 to 2)	294.4 (168.5 to 500.5)	296 (168.9 to 505.9)	157.5 (46.2 to 360.7)
	Prevalence	Rate	76.2 (59.8 to 98)	159.4 (123.8 to 205.3)	1.7 (1.2 to 2.4)	131.2 (110.2 to 153.2)	258.2 (215.5 to 301.5)	2.4 (1.8 to 3.2)	72.2 (29.1 to 134)	61.9 (20.7 to 120.6)	35 (-8.7 to 104.8)
		Number	429 (328 to 556)	424 (323 to 551)	5 (3 to 7)	1730 (1443 to 2043)	1717 (1428 to 2028)	14 (10 to 18)	303.4 (200.3 to 457.7)	304.8 (200.5 to 461.8)	176 (78.7 to 334.5)
	Deaths	Rate	5.1 (3.6 to 7.2)	10.8 (7.4 to 15.2)	0.2 (0.1 to 0.2)	5.4 (4.6 to 6.5)	10.6 (8.8 to 12.5)	0.1 (0.1 to 0.2)	5.9 (-31.5 to 59.5)	-1.8 (-36.9 to 48.3)	-9.2 (-45.1 to 62.5)
		Number	27 (19 to 38)	27 (19 to 37)	0 (0 to 1)	67 (56 to 79)	66 (55 to 79)	1 (1 to 1)	143.3 (63.2 to 262)	144.2 (62.6 to 263.4)	87 (11.5 to 230.4)
	DALYs	Rate	157.3 (111.7 to 215.2)	326.9 (231.3 to 449)	4 (2.5 to 5.9)	161.6 (135.4 to 193.4)	318.5 (266.7 to 381.6)	3.4 (2.6 to 4.7)	2.7 (-30.2 to 52)	-2.6 (-34.3 to 44.4)	-13.5 (-47.8 to 52.5)
		Number	949 (684 to 1290)	937 (674 to 1279)	13 (8 to 19)	2178 (1814 to 2627)	2158 (1796 to 2603)	20 (15 to 28)	129.5 (56.1 to 233.8)	130.4 (56.2 to 236.5)	63.4 (-4.7 to 182.8)
	YLLs	Rate	151.9 (106.6 to 208.6)	315.7 (220.6 to 435.6)	3.8 (2.4 to 5.8)	152 (126.3 to 182.2)	299.7 (248.6 to 360)	3.2 (2.4 to 4.5)	0.1 (-32.5 to 48.7)	-5.1 (-36.4 to 41.2)	-15.5 (-49.5 to 50.9)
		Number	918 (653 to 1250)	906 (642 to 1238)	12 (8 to 18)	2051 (1690 to 2474)	2031 (1672 to 2455)	19 (14 to 27)	123.4 (51.7 to 227.8)	124.3 (51.7 to 229.7)	59.4 (-8 to 179.4)
	YLDs	Rate	5.4 (3.4 to 8)	11.3 (7 to 16.8)	0.1 (0.1 to 0.2)	9.5 (6.5 to 13.3)	18.8 (12.8 to 26.2)	0.2 (0.1 to 0.3)	76.6 (24.6 to 156.1)	67.1 (16.9 to 141.9)	40 (-13.1 to 137.5)
		Number	31 (19 to 47)	31 (19 to 47)	0 (0 to 1)	127 (86 to 179)	126 (85 to 177)	1 (1 to 2)	308.2 (187.4 to 489.3)	309.9 (187 to 493)	179 (65.4 to 372.9)

Province	Measure	Metric	1990			2019			% Change (1990 to 2019)		
			Both	Female	Male	Both	Female	Male	Both	Female	Male
Bushehr	Incidence	Rate	9.1 (5.8 to 13.6)	18.4 (11.7 to 27.6)	0.2 (0.1 to 0.3)	17.5 (14.6 to 20.9)	36.2 (30 to 43.2)	0.2 (0.2 to 0.4)	93.3 (27.8 to 205.2)	97.2 (29.7 to 212.1)	37.8 (-23 to 145.6)
		Number	31 (19 to 45)	30 (19 to 44)	0 (0 to 0)	196 (160 to 237)	195 (160 to 235)	1 (1 to 2)	542.7 (338.6 to 938.7)	544.8 (339.2 to 942.8)	308 (133.2 to 637.3)
	Prevalence	Rate	86 (62.7 to 115.1)	173.7 (125.8 to 233.4)	1.6 (1.1 to 2.2)	161.9 (137.2 to 188.9)	333.1 (281.3 to 389.6)	2.3 (1.7 to 3.1)	88.4 (39.4 to 158)	91.7 (41.4 to 162.9)	40.4 (-5.9 to 112.7)
		Number	280 (192 to 376)	277 (190 to 374)	2 (2 to 3)	1784 (1484 to 2115)	1774 (1475 to 2102)	10 (8 to 14)	538.2 (368.4 to 821.5)	540.1 (369 to 826.7)	326.8 (178.4 to 563.4)
	Deaths	Rate	5.2 (3.4 to 8.1)	10.4 (6.7 to 16.2)	0.1 (0.1 to 0.2)	6 (5.1 to 7)	12.1 (10.2 to 14.1)	0.1 (0.1 to 0.2)	15.1 (-29.5 to 78.2)	15.8 (-29 to 80.6)	-4.9 (-45.2 to 64.3)
		Number	16 (10 to 23)	15 (10 to 23)	0 (0 to 0)	58 (49 to 68)	58 (49 to 68)	0 (0 to 1)	276.6 (140.4 to 486.6)	277.7 (140.1 to 497.5)	181.6 (62.7 to 401.1)
	DALYs	Rate	158.4 (104.8 to 232.4)	321.2 (212.4 to 473.6)	3.3 (2 to 5.1)	186.1 (155.9 to 216.8)	384.1 (321.8 to 447.8)	3 (2.2 to 4)	17.5 (-22.9 to 79)	19.6 (-21.8 to 84.4)	-7.6 (-46.4 to 61.7)
		Number	556 (363 to 810)	550 (356 to 803)	6 (3 to 9)	2104 (1755 to 2450)	2090 (1743 to 2436)	15 (11 to 19)	278.5 (154.4 to 481.7)	279.7 (154.3 to 489.7)	162.3 (51.8 to 362.8)
	YLLs	Rate	152.3 (99.7 to 225.2)	308.9 (201.6 to 457.9)	3.1 (1.9 to 5)	174.3 (146.1 to 203.8)	359.7 (300.3 to 420.7)	2.8 (2.1 to 3.7)	14.5 (-26.1 to 75.4)	16.5 (-25.1 to 80.8)	-9.8 (-47.8 to 59.6)
		Number	535 (351 to 777)	530 (345 to 773)	5 (3 to 8)	1972 (1640 to 2310)	1958 (1629 to 2293)	14 (10 to 18)	268.3 (143.4 to 472.2)	269.5 (143.5 to 476)	155.8 (47.7 to 353.6)
	YLDs	Rate	6.1 (3.4 to 9.9)	12.3 (6.9 to 20.1)	0.1 (0.1 to 0.2)	11.8 (7.9 to 16.4)	24.3 (16.3 to 33.8)	0.2 (0.1 to 0.3)	93.6 (31.2 to 192.7)	97.5 (33.3 to 200.6)	45.5 (-13.7 to 140.6)
		Number	21 (11 to 33)	20 (11 to 33)	0 (0 to 0)	133 (89 to 188)	132 (88 to 186)	1 (1 to 1)	543.9 (336.8 to 906.2)	546.1 (336.1 to 909.3)	332.2 (159.1 to 639.2)

Province	Measure	Metric	1990			2019			% Change (1990 to 2019)		
			Both	Female	Male	Both	Female	Male	Both	Female	Male
Chahar Mahaal and Bakhtiari	Incidence	Rate	8.1 (5.2 to 11.7)	16.7 (10.8 to 24.5)	0.2 (0.1 to 0.3)	12 (9 to 15.7)	23.8 (17.8 to 31.3)	0.2 (0.1 to 0.3)	47.8 (-5.3 to 131.8)	41.9 (-9.7 to 123.9)	15.5 (-37.1 to 104.2)
		Number	27 (18 to 40)	27 (18 to 39)	0 (0 to 0)	116 (86 to 151)	115 (86 to 150)	1 (1 to 1)	321.6 (175.8 to 549.9)	323.2 (175.7 to 552.4)	181.8 (50 to 414.3)
	Prevalence	Rate	77.7 (57.8 to 101.3)	160.8 (119.4 to 211.1)	1.7 (1.2 to 2.4)	117.7 (93.6 to 147.5)	233 (185.1 to 293)	2.1 (1.5 to 2.8)	51.5 (10.6 to 104.1)	44.9 (4.7 to 96.6)	23.4 (-17.7 to 83.6)
		Number	257 (190 to 341)	255 (187 to 338)	3 (2 to 4)	1117 (879 to 1404)	1108 (871 to 1395)	9 (6 to 12)	334 (212.7 to 490.4)	335.4 (212.9 to 496.8)	207.5 (99.5 to 388.7)
	Deaths	Rate	4.5 (2.8 to 6.8)	9.3 (5.8 to 14.1)	0.1 (0.1 to 0.2)	3.9 (3 to 5)	7.5 (5.7 to 9.7)	0.1 (0.1 to 0.1)	-14.5 (-44.2 to 34.2)	-18.6 (-47.4 to 28.6)	-23.1 (-56.3 to 33.4)
		Number	14 (9 to 21)	14 (9 to 20)	0 (0 to 0)	34 (26 to 44)	34 (25 to 43)	0 (0 to 1)	145.1 (62.5 to 273.8)	145.8 (62.3 to 276.8)	96.1 (6.2 to 246.7)
	DALYs	Rate	133.3 (88 to 191.3)	276.9 (181.1 to 399.7)	3.1 (2.1 to 4.8)	118.7 (89.7 to 151.2)	235.7 (177.7 to 300.9)	2.4 (1.6 to 3.2)	-11 (-39.5 to 31.4)	-14.9 (-42.7 to 27)	-23.2 (-57.5 to 29.3)
		Number	470 (316 to 666)	464 (309 to 661)	6 (4 to 9)	1156 (874 to 1483)	1146 (864 to 1471)	10 (7 to 14)	146.1 (67.7 to 264.3)	146.9 (67.5 to 266.9)	78.9 (-0.8 to 205.3)
	YLLs	Rate	127.8 (83.3 to 185.7)	265.5 (170.7 to 387.6)	3 (2 to 4.7)	110.3 (83.4 to 141.7)	219.2 (165.1 to 281.8)	2.2 (1.5 to 3)	-13.7 (-42.5 to 30.6)	-17.4 (-45.3 to 25.8)	-25.4 (-59.1 to 28.1)
		Number	451 (300 to 646)	446 (294 to 642)	6 (4 to 9)	1076 (808 to 1382)	1066 (799 to 1372)	10 (7 to 14)	138.4 (58.3 to 258.1)	139.3 (58 to 260.7)	73.7 (-4.1 to 200)
	YLDs	Rate	5.5 (3.3 to 8.7)	11.4 (6.6 to 18.2)	0.1 (0.1 to 0.2)	8.3 (5.4 to 12.1)	16.5 (10.6 to 24.1)	0.2 (0.1 to 0.3)	51.5 (3.5 to 125.2)	45.3 (-1.5 to 117.4)	24.8 (-25.8 to 107.3)
		Number	19 (11 to 29)	18 (11 to 29)	0 (0 to 0)	80 (51 to 117)	80 (50 to 116)	1 (0 to 1)	330.6 (193.4 to 536.5)	332.2 (193.5 to 547.7)	204.9 (74.6 to 427.5)

Province	Measure	Metric	1990			2019			% Change (1990 to 2019)		
			Both	Female	Male	Both	Female	Male	Both	Female	Male
East Azarbayejan	Incidence	Rate	8 (5.6 to 11.5)	16.4 (11.3 to 23.5)	0.2 (0.1 to 0.3)	17.5 (13.6 to 21.7)	34.7 (27.1 to 43.2)	0.4 (0.3 to 0.5)	117.7 (35.8 to 230.9)	112.3 (31 to 225.7)	65.9 (-7.6 to 201)
		Number	159 (112 to 226)	157 (110 to 223)	2 (1 to 3)	798 (618 to 995)	791 (611 to 989)	7 (5 to 11)	400.6 (216.4 to 664.4)	402.5 (216.5 to 671.3)	254.9 (92 to 565.5)
	Prevalence	Rate	78.5 (60.4 to 101.2)	161.2 (124 to 208.5)	1.8 (1.3 to 2.6)	159.5 (130.5 to 193.1)	317.2 (259 to 384.8)	3.1 (2.2 to 4.2)	103.3 (45.7 to 174.4)	96.8 (39.8 to 165.5)	65.7 (5.8 to 159.5)
		Number	1503 (1142 to 1965)	1486 (1126 to 1949)	17 (11 to 25)	7261 (5869 to 8883)	7199 (5805 to 8824)	62 (44 to 85)	383 (238.6 to 569.6)	384.4 (238.6 to 574.5)	261.7 (120.1 to 488.2)
	Deaths	Rate	4.7 (3.2 to 6.8)	9.6 (6.5 to 14)	0.2 (0.1 to 0.3)	6.2 (5 to 7.7)	12.3 (9.9 to 15.2)	0.2 (0.1 to 0.3)	31.7 (-17.1 to 101.9)	27.5 (-19.8 to 97.8)	14.5 (-33.8 to 96.7)
		Number	85 (58 to 120)	84 (57 to 118)	1 (1 to 2)	260 (210 to 324)	257 (206 to 319)	4 (2 to 5)	204.7 (99.6 to 364.9)	205.6 (98.9 to 370.4)	152.6 (44.6 to 337)
	DALYs	Rate	150.4 (103.6 to 208.6)	306.8 (209.7 to 426.9)	4.2 (2.7 to 6.6)	197.6 (160.9 to 243.4)	392.5 (318 to 483.1)	4.8 (3.5 to 6.7)	31.3 (-12.6 to 98)	27.9 (-15.4 to 93.8)	14.2 (-32.9 to 93.9)
		Number	3127 (2162 to 4298)	3083 (2099 to 4258)	44 (27 to 67)	9146 (7388 to 11335)	9045 (7287 to 11205)	101 (72 to 139)	192.5 (96.1 to 340.6)	193.4 (95.5 to 343.7)	131.6 (35.1 to 306.9)
	YLLs	Rate	145 (98.8 to 201.2)	295.7 (199.8 to 414.3)	4.1 (2.6 to 6.4)	185.9 (148.8 to 232.4)	369.2 (294.8 to 461.7)	4.6 (3.2 to 6.4)	28.2 (-15.8 to 95.1)	24.9 (-18.6 to 92.5)	11.8 (-35 to 93.3)
		Number	3019 (2054 to 4170)	2977 (2006 to 4110)	42 (26 to 65)	8608 (6885 to 10750)	8512 (6788 to 10647)	95 (67 to 132)	185.1 (88.6 to 339.6)	186 (87.5 to 343.1)	126.5 (30.6 to 296.8)
	YLDs	Rate	5.5 (3.3 to 8.6)	11.2 (6.7 to 17.7)	0.2 (0.1 to 0.3)	11.7 (7.7 to 16.5)	23.3 (15.3 to 32.9)	0.3 (0.2 to 0.4)	114.6 (42.6 to 217)	108.8 (37 to 209.7)	75.1 (1.4 to 198.8)
		Number	108 (64 to 169)	107 (63 to 167)	1 (1 to 2)	539 (351 to 762)	533 (346 to 755)	6 (3 to 9)	397.8 (228.7 to 638.1)	399.5 (227.6 to 647)	275.9 (110.2 to 567.4)

Province	Measure	Metric	1990			2019			% Change (1990 to 2019)		
			Both	Female	Male	Both	Female	Male	Both	Female	Male
Fars	Incidence	Rate	8.3 (5.3 to 12.5)	16.7 (10.7 to 25.3)	0.2 (0.1 to 0.3)	18.3 (14.4 to 23.1)	36.6 (28.7 to 46.3)	0.3 (0.2 to 0.5)	121.8 (36.2 to 272.1)	119.9 (34.1 to 273.1)	54.8 (-16.3 to 187.7)
		Number	158 (102 to 234)	156 (101 to 232)	2 (1 to 3)	1002 (783 to 1268)	994 (775 to 1262)	8 (5 to 12)	533.6 (291.1 to 966.7)	536.4 (291.2 to 974.2)	308.2 (117.2 to 675.7)
	Prevalence	Rate	82.2 (60.9 to 109.6)	165.5 (121.4 to 221.8)	1.9 (1.3 to 2.6)	167.9 (136.5 to 205.5)	335.5 (272.9 to 411)	2.9 (2.1 to 4.1)	104.3 (42.6 to 192.5)	102.8 (40.8 to 193.2)	54.9 (-2.2 to 145.4)
		Number	1506 (1080 to 2021)	1490 (1065 to 2008)	17 (11 to 24)	9095 (7316 to 11273)	9026 (7251 to 11207)	69 (49 to 97)	503.7 (313.9 to 793.3)	505.8 (314.6 to 797.9)	316.2 (157.6 to 582.3)
	Deaths	Rate	4.5 (3 to 6.8)	8.9 (5.8 to 13.5)	0.1 (0.1 to 0.2)	6 (4.8 to 7.3)	11.9 (9.5 to 14.6)	0.2 (0.1 to 0.2)	33 (-20.3 to 118.4)	32.9 (-20.6 to 118.6)	6 (-39.1 to 93.4)
		Number	77 (51 to 115)	76 (50 to 114)	1 (1 to 2)	295 (238 to 364)	292 (233 to 361)	3 (2 to 5)	281.9 (134.2 to 530.6)	283.3 (133.7 to 535.8)	193.1 (56.6 to 454.2)
	DALYs	Rate	140.2 (94.6 to 204.8)	284.3 (190.9 to 417.8)	3.9 (2.3 to 6)	193.2 (156 to 236.5)	385.4 (311.2 to 472.5)	4.2 (2.9 to 5.8)	37.7 (-12.4 to 122.2)	35.6 (-14.4 to 119.3)	8.1 (-39.3 to 103)
		Number	2777 (1870 to 4048)	2739 (1834 to 3999)	38 (23 to 60)	10649 (8560 to 13207)	10545 (8458 to 13079)	104 (73 to 148)	283.4 (147.3 to 516.2)	285 (146.8 to 523.1)	171.9 (46.5 to 417.1)
	YLLs	Rate	134.5 (90.6 to 197.3)	272.9 (182.3 to 402.5)	3.7 (2.2 to 5.8)	180.8 (144.3 to 225.6)	360.8 (287.3 to 452.3)	3.9 (2.7 to 5.5)	34.4 (-16.3 to 119.8)	32.2 (-18.1 to 118.1)	5.8 (-41.7 to 100.9)
		Number	2669 (1795 to 3889)	2632 (1752 to 3851)	37 (22 to 57)	9972 (7919 to 12536)	9874 (7822 to 12451)	98 (69 to 140)	273.6 (136.6 to 516.4)	275.1 (136.5 to 519.3)	165.9 (43.3 to 413.7)
	YLDs	Rate	5.7 (3.3 to 9.1)	11.5 (6.5 to 18.4)	0.2 (0.1 to 0.3)	12.3 (8.1 to 18.3)	24.6 (16.1 to 36.5)	0.3 (0.2 to 0.4)	116.9 (40 to 246.7)	115 (37.8 to 246.4)	62.1 (-6.3 to 185.3)
		Number	108 (62 to 175)	107 (61 to 174)	1 (1 to 2)	677 (440 to 1008)	671 (436 to 1000)	6 (4 to 10)	524.6 (298.5 to 923.8)	527.2 (298.7 to 937.9)	327.7 (139.1 to 675.2)

Province	Measure	Metric	1990			2019			% Change (1990 to 2019)		
			Both	Female	Male	Both	Female	Male	Both	Female	Male
Gilan	Incidence	Rate	9.4 (6.2 to 13.6)	18.3 (12 to 26.5)	0.3 (0.2 to 0.5)	19.2 (15.2 to 23.8)	37.8 (29.9 to 47)	0.4 (0.3 to 0.6)	103.9 (29.4 to 218.3)	106 (29.9 to 223.6)	29.8 (-26.9 to 140.4)
		Number	137 (90 to 197)	135 (88 to 195)	2 (1 to 3)	646 (510 to 805)	640 (504 to 800)	6 (4 to 9)	372.4 (204.7 to 659.2)	375 (203.9 to 664.6)	202.5 (71.1 to 468.1)
	Prevalence	Rate	88.3 (66.5 to 115)	170.9 (127.6 to 225)	2.2 (1.6 to 3.2)	175.6 (143.1 to 212.7)	345.2 (281 to 418.8)	3.4 (2.5 to 4.7)	98.9 (43 to 178.2)	102 (43.8 to 184.7)	49.3 (-3.9 to 138.6)
		Number	1236 (905 to 1644)	1220 (889 to 1627)	15 (10 to 22)	5893 (4785 to 7171)	5841 (4730 to 7116)	53 (38 to 75)	377 (240.7 to 590.1)	378.6 (239.6 to 593.4)	249.7 (118.4 to 483.5)
	Deaths	Rate	5 (3.4 to 7.2)	9.5 (6.4 to 13.6)	0.2 (0.1 to 0.3)	6.3 (5.1 to 7.6)	12.3 (10 to 14.9)	0.2 (0.1 to 0.3)	26.2 (-17.6 to 98.1)	29.8 (-15.6 to 104.9)	-11 (-47.2 to 56)
		Number	66 (46 to 95)	65 (44 to 94)	1 (1 to 2)	205 (166 to 247)	202 (163 to 244)	3 (2 to 4)	208.9 (105.8 to 380.5)	210.4 (105.2 to 391.9)	125.7 (31.5 to 299.5)
	DALYs	Rate	157 (109.9 to 217.1)	307.6 (214.4 to 429.8)	5.4 (3.4 to 8)	202.3 (165.1 to 244.1)	396.9 (322.7 to 480.1)	4.9 (3.5 to 6.8)	28.9 (-13.6 to 98.5)	29 (-14.3 to 100.5)	-9.7 (-46 to 54.4)
		Number	2353 (1652 to 3258)	2314 (1620 to 3225)	40 (26 to 59)	6877 (5605 to 8304)	6799 (5527 to 8219)	78 (56 to 109)	192.2 (95.3 to 354.4)	193.8 (95.9 to 363.1)	97.3 (15.1 to 244.1)
	YLLs	Rate	150.4 (104.6 to 209.1)	294.8 (203.1 to 414.1)	5.2 (3.3 to 7.6)	189.4 (152.9 to 229.8)	371.5 (299.4 to 451.8)	4.6 (3.3 to 6.5)	25.9 (-16.1 to 97.4)	26 (-16.8 to 99.5)	-11.7 (-47.5 to 52.8)
		Number	2258 (1567 to 3154)	2220 (1530 to 3109)	38 (24 to 57)	6442 (5203 to 7821)	6369 (5135 to 7746)	74 (52 to 103)	185.2 (88.7 to 349.7)	186.8 (88.2 to 356.8)	92.7 (12.1 to 242.2)
	YLDs	Rate	6.6 (4 to 9.9)	12.8 (7.7 to 19.1)	0.2 (0.1 to 0.4)	12.9 (8.4 to 18.5)	25.4 (16.6 to 36.4)	0.3 (0.2 to 0.5)	96.9 (32.7 to 196)	99.1 (33 to 202.9)	37.7 (-20.1 to 142.7)
		Number	95 (58 to 143)	93 (57 to 141)	1 (1 to 2)	435 (285 to 624)	430 (281 to 617)	5 (3 to 8)	357.8 (202 to 603.6)	360.1 (202.8 to 607.4)	216.7 (83.5 to 470.5)

Province	Measure	Metric	1990			2019			% Change (1990 to 2019)		
			Both	Female	Male	Both	Female	Male	Both	Female	Male
Golestan	Incidence	Rate	7.1 (4.7 to 10.2)	14.3 (9.4 to 20.7)	0.2 (0.1 to 0.3)	15.9 (12.8 to 19.4)	31 (24.9 to 37.9)	0.3 (0.2 to 0.4)	123.3 (40.8 to 253.9)	116.5 (35.1 to 249.2)	44.7 (-14.2 to 150.9)
		Number	48 (32 to 68)	47 (31 to 68)	1 (0 to 1)	308 (248 to 380)	306 (246 to 378)	2 (1 to 3)	542.3 (308 to 912.9)	545.5 (307.6 to 929.6)	270.6 (115.9 to 587.2)
	Prevalence	Rate	68.3 (51.6 to 88.8)	137.3 (103.1 to 180.2)	1.6 (1.1 to 2.2)	142.5 (117.5 to 170.8)	278.1 (229.1 to 333.6)	2.3 (1.7 to 3.1)	108.6 (49.5 to 189.2)	102.5 (44.3 to 184.8)	47.2 (0.8 to 119.1)
		Number	436 (323 to 581)	431 (318 to 575)	5 (3 to 7)	2727 (2243 to 3302)	2709 (2226 to 3286)	18 (13 to 25)	525.4 (338.6 to 798)	528.1 (337.7 to 803.1)	284.5 (154.2 to 506.9)
	Deaths	Rate	4.5 (3 to 6.5)	8.9 (5.9 to 13)	0.1 (0.1 to 0.2)	6 (4.9 to 7.4)	11.7 (9.5 to 14.3)	0.1 (0.1 to 0.2)	35 (-17.3 to 111.5)	30.9 (-20.4 to 107.7)	0.7 (-40.5 to 72.5)
		Number	27 (19 to 39)	27 (18 to 39)	0 (0 to 1)	107 (87 to 131)	106 (86 to 129)	1 (1 to 1)	289.6 (143.5 to 502.2)	291.4 (143.7 to 510.2)	167.3 (52.7 to 372.8)
	DALYs	Rate	142.9 (98.3 to 200.9)	288.9 (197.4 to 408.6)	3.7 (2.3 to 5.6)	200.4 (164.1 to 243.4)	391.3 (320.1 to 475.6)	3.8 (2.7 to 5.3)	40.3 (-12.5 to 114)	35.4 (-16.1 to 108.2)	2.8 (-40.1 to 74.3)
		Number	1010 (700 to 1425)	997 (689 to 1407)	13 (8 to 19)	3947 (3246 to 4808)	3915 (3216 to 4777)	32 (23 to 44)	290.9 (147.2 to 495.2)	292.6 (147.1 to 499.9)	151 (46.5 to 338.3)
	YLLs	Rate	138.1 (94.1 to 196.1)	279.2 (189.3 to 398.5)	3.6 (2.2 to 5.5)	189.8 (154.2 to 231.4)	370.7 (301.2 to 451.8)	3.6 (2.6 to 5)	37.5 (-14.5 to 112.3)	32.7 (-18 to 106.8)	0.8 (-41.9 to 73.1)
		Number	978 (673 to 1390)	965 (660 to 1374)	12 (8 to 19)	3742 (3065 to 4583)	3712 (3029 to 4552)	30 (21 to 42)	282.7 (139.8 to 489.9)	284.4 (140.3 to 494.6)	146.2 (43 to 328.7)
	YLDs	Rate	4.8 (2.8 to 7.5)	9.7 (5.7 to 15.2)	0.1 (0.1 to 0.2)	10.6 (6.9 to 15.2)	20.6 (13.5 to 29.7)	0.2 (0.1 to 0.3)	119.9 (44.4 to 234.1)	113.4 (38.9 to 226.5)	54.8 (-3.8 to 148.7)
		Number	32 (19 to 50)	32 (18 to 50)	0 (0 to 1)	205 (135 to 299)	204 (133 to 297)	2 (1 to 3)	539.1 (311.7 to 876)	542.2 (311.1 to 888.7)	294.1 (138.8 to 567.2)

Province	Measure	Metric	1990			2019			% Change (1990 to 2019)		
			Both	Female	Male	Both	Female	Male	Both	Female	Male
Hamadan	Incidence	Rate	8.3 (5.9 to 11.8)	16.9 (11.9 to 24.3)	0.2 (0.1 to 0.3)	14.5 (11.6 to 17.7)	28.8 (23.1 to 35.2)	0.2 (0.1 to 0.3)	75.2 (13.9 to 157.5)	70.9 (10.8 to 153.3)	19.6 (-34.3 to 117.6)
		Number	78 (55 to 110)	77 (55 to 109)	1 (0 to 1)	284 (227 to 347)	283 (225 to 345)	2 (1 to 3)	266.3 (137.7 to 433.7)	267.7 (137.8 to 437.5)	130.8 (25.6 to 315.7)
	Prevalence	Rate	79.2 (61.2 to 103.5)	161.7 (124 to 212.2)	1.6 (1.2 to 2.3)	135.4 (112.1 to 162.5)	268.5 (221.4 to 323)	2 (1.5 to 2.6)	70.9 (24.4 to 128)	66.1 (20.1 to 122.1)	23.4 (-17.3 to 85.4)
		Number	719 (554 to 950)	712 (549 to 944)	7 (5 to 10)	2629 (2167 to 3173)	2611 (2150 to 3157)	18 (13 to 24)	265.7 (159.8 to 392.7)	266.9 (159.9 to 395.6)	147.7 (62.2 to 278.5)
	Deaths	Rate	4.8 (3.4 to 7)	9.8 (6.8 to 14.4)	0.1 (0.1 to 0.2)	5.4 (4.4 to 6.6)	10.6 (8.7 to 13)	0.1 (0.1 to 0.1)	11.7 (-28.1 to 69.2)	8.6 (-30.5 to 66)	-15 (-51.7 to 40.9)
		Number	42 (30 to 60)	41 (29 to 60)	1 (0 to 1)	100 (81 to 122)	99 (81 to 121)	1 (1 to 1)	138.9 (56.9 to 257.2)	139.7 (56.5 to 259.9)	77.9 (0.5 to 193.8)
	DALYs	Rate	150.9 (109.1 to 215.1)	307.1 (220.6 to 441.8)	3.3 (2.1 to 5.2)	168.8 (137.3 to 205.9)	335 (271.7 to 408.4)	2.8 (2 to 3.8)	11.9 (-25.2 to 63.5)	9.1 (-27.5 to 60.5)	-15.5 (-51.9 to 40.9)
		Number	1458 (1058 to 2050)	1441 (1038 to 2034)	17 (11 to 26)	3324 (2694 to 4045)	3298 (2670 to 4020)	26 (18 to 36)	128 (53.2 to 232.1)	128.9 (52.9 to 234.5)	54.9 (-12.7 to 158.6)
	YLLs	Rate	145.3 (104.4 to 207.1)	295.8 (211 to 425.5)	3.2 (2 to 5.1)	159 (128.1 to 195.4)	315.6 (254.2 to 386.9)	2.6 (1.8 to 3.6)	9.4 (-27.6 to 61.9)	6.7 (-29.9 to 58.2)	-17.2 (-53.1 to 39.3)
		Number	1406 (1005 to 1993)	1390 (989 to 1976)	16 (10 to 25)	3132 (2521 to 3835)	3108 (2495 to 3807)	24 (17 to 34)	122.8 (47.4 to 226.4)	123.6 (47.5 to 228.2)	51.5 (-15.1 to 153.4)
	YLDs	Rate	5.6 (3.4 to 8.7)	11.3 (6.9 to 17.9)	0.1 (0.1 to 0.2)	9.8 (6.5 to 14)	19.5 (12.8 to 27.7)	0.2 (0.1 to 0.3)	76.1 (19.1 to 158.7)	71.5 (15.7 to 153.1)	27.6 (-24 to 113.6)
		Number	52 (32 to 81)	51 (31 to 81)	1 (0 to 1)	192 (127 to 275)	191 (125 to 274)	1 (1 to 2)	269.3 (145.3 to 442.9)	270.7 (144.8 to 446.8)	147.6 (46.7 to 311.1)

Province	Measure	Metric	1990			2019			% Change (1990 to 2019)		
			Both	Female	Male	Both	Female	Male	Both	Female	Male
Hormozgan	Incidence	Rate	6 (3.7 to 8.6)	12.4 (7.6 to 17.8)	0.4 (0.2 to 0.5)	12.8 (10.3 to 15.8)	25.5 (20.4 to 31.4)	0.4 (0.3 to 0.6)	113 (38.1 to 251.4)	106 (31 to 246)	7.7 (-41.7 to 104.5)
		Number	27 (15 to 38)	26 (15 to 37)	1 (0 to 1)	196 (156 to 243)	193 (154 to 240)	3 (2 to 4)	635.2 (370.8 to 1194.7)	649 (375.3 to 1220.8)	210.9 (61.8 to 518.9)
	Prevalence	Rate	58.2 (42.8 to 74.9)	120.5 (88.5 to 155.6)	2.6 (1.7 to 3.6)	116.5 (96.5 to 138.9)	231.7 (191.7 to 277)	3.4 (2.5 to 4.6)	100.4 (47.4 to 174.5)	92.3 (41 to 169.9)	31.1 (-20.8 to 117.6)
		Number	247 (175 to 324)	242 (169 to 316)	6 (4 to 9)	1755 (1439 to 2134)	1733 (1416 to 2111)	22 (16 to 30)	609.3 (398.6 to 931.4)	617.3 (401.8 to 946.5)	278.1 (117.7 to 546.1)
	Deaths	Rate	4.2 (2.7 to 6)	8.4 (5.4 to 12.4)	0.3 (0.2 to 0.4)	5.1 (4.2 to 6.1)	10.1 (8.3 to 12.1)	0.2 (0.1 to 0.3)	22.7 (-20.6 to 93.8)	19.3 (-23.8 to 90.3)	-30.7 (-60.9 to 31.8)
		Number	17 (11 to 24)	16 (10 to 24)	1 (0 to 1)	68 (55 to 81)	67 (54 to 80)	1 (1 to 2)	298.5 (163 to 558.7)	306.2 (163.4 to 583.1)	98.6 (11.9 to 291.9)
	DALYs	Rate	126 (78.3 to 179.7)	260.8 (160.9 to 376.2)	7.9 (4.6 to 11.8)	155.8 (128 to 185.3)	309 (253 to 368.1)	5.7 (4 to 7.8)	23.7 (-17.7 to 110.7)	18.5 (-21.9 to 104.9)	-28.7 (-59.2 to 38.2)
		Number	585 (341 to 831)	566 (325 to 815)	20 (11 to 30)	2419 (1958 to 2893)	2379 (1926 to 2848)	40 (28 to 54)	313.5 (172 to 653)	320.7 (172.6 to 670)	104 (14.1 to 306.8)
	YLLs	Rate	121.9 (75.5 to 173.9)	252.5 (154.9 to 364.4)	7.7 (4.4 to 11.5)	147.2 (119.9 to 176.4)	292.1 (236.3 to 350.7)	5.4 (3.8 to 7.4)	20.7 (-20.4 to 106.9)	15.7 (-24.5 to 100.3)	-30.4 (-60.2 to 34.7)
		Number	567 (326 to 812)	548 (316 to 792)	19 (10 to 29)	2287 (1846 to 2755)	2249 (1811 to 2711)	38 (27 to 51)	303.2 (162.1 to 633)	310.2 (162.7 to 658.2)	99.6 (11.7 to 300.4)
	YLDs	Rate	4 (2.4 to 6.2)	8.3 (4.9 to 12.9)	0.2 (0.1 to 0.4)	8.6 (5.7 to 12)	17 (11.2 to 23.9)	0.3 (0.2 to 0.5)	112.8 (42.3 to 224.5)	105.3 (35.4 to 220.1)	23.8 (-32.2 to 124)
		Number	18 (10 to 27)	17 (10 to 26)	1 (0 to 1)	132 (88 to 186)	130 (86 to 184)	2 (1 to 3)	643.5 (391.7 to 1117.1)	656.1 (394 to 1154)	255.1 (89.6 to 567.4)

Province	Measure	Metric	1990			2019			% Change (1990 to 2019)		
			Both	Female	Male	Both	Female	Male	Both	Female	Male
Ilam	Incidence	Rate	6.3 (3.9 to 9.5)	13.9 (8.5 to 21.4)	0.2 (0.1 to 0.3)	15.9 (13.1 to 19.3)	31.6 (26.1 to 38.3)	0.4 (0.3 to 0.5)	153.4 (50.1 to 329.5)	128 (33.5 to 291.1)	65.3 (-10.6 to 208.4)
		Number	12 (8 to 18)	12 (7 to 18)	0 (0 to 0)	94 (77 to 113)	93 (76 to 112)	1 (1 to 1)	674.3 (374 to 1195.8)	680.8 (373.3 to 1215.8)	323.8 (123.7 to 688.2)
	Prevalence	Rate	61.8 (44.5 to 83.1)	137.6 (99 to 187.3)	1.9 (1.4 to 2.6)	145.6 (121.8 to 173)	289.3 (242 to 343)	3.2 (2.4 to 4.4)	135.7 (62.3 to 236)	110.2 (43.4 to 203.9)	68.7 (6.6 to 172.5)
		Number	115 (82 to 154)	113 (81 to 153)	2 (1 to 3)	848 (708 to 1012)	840 (698 to 1003)	8 (6 to 11)	637.3 (407.7 to 986.4)	642.3 (407.3 to 998.9)	336.9 (161.1 to 611.3)
	Deaths	Rate	3.7 (2.4 to 5.8)	8.3 (5.1 to 13.2)	0.2 (0.1 to 0.2)	5.7 (4.7 to 6.9)	11.3 (9.3 to 13.7)	0.2 (0.1 to 0.2)	53.1 (-12.7 to 161.5)	37.1 (-22.8 to 137.1)	11.4 (-35.9 to 104.5)
		Number	6 (4 to 10)	6 (4 to 10)	0 (0 to 0)	30 (24 to 36)	29 (24 to 36)	0 (0 to 1)	357.4 (173.6 to 658.1)	361.4 (173.2 to 671.7)	186.8 (61.3 to 428.4)
	DALYs	Rate	111.9 (71.7 to 167.2)	246.4 (154.9 to 373.2)	4.1 (2.5 to 6.4)	174.4 (143.7 to 211.2)	344.5 (284 to 417.6)	4.7 (3.4 to 6.4)	55.9 (-4.5 to 154.6)	39.8 (-15.7 to 130.6)	13.2 (-34.9 to 110)
		Number	225 (144 to 329)	220 (140 to 324)	5 (3 to 7)	1036 (860 to 1245)	1023 (847 to 1234)	13 (9 to 18)	361.1 (189.5 to 645.1)	364.8 (188.1 to 657.6)	180.3 (59.9 to 417.2)
	YLLs	Rate	107.5 (68.8 to 161.1)	236.9 (148.3 to 360.5)	4 (2.4 to 6.1)	163.8 (134 to 198.8)	323.4 (264.8 to 393.6)	4.4 (3.1 to 6.1)	52.3 (-7.4 to 151.4)	36.5 (-18.6 to 126.9)	10.7 (-36.4 to 107.1)
		Number	216 (137 to 319)	212 (134 to 314)	4 (3 to 7)	973 (802 to 1179)	961 (789 to 1165)	12 (9 to 17)	349.7 (180.2 to 638.5)	353.3 (179.6 to 653.3)	174.1 (55.2 to 408.9)
	YLDs	Rate	4.3 (2.5 to 7)	9.5 (5.4 to 15.8)	0.2 (0.1 to 0.3)	10.7 (7 to 14.9)	21.1 (14 to 29.7)	0.3 (0.2 to 0.4)	146.4 (57 to 292)	121.4 (39.9 to 253.9)	74.3 (-0.4 to 201.6)
		Number	8 (5 to 14)	8 (5 to 13)	0 (0 to 0)	63 (41 to 88)	62 (41 to 88)	1 (0 to 1)	657.5 (389.6 to 1135.6)	663.6 (389.9 to 1153.3)	348.5 (146.6 to 700.5)

Province	Measure	Metric	1990			2019			% Change (1990 to 2019)		
			Both	Female	Male	Both	Female	Male	Both	Female	Male
Isfahan	Incidence	Rate	9 (5.8 to 13.6)	18.1 (11.6 to 27.5)	0.2 (0.1 to 0.3)	17.6 (13.9 to 21.8)	35.5 (27.9 to 44)	0.4 (0.2 to 0.5)	96.2 (23.4 to 223.7)	95.9 (23 to 227.6)	58.1 (-11.6 to 189.3)
		Number	193 (123 to 287)	191 (121 to 285)	2 (1 to 4)	1069 (835 to 1338)	1059 (827 to 1327)	10 (6 to 14)	454.4 (255 to 829.9)	455.9 (253.3 to 838.4)	331 (138.6 to 703.8)
	Prevalence	Rate	89.6 (66.2 to 120.8)	179.8 (132.2 to 243)	2 (1.4 to 2.8)	163.1 (133.5 to 198.4)	328.1 (268.2 to 399.2)	3.1 (2.2 to 4.4)	82 (29.1 to 161.7)	82.5 (28.9 to 163)	55.7 (-1.2 to 152.4)
		Number	1860 (1340 to 2537)	1840 (1322 to 2517)	20 (13 to 29)	9829 (7972 to 12144)	9744 (7896 to 12072)	85 (61 to 121)	428.5 (270.6 to 675.9)	429.6 (270.1 to 678.9)	328.3 (164.1 to 614.5)
	Deaths	Rate	4.7 (2.9 to 7)	9.2 (5.7 to 13.9)	0.2 (0.1 to 0.2)	5.8 (4.7 to 7.1)	11.7 (9.5 to 14.4)	0.2 (0.1 to 0.2)	24.6 (-24.2 to 112)	27 (-23.5 to 118.2)	11 (-35.4 to 101.5)
		Number	90 (56 to 134)	89 (55 to 133)	1 (1 to 2)	325 (265 to 401)	321 (260 to 396)	4 (3 to 6)	261.4 (127.1 to 532.8)	261.9 (126.3 to 538.7)	225.9 (87 to 508.8)
	DALYs	Rate	140.9 (88.3 to 206.4)	285.7 (176.2 to 422.1)	3.8 (2.3 to 5.8)	181.4 (148.7 to 222.7)	364.1 (298.1 to 448.1)	4.3 (3.1 to 6.1)	28.8 (-16.1 to 121.9)	27.5 (-17.5 to 124.9)	13.6 (-32.5 to 110)
		Number	3119 (1897 to 4605)	3077 (1853 to 4552)	42 (26 to 64)	11118 (9116 to 13664)	10995 (8974 to 13535)	123 (88 to 172)	256.4 (130.1 to 521.1)	257.3 (128.4 to 525.9)	192.8 (69.5 to 441.4)
	YLLs	Rate	134.7 (82.5 to 198.6)	273.2 (164.4 to 405.2)	3.7 (2.2 to 5.6)	169.5 (136.5 to 208.5)	340.2 (272.8 to 420.3)	4.1 (2.9 to 5.8)	25.9 (-19.6 to 119.3)	24.5 (-20.9 to 120.9)	11.3 (-34.9 to 103.2)
		Number	2986 (1800 to 4446)	2946 (1762 to 4395)	40 (25 to 61)	10393 (8364 to 12769)	10277 (8248 to 12685)	116 (82 to 164)	248 (121.6 to 516.9)	248.8 (121 to 524.8)	186.4 (64.9 to 431.5)
	YLDs	Rate	6.2 (3.6 to 9.9)	12.5 (7.2 to 20)	0.2 (0.1 to 0.3)	11.9 (7.9 to 16.9)	23.9 (15.8 to 33.9)	0.3 (0.2 to 0.4)	92.1 (24.5 to 204.9)	91.9 (23.5 to 209.8)	63.8 (-4.5 to 188)
		Number	133 (75 to 213)	131 (73 to 210)	2 (1 to 3)	726 (474 to 1031)	718 (467 to 1022)	7 (4 to 12)	446.4 (254.4 to 803.9)	447.7 (252.8 to 812.9)	344.7 (156.4 to 697.3)

Province	Measure	Metric	1990			2019			% Change (1990 to 2019)		
			Both	Female	Male	Both	Female	Male	Both	Female	Male
Kerman	Incidence	Rate	7.4 (5.1 to 10.7)	15.1 (10.2 to 21.9)	0.2 (0.1 to 0.3)	13.1 (10.7 to 16.3)	26.4 (21.4 to 33)	0.3 (0.2 to 0.4)	76.3 (13.9 to 165.1)	75.2 (12.3 to 165.6)	26.6 (-27.3 to 130.9)
		Number	72 (50 to 103)	71 (49 to 102)	1 (1 to 2)	407 (328 to 514)	404 (325 to 510)	4 (3 to 5)	468.2 (274.6 to 758.5)	471.2 (273.8 to 770.3)	260.6 (101.1 to 562.3)
	Prevalence	Rate	71.1 (53.5 to 91.3)	144.1 (108.2 to 186.4)	1.8 (1.3 to 2.4)	120.6 (101 to 146.7)	242.8 (202.7 to 295.9)	2.4 (1.8 to 3.3)	69.6 (23.8 to 124.8)	68.4 (22.5 to 123.9)	35.2 (-10.5 to 104.4)
		Number	662 (492 to 875)	654 (484 to 866)	8 (6 to 12)	3704 (3056 to 4568)	3672 (3026 to 4536)	32 (24 to 46)	459.5 (302 to 656.5)	461.5 (302.3 to 661.4)	295.8 (155.6 to 525.4)
	Deaths	Rate	4.6 (3.1 to 6.6)	9.2 (6.2 to 13.3)	0.2 (0.1 to 0.2)	5.1 (4.2 to 6.1)	10.2 (8.3 to 12.2)	0.2 (0.1 to 0.2)	11.8 (-28.5 to 69.2)	11.1 (-29.5 to 70.4)	-9 (-46.1 to 58.5)
		Number	41 (28 to 58)	40 (27 to 58)	1 (0 to 1)	142 (115 to 171)	140 (113 to 169)	2 (1 to 3)	248.5 (126.4 to 429.9)	250 (125.2 to 436.5)	161.6 (53.9 to 361.9)
	DALYs	Rate	142.2 (99 to 197.2)	289.3 (200 to 403.4)	4.3 (2.8 to 6.7)	160.4 (131.2 to 192.4)	322.3 (263 to 387)	3.9 (2.8 to 5.4)	12.8 (-24.5 to 66.6)	11.4 (-26.3 to 66.3)	-9.6 (-47.3 to 57.3)
		Number	1424 (995 to 1959)	1402 (975 to 1939)	22 (14 to 33)	5055 (4091 to 6122)	5000 (4033 to 6055)	55 (40 to 76)	254.9 (136.9 to 426.2)	256.6 (136.7 to 433.4)	150.9 (45.8 to 345)
	YLLs	Rate	137.2 (95 to 191.4)	279.2 (191.4 to 392.3)	4.2 (2.7 to 6.5)	151.6 (122.5 to 182.5)	304.7 (245.1 to 368.2)	3.7 (2.7 to 5.1)	10.5 (-27.3 to 65)	9.1 (-28.8 to 64.6)	-11.2 (-48.3 to 54.9)
		Number	1376 (956 to 1910)	1355 (937 to 1889)	21 (14 to 32)	4780 (3802 to 5788)	4728 (3753 to 5735)	52 (38 to 73)	247.4 (131.6 to 421.5)	249 (131.1 to 424.8)	146.3 (43.6 to 341.3)
	YLDs	Rate	5 (3 to 7.8)	10.1 (6 to 16)	0.2 (0.1 to 0.2)	8.8 (5.8 to 12.4)	17.7 (11.7 to 25.1)	0.2 (0.1 to 0.3)	75.8 (17.5 to 158.9)	74.7 (16 to 160.4)	35.2 (-18.8 to 127.6)
		Number	48 (29 to 75)	47 (28 to 74)	1 (0 to 1)	275 (181 to 393)	272 (179 to 390)	3 (2 to 5)	471.3 (282.1 to 760.1)	474.1 (280 to 770.5)	286.7 (127.5 to 572.4)

Province	Measure	Metric	1990			2019			% Change (1990 to 2019)		
			Both	Female	Male	Both	Female	Male	Both	Female	Male
Kermanshah	Incidence	Rate	7.5 (5.1 to 10.6)	15.7 (10.6 to 22.6)	0.2 (0.1 to 0.3)	15.2 (12.1 to 18.9)	29.8 (23.6 to 37.3)	0.3 (0.2 to 0.4)	103.3 (34.7 to 221)	89.8 (23.3 to 203)	28 (-27.7 to 124.9)
		Number	66 (44 to 93)	65 (43 to 92)	1 (1 to 2)	330 (262 to 413)	327 (259 to 410)	3 (2 to 4)	401.4 (231.6 to 700.5)	404.7 (231.9 to 712.4)	183.5 (57.1 to 409)
	Prevalence	Rate	69.7 (53.2 to 89.9)	147.9 (113.2 to 191.7)	1.8 (1.3 to 2.6)	137.9 (112.6 to 166.5)	270.5 (221 to 327)	2.6 (1.9 to 3.5)	97.8 (46.5 to 179.6)	82.9 (34.7 to 159.4)	39.4 (-9.5 to 108.2)
		Number	594 (438 to 782)	586 (430 to 775)	8 (5 to 11)	2978 (2430 to 3635)	2953 (2406 to 3607)	25 (18 to 35)	401.1 (261.9 to 624.8)	403.6 (263 to 634)	212.3 (98.2 to 398.4)
	Deaths	Rate	4.6 (3.2 to 6.7)	9.8 (6.5 to 14.4)	0.2 (0.1 to 0.3)	5.8 (4.7 to 7)	11.4 (9.1 to 13.8)	0.2 (0.1 to 0.2)	25.5 (-19 to 97.7)	16.6 (-25.5 to 84.9)	-13.1 (-48 to 61.1)
		Number	37 (25 to 53)	36 (25 to 52)	1 (0 to 1)	117 (94 to 142)	115 (92 to 141)	1 (1 to 2)	214.4 (106.5 to 389.1)	216.5 (106.6 to 395.6)	103 (19.1 to 275.7)
	DALYs	Rate	144.9 (98.3 to 202.1)	304 (205.1 to 428.9)	4.6 (2.8 to 7)	181.1 (143.8 to 220.1)	354.4 (280.8 to 430.3)	4.1 (3 to 5.4)	24.9 (-15.7 to 92.7)	16.6 (-22.6 to 79.2)	-11.3 (-47.9 to 63.8)
		Number	1331 (902 to 1859)	1309 (874 to 1832)	22 (14 to 34)	3986 (3139 to 4858)	3945 (3103 to 4811)	41 (30 to 55)	199.5 (101.9 to 365.8)	201.3 (102.4 to 370)	88.2 (9.7 to 247)
	YLLs	Rate	140 (94.8 to 194.9)	293.6 (198.5 to 414)	4.4 (2.7 to 6.8)	170.9 (135.6 to 209.1)	334.5 (263.8 to 410.4)	3.8 (2.8 to 5.2)	22.1 (-19.2 to 89.8)	13.9 (-24.8 to 76.5)	-13.1 (-49.6 to 61.9)
		Number	1287 (869 to 1803)	1266 (842 to 1784)	21 (13 to 33)	3764 (2973 to 4622)	3725 (2929 to 4575)	39 (28 to 53)	192.4 (94.4 to 355)	194.2 (94.7 to 359.1)	84.2 (6.8 to 241.9)
	YLDs	Rate	5 (3 to 7.7)	10.4 (6.4 to 16.1)	0.2 (0.1 to 0.3)	10.2 (6.5 to 14.8)	19.9 (12.7 to 29.1)	0.2 (0.1 to 0.4)	104.8 (40.2 to 210.8)	90.7 (29.7 to 191.4)	39.2 (-16.3 to 130.2)
		Number	44 (26 to 68)	43 (26 to 67)	1 (0 to 1)	221 (140 to 326)	219 (138 to 324)	2 (1 to 3)	406.5 (239.7 to 695.7)	409.8 (239.6 to 708.9)	206.6 (78.9 to 431.1)

Province	Measure	Metric	1990			2019			% Change (1990 to 2019)		
			Both	Female	Male	Both	Female	Male	Both	Female	Male
Khorasan-e-Razavi	Incidence	Rate	7.4 (5 to 10.2)	15.1 (10.1 to 20.9)	0.2 (0.1 to 0.3)	15 (12.1 to 18.4)	29.7 (23.8 to 36.5)	0.2 (0.2 to 0.3)	103 (34 to 216.1)	96.5 (28.8 to 207.2)	27.5 (-26.7 to 122.9)
		Number	190 (127 to 258)	188 (125 to 256)	2 (1 to 3)	988 (785 to 1225)	982 (780 to 1217)	6 (4 to 9)	418.9 (245.4 to 698.1)	421.5 (245.5 to 703.3)	187.5 (64.2 to 404.9)
	Prevalence	Rate	70.1 (52.6 to 89.9)	143.2 (106.9 to 184.5)	1.5 (1.1 to 2.2)	136.1 (112.5 to 164.1)	268.4 (221.4 to 324.3)	2.1 (1.5 to 2.8)	94 (43.3 to 165.4)	87.3 (37.5 to 156.9)	33.7 (-8.4 to 100.4)
		Number	1736 (1256 to 2242)	1717 (1240 to 2221)	18 (13 to 27)	8862 (7273 to 10770)	8805 (7219 to 10713)	57 (42 to 79)	410.6 (266.4 to 612.7)	412.7 (267 to 617.4)	211.7 (103.2 to 381.3)
	Deaths	Rate	4.6 (3.1 to 6.6)	9.4 (6.3 to 13.5)	0.1 (0.1 to 0.2)	5.6 (4.7 to 6.8)	11 (9.2 to 13.3)	0.1 (0.1 to 0.2)	21.7 (-19.7 to 88)	17.4 (-23.3 to 82.4)	-11.9 (-47.4 to 40.3)
		Number	110 (73 to 156)	108 (72 to 154)	1 (1 to 2)	337 (279 to 408)	333 (276 to 404)	3 (2 to 4)	206.1 (101.4 to 366.1)	207.5 (101.9 to 370)	106.2 (21.4 to 232.6)
	DALYs	Rate	145.3 (97.3 to 202.3)	296.6 (197.5 to 415.5)	3.5 (2.3 to 5.2)	177.7 (147.7 to 215.8)	350.8 (290.9 to 426.1)	3 (2.1 to 4)	22.4 (-17.7 to 85.4)	18.3 (-20.8 to 80.6)	-13.2 (-49.4 to 39.1)
		Number	3884 (2564 to 5442)	3838 (2517 to 5408)	46 (30 to 71)	11814 (9745 to 14355)	11728 (9654 to 14270)	87 (62 to 118)	204.2 (105.5 to 369.9)	205.6 (105.5 to 373.8)	87.6 (7.9 to 209.6)
	YLLs	Rate	140.3 (92.9 to 197.1)	286.5 (189 to 403.7)	3.3 (2.2 to 5)	167.8 (138.5 to 204.6)	331.1 (271.9 to 404.5)	2.8 (2 to 3.8)	19.6 (-20.2 to 84.1)	15.6 (-23.4 to 78.4)	-15.1 (-50.9 to 36.2)
		Number	3757 (2465 to 5282)	3713 (2421 to 5249)	45 (29 to 69)	11154 (9111 to 13634)	11072 (9021 to 13559)	82 (58 to 112)	196.9 (98.3 to 364.2)	198.2 (97.9 to 369.8)	83.3 (4.5 to 203.1)
	YLDs	Rate	4.9 (3 to 7.6)	10.1 (6.1 to 15.6)	0.1 (0.1 to 0.2)	10 (6.7 to 14.5)	19.7 (13.1 to 28.6)	0.2 (0.1 to 0.3)	101.9 (41.8 to 194.1)	95.4 (36.8 to 186.2)	37.2 (-15.2 to 122.6)
		Number	127 (75 to 198)	125 (74 to 196)	2 (1 to 3)	660 (434 to 952)	655 (429 to 948)	5 (3 to 8)	421.3 (263.3 to 682)	423.9 (264.4 to 688.3)	211 (86 to 421.8)

Province	Measure	Metric	1990			2019			% Change (1990 to 2019)		
			Both	Female	Male	Both	Female	Male	Both	Female	Male
Khuzestan	Incidence	Rate	7.9 (5.4 to 11.2)	15.8 (10.9 to 22.8)	0.3 (0.2 to 0.4)	17.2 (13.5 to 21.3)	34.4 (27 to 42.7)	0.3 (0.2 to 0.4)	118.3 (37.3 to 242.3)	116.9 (35.5 to 238.8)	10.8 (-39.6 to 92.6)
		Number	120 (83 to 170)	118 (81 to 169)	2 (1 to 3)	791 (618 to 988)	786 (613 to 982)	5 (4 to 8)	558.1 (319.5 to 936.9)	563.6 (320.4 to 963.4)	199.6 (66.7 to 422)
	Prevalence	Rate	76.5 (58.3 to 99.3)	153.6 (116.4 to 200.9)	2.1 (1.5 to 2.9)	155.5 (126.7 to 187.7)	310.3 (251.9 to 375.8)	2.5 (1.8 to 3.4)	103.2 (43.6 to 179.8)	102 (42 to 180.4)	22.3 (-22.9 to 83.9)
		Number	1115 (836 to 1464)	1101 (822 to 1447)	15 (10 to 21)	7066 (5714 to 8650)	7018 (5664 to 8601)	49 (35 to 67)	533.5 (342.4 to 792.1)	537.5 (343.6 to 805.1)	232.3 (107.5 to 418.2)
	Deaths	Rate	4.6 (3.2 to 6.6)	9.1 (6.3 to 13)	0.2 (0.1 to 0.3)	6.2 (5 to 7.5)	12.2 (10 to 14.9)	0.1 (0.1 to 0.2)	34.4 (-14.6 to 103.5)	34.5 (-15 to 104.3)	-23.2 (-54.8 to 32.5)
		Number	64 (45 to 89)	63 (44 to 88)	1 (1 to 2)	254 (206 to 310)	251 (204 to 308)	3 (2 to 4)	297.5 (155.8 to 492.3)	301.1 (156.9 to 501.3)	112.3 (23.9 to 273.8)
	DALYs	Rate	145.9 (104.2 to 203.9)	295.1 (209.5 to 414.3)	5 (3.1 to 7.5)	199.4 (162.8 to 241.8)	398 (324.6 to 484.1)	3.8 (2.7 to 5.3)	36.7 (-10.7 to 99.8)	34.9 (-12.5 to 98.8)	-23.4 (-55.3 to 33)
		Number	2309 (1647 to 3253)	2270 (1612 to 3203)	39 (24 to 57)	9264 (7557 to 11257)	9186 (7464 to 11182)	78 (56 to 109)	301.2 (163.5 to 487.7)	304.7 (164.7 to 498.2)	100.3 (17.9 to 257.3)
	YLLs	Rate	140.5 (99.3 to 197.9)	284.4 (198.8 to 400.5)	4.8 (3 to 7.3)	187.9 (152.3 to 230.1)	375.2 (302.4 to 460.8)	3.6 (2.6 to 5.1)	33.7 (-13.5 to 96.9)	31.9 (-15.1 to 95.7)	-25 (-56.6 to 30.8)
		Number	2228 (1572 to 3127)	2190 (1533 to 3079)	38 (23 to 56)	8734 (7049 to 10722)	8661 (6969 to 10659)	74 (52 to 104)	292.1 (154.2 to 482.9)	295.4 (155.7 to 493)	96 (14.6 to 253.8)
	YLDs	Rate	5.3 (3.3 to 8.4)	10.7 (6.5 to 16.9)	0.2 (0.1 to 0.3)	11.5 (7.5 to 16.3)	22.9 (14.9 to 32.5)	0.2 (0.1 to 0.4)	115.1 (38.9 to 221.4)	113.7 (37.4 to 221.7)	21.6 (-30.8 to 97.1)
		Number	81 (49 to 129)	80 (48 to 127)	1 (1 to 2)	530 (345 to 758)	526 (342 to 752)	4 (3 to 7)	553.5 (325.8 to 893.5)	558.8 (327.1 to 916.4)	226.7 (87.3 to 447.2)

Province	Measure	Metric	1990			2019			% Change (1990 to 2019)		
			Both	Female	Male	Both	Female	Male	Both	Female	Male
Kohgiluyeh and Boyer-Ahmad	Incidence	Rate	5.1 (3.3 to 7.9)	10.4 (6.6 to 16.2)	0.4 (0.2 to 0.6)	11.8 (8.9 to 15.1)	24.1 (18.2 to 31)	0.4 (0.3 to 0.6)	131.1 (32.2 to 300.2)	131.8 (30.5 to 310.2)	6 (-46 to 104.6)
		Number	10 (7 to 15)	10 (6 to 15)	0 (0 to 1)	78 (58 to 100)	77 (56 to 99)	1 (1 to 2)	668.6 (345.5 to 1225.4)	688.4 (346.3 to 1281.5)	187.7 (42.5 to 474.4)
	Prevalence	Rate	55.8 (41.6 to 75)	114 (84.6 to 153.2)	3 (2.1 to 4.2)	111.7 (88.4 to 137.1)	227.5 (179.3 to 280.1)	3.6 (2.6 to 5.1)	100.3 (35.6 to 181.6)	99.6 (34.5 to 184.8)	22.9 (-27.7 to 106.5)
		Number	104 (76 to 140)	101 (73 to 137)	3 (2 to 5)	720 (557 to 901)	709 (548 to 891)	11 (8 to 14)	593 (359.4 to 913.3)	604.2 (363 to 935.9)	236.1 (87.6 to 479.4)
	Deaths	Rate	3.2 (2.1 to 5.1)	6.4 (4 to 10.2)	0.3 (0.2 to 0.4)	4.1 (3.2 to 5.2)	8.5 (6.7 to 10.9)	0.2 (0.1 to 0.3)	28.5 (-26.2 to 116.1)	33.8 (-23.9 to 128.6)	-30.9 (-63.1 to 25.4)
		Number	5 (4 to 8)	5 (3 to 8)	0 (0 to 0)	23 (18 to 30)	23 (18 to 29)	0 (0 to 1)	324.8 (153.7 to 592)	335.9 (156.7 to 635.8)	96 (4.5 to 283.2)
	DALYs	Rate	90.9 (61.1 to 137.4)	186.5 (122.4 to 285.1)	7.4 (4.5 to 11.6)	125.4 (98.4 to 159.9)	253.3 (198.6 to 323.2)	5.1 (3.5 to 7.1)	38 (-15.9 to 122)	35.8 (-19.6 to 124.7)	-31.4 (-63.3 to 24.9)
		Number	192 (128 to 284)	183 (120 to 274)	9 (5 to 14)	831 (647 to 1064)	815 (633 to 1050)	16 (11 to 22)	333.5 (168.3 to 600.6)	346.1 (172.4 to 633.9)	75.5 (-5.7 to 222.8)
	YLLs	Rate	87.2 (58 to 132.6)	179 (116.3 to 275.4)	7.1 (4.3 to 11.2)	117.3 (91.5 to 151.4)	236.8 (183.6 to 306.6)	4.7 (3.2 to 6.8)	34.5 (-18.8 to 119.2)	32.3 (-21.8 to 121.6)	-33.2 (-64.9 to 24.9)
		Number	185 (122 to 272)	176 (114 to 265)	9 (5 to 14)	777 (596 to 1005)	763 (581 to 987)	15 (10 to 21)	321.4 (159.2 to 587.5)	333.7 (163 to 623.4)	70.8 (-8.1 to 218.7)
	YLDs	Rate	3.7 (2.2 to 5.9)	7.5 (4.4 to 12.1)	0.3 (0.1 to 0.4)	8.1 (5.1 to 11.6)	16.5 (10.5 to 23.5)	0.3 (0.2 to 0.5)	121.1 (35.6 to 246.8)	120.6 (33.6 to 252.4)	18.6 (-36.2 to 119.4)
		Number	7 (4 to 11)	7 (4 to 11)	0 (0 to 0)	54 (34 to 79)	53 (33 to 78)	1 (1 to 1)	643 (353.8 to 1101.4)	660.1 (354.7 to 1138.1)	220.9 (67.3 to 503.1)

Province	Measure	Metric	1990			2019			% Change (1990 to 2019)		
			Both	Female	Male	Both	Female	Male	Both	Female	Male
Kurdistan	Incidence	Rate	7.4 (5.2 to 10.4)	15.6 (10.8 to 22.3)	0.2 (0.1 to 0.3)	13.7 (11.2 to 16.7)	27.3 (22.1 to 33.2)	0.2 (0.2 to 0.4)	86.1 (26.6 to 173.4)	75.2 (17.3 to 160.3)	16.2 (-34.7 to 113.7)
		Number	48 (34 to 67)	47 (33 to 66)	1 (0 to 1)	230 (185 to 281)	228 (183 to 280)	2 (1 to 3)	383.1 (226.1 to 605.6)	386 (226.1 to 613.5)	173.4 (52.7 to 412.1)
	Prevalence	Rate	69 (52.7 to 89.3)	146.8 (111.9 to 191.6)	1.7 (1.2 to 2.5)	124.9 (103.4 to 148.4)	248.4 (205.4 to 294.3)	2.2 (1.6 to 3.1)	81 (36.7 to 140.9)	69.2 (26.8 to 126.6)	27.3 (-15.4 to 96.8)
		Number	434 (330 to 573)	428 (324 to 567)	5 (4 to 8)	2084 (1711 to 2486)	2067 (1695 to 2468)	17 (12 to 23)	380.4 (250.2 to 549.8)	382.6 (250.9 to 555.1)	203.7 (97.6 to 380)
	Deaths	Rate	4.6 (3.3 to 6.4)	9.7 (6.8 to 13.8)	0.2 (0.1 to 0.2)	5.4 (4.5 to 6.5)	10.8 (8.9 to 12.9)	0.1 (0.1 to 0.2)	18.8 (-21.4 to 74.7)	11.3 (-26.9 to 65.5)	-19.9 (-54.2 to 36.6)
		Number	27 (20 to 37)	27 (19 to 37)	0 (0 to 1)	83 (69 to 100)	83 (68 to 99)	1 (1 to 1)	206.3 (107.1 to 342.7)	208.2 (107 to 348.6)	96.8 (12.4 to 241.6)
	DALYs	Rate	140.9 (103.1 to 192.6)	296.6 (214.7 to 406.3)	4 (2.7 to 6)	162.4 (134.7 to 195.7)	322.1 (266.4 to 388.3)	3.3 (2.3 to 4.5)	15.2 (-21.3 to 63.2)	8.6 (-26.6 to 55.3)	-18.2 (-53 to 41.5)
		Number	948 (693 to 1293)	934 (680 to 1282)	14 (9 to 21)	2759 (2278 to 3332)	2734 (2253 to 3310)	26 (18 to 35)	191 (98 to 313.2)	192.5 (98.2 to 319.2)	84.6 (7 to 216.5)
	YLLs	Rate	136 (98.4 to 186.1)	286.3 (205.4 to 391.8)	3.9 (2.5 to 5.8)	153.3 (125.3 to 185.8)	304 (248.1 to 369.3)	3.1 (2.2 to 4.3)	12.7 (-24.2 to 61.4)	6.2 (-28.9 to 53.7)	-19.9 (-53.9 to 40.4)
		Number	917 (667 to 1260)	903 (653 to 1249)	14 (9 to 20)	2605 (2123 to 3170)	2581 (2100 to 3142)	24 (17 to 33)	184.2 (92.4 to 305.6)	185.7 (93.2 to 309.6)	80.6 (3.9 to 209.1)
	YLDs	Rate	4.9 (3 to 7.5)	10.3 (6.3 to 15.8)	0.1 (0.1 to 0.2)	9.1 (5.8 to 12.7)	18.1 (11.6 to 25.3)	0.2 (0.1 to 0.3)	87.1 (30.4 to 168.7)	76 (22.1 to 156.7)	27.2 (-24.1 to 115.3)
		Number	32 (20 to 49)	31 (19 to 48)	0 (0 to 1)	154 (99 to 216)	153 (98 to 214)	1 (1 to 2)	388.2 (239.1 to 596.8)	391 (238.4 to 606.5)	198.6 (75.6 to 418.7)

Province	Measure	Metric	1990			2019			% Change (1990 to 2019)		
			Both	Female	Male	Both	Female	Male	Both	Female	Male
Lorestan	Incidence	Rate	7.2 (5 to 10.4)	15.2 (10.4 to 21.9)	0.3 (0.2 to 0.4)	13.3 (10.1 to 16.8)	26 (19.8 to 33)	0.3 (0.2 to 0.5)	84 (14.8 to 177.6)	71.2 (4.8 to 159.4)	14.9 (-38 to 118.2)
		Number	53 (37 to 75)	52 (36 to 74)	1 (1 to 2)	239 (182 to 303)	237 (180 to 301)	2 (2 to 4)	354.1 (188.7 to 588)	358.2 (188.6 to 600.1)	138.1 (26.7 to 369.9)
	Prevalence	Rate	69 (52.8 to 90.2)	145.6 (110.8 to 191.6)	2.1 (1.5 to 3)	126 (100.4 to 154.5)	245.6 (195.5 to 301.1)	2.8 (2 to 3.9)	82.5 (27.9 to 145.9)	68.7 (17.9 to 127.2)	28.4 (-20.3 to 107.3)
		Number	486 (368 to 647)	478 (361 to 640)	8 (5 to 11)	2227 (1760 to 2735)	2206 (1737 to 2713)	21 (15 to 30)	358.1 (220.4 to 525.3)	361.2 (221.6 to 530.9)	170.1 (62.2 to 366)
	Deaths	Rate	4.4 (3 to 6.4)	9.3 (6.1 to 13.6)	0.2 (0.1 to 0.3)	4.7 (3.6 to 6)	9.2 (7 to 11.5)	0.2 (0.1 to 0.2)	6.8 (-34.8 to 66.3)	-1.2 (-39.7 to 56.2)	-23.5 (-56.7 to 37.5)
		Number	29 (20 to 42)	29 (19 to 42)	1 (0 to 1)	78 (59 to 98)	77 (58 to 97)	1 (1 to 2)	164.2 (67.4 to 317.5)	166.5 (67.6 to 324.6)	63.7 (-9.6 to 192.5)
	DALYs	Rate	133.9 (94.4 to 189.4)	281.3 (196 to 401.8)	5.2 (3.3 to 7.9)	146.6 (113.2 to 184.5)	285.6 (220 to 359.2)	3.9 (2.7 to 5.5)	9.5 (-29.9 to 71.2)	1.5 (-35.3 to 60.5)	-24.7 (-57.9 to 32.4)
		Number	1017 (715 to 1437)	996 (692 to 1418)	21 (13 to 32)	2649 (2055 to 3343)	2618 (2027 to 3316)	32 (22 to 45)	160.5 (68 to 307)	162.9 (68 to 315.3)	49.8 (-18.2 to 165.1)
	YLLs	Rate	129 (90 to 182.7)	271.1 (186.9 to 386.2)	5 (3.2 to 7.7)	137.5 (104.4 to 173.9)	267.9 (203.7 to 339.4)	3.7 (2.5 to 5.2)	6.6 (-32.3 to 69)	-1.2 (-37.8 to 58.5)	-26.6 (-59.4 to 29.6)
		Number	982 (689 to 1396)	961 (665 to 1376)	20 (13 to 31)	2486 (1898 to 3169)	2456 (1864 to 3144)	30 (20 to 42)	153.3 (61.3 to 301.2)	155.5 (61.5 to 310.4)	45.9 (-20.2 to 160.8)
	YLDs	Rate	4.8 (2.9 to 7.5)	10.2 (6.2 to 15.8)	0.2 (0.1 to 0.3)	9.1 (5.9 to 13.1)	17.7 (11.4 to 25.5)	0.2 (0.1 to 0.4)	87.7 (23.5 to 174.7)	74.5 (13.5 to 155.6)	27.2 (-28.4 to 125.9)
		Number	35 (21 to 55)	35 (21 to 54)	1 (0 to 1)	163 (105 to 236)	161 (104 to 234)	2 (1 to 3)	363.2 (202.3 to 584.6)	367.2 (203.3 to 593.3)	163.9 (48 to 397.3)

Province	Measure	Metric	1990			2019			% Change (1990 to 2019)		
			Both	Female	Male	Both	Female	Male	Both	Female	Male
Markazi	Incidence	Rate	8.9 (6.1 to 12.3)	17.5 (12 to 24.5)	0.2 (0.1 to 0.4)	15.4 (12.4 to 19.1)	30.7 (24.6 to 37.9)	0.3 (0.2 to 0.4)	73.9 (17.3 to 160.7)	74.9 (17.7 to 163.8)	29.2 (-24.8 to 125)
		Number	62 (42 to 85)	61 (42 to 84)	1 (0 to 1)	255 (205 to 315)	253 (203 to 313)	2 (1 to 3)	313.1 (177.1 to 520.8)	314.6 (177 to 525.9)	188.9 (62.2 to 414.7)
	Prevalence	Rate	83.1 (63.4 to 105.7)	164.8 (124.9 to 210.8)	1.9 (1.4 to 2.7)	142.3 (118.2 to 169.8)	282 (233.4 to 337.5)	2.6 (1.9 to 3.6)	71.2 (27.3 to 128.9)	71.1 (26.6 to 130.1)	37.3 (-9.3 to 106)
		Number	567 (428 to 726)	561 (421 to 720)	6 (4 to 9)	2329 (1917 to 2810)	2309 (1899 to 2791)	20 (14 to 27)	310.6 (198.9 to 460.5)	311.6 (199 to 464)	214.4 (100.7 to 390)
	Deaths	Rate	5.2 (3.7 to 7.5)	10.3 (7.2 to 14.9)	0.2 (0.1 to 0.2)	5.7 (4.7 to 6.9)	11.2 (9.2 to 13.5)	0.1 (0.1 to 0.2)	8.9 (-28.1 to 59.4)	8.8 (-28.5 to 60.8)	-11.2 (-46.6 to 44.7)
		Number	34 (24 to 47)	33 (23 to 47)	1 (0 to 1)	90 (73 to 108)	89 (73 to 107)	1 (1 to 1)	166.3 (75.6 to 283.2)	167 (75.7 to 286.2)	114.9 (27.1 to 256.8)
	DALYs	Rate	161.6 (115 to 222.1)	318.8 (225.1 to 441)	4.3 (2.8 to 6.5)	175.2 (143.2 to 211)	347.7 (283.3 to 418.9)	3.9 (2.8 to 5.3)	8.4 (-26.1 to 55.4)	9.1 (-26.2 to 56.7)	-10.3 (-45.8 to 50.9)
		Number	1163 (825 to 1605)	1148 (811 to 1585)	16 (10 to 24)	2906 (2368 to 3517)	2876 (2334 to 3480)	30 (22 to 42)	149.8 (71.8 to 258.2)	150.6 (71.7 to 261.2)	92 (15.8 to 221.3)
	YLLs	Rate	155.7 (109.7 to 215.4)	307.2 (215.8 to 427.6)	4.2 (2.6 to 6.3)	164.8 (134 to 199.1)	327.1 (265.1 to 394.9)	3.7 (2.7 to 5.1)	5.9 (-28.3 to 51.9)	6.5 (-28.2 to 54.2)	-12.1 (-47.8 to 49)
		Number	1122 (796 to 1551)	1107 (777 to 1534)	15 (10 to 23)	2735 (2211 to 3328)	2706 (2181 to 3299)	29 (21 to 40)	143.7 (66.6 to 252.5)	144.5 (65.7 to 256.4)	87.8 (11.4 to 218)
	YLDs	Rate	5.9 (3.5 to 8.8)	11.6 (6.9 to 17.5)	0.2 (0.1 to 0.3)	10.4 (6.8 to 14.7)	20.5 (13.4 to 29.2)	0.2 (0.1 to 0.4)	75.6 (21 to 150.7)	76.2 (21.1 to 153.6)	38.7 (-17.2 to 130.4)
		Number	41 (24 to 63)	41 (24 to 62)	1 (0 to 1)	171 (113 to 246)	169 (111 to 244)	2 (1 to 3)	316.2 (187 to 505.9)	317.6 (187.3 to 513)	210.7 (83.5 to 426.9)

Province	Measure	Metric	1990			2019			% Change (1990 to 2019)		
			Both	Female	Male	Both	Female	Male	Both	Female	Male
Mazandaran	Incidence	Rate	9.8 (6.6 to 14.7)	19.5 (13.1 to 29.1)	0.2 (0.1 to 0.3)	20.8 (16.5 to 25.8)	41.2 (32.6 to 51.1)	0.3 (0.2 to 0.4)	112.1 (31.1 to 241)	111.8 (30.4 to 242)	31.5 (-26.4 to 137.6)
		Number	146 (99 to 215)	144 (98 to 214)	2 (1 to 2)	881 (698 to 1098)	875 (693 to 1091)	5 (4 to 8)	504.3 (273.5 to 869.9)	506.8 (273.5 to 879)	264.2 (97.9 to 572.8)
	Prevalence	Rate	96.2 (71.8 to 129.9)	190.3 (141.6 to 258.3)	2 (1.4 to 2.9)	191.4 (156.2 to 232.6)	378.5 (308.3 to 460.9)	2.7 (2 to 3.8)	99 (36.9 to 183.5)	98.9 (36.8 to 184.1)	34.6 (-13.7 to 107.9)
		Number	1372 (1009 to 1876)	1359 (996 to 1862)	13 (9 to 19)	8044 (6514 to 9843)	7993 (6463 to 9787)	51 (36 to 73)	486.2 (292.9 to 753.6)	488.2 (293.1 to 755.6)	277 (133.5 to 512)
	Deaths	Rate	4.7 (3.2 to 7.2)	9.3 (6.2 to 14.2)	0.1 (0.1 to 0.2)	6.2 (5.1 to 7.6)	12.1 (9.9 to 14.9)	0.1 (0.1 to 0.2)	30.6 (-20 to 102.6)	31 (-20.1 to 105.1)	-8.7 (-46.9 to 60.2)
		Number	63 (43 to 95)	62 (42 to 94)	1 (1 to 1)	247 (201 to 304)	245 (198 to 302)	2 (2 to 3)	292.8 (146.5 to 509.1)	294.5 (146.7 to 516.6)	175.2 (56 to 388.6)
	DALYs	Rate	150.1 (104 to 222.1)	298.4 (206.3 to 443.5)	3.8 (2.3 to 5.9)	204.3 (167.1 to 250.6)	403.7 (329.8 to 496)	3.4 (2.4 to 4.7)	36.1 (-13 to 107.1)	35.3 (-14.3 to 106.7)	-8.8 (-46.5 to 59.3)
		Number	2291 (1580 to 3356)	2263 (1559 to 3325)	28 (17 to 43)	8721 (7133 to 10699)	8655 (7063 to 10624)	66 (47 to 91)	280.7 (146.4 to 477.7)	282.5 (146.2 to 483.5)	138 (36.9 to 317)
	YLLs	Rate	143.4 (98.2 to 212.6)	285.2 (193.5 to 426.3)	3.6 (2.2 to 5.7)	190.3 (153.8 to 234.5)	376.1 (303.5 to 464.1)	3.2 (2.3 to 4.5)	32.7 (-16.2 to 104.2)	31.9 (-17.2 to 104.2)	-11 (-48.2 to 58.1)
		Number	2192 (1494 to 3216)	2165 (1466 to 3191)	27 (16 to 42)	8131 (6551 to 10036)	8069 (6498 to 9969)	62 (43 to 86)	271 (134.2 to 469.3)	272.7 (134.1 to 475.5)	131.8 (31 to 308.2)
	YLDs	Rate	6.7 (4 to 10.9)	13.2 (7.8 to 21.7)	0.2 (0.1 to 0.3)	13.9 (9.1 to 19.8)	27.6 (17.9 to 39.3)	0.2 (0.1 to 0.4)	108.2 (33.3 to 220.1)	108.1 (32.5 to 223.7)	38.4 (-18.3 to 134.1)
		Number	99 (58 to 164)	98 (57 to 162)	1 (1 to 2)	590 (383 to 846)	586 (380 to 841)	4 (3 to 7)	496.1 (275.6 to 839)	498.6 (275 to 846.3)	281.4 (116.6 to 570.9)

Province	Measure	Metric	1990			2019			% Change (1990 to 2019)		
			Both	Female	Male	Both	Female	Male	Both	Female	Male
North Khorasan	Incidence	Rate	6.7 (4.7 to 9.3)	13.5 (9.2 to 18.9)	0.3 (0.2 to 0.5)	13.6 (11.2 to 16.6)	26.6 (21.9 to 32.6)	0.4 (0.2 to 0.5)	102.3 (32.3 to 195.2)	96.6 (27.9 to 192.1)	0.7 (-41.1 to 76.4)
		Number	23 (16 to 31)	22 (15 to 31)	1 (0 to 1)	115 (95 to 140)	114 (93 to 139)	1 (1 to 2)	407.3 (240.9 to 634.9)	414.6 (245.8 to 654.1)	126.6 (31.4 to 305)
	Prevalence	Rate	63.5 (49.5 to 80.6)	129.1 (100.3 to 164.8)	2.5 (1.7 to 3.5)	122.8 (103.2 to 146.5)	240.3 (202.2 to 285.8)	3 (2.3 to 4)	93.3 (44.9 to 153)	86.1 (38.7 to 145.9)	19.7 (-22.1 to 84.8)
		Number	204 (159 to 264)	200 (154 to 260)	4 (3 to 6)	1032 (867 to 1230)	1021 (856 to 1219)	11 (8 to 15)	405.8 (271.6 to 567.8)	410.6 (273 to 580.3)	171.9 (72.2 to 336)
	Deaths	Rate	4.5 (3.1 to 6.2)	9 (6.1 to 12.6)	0.3 (0.2 to 0.4)	5.4 (4.4 to 6.4)	10.5 (8.7 to 12.6)	0.2 (0.1 to 0.3)	19.6 (-22.3 to 80)	16.7 (-24.6 to 78.1)	-31 (-59.9 to 19.2)
		Number	14 (10 to 19)	14 (9 to 18)	0 (0 to 1)	42 (35 to 51)	42 (34 to 50)	1 (1 to 1)	203.8 (103.6 to 345.8)	208.1 (106.5 to 357.6)	63.4 (-5.2 to 185.9)
	DALYs	Rate	141 (100.1 to 186.4)	283.6 (197.7 to 379.1)	7.5 (4.8 to 11.3)	171.8 (142.9 to 205.4)	334.8 (278.1 to 400.4)	5.2 (3.8 to 7.1)	21.8 (-16.9 to 77)	18 (-20.4 to 76)	-31.7 (-60.2 to 18.1)
		Number	501 (359 to 664)	488 (346 to 649)	14 (9 to 20)	1474 (1226 to 1757)	1454 (1208 to 1737)	20 (15 to 27)	193.9 (103.6 to 321.2)	198 (103.6 to 337.3)	46.3 (-14.7 to 155.1)
	YLLs	Rate	136.5 (96.2 to 181.4)	274.7 (190.1 to 367.9)	7.3 (4.6 to 10.9)	162.7 (134.3 to 196)	317 (260.7 to 380.3)	4.9 (3.6 to 6.7)	19.2 (-19.4 to 74.2)	15.4 (-23 to 72.6)	-33.2 (-61 to 16.3)
		Number	487 (346 to 647)	473 (332 to 635)	13 (8 to 20)	1397 (1151 to 1679)	1378 (1135 to 1659)	19 (14 to 26)	187 (96.4 to 319.2)	191.1 (96.9 to 331.7)	43.1 (-17.1 to 150)
	YLDs	Rate	4.4 (2.8 to 6.7)	8.9 (5.6 to 13.7)	0.2 (0.1 to 0.4)	9.1 (6.1 to 12.7)	17.7 (11.8 to 24.8)	0.3 (0.2 to 0.4)	104.8 (42.5 to 187.2)	98.6 (36.8 to 183.8)	14.8 (-31.4 to 88.4)
		Number	15 (9 to 23)	14 (9 to 22)	0 (0 to 1)	77 (51 to 109)	76 (51 to 108)	1 (1 to 2)	419.2 (262.4 to 641.3)	426.2 (265.6 to 653.2)	156.9 (53.1 to 341.3)

Province	Measure	Metric	1990			2019			% Change (1990 to 2019)		
			Both	Female	Male	Both	Female	Male	Both	Female	Male
Qazvin	Incidence	Rate	7.1 (4.7 to 10.2)	14.2 (9.3 to 20.5)	0.2 (0.2 to 0.4)	15.4 (12.4 to 19)	30.8 (24.8 to 37.9)	0.3 (0.2 to 0.4)	117.2 (37.1 to 248.5)	116.7 (36 to 248.8)	26.1 (-31.2 to 126.1)
		Number	34 (23 to 49)	34 (22 to 49)	1 (0 to 1)	221 (177 to 272)	220 (175 to 270)	2 (1 to 3)	541.6 (314.3 to 923.8)	547.2 (315.5 to 939.9)	210.5 (72.3 to 452.3)
	Prevalence	Rate	68.9 (52.2 to 89.3)	137.7 (104 to 179.4)	2 (1.4 to 2.7)	141.7 (117.4 to 169.8)	282.2 (233.3 to 338.8)	2.7 (2 to 3.7)	105.7 (49 to 188.2)	104.9 (47.8 to 188)	37.1 (-11.5 to 108.1)
		Number	323 (240 to 423)	319 (236 to 417)	5 (3 to 7)	2002 (1637 to 2410)	1986 (1621 to 2394)	16 (12 to 23)	519 (344.6 to 781.9)	523 (345.7 to 792.6)	242.7 (116.5 to 437.4)
	Deaths	Rate	4.4 (2.9 to 6.7)	8.8 (5.7 to 13.3)	0.2 (0.1 to 0.3)	5.4 (4.4 to 6.7)	10.6 (8.6 to 13.2)	0.2 (0.1 to 0.2)	22.5 (-24.9 to 103.2)	21.3 (-26 to 101.7)	-14.7 (-51.3 to 48.4)
		Number	20 (13 to 29)	19 (13 to 28)	0 (0 to 1)	70 (57 to 86)	69 (56 to 85)	1 (1 to 1)	255.8 (124.1 to 480.4)	258.7 (122.4 to 485.2)	114 (20.5 to 285.3)
	DALYs	Rate	134.1 (89.9 to 191.6)	269.6 (180.1 to 388.1)	4.8 (3 to 7.2)	172.9 (139.9 to 211.7)	345.3 (279 to 422.9)	4 (2.9 to 5.4)	28.9 (-16 to 105)	28.1 (-17.5 to 103.7)	-17.2 (-53.3 to 47.5)
		Number	679 (459 to 950)	667 (445 to 940)	12 (8 to 18)	2506 (2017 to 3052)	2481 (1994 to 3029)	24 (18 to 33)	269.1 (146.1 to 474.8)	272.2 (146.8 to 486)	97.9 (13.5 to 251.7)
	YLLs	Rate	129.3 (85.7 to 186.5)	260 (171.6 to 377.4)	4.6 (2.9 to 7)	162.5 (129.8 to 200.2)	324.5 (258.5 to 400.1)	3.7 (2.7 to 5.1)	25.7 (-18.9 to 103.1)	24.8 (-19.8 to 101.7)	-19.2 (-54.6 to 45.4)
		Number	656 (438 to 924)	644 (428 to 914)	12 (7 to 18)	2356 (1878 to 2892)	2333 (1856 to 2869)	23 (16 to 32)	259.4 (138 to 462.7)	262.5 (138.5 to 474.7)	93 (9.1 to 248.8)
	YLDs	Rate	4.8 (2.9 to 7.4)	9.6 (5.7 to 14.9)	0.2 (0.1 to 0.3)	10.4 (7 to 14.7)	20.8 (13.8 to 29.4)	0.2 (0.1 to 0.4)	116.4 (46.9 to 232.8)	115.9 (45.7 to 235.6)	37.7 (-20.7 to 134.8)
		Number	23 (14 to 36)	23 (13 to 36)	0 (0 to 1)	150 (99 to 213)	148 (98 to 211)	1 (1 to 2)	541.5 (335.7 to 898.2)	546.9 (337.3 to 910.5)	238.2 (96.7 to 484)

Province	Measure	Metric	1990			2019			% Change (1990 to 2019)		
			Both	Female	Male	Both	Female	Male	Both	Female	Male
Qom	Incidence	Rate	9.8 (6.1 to 14.2)	19.8 (12.2 to 28.9)	0.5 (0.3 to 0.8)	18.3 (14.8 to 22.7)	37.4 (30.3 to 46.4)	0.5 (0.3 to 0.7)	86.1 (21.9 to 209.4)	89.2 (24.5 to 218.8)	-3.5 (-48 to 81)
		Number	36 (20 to 52)	36 (20 to 51)	1 (1 to 1)	240 (193 to 300)	238 (190 to 297)	3 (2 to 4)	560.5 (337.1 to 1073.2)	569 (341.6 to 1094.9)	218.3 (72.8 to 501.8)
	Prevalence	Rate	88.3 (62 to 115.2)	178.3 (123.2 to 234.5)	3.5 (2.4 to 5)	163.7 (136.3 to 199.5)	334 (277.9 to 408)	4.1 (3.1 to 5.6)	85.5 (36.3 to 170.8)	87.3 (36.8 to 177.7)	16.9 (-28.6 to 96.3)
		Number	319 (205 to 428)	313 (201 to 422)	6 (4 to 9)	2141 (1764 to 2625)	2117 (1743 to 2599)	24 (18 to 34)	570.3 (379.9 to 961)	576.2 (384.3 to 975.9)	280.2 (126.6 to 566.2)
	Deaths	Rate	6.2 (3.9 to 9.1)	12.4 (7.6 to 18.2)	0.4 (0.2 to 0.6)	6.9 (5.6 to 8.3)	14.3 (11.6 to 17.2)	0.2 (0.2 to 0.3)	10.6 (-28.6 to 84.4)	15.4 (-26.2 to 97)	-35.3 (-63.2 to 14.3)
		Number	21 (12 to 30)	20 (12 to 29)	1 (0 to 1)	81 (66 to 99)	80 (65 to 97)	1 (1 to 2)	289.3 (153.9 to 604.1)	294.5 (154.5 to 646.1)	119.8 (25.5 to 308.9)
	DALYs	Rate	190.6 (108.5 to 271.9)	385.3 (212.3 to 551.1)	10.2 (6.1 to 15.2)	210.8 (171.7 to 255.9)	429 (347.6 to 521.7)	6.5 (4.6 to 8.6)	10.6 (-26.8 to 96.5)	11.3 (-27.1 to 106.3)	-36.1 (-63.6 to 19)
		Number	741 (382 to 1046)	721 (371 to 1021)	20 (11 to 29)	2805 (2280 to 3443)	2765 (2236 to 3394)	40 (28 to 53)	278.7 (151.6 to 635.6)	283.5 (152.4 to 660.9)	102 (15.5 to 289.2)
	YLLs	Rate	184.2 (105 to 263.1)	372.5 (203.7 to 535.7)	9.8 (5.9 to 14.8)	198.7 (161 to 243.2)	404.3 (326.3 to 495.9)	6.1 (4.4 to 8.2)	7.8 (-29.4 to 94.5)	8.6 (-29.5 to 104.4)	-37.6 (-64.6 to 16.8)
		Number	717 (365 to 1017)	698 (352 to 993)	19 (11 to 28)	2645 (2128 to 3262)	2607 (2090 to 3221)	38 (27 to 50)	268.9 (140.3 to 619)	273.6 (141 to 643.5)	97.1 (12.5 to 280)
	YLDs	Rate	6.4 (3.4 to 9.9)	12.8 (6.8 to 20)	0.3 (0.2 to 0.5)	12.1 (8 to 17.5)	24.7 (16.1 to 35.6)	0.4 (0.2 to 0.6)	90.2 (30.9 to 205.3)	92.4 (31.1 to 213.4)	10.2 (-38.4 to 97.9)
		Number	24 (12 to 37)	23 (12 to 37)	1 (0 to 1)	160 (104 to 233)	158 (103 to 230)	2 (1 to 3)	573.6 (359.6 to 1049.3)	581.7 (362.1 to 1071.1)	258.1 (101 to 572.6)

Province	Measure	Metric	1990			2019			% Change (1990 to 2019)		
			Both	Female	Male	Both	Female	Male	Both	Female	Male
Semnan	Incidence	Rate	9.3 (6.2 to 13.8)	18.1 (11.9 to 27)	0.4 (0.2 to 0.6)	18.6 (14.7 to 23.1)	36.6 (28.9 to 45.6)	0.7 (0.5 to 0.9)	99.6 (27.8 to 226.6)	101.9 (27.9 to 232.3)	78.4 (-1.4 to 218.3)
		Number	28 (18 to 41)	27 (18 to 40)	1 (0 to 1)	148 (117 to 184)	145 (115 to 181)	2 (2 to 3)	433.9 (243.7 to 773.2)	436 (243.7 to 783.2)	327.8 (132.1 to 683.9)
	Prevalence	Rate	87.8 (66.5 to 116.2)	170.8 (128 to 227.6)	2.8 (1.9 to 3.9)	169.8 (138.6 to 205.8)	335 (272.6 to 407.9)	5.3 (3.9 to 7.3)	93.5 (38.4 to 172)	96.1 (40 to 178.1)	90 (15.4 to 206.7)
		Number	256 (191 to 341)	252 (187 to 337)	4 (3 to 6)	1339 (1088 to 1622)	1321 (1071 to 1605)	19 (13 to 26)	423.4 (270 to 655.8)	424.4 (268.5 to 661.9)	359.5 (172.3 to 655.5)
	Deaths	Rate	5.4 (3.6 to 8.3)	10.4 (6.8 to 15.8)	0.3 (0.2 to 0.4)	6.3 (5.1 to 7.7)	12.1 (9.7 to 15)	0.3 (0.2 to 0.4)	15.7 (-28.4 to 90.2)	16.9 (-28.4 to 93.7)	20.1 (-31.1 to 112.5)
		Number	15 (10 to 22)	15 (10 to 22)	0 (0 to 1)	46 (37 to 57)	45 (36 to 56)	1 (1 to 1)	207.3 (92.2 to 402.9)	207.7 (90.6 to 408.4)	193 (66.8 to 424.8)
	DALYs	Rate	164.9 (113.1 to 239.3)	323 (220.3 to 472.3)	6.9 (4.4 to 10.4)	198.1 (159.4 to 245.2)	390.3 (312.2 to 485)	8.2 (6 to 10.8)	20.1 (-21.4 to 90.9)	20.8 (-21 to 94.4)	17.9 (-32.9 to 110.4)
		Number	505 (346 to 736)	495 (336 to 724)	11 (7 to 16)	1588 (1273 to 1942)	1558 (1247 to 1916)	29 (21 to 39)	214.1 (107.6 to 400.4)	214.9 (105.8 to 404.6)	176.7 (56.2 to 400.2)
	YLLs	Rate	158.7 (107.5 to 230.9)	310.9 (210 to 455.1)	6.7 (4.2 to 10)	185.7 (148.6 to 231.6)	365.8 (292.7 to 458)	7.7 (5.6 to 10.3)	17 (-23.9 to 89.1)	17.7 (-24.3 to 92.1)	15.2 (-35 to 106.6)
		Number	487 (332 to 709)	477 (323 to 698)	10 (6 to 16)	1488 (1191 to 1839)	1460 (1165 to 1809)	28 (20 to 37)	205.6 (100.1 to 393)	206.4 (98.6 to 399.1)	170.4 (52.1 to 394.6)
	YLDs	Rate	6.2 (3.6 to 9.7)	12.1 (7 to 19.1)	0.3 (0.1 to 0.4)	12.5 (8.4 to 17.8)	24.5 (16.4 to 35.1)	0.5 (0.3 to 0.8)	100.6 (33.6 to 209.2)	102.9 (34.1 to 217)	88.3 (7.6 to 228.3)
		Number	19 (11 to 29)	18 (10 to 29)	0 (0 to 1)	99 (67 to 143)	98 (65 to 140)	2 (1 to 3)	436.6 (256.6 to 737.1)	438.3 (255.4 to 747.2)	350.4 (150.2 to 699.4)

Province	Measure	Metric	1990			2019			% Change (1990 to 2019)		
			Both	Female	Male	Both	Female	Male	Both	Female	Male
Sistan and Baluchistan	Incidence	Rate	5.5 (3.3 to 7.8)	12 (7.3 to 17.2)	0.2 (0.1 to 0.3)	10.1 (7.8 to 12.9)	20 (15.5 to 25.7)	0.2 (0.1 to 0.3)	83 (15.2 to 206.1)	66.1 (3.7 to 183.6)	-7.1 (-47.9 to 75.2)
		Number	35 (20 to 51)	35 (20 to 50)	1 (0 to 1)	183 (141 to 237)	181 (139 to 236)	1 (1 to 2)	418.5 (224.8 to 775.5)	424.7 (226.6 to 802.9)	113.6 (17.7 to 316.1)
	Prevalence	Rate	52.6 (38.5 to 67.8)	115.2 (84 to 149.7)	1.7 (1.1 to 2.4)	90.4 (73.6 to 112.4)	179.6 (146.6 to 223.7)	1.8 (1.3 to 2.4)	72.1 (24.9 to 142.3)	55.9 (12.2 to 121.4)	7 (-27.8 to 60.2)
		Number	320 (221 to 422)	315 (216 to 417)	5 (3 to 8)	1608 (1285 to 2014)	1594 (1272 to 2000)	14 (10 to 18)	401.9 (251.8 to 641.1)	406.3 (252.7 to 651.8)	151.3 (59.4 to 301.6)
	Deaths	Rate	4 (2.6 to 5.7)	8.7 (5.5 to 12.5)	0.2 (0.1 to 0.3)	4.9 (3.8 to 6.1)	9.7 (7.6 to 12.2)	0.1 (0.1 to 0.2)	22.3 (-23.6 to 96.8)	11.4 (-31.4 to 84.2)	-29.1 (-60.1 to 33.3)
		Number	24 (15 to 33)	23 (15 to 33)	1 (0 to 1)	78 (61 to 98)	77 (60 to 97)	1 (1 to 1)	230.1 (109.2 to 445.4)	234 (109.2 to 463.8)	63 (-9.9 to 242)
	DALYs	Rate	120.1 (77 to 167.6)	262.4 (166.7 to 371.5)	4.7 (2.5 to 7.3)	151.9 (118.5 to 190.3)	300.7 (234.6 to 377)	3.2 (2.3 to 4.4)	26.5 (-19.4 to 104.7)	14.6 (-27.1 to 90.1)	-31.4 (-62.1 to 41.4)
		Number	815 (502 to 1146)	798 (487 to 1129)	17 (8 to 28)	2834 (2216 to 3587)	2807 (2192 to 3561)	27 (19 to 37)	247.7 (123.4 to 481.4)	251.8 (125.6 to 491.5)	55.9 (-15.1 to 236)
	YLLs	Rate	116.5 (74 to 163.9)	254.5 (162.6 to 362)	4.5 (2.4 to 7.2)	145.2 (112.4 to 183)	287.4 (221.9 to 362.6)	3 (2.1 to 4.2)	24.6 (-21.2 to 104.4)	12.9 (-29.4 to 90)	-32.5 (-62.9 to 39.5)
		Number	792 (485 to 1115)	775 (473 to 1098)	17 (8 to 27)	2712 (2095 to 3457)	2686 (2069 to 3432)	26 (18 to 36)	242.3 (119 to 475.4)	246.3 (120.1 to 485.4)	53.5 (-16.5 to 235.8)
	YLDs	Rate	3.6 (2 to 5.5)	7.9 (4.4 to 12)	0.1 (0.1 to 0.2)	6.7 (4.5 to 9.7)	13.2 (8.8 to 19.2)	0.1 (0.1 to 0.2)	85.2 (20.1 to 193.3)	68.2 (7.4 to 169.3)	4 (-38.3 to 79.2)
		Number	23 (12 to 36)	22 (12 to 35)	0 (0 to 1)	123 (80 to 179)	121 (79 to 177)	1 (1 to 2)	434.4 (246.4 to 780.2)	440.7 (247.6 to 798.3)	138.8 (37.5 to 330.5)

Province	Measure	Metric	1990			2019			% Change (1990 to 2019)		
			Both	Female	Male	Both	Female	Male	Both	Female	Male
South Khorasan	Incidence	Rate	8.1 (5.6 to 11.2)	16.5 (11.4 to 22.9)	0.3 (0.2 to 0.4)	15.5 (12.5 to 19.2)	30 (24.2 to 37.3)	0.3 (0.2 to 0.4)	91.9 (23.3 to 185.4)	81.7 (15.7 to 172.8)	2.8 (-43.4 to 83.2)
		Number	32 (22 to 44)	31 (22 to 43)	1 (0 to 1)	121 (97 to 150)	120 (96 to 149)	1 (1 to 1)	282.9 (149.6 to 463.4)	286.3 (149.4 to 473.8)	80.1 (-0.9 to 219.6)
	Prevalence	Rate	74.6 (58 to 94.9)	153.3 (118.5 to 196.2)	2.1 (1.5 to 3)	139.5 (115.4 to 168.9)	270.2 (223.7 to 328.2)	2.5 (1.8 to 3.4)	87.1 (35.8 to 146.3)	76.3 (27.3 to 133.9)	16.4 (-25.6 to 74.4)
		Number	287 (221 to 369)	283 (218 to 365)	4 (3 to 6)	1082 (895 to 1316)	1073 (887 to 1307)	9 (7 to 12)	277.4 (171.8 to 402.6)	279.9 (172.4 to 408.1)	108.7 (29.4 to 221.2)
	Deaths	Rate	5.1 (3.6 to 7.2)	10.4 (7.3 to 14.8)	0.2 (0.1 to 0.3)	6.1 (5 to 7.5)	11.7 (9.5 to 14.5)	0.1 (0.1 to 0.2)	19.8 (-23.7 to 76.4)	12.6 (-29 to 67.9)	-26.2 (-58.3 to 28.4)
		Number	19 (13 to 26)	18 (13 to 26)	0 (0 to 1)	45 (37 to 56)	45 (36 to 55)	0 (0 to 1)	140.7 (54.3 to 250.4)	142.9 (55.1 to 255.3)	34.9 (-23.4 to 143.6)
	DALYs	Rate	154 (111.3 to 211.6)	315.3 (227.1 to 435)	5.2 (3.4 to 7.8)	184.2 (150.7 to 225)	356.2 (290.7 to 435.6)	3.7 (2.7 to 5)	19.6 (-20.5 to 71.9)	13 (-25.6 to 63.6)	-29.5 (-60 to 23.8)
		Number	624 (453 to 854)	612 (442 to 844)	11 (7 to 17)	1442 (1180 to 1765)	1429 (1167 to 1751)	13 (10 to 18)	131.3 (55.2 to 231.4)	133.4 (55.2 to 236.8)	18.9 (-32.6 to 110.9)
	YLLs	Rate	148.7 (106.5 to 205.2)	304.5 (217 to 422)	5.1 (3.2 to 7.6)	174 (141.7 to 215)	336.5 (273.6 to 415.9)	3.5 (2.5 to 4.8)	17 (-23.3 to 69.2)	10.5 (-28.3 to 61.4)	-31.1 (-61.5 to 22.1)
		Number	603 (433 to 830)	592 (424 to 817)	11 (7 to 16)	1363 (1107 to 1675)	1350 (1093 to 1660)	13 (9 to 17)	126 (49.1 to 226.8)	128 (49.6 to 231.9)	16.1 (-35 to 110)
	YLDs	Rate	5.3 (3.3 to 8)	10.8 (6.8 to 16.4)	0.2 (0.1 to 0.3)	10.2 (6.8 to 14.5)	19.7 (13.1 to 28.2)	0.2 (0.1 to 0.3)	92.8 (31.9 to 176.2)	82.6 (23.8 to 165.1)	13.7 (-33.6 to 88.9)
		Number	21 (13 to 31)	20 (13 to 31)	0 (0 to 1)	80 (53 to 115)	79 (52 to 114)	1 (0 to 1)	285.9 (167.2 to 457.1)	289.3 (166.6 to 467)	100.6 (14.7 to 237.2)

Province	Measure	Metric	1990			2019			% Change (1990 to 2019)		
			Both	Female	Male	Both	Female	Male	Both	Female	Male
Tehran	Incidence	Rate	14.9 (10.4 to 21.2)	30.3 (21 to 43)	0.3 (0.2 to 0.5)	20.2 (15.8 to 25.9)	40.5 (31.5 to 51.9)	0.3 (0.2 to 0.4)	35.5 (-12.9 to 111)	33.8 (-14.6 to 109.2)	-2.1 (-51.4 to 84.7)
		Number	750 (532 to 1061)	743 (526 to 1052)	7 (4 to 11)	3206 (2493 to 4146)	3185 (2474 to 4128)	21 (14 to 30)	327.7 (181.7 to 561.2)	328.7 (180.7 to 565.5)	212.2 (58.8 to 500.1)
	Prevalence	Rate	137.9 (105.1 to 182.6)	279.2 (213.2 to 370.2)	2.5 (1.7 to 3.9)	186.8 (151.4 to 229.9)	373.4 (302.2 to 460)	2.7 (2 to 3.6)	35.5 (-4.8 to 92.9)	33.7 (-6.6 to 91.3)	6.1 (-37.7 to 72.1)
		Number	6800 (5194 to 9118)	6742 (5152 to 9059)	58 (38 to 89)	29481 (23661 to 36680)	29289 (23436 to 36511)	192 (140 to 262)	333.5 (202.4 to 520)	334.4 (202.3 to 522.9)	233.9 (98.6 to 458.1)
	Deaths	Rate	7.2 (5 to 10.5)	14.3 (9.9 to 20.8)	0.2 (0.1 to 0.3)	6.8 (5.4 to 8.4)	13.6 (10.9 to 16.9)	0.1 (0.1 to 0.2)	-6 (-39.7 to 47.9)	-4.9 (-39.1 to 50.5)	-26.4 (-61.7 to 37.1)
		Number	313 (222 to 442)	310 (219 to 439)	4 (2 to 6)	994 (789 to 1233)	984 (780 to 1224)	10 (7 to 13)	217.3 (106.7 to 393.5)	218.1 (105.6 to 397.8)	157.7 (33.8 to 381.3)
	DALYs	Rate	210.9 (153.1 to 295.1)	430.3 (312 to 605.3)	4.6 (2.9 to 7.5)	201 (160.6 to 250.7)	400.8 (320.5 to 501.2)	3.5 (2.5 to 4.8)	-4.7 (-36.8 to 45.2)	-6.9 (-38.6 to 41.6)	-25.4 (-60 to 34.1)
		Number	10945 (8041 to 15328)	10829 (7948 to 15207)	116 (72 to 187)	32174 (25483 to 40287)	31917 (25259 to 40059)	257 (181 to 354)	194 (92.9 to 346.5)	194.8 (92.3 to 348.8)	121.2 (21 to 301.8)
	YLLs	Rate	201 (144.6 to 284)	410.4 (293.7 to 582)	4.4 (2.7 to 7.2)	187.4 (147.5 to 233.2)	373.6 (293.3 to 466.8)	3.2 (2.3 to 4.5)	-6.8 (-38.9 to 43.2)	-9 (-40.8 to 41.1)	-26.9 (-61.6 to 33.7)
		Number	10446 (7614 to 14769)	10335 (7509 to 14670)	111 (68 to 180)	30009 (23426 to 37517)	29769 (23212 to 37323)	240 (167 to 335)	187.3 (84.9 to 339)	188.1 (84.4 to 342.2)	116.3 (16.5 to 298.6)
	YLDs	Rate	9.9 (6.1 to 14.8)	20 (12.4 to 30)	0.2 (0.1 to 0.4)	13.6 (8.8 to 19.6)	27.2 (17.6 to 39)	0.2 (0.1 to 0.3)	38.1 (-6.7 to 107.1)	36.2 (-8.4 to 104.3)	4.8 (-44.4 to 89)
		Number	499 (313 to 772)	494 (310 to 766)	5 (3 to 9)	2165 (1393 to 3115)	2148 (1378 to 3093)	17 (10 to 25)	333.9 (188.4 to 557.1)	334.9 (188.6 to 561)	230.8 (79.5 to 504.5)

Province	Measure	Metric	1990			2019			% Change (1990 to 2019)		
			Both	Female	Male	Both	Female	Male	Both	Female	Male
West Azarbayejan	Incidence	Rate	8 (5.8 to 11.2)	16.5 (11.8 to 22.9)	0.2 (0.1 to 0.3)	15.7 (12.9 to 19.4)	31 (25.4 to 38.4)	0.2 (0.1 to 0.3)	95.3 (30.3 to 186.9)	88 (25.3 to 178.2)	25.5 (-25.9 to 108.1)
		Number	96 (69 to 131)	95 (69 to 130)	1 (1 to 1)	514 (419 to 635)	511 (416 to 632)	3 (2 to 4)	436.3 (265 to 686.6)	438.5 (265.7 to 695.4)	207.6 (72.7 to 424.2)
	Prevalence	Rate	75.1 (59.7 to 95.1)	154.3 (122.3 to 196.2)	1.5 (1.1 to 2.1)	141 (119.1 to 168.8)	277.5 (234.3 to 331.2)	2 (1.5 to 2.6)	87.7 (40.6 to 144.5)	79.9 (34.7 to 136.8)	30.8 (-9.8 to 89.9)
		Number	873 (690 to 1119)	865 (681 to 1110)	8 (6 to 12)	4615 (3869 to 5588)	4588 (3837 to 5525)	27 (20 to 37)	428.5 (289.9 to 601)	430.4 (290.2 to 605.9)	229.2 (116.6 to 386.2)
	Deaths	Rate	5.2 (3.7 to 7.6)	10.6 (7.5 to 15.5)	0.1 (0.1 to 0.2)	6.4 (5.3 to 7.6)	12.4 (10.2 to 14.8)	0.1 (0.1 to 0.2)	22.4 (-22 to 82.6)	17 (-25.3 to 75.4)	-11.9 (-46.2 to 44.1)
		Number	55 (40 to 78)	54 (39 to 77)	1 (0 to 1)	184 (151 to 222)	182 (150 to 220)	1 (1 to 2)	234.6 (119.9 to 392)	236 (119.2 to 399)	120.9 (32.3 to 263.5)
	DALYs	Rate	153.5 (112.7 to 215.4)	314.8 (229.3 to 444.5)	3.2 (2.2 to 5.1)	185.2 (152.5 to 223.6)	365.6 (300.4 to 442)	2.8 (2.1 to 3.8)	20.7 (-19.1 to 74.6)	16.1 (-22.8 to 69.7)	-12.9 (-46.5 to 39.7)
		Number	1942 (1447 to 2721)	1921 (1428 to 2705)	21 (14 to 33)	6194 (5096 to 7490)	6152 (5052 to 7444)	41 (31 to 56)	218.9 (117.1 to 355.9)	220.2 (117.5 to 362.2)	102.1 (20.8 to 229.3)
	YLLs	Rate	148.2 (108.4 to 209.3)	304 (220.3 to 433.1)	3.1 (2.1 to 4.9)	174.9 (143.3 to 212)	345.2 (282.5 to 420)	2.7 (1.9 to 3.6)	18 (-21.4 to 73)	13.6 (-24.8 to 67.9)	-14.8 (-48.4 to 38.1)
		Number	1879 (1389 to 2656)	1859 (1369 to 2637)	20 (13 to 32)	5852 (4778 to 7126)	5813 (4739 to 7087)	39 (29 to 53)	211.5 (110.7 to 353)	212.7 (110.2 to 356.7)	97.6 (16.4 to 223.9)
	YLDs	Rate	5.3 (3.3 to 8.1)	10.8 (6.8 to 16.6)	0.1 (0.1 to 0.2)	10.3 (6.8 to 14.5)	20.3 (13.4 to 28.4)	0.2 (0.1 to 0.3)	95.5 (36.4 to 176.5)	87.9 (30.6 to 167.1)	35.1 (-15.8 to 113.2)
		Number	63 (39 to 97)	63 (39 to 96)	1 (0 to 1)	342 (227 to 482)	339 (225 to 479)	2 (1 to 4)	438.6 (271.3 to 663.9)	440.8 (272 to 669.6)	231.9 (100 to 433.6)

Province	Measure	Metric	1990			2019			% Change (1990 to 2019)		
			Both	Female	Male	Both	Female	Male	Both	Female	Male
Yazd	Incidence	Rate	9.9 (6.5 to 14.6)	19.3 (12.6 to 28.4)	0.5 (0.3 to 0.7)	20.3 (15.8 to 25.4)	40.8 (31.4 to 51.7)	0.9 (0.6 to 1.3)	104.6 (28.1 to 221.6)	111.8 (32.3 to 234.9)	91.4 (9.5 to 257.3)
		Number	38 (25 to 56)	38 (25 to 55)	1 (1 to 1)	234 (180 to 298)	230 (175 to 292)	4 (3 to 6)	510.1 (287.7 to 848.9)	511.8 (284.6 to 863.5)	429.8 (199.1 to 915.3)
	Prevalence	Rate	93.3 (69.1 to 123.6)	181.1 (133 to 241.1)	3.4 (2.4 to 4.7)	184.1 (148.2 to 226.8)	372.4 (298 to 459.4)	6.9 (5 to 10)	97.2 (38.9 to 174.1)	105.6 (43.8 to 187.5)	104.4 (29 to 237.8)
		Number	355 (260 to 472)	348 (254 to 465)	6 (4 to 9)	2113 (1671 to 2626)	2078 (1637 to 2590)	36 (25 to 51)	495.9 (316.1 to 742.5)	496.4 (313.4 to 750.6)	466 (242.2 to 862.8)
	Deaths	Rate	5.9 (3.7 to 9.1)	11 (7 to 17.1)	0.3 (0.2 to 0.5)	6.7 (5.3 to 8.2)	13.1 (10.4 to 16.2)	0.4 (0.3 to 0.6)	14 (-30.2 to 83.4)	18.8 (-26.9 to 91.6)	22.7 (-24.7 to 114)
		Number	21 (14 to 31)	20 (13 to 31)	1 (0 to 1)	69 (54 to 85)	67 (53 to 83)	2 (1 to 3)	229.7 (109 to 424)	229.2 (106 to 426.5)	247.8 (95.9 to 518.4)
	DALYs	Rate	177.3 (119.7 to 259)	348.9 (233.6 to 512.6)	8.5 (5.4 to 12.4)	203.9 (161.8 to 252.2)	410.5 (321.4 to 508.5)	10.4 (7.3 to 14.3)	15 (-24.7 to 75.2)	17.7 (-23.1 to 80.4)	22.6 (-28.4 to 110.6)
		Number	713 (486 to 1034)	696 (470 to 1020)	17 (11 to 25)	2375 (1870 to 2953)	2319 (1816 to 2899)	55 (38 to 76)	232.9 (118 to 405.7)	233 (116.2 to 412.1)	226.9 (85.3 to 465.9)
	YLLs	Rate	170.7 (113.7 to 252.1)	336.1 (222.6 to 496.5)	8.2 (5.2 to 12)	190.3 (150.1 to 237)	383.2 (299 to 480.2)	9.8 (6.9 to 13.6)	11.5 (-27.8 to 72.9)	14 (-26.3 to 79.8)	19.6 (-31 to 109.2)
		Number	688 (466 to 1006)	671 (450 to 989)	16 (10 to 24)	2217 (1730 to 2782)	2165 (1681 to 2730)	52 (36 to 72)	222.3 (109.2 to 402.6)	222.4 (107.5 to 407)	218.8 (78.9 to 460)
	YLDs	Rate	6.6 (3.8 to 10.5)	12.8 (7.3 to 20.4)	0.3 (0.2 to 0.5)	13.6 (8.8 to 19.8)	27.4 (17.6 to 40)	0.6 (0.4 to 1)	106.3 (36.7 to 208.2)	113.6 (39.1 to 222.9)	102.2 (17.1 to 262.1)
		Number	26 (15 to 41)	25 (14 to 40)	1 (0 to 1)	158 (101 to 230)	155 (99 to 225)	3 (2 to 5)	516.4 (304.3 to 830)	517.7 (304.1 to 842.4)	458.2 (216.8 to 912.1)

Province	Measure	Metric	1990			2019			% Change (1990 to 2019)		
			Both	Female	Male	Both	Female	Male	Both	Female	Male
Zanjan	Incidence	Rate	5.7 (3.8 to 8.4)	11.4 (7.5 to 17)	0.1 (0.1 to 0.2)	12.6 (10.4 to 15.1)	24.5 (20.2 to 29.4)	0.2 (0.2 to 0.3)	121.9 (45.6 to 247.8)	115 (39.8 to 238.7)	54 (-10.4 to 159.7)
		Number	25 (17 to 37)	25 (17 to 36)	0 (0 to 1)	138 (113 to 166)	137 (112 to 165)	1 (1 to 2)	442.1 (265.8 to 730)	445 (265.4 to 741)	228.3 (89.3 to 455.5)
	Prevalence	Rate	58.9 (45.3 to 75.6)	118.2 (90.2 to 153.5)	1.4 (1 to 2)	115.3 (97.3 to 136.7)	224.2 (188.8 to 264.9)	2.1 (1.6 to 2.8)	95.8 (50.9 to 162)	89.7 (44.3 to 155.9)	47.7 (2.4 to 116.4)
		Number	256 (196 to 335)	253 (193 to 331)	3 (2 to 4)	1258 (1059 to 1481)	1248 (1048 to 1469)	10 (8 to 14)	392 (271.5 to 560.5)	394 (271.7 to 565.7)	230.2 (118.9 to 389.8)
	Deaths	Rate	3.6 (2.3 to 5.7)	7.2 (4.6 to 11.3)	0.1 (0.1 to 0.2)	5 (4.1 to 5.9)	9.6 (7.9 to 11.3)	0.1 (0.1 to 0.2)	37.7 (-15.5 to 114.9)	33.2 (-18.9 to 109.2)	6.6 (-33 to 85.5)
		Number	15 (10 to 22)	15 (10 to 22)	0 (0 to 0)	51 (43 to 61)	51 (42 to 60)	1 (0 to 1)	245.8 (120 to 422.8)	247.4 (119.2 to 429.7)	143.5 (48.4 to 331.9)
	DALYs	Rate	105 (71.2 to 154.2)	212.4 (141.9 to 315.1)	2.9 (1.9 to 4.3)	142 (118.6 to 168.2)	276.6 (230.1 to 328.3)	3 (2.2 to 4)	35.2 (-11.9 to 101.4)	30.2 (-15.7 to 95.6)	3.2 (-35.8 to 76.4)
		Number	496 (338 to 721)	489 (332 to 715)	7 (5 to 11)	1571 (1310 to 1868)	1556 (1296 to 1848)	15 (11 to 20)	216.7 (110.5 to 366)	218.3 (110.9 to 369.9)	106.6 (25.4 to 252.1)
	YLLs	Rate	101.1 (67.6 to 149.1)	204.5 (135.3 to 303.3)	2.8 (1.8 to 4.2)	133.6 (110.7 to 158.4)	260.3 (214.3 to 309.1)	2.8 (2.1 to 3.7)	32.2 (-15 to 99.1)	27.3 (-18.7 to 93.8)	0.9 (-38.3 to 73.4)
		Number	478 (322 to 699)	471 (316 to 693)	7 (5 to 11)	1479 (1223 to 1762)	1465 (1209 to 1746)	14 (10 to 19)	209.1 (103.1 to 362.3)	210.7 (103.6 to 366.2)	101.6 (21.9 to 247)
	YLDs	Rate	3.9 (2.4 to 6.2)	7.9 (4.8 to 12.6)	0.1 (0.1 to 0.2)	8.4 (5.7 to 11.7)	16.3 (11 to 22.8)	0.2 (0.1 to 0.3)	111.9 (48.2 to 204.9)	105.3 (43.1 to 196.9)	59.4 (-1 to 149.6)
		Number	18 (11 to 28)	17 (11 to 27)	0 (0 to 0)	92 (62 to 129)	91 (62 to 129)	1 (1 to 1)	421.4 (271.6 to 654.9)	424 (271.9 to 661.2)	243.6 (106.3 to 455.3)

Data in parentheses are 95% uncertainty intervals
* Age-standardized rate (per 100,000) † All ages
