# Supplementary material for: National and subnational burden of female and male breast cancer and risk factors in Iran from 1990 to 2019: results from the Global Burden of Disease study 2019
Source: Breast Cancer Res. 2023 Apr 26;25:47. doi: 10.1186/s13058-023-01633-4 (PMC10131337; doi:10.1186/s13058-023-01633-4)
Supplement: Supplementary file 8 — Additional file 8. Table 4 Percent changes (%) in breast cancer age-standardized rates and all-ages numbers of incidence, prevalence, deaths, disability-adjusted life years (DALYs), years of life lost (YLLs), and years lived with disability (YLDs) over two major time periods, between 1990 and 2010 and between 2010 and 2019, in Iran and all 31 provinces for both sexes, females, and males. [file 13058_2023_1633_MOESM8_ESM.pdf]

Location	Measure	Metric	2010			% Change					
						1990 to 2010			2010 to 2019		
			Both	Female	Male	Both	Female	Male	Both	Female	Male
Iran (Islamic Republic of)	Incidence	Rate[*]	12.6 (11.8 to 13.6)	25.1 (23.4 to 27.1)	0.3 (0.2 to 0.3)	36.7 (5.8 to 67.5)	35.2 (21.5 to 50.4)	33.7 (3.1 to 64.1)	35.5 (21.7 to 50.8)	13.4 (-15.2 to 53.8)	13.4 (-6.9 to 37)
		Number[†]	8113 (7556 to 8766)	8035 (7480 to 8692)	78 (70 to 88)	182.6 (122 to 240.5)	83.2 (64.2 to 104.4)	183.4 (121.9 to 242.2)	83.5 (64.4 to 104.6)	117.4 (62.7 to 189.7)	53.7 (25.3 to 83.8)
	Prevalence	Rate	117.1 (108.7 to 125.8)	232.7 (216.1 to 249.8)	2.3 (2 to 2.7)	33.2 (10.2 to 56.5)	34 (22.3 to 46.9)	30.3 (7.7 to 53.4)	34.1 (22.3 to 47.3)	15 (-8.6 to 44.3)	19.3 (0.5 to 40.7)
		Number	74034 (68695 to 79349)	73362 (68068 to 78679)	673 (585 to 779)	179.8 (130.7 to 227.9)	82.7 (65.9 to 101.7)	180.4 (131 to 228.9)	82.9 (66 to 101.8)	125.7 (77.6 to 184)	59.1 (32.6 to 87.7)
	Deaths	Rate	5.3 (5 to 5.7)	10.6 (10 to 11.2)	0.2 (0.1 to 0.2)	3.9 (-20.7 to 28.9)	12 (2.9 to 21.9)	2.6 (-22 to 27.8)	11.9 (2.7 to 21.8)	-8 (-27.4 to 21.6)	-3.8 (-16.5 to 8.8)
		Number	3111 (2947 to 3290)	3069 (2908 to 3246)	42 (39 to 45)	116.7 (69 to 161.3)	53 (40.5 to 66.4)	117.2 (68.8 to 163)	53.3 (40.5 to 66.9)	82.5 (42.5 to 138.3)	33.1 (15.9 to 50.4)
	DALYs	Rate	166.2 (157.5 to 176.5)	329.1 (312 to 349.5)	4.1 (3.8 to 4.4)	5.8 (-16.5 to 26)	11.7 (2.7 to 21.4)	2.8 (-19.1 to 22.6)	12 (3 to 21.9)	-8.7 (-28.5 to 19.1)	-2.2 (-15 to 10.1)
		Number	107929 (102426 to 114393)	106699 (101232 to 113211)	1230 (1140 to 1312)	113.1 (70.5 to 149.9)	51.1 (39 to 63.6)	113.7 (70.3 to 151.3)	51.3 (39.2 to 64.1)	68.9 (33.3 to 115.9)	30.5 (13 to 46.9)
	YLLs	Rate	157.7 (149.6 to 167)	312.3 (296 to 331.1)	3.9 (3.6 to 4.2)	4.6 (-17.7 to 24.7)	10.4 (1.4 to 19.7)	1.6 (-20.3 to 21.5)	10.7 (1.7 to 20.3)	-9.8 (-29.5 to 17.6)	-3.2 (-16 to 9.1)
		Number	102470 (97390 to 108321)	101300 (96260 to 107108)	1170 (1084 to 1245)	110.3 (67.8 to 146.8)	49.4 (37.1 to 62)	111 (67.7 to 148.1)	49.6 (37.2 to 62.3)	66.7 (31.2 to 112.6)	29.1 (11.7 to 45.7)
	YLDs	Rate	8.5 (6 to 11.6)	16.8 (11.9 to 23.1)	0.2 (0.1 to 0.3)	36.3 (8.5 to 64.5)	35.5 (22.3 to 51.4)	33.3 (5.9 to 61.7)	35.7 (22.4 to 51.9)	17.6 (-10.9 to 54.5)	17.6 (-3 to 41.6)
		Number	5459 (3822 to 7537)	5399 (3780 to 7465)	59 (41 to 85)	183 (124.3 to 239.6)	83.4 (64.9 to 105.7)	183.8 (124.5 to 241.8)	83.7 (65 to 106.4)	127.1 (70.6 to 193.5)	57.3 (28.7 to 88.7)

Location	Measure	Metric	2010			% Change					
						1990 to 2010			2010 to 2019		
			Both	Female	Male	Both	Female	Male	Both	Female	Male
**Alborz**	**Incidence**	**Rate***	15.3 (13 to 17.8)	31.5 (26.7 to 36.6)	0.5 (0.4 to 0.6)	24.5 (-14.8 to 101.1)	34.6 (2.7 to 76.4)	22.3 (-16.8 to 99.4)	32.8 (0.9 to 74.3)	23.8 (-28.5 to 130.1)	11 (-27.6 to 66.3)
		**Number†**	315 (265 to 372)	311 (261 to 367)	4 (3 to 6)	248.7 (133.4 to 493)	104.3 (52.8 to 169.6)	249 (132.5 to 501.3)	104.8 (52.7 to 172.8)	229.6 (87.9 to 538.8)	72.3 (11.4 to 159.9)
	**Prevalence**	**Rate**	143.3 (124.9 to 164.8)	294.5 (255.9 to 339.8)	4 (3.1 to 5.1)	27.7 (-6 to 83.9)	32.4 (4.9 to 67.7)	25.4 (-7.9 to 81.1)	30.3 (3.2 to 65.2)	30.9 (-17.1 to 113.8)	15.2 (-21.2 to 64.4)
		**Number**	2905 (2499 to 3366)	2869 (2464 to 3328)	36 (28 to 47)	260.9 (157.2 to 465)	103.1 (58.4 to 162.7)	261.1 (155.8 to 467)	103.4 (58.7 to 164.3)	248 (114 to 493.5)	76.1 (20 to 152.2)
	**Deaths**	**Rate**	5.8 (5.1 to 6.7)	12.1 (10.4 to 13.9)	0.3 (0.2 to 0.3)	-10.5 (-40.1 to 41.5)	12 (-12 to 41.9)	-10.2 (-40.3 to 43.4)	10.9 (-12.7 to 41.6)	-4.9 (-40.7 to 65.9)	-6.9 (-34.6 to 34.7)
		**Number**	103 (89 to 118)	101 (87 to 116)	2 (2 to 2)	149.1 (70.8 to 285.9)	74.8 (36.9 to 124.2)	149 (69.8 to 292.2)	75.3 (37 to 126.4)	154.8 (55.9 to 361.4)	49.2 (4.4 to 114.5)
	**DALYs**	**Rate**	175.3 (152.9 to 200)	357.9 (311 to 408.4)	6.4 (5.1 to 8.1)	-10.4 (-37.8 to 37.5)	11.6 (-11.5 to 41.3)	-13 (-39.6 to 33.9)	10.2 (-12.9 to 40.1)	-5 (-41.2 to 70)	-3.8 (-32.9 to 37)
		**Number**	3658 (3174 to 4178)	3596 (3105 to 4119)	62 (49 to 78)	144.2 (71.2 to 285.3)	69.1 (32.7 to 116.2)	144.2 (70.5 to 291.1)	69.5 (32.5 to 117.6)	144.8 (52.7 to 357.7)	45.2 (-1.9 to 109)
	**YLLs**	**Rate**	165 (142.7 to 189.7)	336.6 (290.2 to 388.3)	6.1 (4.8 to 7.6)	-12 (-39.4 to 36.5)	10.2 (-13.9 to 41.6)	-14.6 (-41.5 to 34.2)	8.8 (-15.3 to 40.2)	-6.6 (-43 to 69.4)	-4.9 (-34.2 to 37)
		**Number**	3444 (2964 to 3964)	3385 (2907 to 3920)	59 (46 to 73)	139.6 (65.7 to 285.7)	67 (28.7 to 116.4)	139.5 (64.8 to 289)	67.4 (28.8 to 118.1)	140.8 (49.1 to 353.5)	43.5 (-3.9 to 108.3)
	**YLDs**	**Rate**	10.4 (7.1 to 14.6)	21.2 (14.5 to 29.9)	0.4 (0.2 to 0.5)	27.4 (-11.8 to 98.1)	33.9 (1.3 to 76)	24.9 (-13.8 to 97.3)	32 (-0.4 to 74.2)	30.7 (-21.8 to 129.7)	14.2 (-25.5 to 68.2)
		**Number**	214 (145 to 302)	211 (142 to 298)	3 (2 to 5)	256.6 (145.2 to 480.5)	103.4 (51.8 to 173.7)	256.8 (144.6 to 481.8)	103.9 (51.4 to 176.3)	247.7 (102.6 to 534.5)	75.2 (15.3 to 159.3)

Location	Measure	Metric	2010			% Change					
						1990 to 2010			2010 to 2019		
			Both	Female	Male	Both	Female	Male	Both	Female	Male
**Ardebil**	**Incidence**	**Rate***	10.7 (9.3 to 12.3)	21.1 (18.3 to 24.4)	0.2 (0.2 to 0.3)	28.9 (-8.7 to 83.5)	32.5 (5.5 to 63.5)	21.8 (-14.1 to 74.9)	32.6 (5.2 to 63.6)	21.8 (-23.9 to 105.7)	6.8 (-25.7 to 56.1)
		**Number†**	110 (95 to 128)	109 (94 to 126)	1 (1 to 1)	130.4 (62.1 to 228.6)	71.2 (34.6 to 113.2)	130.8 (61.7 to 231.5)	71.6 (34.7 to 114.1)	99.4 (24.6 to 237.1)	29.1 (-10.5 to 87.2)
	**Prevalence**	**Rate**	100.9 (89.9 to 113.8)	198.8 (176.7 to 224.2)	2.1 (1.7 to 2.6)	32.4 (3.9 to 69.9)	30.1 (7.9 to 55.4)	24.7 (-2.6 to 61.7)	29.9 (7.5 to 55.4)	19.7 (-15.1 to 67.2)	12.8 (-15.4 to 54.5)
		**Number**	1024 (903 to 1157)	1014 (894 to 1146)	10 (8 to 13)	138.7 (80.6 to 213.9)	69 (38.6 to 104.6)	139.1 (80.4 to 216.4)	69.3 (38.7 to 105.3)	104.5 (43.2 to 201.8)	35 (-0.2 to 86.5)
	**Deaths**	**Rate**	4.8 (4.3 to 5.4)	9.4 (8.4 to 10.7)	0.1 (0.1 to 0.2)	-6.8 (-34.7 to 38.3)	13.6 (-8 to 37.4)	-12.4 (-39.1 to 31.7)	12.2 (-9.3 to 35.8)	-4.1 (-37.3 to 59.7)	-5.3 (-32.5 to 30)
		**Number**	46 (41 to 52)	45 (41 to 51)	1 (1 to 1)	68.3 (20.1 to 142.1)	44.6 (16.4 to 76.3)	68.4 (20 to 143.4)	45 (16.6 to 77.5)	64 (1.7 to 174.4)	14 (-18.1 to 58.8)
	**DALYs**	**Rate**	143.3 (127.6 to 162.4)	280.8 (250 to 318.5)	3.6 (2.9 to 4.4)	-8.9 (-33.8 to 29)	12.8 (-8.9 to 38.2)	-14.1 (-38 to 22.2)	13.5 (-8.7 to 39)	-8.3 (-40.9 to 49.8)	-5.6 (-32 to 30.6)
		**Number**	1486 (1324 to 1692)	1468 (1306 to 1672)	18 (14 to 22)	56.5 (14.1 to 119.5)	46.6 (17.6 to 80.7)	56.7 (13.9 to 120.2)	47 (17.6 to 81.5)	42 (-10.4 to 130.9)	15 (-17.8 to 60.2)
	**YLLs**	**Rate**	136.1 (121.3 to 154.4)	266.6 (237.8 to 303.1)	3.4 (2.8 to 4.2)	-10.4 (-35.2 to 27.1)	11.7 (-10.8 to 37.8)	-15.5 (-39.2 to 21.3)	12.4 (-10.6 to 38.9)	-9.7 (-42.2 to 48.6)	-6.5 (-32.8 to 30.8)
		**Number**	1411 (1260 to 1611)	1394 (1242 to 1593)	17 (14 to 21)	53.7 (11.7 to 118.1)	45.3 (15.3 to 80.3)	53.9 (11.8 to 118.4)	45.7 (15.7 to 81.2)	39.7 (-12.6 to 129.8)	14.1 (-18.6 to 60)
	**YLDs**	**Rate**	7.2 (4.9 to 9.9)	14.2 (9.6 to 19.5)	0.2 (0.1 to 0.3)	33.1 (-2.9 to 82.9)	32.7 (5.4 to 67.7)	25.8 (-8.5 to 74.3)	32.8 (5.2 to 67.1)	26.6 (-17 to 98.4)	10.6 (-22.5 to 59.2)
		**Number**	74 (51 to 102)	74 (50 to 101)	1 (1 to 1)	138.4 (71.7 to 233.3)	71.2 (34.7 to 116.8)	138.7 (71.1 to 235.7)	71.7 (34.9 to 117.7)	111.1 (36 to 236.9)	32.1 (-6.6 to 89.7)

Location	Measure	Metric	2010			% Change					
						1990 to 2010			2010 to 2019		
			Both	Female	Male	Both	Female	Male	Both	Female	Male
Bushehr	Incidence	Rate*	13.9 (12.4 to 15.7)	29.4 (26.3 to 33)	0.3 (0.2 to 0.3)	53.4 (2.4 to 132.6)	26 (4.2 to 51.5)	60 (6.7 to 144.3)	23.3 (1.7 to 48.4)	43.9 (-10.1 to 150.2)	-4.2 (-34.1 to 39.3)
		Number†	107 (94 to 121)	106 (94 to 120)	1 (1 to 1)	250.8 (140 to 442.8)	83.2 (49.4 to 122.3)	251.2 (139.5 to 445)	83.6 (49.8 to 123)	203.8 (86.3 to 417.9)	34.3 (-8.4 to 93.2)
	Prevalence	Rate	126.7 (114.3 to 141)	266.6 (240.3 to 297.3)	2.2 (1.7 to 2.8)	47.4 (9.5 to 99.4)	27.8 (8.8 to 49.4)	53.4 (13.3 to 108.4)	25 (5.7 to 46.5)	32.7 (-6.1 to 90.9)	5.8 (-20.9 to 44.2)
		Number	959 (857 to 1077)	952 (851 to 1070)	7 (6 to 9)	243.2 (153.9 to 379.2)	86 (54.8 to 121.1)	243.6 (154.2 to 380.9)	86.3 (55 to 121.8)	193.9 (100.7 to 326.5)	45.2 (8.1 to 96.7)
	Deaths	Rate	5.9 (5.4 to 6.5)	12.3 (11.2 to 13.6)	0.2 (0.1 to 0.2)	14.2 (-26.6 to 75.7)	0.8 (-14.1 to 17.6)	17.8 (-24.7 to 82.8)	-1.7 (-16.4 to 15.2)	16 (-25.8 to 87.7)	-18.1 (-41 to 12.1)
		Number	40 (36 to 44)	39 (36 to 43)	0 (0 to 1)	155.8 (71.6 to 294.6)	47.2 (24 to 73.2)	155.9 (70.9 to 292.3)	47.6 (24 to 73.9)	145.3 (56 to 309.1)	14.8 (-16.4 to 55.9)
	DALYs	Rate	181 (164.7 to 199.9)	381.3 (346.7 to 421.5)	3.7 (3 to 4.4)	14.3 (-22 to 71.2)	2.8 (-13.1 to 20.7)	18.7 (-19.2 to 79.3)	0.7 (-15 to 18.3)	13.3 (-26.3 to 86.2)	-18.4 (-40 to 10.2)
		Number	1418 (1287 to 1572)	1405 (1276 to 1558)	13 (11 to 16)	155.2 (77.8 to 286.3)	48.4 (23.9 to 75.2)	155.4 (77.4 to 290.2)	48.7 (24.1 to 75.8)	133.8 (50.5 to 281.4)	12.2 (-16.8 to 50.7)
	YLLs	Rate	171.8 (156.6 to 189.2)	361.9 (329.7 to 399.5)	3.5 (2.9 to 4.2)	12.8 (-23.4 to 70)	1.4 (-15 to 19.5)	17.2 (-20.9 to 78.3)	-0.6 (-17 to 17.3)	12 (-28 to 85.3)	-19.5 (-41.3 to 9)
		Number	1347 (1220 to 1493)	1335 (1209 to 1479)	12 (10 to 15)	151.6 (73.5 to 285.3)	46.4 (21.4 to 73.7)	151.8 (72.8 to 289.2)	46.7 (21.5 to 73.9)	131 (48.8 to 280.2)	10.8 (-18.2 to 49.3)
	YLDs	Rate	9.2 (6.4 to 12.7)	19.4 (13.4 to 26.8)	0.2 (0.1 to 0.3)	50.9 (3.7 to 122.6)	28.3 (3.2 to 55.3)	57.4 (7.8 to 134.2)	25.5 (0.5 to 52.4)	43.8 (-6.3 to 126.4)	1.2 (-28.4 to 45.5)
		Number	71 (49 to 99)	71 (49 to 98)	1 (0 to 1)	246.6 (142.2 to 421.3)	85.8 (47 to 129.1)	247 (141.5 to 423.5)	86.2 (47.1 to 129.6)	208.7 (95.8 to 391.4)	40 (-1 to 98)

Location	Measure	Metric	2010			% Change					
						1990 to 2010			2010 to 2019		
			Both	Female	Male	Both	Female	Male	Both	Female	Male
Chahar Mahaal and Bakhtiari	Incidence	Rate*	10.3 (8.5 to 12.4)	20.4 (16.7 to 24.5)	0.2 (0.2 to 0.3)	27.8 (-10.4 to 95.2)	15.7 (-13.8 to 57.1)	21.7 (-15.4 to 88.5)	16.6 (-13.4 to 58.3)	29.5 (-16.9 to 116.2)	-10.8 (-43.1 to 35)
		Number†	69 (57 to 83)	69 (57 to 83)	1 (1 to 1)	152.7 (75.5 to 280.8)	66.8 (22.9 to 126.8)	153 (75 to 284.2)	67.3 (22.9 to 128.1)	134 (46.6 to 294)	20.4 (-21.8 to 85.1)
	Prevalence	Rate	97.8 (83.3 to 113.9)	192.6 (163.7 to 224.2)	2 (1.6 to 2.7)	25.9 (-2.2 to 68.6)	20.4 (-4.8 to 53.2)	19.8 (-7.8 to 61.7)	21 (-4.9 to 54.3)	21.9 (-12.9 to 75.6)	1.3 (-26.8 to 41.5)
		Number	647 (551 to 755)	641 (544 to 749)	6 (5 to 8)	151.5 (89.9 to 238.7)	72.6 (33.5 to 122.1)	151.7 (89.6 to 242.2)	72.9 (33.6 to 122.9)	129.7 (58.7 to 235.8)	33.8 (-3.8 to 89)
	Deaths	Rate	4.3 (3.6 to 5)	8.4 (7 to 9.9)	0.1 (0.1 to 0.2)	-4.9 (-35.9 to 50.4)	-10.1 (-31.6 to 18.2)	-9 (-38.5 to 44.9)	-10.5 (-32 to 18)	3.1 (-29.9 to 62.6)	-25.5 (-50.2 to 7.5)
		Number	27 (22 to 31)	26 (22 to 31)	0 (0 to 0)	91.9 (34.3 to 195)	27.7 (-3.1 to 68.3)	91.9 (33.2 to 197.4)	28.1 (-3 to 69.7)	96 (28.8 to 214.1)	0.1 (-33.7 to 45.7)
	DALYs	Rate	126.6 (105.5 to 147.6)	248.6 (206.9 to 291.2)	3.2 (2.5 to 4.1)	-5.1 (-32.6 to 42.9)	-6.3 (-29.1 to 23)	-10.2 (-36.6 to 36.2)	-5.2 (-28.7 to 24.6)	1.1 (-31.8 to 61.6)	-24.1 (-47.7 to 9.8)
		Number	856 (714 to 1003)	846 (703 to 993)	10 (8 to 13)	82.2 (29.5 to 172.3)	35.1 (1.5 to 77.3)	82.3 (28.9 to 173.9)	35.5 (1.8 to 78.3)	75 (17.6 to 175.5)	2.2 (-29.7 to 48.7)
	YLLs	Rate	119.6 (98.6 to 140.5)	234.9 (193.2 to 276.7)	3 (2.3 to 3.9)	-6.4 (-34.4 to 43.1)	-7.8 (-31.2 to 22.1)	-11.5 (-38 to 36.4)	-6.7 (-30.5 to 23.8)	-0.2 (-33.5 to 61.6)	-25.3 (-49.1 to 8.9)
		Number	809 (672 to 947)	799 (661 to 937)	10 (8 to 12)	79.3 (25.9 to 170.9)	33 (-1.3 to 76.6)	79.4 (25.3 to 172.1)	33.4 (-1.4 to 77.7)	72.3 (15.1 to 173.7)	0.8 (-31.6 to 46.4)
	YLDs	Rate	7 (4.6 to 9.9)	13.7 (9.1 to 19.5)	0.2 (0.1 to 0.3)	27.1 (-8.9 to 85.7)	19.2 (-9.9 to 60.9)	21.1 (-14.5 to 78.3)	20 (-9.4 to 62.1)	30.8 (-12.4 to 102.2)	-4.6 (-36.6 to 40.7)
		Number	47 (31 to 67)	46 (31 to 66)	1 (0 to 1)	151.9 (75 to 273.4)	70.9 (27.4 to 130.9)	152.1 (74.8 to 275.5)	71.5 (27.4 to 132)	140.5 (56.2 to 280.7)	26.7 (-14.6 to 87.2)

Location	Measure	Metric	2010			% Change					
						1990 to 2010			2010 to 2019		
			Both	Female	Male	Both	Female	Male	Both	Female	Male
**East Azarbayejan**	**Incidence**	**Rate***	11.7 (10 to 13.5)	23.1 (19.8 to 26.6)	0.3 (0.2 to 0.4)	45.4 (-4 to 116.2)	49.7 (13.2 to 91.3)	41.3 (-7.7 to 110.6)	50.3 (13.8 to 93.2)	35.6 (-16.7 to 131.4)	22.4 (-17 to 76.9)
		**Number†**	411 (349 to 476)	407 (345 to 471)	4 (3 to 6)	157.7 (72.1 to 284)	94.2 (46.2 to 152)	158.2 (71.4 to 285.2)	94.6 (45.9 to 153.1)	117.3 (29.9 to 276.7)	63.3 (10 to 136.9)
	**Prevalence**	**Rate**	108.3 (94.6 to 123)	214.8 (187.4 to 243.9)	2.4 (1.9 to 3)	38.1 (2.9 to 80.9)	47.2 (17.1 to 82.8)	33.3 (-1.2 to 75.2)	47.7 (17.4 to 83.9)	28.4 (-12.1 to 87.8)	29 (-6.6 to 76.3)
		**Number**	3777 (3285 to 4315)	3740 (3248 to 4279)	37 (29 to 46)	151.2 (84.2 to 238)	92.3 (50.5 to 142.9)	151.6 (83.6 to 238.8)	92.5 (50.5 to 143.4)	115.5 (44.8 to 234.7)	67.9 (20.8 to 131.7)
	**Deaths**	**Rate**	5.2 (4.5 to 5.9)	10.2 (8.8 to 11.6)	0.2 (0.2 to 0.2)	8.6 (-25.9 to 58.7)	21.3 (-4.7 to 52.1)	5.4 (-28.2 to 56.3)	21 (-5.4 to 52.6)	12.6 (-25.3 to 85.1)	1.7 (-28.7 to 41.4)
		**Number**	163 (142 to 187)	161 (140 to 184)	3 (2 to 3)	91 (32.7 to 176.7)	59.6 (24.8 to 102.3)	91.1 (32.1 to 179)	59.9 (25 to 103.5)	82.8 (16.1 to 209.3)	38.2 (-5.4 to 96)
	**DALYs**	**Rate**	164.6 (143.5 to 188)	325.2 (282.5 to 371.5)	4.6 (3.8 to 5.7)	9.4 (-23.8 to 57.3)	20 (-5 to 51.6)	6 (-26.4 to 53.5)	20.7 (-4.6 to 52.9)	9.1 (-29.3 to 78.9)	4.6 (-27.1 to 44.8)
		**Number**	5892 (5102 to 6766)	5818 (5029 to 6689)	73 (60 to 91)	88.4 (32.4 to 170.2)	55.2 (22.6 to 97)	88.7 (31.8 to 174.4)	55.5 (22.2 to 97.9)	68.9 (7.6 to 182.6)	37.1 (-6.1 to 91.1)
	**YLLs**	**Rate**	156.8 (135.6 to 179.6)	309.6 (267.1 to 355.3)	4.4 (3.6 to 5.5)	8.1 (-25.1 to 58)	18.6 (-7.1 to 51.3)	4.7 (-27.6 to 53.4)	19.2 (-6.7 to 52.7)	8 (-30.7 to 79.6)	3.5 (-28.4 to 43.8)
		**Number**	5612 (4813 to 6472)	5542 (4745 to 6401)	70 (57 to 87)	85.9 (29.7 to 170)	53.4 (19.7 to 97)	86.2 (29.3 to 173.1)	53.6 (19.6 to 97.8)	67 (5.3 to 182.6)	35.7 (-7.3 to 90.5)
	**YLDs**	**Rate**	7.9 (5.3 to 11.1)	15.6 (10.5 to 22)	0.2 (0.1 to 0.3)	44 (0.6 to 105.8)	49.1 (13.7 to 96.1)	39.5 (-2.7 to 99.7)	49.7 (13.8 to 97.5)	37.5 (-13.5 to 121.4)	27.4 (-11.7 to 81.2)
		**Number**	279 (188 to 397)	276 (185 to 393)	3 (2 to 5)	158.3 (79 to 275.2)	92.8 (44.7 to 157.4)	158.7 (78.7 to 277.5)	93.1 (44.8 to 158.6)	124.9 (39.6 to 266.1)	67.1 (15.8 to 140.6)

Location	Measure	Metric	2010			% Change					
						1990 to 2010			2010 to 2019		
			Both	Female	Male	Both	Female	Male	Both	Female	Male
**Fars**	**Incidence**	**Rate***	13.2 (11.2 to 15.5)	26.3 (22.3 to 30.8)	0.3 (0.2 to 0.3)	60.1 (3.2 to 148.2)	38.5 (4.7 to 83.3)	57.5 (0.9 to 146.6)	39.6 (5.2 to 84.9)	24.3 (-22.8 to 116.6)	24.5 (-17 to 85.8)
		**Number†**	532 (449 to 628)	528 (445 to 624)	5 (4 to 6)	236.5 (117.7 to 433)	88.3 (40.6 to 151.7)	237.6 (117.3 to 436.5)	88.5 (40.5 to 152.4)	142.2 (47.3 to 333.8)	68.5 (13.3 to 154.5)
	**Prevalence**	**Rate**	121.7 (105.5 to 139.7)	241.6 (209.3 to 277.2)	2.2 (1.7 to 2.9)	48.1 (7.5 to 103.4)	37.9 (8.2 to 75)	46.1 (5.4 to 101.3)	38.8 (8.8 to 76.7)	20 (-16.9 to 76.5)	29 (-6.3 to 79.8)
		**Number**	4823 (4141 to 5575)	4783 (4108 to 5531)	40 (31 to 52)	220.1 (126 to 353.7)	88.6 (45.4 to 144.3)	221 (125.9 to 356.9)	88.7 (45.4 to 144.8)	141.8 (60.8 to 272.3)	72.1 (23.1 to 141.2)
	**Deaths**	**Rate**	5.4 (4.7 to 6.2)	10.7 (9.3 to 12.2)	0.2 (0.1 to 0.2)	20.2 (-21.1 to 86.6)	10.6 (-13 to 40.5)	19.6 (-21.9 to 87)	11.1 (-12.8 to 41.1)	1.6 (-34.6 to 71.7)	4.3 (-27.2 to 46.1)
		**Number**	195 (169 to 225)	193 (167 to 222)	2 (2 to 3)	152.7 (67.5 to 291.8)	51.1 (18.6 to 93)	153.4 (66.9 to 296.1)	51.2 (18.4 to 93.6)	106.3 (26.8 to 257)	42.1 (-2.2 to 103.3)
	**DALYs**	**Rate**	173.7 (150.2 to 199.1)	343.6 (296.8 to 394.4)	4 (3.2 to 5)	23.8 (-15.8 to 88.1)	11.2 (-12.6 to 41.5)	20.9 (-18.3 to 84.3)	12.2 (-12.3 to 43)	2.7 (-34.6 to 75.2)	5.2 (-26.9 to 49.7)
		**Number**	7049 (6043 to 8110)	6975 (5970 to 8028)	74 (59 to 92)	153.8 (71.9 to 288.4)	51.1 (19.3 to 92.8)	154.6 (71.5 to 292.5)	51.2 (18.4 to 93.5)	93.4 (23.2 to 231.3)	40.6 (-4.5 to 101.6)
	**YLLs**	**Rate**	164.8 (142.2 to 190.4)	326.1 (280.8 to 377.2)	3.8 (3 to 4.8)	22.5 (-17.8 to 88.9)	9.7 (-14.5 to 40.7)	19.5 (-20.3 to 86.2)	10.6 (-14.2 to 42.2)	1.7 (-35.6 to 74.1)	4 (-28.8 to 49.3)
		**Number**	6691 (5711 to 7730)	6621 (5642 to 7636)	71 (56 to 89)	150.7 (67.3 to 288.5)	49 (15.8 to 93.6)	151.5 (66.9 to 293)	49.1 (15.6 to 94.2)	91.3 (20.4 to 228.3)	39 (-7 to 102)
	**YLDs**	**Rate**	8.8 (6 to 12.5)	17.5 (12 to 24.7)	0.2 (0.1 to 0.3)	55.5 (4.9 to 134.3)	39.5 (5.2 to 83.3)	53 (2.7 to 131.9)	40.5 (4.9 to 85.7)	26.1 (-19.2 to 109.4)	28.6 (-11.3 to 88.2)
		**Number**	358 (243 to 505)	354 (240 to 501)	4 (2 to 5)	230.2 (120.6 to 410.5)	89.2 (41.3 to 153.3)	231.2 (120 to 416.9)	89.3 (41.1 to 153.8)	148.8 (54.9 to 321.4)	71.9 (17.8 to 151.1)

Location	Measure	Metric	2010			% Change					
						1990 to 2010			2010 to 2019		
			Both	Female	Male	Both	Female	Male	Both	Female	Male
Gilan	Incidence	Rate*	14.6 (12.5 to 16.7)	28.3 (24.4 to 32.7)	0.3 (0.3 to 0.5)	54.5 (3.8 to 134.8)	32 (1.5 to 70.2)	54.6 (3.1 to 137)	33.3 (2.4 to 72.5)	14.3 (-28.6 to 86.9)	13.6 (-24.8 to 69.2)
		Number†	389 (333 to 450)	385 (330 to 446)	4 (3 to 6)	184.5 (92.7 to 336.2)	66 (27.9 to 115.5)	185.7 (92.5 to 341)	66.2 (27.7 to 116.4)	103.9 (26.9 to 247.6)	48.3 (-2.2 to 124.6)
	Prevalence	Rate	134.3 (117.3 to 151.4)	261.7 (228.1 to 295.3)	2.9 (2.3 to 3.8)	52.1 (14.3 to 103.5)	30.7 (5 to 62.8)	53.1 (14.4 to 106.3)	31.9 (5.6 to 64.6)	27.6 (-11.3 to 86.6)	17 (-16.1 to 64.9)
		Number	3560 (3101 to 4032)	3525 (3069 to 3999)	35 (27 to 45)	188.2 (111.4 to 294.8)	65.5 (31.7 to 107.6)	188.9 (110.6 to 297.5)	65.7 (31.5 to 108.1)	131.3 (55.7 to 253.9)	51.2 (5.7 to 115.2)
	Deaths	Rate	5.7 (5.1 to 6.5)	11 (9.8 to 12.5)	0.2 (0.2 to 0.2)	14.4 (-21.9 to 68.9)	10.3 (-13.8 to 38.4)	16.3 (-21.1 to 73.1)	11.6 (-13.1 to 40.6)	-10.1 (-39.9 to 50.2)	-0.9 (-32 to 39.2)
		Number	143 (127 to 162)	141 (125 to 160)	2 (2 to 3)	116 (48.8 to 221.8)	43 (11.3 to 80.1)	116.9 (48.8 to 225.9)	43.1 (11 to 80.7)	69.8 (10.3 to 180.3)	32.9 (-8.2 to 89.1)
	DALYs	Rate	181.4 (160.1 to 205.1)	352.5 (310.5 to 399.1)	4.9 (4 to 6.1)	15.6 (-18 to 69.4)	11.6 (-13.2 to 39.1)	14.6 (-19.1 to 68.8)	12.6 (-12.6 to 40.9)	-9.1 (-40.6 to 45.6)	-0.7 (-30.7 to 39.6)
		Number	4880 (4308 to 5526)	4819 (4248 to 5465)	61 (50 to 76)	107.4 (46.9 to 206.5)	40.9 (9.5 to 76.9)	108.3 (46.3 to 209.2)	41.1 (9.3 to 77.3)	54.2 (-0.9 to 146.5)	28 (-10.8 to 82.1)
	YLLs	Rate	171.6 (151.4 to 194.7)	333.5 (293.7 to 378.9)	4.7 (3.8 to 5.8)	14.1 (-20 to 68.5)	10.4 (-15 to 39.1)	13.1 (-21 to 68.4)	11.4 (-14.6 to 40.8)	-10.3 (-41.8 to 45.4)	-1.6 (-31.6 to 38.7)
		Number	4618 (4070 to 5251)	4560 (4011 to 5189)	58 (47 to 72)	104.5 (43.1 to 204.5)	39.5 (7 to 76.3)	105.4 (43.1 to 206.8)	39.7 (6.4 to 76.7)	52 (-2.9 to 144.6)	26.8 (-13.2 to 81.7)
	YLDs	Rate	9.8 (6.6 to 13.8)	19 (12.9 to 26.8)	0.3 (0.2 to 0.4)	49 (2.5 to 118.6)	32.2 (1.8 to 69)	49.2 (2.3 to 121.8)	33.4 (2.4 to 70.7)	18.3 (-24.7 to 88.6)	16.4 (-21.8 to 68.5)
		Number	262 (177 to 368)	259 (175 to 364)	3 (2 to 5)	175.7 (90.4 to 312.6)	66 (26.9 to 113.9)	176.8 (90.2 to 317.9)	66.2 (26.4 to 114.5)	110.8 (31.9 to 249.5)	50.2 (1.2 to 121.6)

Location	Measure	Metric	2010			% Change					
						1990 to 2010			2010 to 2019		
			Both	Female	Male	Both	Female	Male	Both	Female	Male
Golestan	Incidence	Rate*	10.6 (9.3 to 12)	20.7 (18.2 to 23.5)	0.2 (0.2 to 0.3)	48.6 (4.1 to 123.6)	50.3 (16.4 to 90)	44.3 (0.3 to 118)	50 (16 to 90.1)	18.4 (-24 to 96.2)	22.3 (-14.8 to 72.3)
		Number†	151 (132 to 173)	150 (131 to 172)	1 (1 to 2)	214.6 (122.3 to 369.2)	104.2 (56.5 to 162.5)	215.6 (122.4 to 372.4)	104.5 (56.7 to 163.4)	132.7 (49.3 to 292.2)	59.3 (11.4 to 125.3)
	Prevalence	Rate	96.1 (86 to 108.3)	187.8 (167.8 to 212.4)	1.9 (1.5 to 2.4)	40.6 (9.9 to 85.6)	48.3 (19.6 to 82.1)	36.8 (6.6 to 81.1)	48 (18.9 to 81.9)	17.2 (-14 to 64.9)	25.6 (-6.5 to 65.9)
		Number	1347 (1188 to 1527)	1335 (1178 to 1515)	11 (9 to 14)	208.9 (134.5 to 315.5)	102.5 (60.4 to 153.1)	209.6 (134.3 to 317.2)	102.8 (60.3 to 153.8)	138 (70.2 to 243.4)	61.6 (19.7 to 119.7)
	Deaths	Rate	4.9 (4.4 to 5.5)	9.5 (8.5 to 10.7)	0.1 (0.1 to 0.2)	9.2 (-23.5 to 59.7)	23.7 (-3.6 to 53.9)	6.7 (-25.3 to 56.5)	22.7 (-4.4 to 52.7)	-7.4 (-38.3 to 48.8)	8.8 (-24 to 50.6)
		Number	63 (56 to 70)	62 (55 to 70)	1 (1 to 1)	128.4 (63.1 to 232.3)	70.5 (32.7 to 113.4)	129.1 (62.4 to 236.9)	70.9 (32.6 to 114.4)	87.1 (20.7 to 216.8)	42.9 (0.2 to 98.6)
	DALYs	Rate	156.5 (139.8 to 177.3)	305.3 (272.5 to 345.7)	3.6 (2.9 to 4.3)	9.5 (-21.4 to 55.7)	28 (0.6 to 60.1)	5.7 (-24.5 to 52.4)	28.2 (0.3 to 60.6)	-4 (-36.5 to 56.9)	7.1 (-24.4 to 46.4)
		Number	2282 (2024 to 2590)	2259 (2001 to 2568)	23 (19 to 27)	125.9 (64 to 219.4)	73 (34.8 to 116.9)	126.5 (63.3 to 222.5)	73.3 (34.9 to 117.7)	81.8 (18.5 to 197.3)	38 (-2.8 to 89.3)
	YLLs	Rate	149.5 (133.3 to 168.9)	291.6 (259.6 to 329.9)	3.4 (2.8 to 4.1)	8.3 (-22.5 to 54.8)	27 (-1.1 to 59.1)	4.4 (-25.6 to 51.1)	27.1 (-1.8 to 59.4)	-5 (-37.3 to 56.9)	6.2 (-25.1 to 47)
		Number	2181 (1926 to 2474)	2159 (1907 to 2456)	22 (18 to 26)	123.1 (60.8 to 217.8)	71.6 (32.3 to 116)	123.6 (60.4 to 221.8)	71.9 (32.2 to 116.8)	79.8 (17.3 to 194.5)	37 (-3.7 to 90.2)
	YLDs	Rate	7 (4.8 to 9.7)	13.7 (9.4 to 19)	0.2 (0.1 to 0.2)	46 (4.4 to 109.5)	50.7 (15.3 to 93.9)	41.8 (1.4 to 105.4)	50.5 (14.9 to 94)	23.1 (-18 to 91.6)	25.8 (-10.5 to 74)
		Number	101 (68 to 141)	100 (68 to 140)	1 (1 to 1)	213.2 (121.3 to 351.4)	104.1 (53.8 to 164.7)	214 (121.2 to 355.4)	104.5 (53.7 to 165.9)	143.8 (61 to 282.1)	61.7 (14.9 to 127.5)

Location	Measure	Metric	2010			% Change					
						1990 to 2010			2010 to 2019		
			Both	Female	Male	Both	Female	Male	Both	Female	Male
Hamadan	Incidence	Rate*	11.2 (9.8 to 12.9)	22 (19.2 to 25.4)	0.2 (0.1 to 0.2)	35 (-7.7 to 99.8)	29.8 (2.2 to 64.6)	30.5 (-11.3 to 94.3)	31 (3.1 to 66.8)	10.6 (-30.8 to 85.7)	8.2 (-26.6 to 55)
		Number†	178 (155 to 205)	176 (154 to 204)	1 (1 to 2)	128.7 (56.9 to 236)	60.1 (26.2 to 103.9)	129.3 (56.7 to 240.1)	60.4 (26.4 to 104.8)	75.9 (10.9 to 194.2)	31.2 (-10.8 to 87.1)
	Prevalence	Rate	105.2 (93.6 to 118.7)	206.7 (183.7 to 233.4)	1.7 (1.4 to 2.2)	32.8 (0.7 to 74.8)	28.8 (5.7 to 58.1)	27.9 (-3.5 to 69.3)	29.9 (6.6 to 59.9)	7.2 (-23.2 to 53.4)	15.1 (-13.1 to 51.6)
		Number	1647 (1464 to 1869)	1634 (1450 to 1858)	13 (10 to 17)	129 (71.4 to 207.3)	59.7 (30.5 to 97.6)	129.5 (71 to 209)	59.8 (30.3 to 98)	79.6 (27.1 to 159.2)	37.9 (3.4 to 82)
	Deaths	Rate	4.9 (4.3 to 5.6)	9.6 (8.4 to 10.9)	0.1 (0.1 to 0.1)	1.3 (-31.9 to 46)	10.2 (-13.3 to 37.1)	-1.9 (-34.3 to 42.8)	10.7 (-13 to 38)	-11.1 (-43.4 to 44.5)	-4.4 (-34.3 to 31.8)
		Number	73 (64 to 83)	72 (63 to 82)	1 (1 to 1)	74.7 (18.6 to 151.1)	36.8 (7.7 to 70.8)	75 (18.4 to 153.2)	37 (7.6 to 71.2)	51 (-5.7 to 142.5)	17.8 (-18.7 to 61.6)
	DALYs	Rate	155.2 (135.5 to 176.6)	303.9 (265.5 to 345.7)	3 (2.4 to 3.6)	2.8 (-29 to 46.2)	8.8 (-14.2 to 35)	-1 (-32.1 to 41.1)	10.2 (-13.1 to 36.7)	-10.4 (-41.8 to 41.4)	-5.7 (-35.1 to 30.2)
		Number	2471 (2162 to 2803)	2448 (2139 to 2780)	23 (18 to 27)	69.5 (17.3 to 140.4)	34.6 (6.2 to 67.3)	69.9 (17.2 to 141.7)	34.7 (6.1 to 67.6)	36.2 (-11 to 112.7)	13.7 (-22 to 55.6)
	YLLs	Rate	147.6 (128.1 to 168.5)	289 (251.4 to 330.3)	2.8 (2.2 to 3.5)	1.6 (-30.7 to 46.9)	7.7 (-15.6 to 35.4)	-2.3 (-33.7 to 41)	9.2 (-14.3 to 37.2)	-11.3 (-43.2 to 40.3)	-6.7 (-36.1 to 30)
		Number	2350 (2050 to 2681)	2328 (2028 to 2661)	22 (17 to 26)	67.2 (14.7 to 140.3)	33.3 (4.6 to 68)	67.6 (14.4 to 141.4)	33.5 (4.3 to 68.4)	34.5 (-13.1 to 111.3)	12.6 (-24 to 54.8)
	YLDs	Rate	7.6 (5.1 to 10.6)	14.9 (10.1 to 20.8)	0.1 (0.1 to 0.2)	36 (-3.7 to 93.7)	29.5 (1.4 to 64.5)	31.3 (-7.4 to 88.4)	30.7 (1.8 to 66.4)	13.5 (-25.3 to 79.3)	12.5 (-20.7 to 55.8)
		Number	121 (83 to 169)	119 (82 to 168)	1 (1 to 2)	131.6 (62.2 to 231.1)	59.5 (23.8 to 101.8)	132.2 (62 to 233)	59.7 (24 to 102.8)	83.5 (21.5 to 188.4)	35 (-4.4 to 87.4)

Location	Measure	Metric	2010			% Change					
						1990 to 2010			2010 to 2019		
			Both	Female	Male	Both	Female	Male	Both	Female	Male
**Hormozgan**	**Incidence**	**Rate***	10.1 (8.8 to 11.4)	20 (17.5 to 22.6)	0.4 (0.3 to 0.5)	68.3 (15.7 to 173.2)	26.6 (-0.2 to 56)	61.5 (9.7 to 167.9)	27.6 (0.1 to 57.7)	2.5 (-37.2 to 86.7)	5.1 (-30.5 to 52.6)
		**Number†**	99 (86 to 113)	98 (85 to 111)	2 (1 to 2)	273.2 (156 to 536.2)	97 (52.1 to 146.5)	278.9 (157.6 to 548.4)	97.6 (52.3 to 148.2)	97 (16.8 to 263.3)	57.9 (6.8 to 128)
	**Prevalence**	**Rate**	87.8 (77.3 to 98.4)	173.5 (152.9 to 194.1)	3 (2.4 to 3.8)	51 (16.5 to 101.3)	32.7 (8.8 to 59.6)	44 (9.7 to 94.9)	33.6 (9.2 to 61)	16.5 (-23.6 to 84.5)	12.5 (-21.4 to 53.3)
		**Number**	856 (747 to 963)	842 (735 to 947)	13 (11 to 17)	245.7 (157.1 to 394.6)	105.2 (64.1 to 150.1)	248.6 (157.5 to 400.2)	105.8 (64.4 to 151.4)	128 (44.5 to 271.4)	65.8 (17.6 to 129.4)
	**Deaths**	**Rate**	5.1 (4.5 to 5.7)	10 (8.8 to 11.2)	0.2 (0.2 to 0.3)	22.3 (-14.8 to 84.4)	0.3 (-18.6 to 21.8)	18.3 (-18.8 to 82.7)	0.9 (-18.5 to 22.6)	-21 (-50.7 to 43.3)	-12.2 (-37.9 to 23.9)
		**Number**	44 (39 to 50)	44 (39 to 49)	1 (1 to 1)	161.6 (82.8 to 316.8)	52.4 (24.6 to 86)	165.8 (84 to 320.1)	52.8 (24.2 to 87.6)	51.2 (-6.1 to 172.8)	31.4 (-8.3 to 84.6)
	**DALYs**	**Rate**	151.4 (133.5 to 170.3)	298.4 (263 to 335.5)	6.4 (5.2 to 7.9)	20.2 (-14.8 to 96.7)	2.9 (-15.8 to 25.2)	14.4 (-19.8 to 89.7)	3.6 (-15.7 to 26.5)	-19.9 (-49.6 to 43.9)	-11 (-38.9 to 25.1)
		**Number**	1525 (1347 to 1716)	1495 (1320 to 1685)	30 (25 to 37)	160.7 (84.9 to 355.4)	58.6 (28.4 to 94.8)	164.3 (85.3 to 367)	59.2 (28.3 to 96)	54.3 (-3.2 to 181.3)	32.2 (-7.5 to 84.4)
	**YLLs**	**Rate**	144.9 (128 to 162.6)	285.5 (252.2 to 321.5)	6.1 (5 to 7.5)	18.8 (-16.3 to 94.8)	1.6 (-17.4 to 24.5)	13.1 (-21.3 to 88.8)	2.3 (-17.2 to 25.4)	-21 (-50.4 to 43)	-11.9 (-39.7 to 25.6)
		**Number**	1460 (1291 to 1647)	1431 (1259 to 1617)	29 (24 to 36)	157.3 (81.7 to 353.4)	56.7 (26.1 to 93.6)	160.9 (82.3 to 364.9)	57.2 (25.7 to 94.5)	52.5 (-5.1 to 180.2)	30.9 (-8.8 to 83.6)
	**YLDs**	**Rate**	6.5 (4.4 to 9)	12.9 (8.7 to 17.7)	0.3 (0.2 to 0.4)	62.6 (15.7 to 141.2)	30.8 (2.8 to 63.6)	55.8 (10 to 135.5)	31.7 (2.8 to 65.6)	12.3 (-31.2 to 93)	10.3 (-27.1 to 59.1)
		**Number**	65 (44 to 90)	64 (43 to 89)	1 (1 to 2)	267.7 (155 to 486.4)	102.2 (56.7 to 156.6)	272.5 (154.6 to 507.9)	103 (56.6 to 157.6)	117.4 (31.2 to 277.3)	63.4 (10.3 to 132)

Location	Measure	Metric	2010			% Change					
						1990 to 2010			2010 to 2019		
			Both	Female	Male	Both	Female	Male	Both	Female	Male
**Ilam**	**Incidence**	**Rate***	12.1 (10.7 to 13.7)	24.4 (21.4 to 27.6)	0.4 (0.3 to 0.5)	93 (24.3 to 216.9)	31.3 (5 to 62.4)	76.2 (11.5 to 191.6)	29.4 (3.8 to 59.7)	66 (-0.3 to 189.3)	-0.4 (-31.8 to 42.5)
		**Number†**	51 (45 to 58)	50 (44 to 57)	1 (1 to 1)	322.5 (177.9 to 576.1)	83.3 (45.9 to 125.4)	324.3 (177.7 to 587)	84 (46.2 to 126.6)	222.2 (95.5 to 462.9)	31.5 (-10.5 to 88.1)
	**Prevalence**	**Rate**	109 (96.6 to 121.8)	219.9 (195 to 245.4)	3 (2.4 to 3.8)	76.5 (29 to 147.1)	33.5 (10.6 to 60.5)	59.8 (15.6 to 124.5)	31.6 (9 to 58.4)	59 (7.8 to 135.5)	6.1 (-21 to 46.7)
		**Number**	455 (402 to 513)	449 (396 to 506)	6 (5 to 8)	295.8 (186.9 to 466.1)	86.3 (52.3 to 125)	297.1 (185.5 to 469.8)	86.9 (53 to 126.5)	218.4 (112.6 to 388)	37.2 (-0.7 to 91.2)
	**Deaths**	**Rate**	5.1 (4.6 to 5.7)	10.5 (9.3 to 11.6)	0.2 (0.2 to 0.2)	37.8 (-12.7 to 118.4)	11 (-9.4 to 36.1)	26.7 (-21 to 104.9)	8.2 (-11.8 to 32.8)	26.3 (-18.7 to 111.7)	-11.8 (-37.9 to 20.6)
		**Number**	19 (17 to 22)	19 (17 to 21)	0 (0 to 0)	200 (95.9 to 369)	52.5 (23.8 to 85.9)	201.3 (95.7 to 379.3)	53.2 (23.9 to 87.6)	144.8 (55.6 to 316.2)	17.1 (-17.6 to 59.1)
	**DALYs**	**Rate**	155.5 (137.8 to 172.4)	310.5 (275 to 344.1)	5.4 (4.4 to 6.6)	39 (-8 to 112.9)	12.2 (-8.9 to 36.3)	26 (-17.5 to 96.7)	10.9 (-9.7 to 34.5)	30.2 (-16.4 to 116)	-13.1 (-38.1 to 16.8)
		**Number**	665 (588 to 739)	654 (577 to 728)	11 (9 to 14)	196 (99.3 to 353.8)	55.8 (27.6 to 88.2)	197 (99.1 to 359.7)	56.5 (28 to 89.9)	147.6 (57.4 to 315.9)	13.2 (-19.1 to 55.2)
	**YLLs**	**Rate**	147.5 (130.5 to 163.7)	294.5 (260.8 to 326.9)	5.1 (4.2 to 6.3)	37.1 (-9.6 to 113.3)	11 (-10.3 to 35.8)	24.3 (-19.2 to 96.4)	9.8 (-11.1 to 34.1)	28.6 (-17.8 to 115.6)	-13.9 (-38.6 to 16.3)
		**Number**	631 (559 to 701)	620 (549 to 689)	11 (9 to 13)	191.7 (95 to 351.3)	54.2 (25.6 to 87.9)	192.7 (94.4 to 357.3)	54.9 (25.7 to 89.1)	144.4 (55.4 to 312.9)	12.2 (-20.4 to 54.9)
	**YLDs**	**Rate**	8 (5.5 to 11)	16.1 (11.1 to 22.2)	0.3 (0.2 to 0.4)	84.9 (26.6 to 181.9)	33.2 (4.8 to 66.6)	68.4 (13.7 to 157.6)	31.5 (3.6 to 64)	68.7 (5.8 to 171.8)	3.3 (-26.8 to 48.8)

Location	Measure	Metric	2010			% Change					
						1990 to 2010			2010 to 2019		
			Both	Female	Male	Both	Female	Male	Both	Female	Male
		**Number**	34 (23 to 47)	33 (23 to 47)	1 (0 to 1)	308.7 (175.3 to 531.5)	85.3 (45.2 to 132.7)	310.2 (174.5 to 535.8)	86.1 (45.8 to 134.6)	233.4 (107 to 446.8)	34.5 (-6.4 to 92.1)
**Isfahan**	**Incidence**	**Rate***	12.6 (10.8 to 14.5)	25.5 (21.8 to 29.3)	0.3 (0.2 to 0.4)	40.7 (-7.2 to 128.5)	39.4 (7.8 to 80.6)	40.9 (-7.3 to 130.5)	39 (7.2 to 80.5)	20.9 (-25.6 to 109.8)	30.7 (-12.5 to 95.3)
		**Number†**	581 (496 to 671)	576 (490 to 665)	6 (4 to 7)	201.4 (98.1 to 391.1)	84 (40.6 to 140.3)	202 (97.5 to 394)	84.1 (40.4 to 141.3)	147.5 (50.7 to 347.7)	74.2 (16.6 to 162.9)
	**Prevalence**	**Rate**	119.3 (104.1 to 135)	240.7 (210.1 to 273.2)	2.3 (1.8 to 3)	33.1 (-2 to 86.1)	36.8 (10.5 to 70.8)	33.9 (-2.2 to 89.7)	36.3 (9.9 to 69.9)	16.7 (-20.6 to 76.2)	33.5 (-3.7 to 89.7)
		**Number**	5393 (4701 to 6145)	5345 (4657 to 6090)	48 (38 to 63)	190 (109.2 to 319.5)	82.3 (44.8 to 129.4)	190.5 (109 to 322.3)	82.3 (44.9 to 130.2)	143.9 (62.9 to 276.6)	75.6 (25.1 to 149.8)
	**Deaths**	**Rate**	5 (4.4 to 5.7)	10.1 (8.8 to 11.4)	0.2 (0.1 to 0.2)	7.7 (-29.6 to 75.1)	15.7 (-9.1 to 48.2)	9.7 (-28.6 to 80.2)	15.7 (-8.9 to 48.7)	0.5 (-35.5 to 71.4)	10.4 (-23.3 to 55.3)
		**Number**	209 (184 to 237)	206 (181 to 234)	3 (2 to 4)	132.3 (52.9 to 282.4)	55.5 (21.5 to 100.5)	132.6 (52.3 to 287.1)	55.6 (21.3 to 101.5)	114.3 (34.8 to 277)	52 (4.3 to 114.3)
	**DALYs**	**Rate**	159.6 (140.1 to 181.1)	321.3 (281.7 to 365.4)	3.9 (3.2 to 4.9)	13.3 (-23.7 to 80.8)	13.7 (-10.1 to 44.1)	12.5 (-24.7 to 82)	13.3 (-10.6 to 44)	2.9 (-34 to 81)	10.4 (-23.7 to 52.1)
		**Number**	7395 (6512 to 8426)	7310 (6404 to 8349)	85 (70 to 108)	137.1 (58.8 to 295.8)	50.3 (17.3 to 91.3)	137.6 (58.2 to 300.1)	50.4 (17.2 to 91.7)	103 (28.8 to 252.3)	44.3 (-2.1 to 106.2)
	**YLLs**	**Rate**	151 (132.4 to 171.8)	304 (266.2 to 346.6)	3.7 (3 to 4.7)	12.1 (-25.6 to 83)	12.3 (-12.9 to 44.8)	11.3 (-25.9 to 85.4)	11.9 (-13.3 to 45)	2 (-35.6 to 80)	9.1 (-24.9 to 52.4)
		**Number**	7000 (6138 to 8009)	6919 (6051 to 7918)	81 (66 to 103)	134.4 (56.1 to 299)	48.5 (14.6 to 91.8)	134.9 (55.5 to 302)	48.5 (14.4 to 92.6)	100.9 (25.8 to 253.9)	42.6 (-4 to 105.6)
	**YLDs**	**Rate**	8.6 (5.8 to 12)	17.3 (11.8 to 24)	0.2 (0.1 to 0.3)	38.2 (-6.3 to 109)	39.1 (5.2 to 81)	38.5 (-6.8 to 111)	38.5 (4.5 to 80.9)	22.3 (-22.7 to 103.7)	34 (-9.3 to 96.8)

Location	Measure	Metric	2010			% Change					
						1990 to 2010			2010 to 2019		
			Both	Female	Male	Both	Female	Male	Both	Female	Male
		Number	395 (269 to 547)	391 (266 to 541)	4 (3 to 6)	197.6 (101.5 to 364.6)	83.6 (37.2 to 141.2)	198.2 (101 to 365.9)	83.7 (37.4 to 141.8)	152.3 (59.1 to 326.4)	76.2 (19.6 to 161.5)
Kerman	Incidence	Rate*	10.9 (9.5 to 12.4)	21.5 (18.7 to 24.5)	0.3 (0.2 to 0.3)	46.7 (1.2 to 114.6)	20.2 (-3.9 to 49.6)	42.7 (-2 to 110.3)	22.7 (-2.3 to 52.9)	25.3 (-19.2 to 108.6)	1 (-28.9 to 42.6)
		Number†	236 (206 to 269)	233 (203 to 266)	3 (2 to 3)	229.2 (125.5 to 385.4)	72.6 (36.5 to 118.9)	230.3 (124.4 to 391.3)	72.9 (36.7 to 119.8)	150.4 (57.1 to 316.8)	44 (2.2 to 103.4)
	Prevalence	Rate	99 (88.2 to 111.5)	195.5 (174 to 220.3)	2.2 (1.8 to 2.8)	39.1 (6.3 to 81)	21.9 (0.9 to 47.5)	35.6 (3.2 to 76.9)	24.2 (2.3 to 50.6)	24.3 (-11.1 to 78.3)	8.8 (-17.2 to 45.1)
		Number	2115 (1870 to 2397)	2094 (1849 to 2375)	21 (17 to 27)	219.5 (138.5 to 324.6)	75.1 (42.3 to 116)	220.2 (138.1 to 325.7)	75.4 (42.4 to 116.5)	160.9 (80.9 to 279.9)	51.7 (13.6 to 106.6)
	Deaths	Rate	5 (4.4 to 5.6)	9.7 (8.7 to 10.9)	0.2 (0.1 to 0.2)	8 (-25.4 to 61.6)	3.5 (-16.5 to 28.1)	5.8 (-27.3 to 59.2)	5 (-15.7 to 30)	1 (-32.4 to 61.9)	-10 (-35.2 to 21.8)
		Number	96 (86 to 108)	95 (84 to 106)	1 (1 to 2)	135.7 (63.6 to 248)	47.9 (18.7 to 83.7)	136.3 (63.3 to 252.2)	48.1 (18.5 to 84.5)	101.8 (32.4 to 229.4)	29.7 (-7.5 to 79.3)
	DALYs	Rate	154.2 (137.1 to 172.7)	303.3 (269.8 to 339.5)	4.3 (3.4 to 5.3)	8.5 (-22.9 to 57.2)	4 (-15.7 to 29.7)	4.8 (-25.9 to 53.6)	6.3 (-14.1 to 32.8)	-0.4 (-34.2 to 59.3)	-9.2 (-34.9 to 22.5)
		Number	3389 (3013 to 3786)	3346 (2971 to 3740)	43 (35 to 53)	138 (69.3 to 243.4)	49.2 (19.6 to 86.3)	138.6 (69.1 to 247.4)	49.4 (19.6 to 87.2)	96.1 (29.5 to 215.8)	27.9 (-9 to 75.9)
	YLLs	Rate	147 (130.4 to 164.7)	289.2 (256.5 to 324.1)	4.1 (3.3 to 5)	7.2 (-24.1 to 56.7)	3.1 (-16.9 to 29.3)	3.6 (-27.1 to 52.7)	5.4 (-15.3 to 32.9)	-1.5 (-35.3 to 57.9)	-9.9 (-36.2 to 22.5)
		Number	3232 (2878 to 3623)	3191 (2835 to 3579)	41 (33 to 51)	134.9 (66.4 to 242.4)	47.9 (17.3 to 86.6)	135.5 (66.2 to 247.2)	48.2 (17.2 to 87.7)	93.9 (27.8 to 215.7)	27 (-10.5 to 75.9)
	YLDs	Rate	7.2 (4.8 to 9.8)	14.1 (9.5 to 19.4)	0.2 (0.1 to 0.3)	43.6 (1.4 to 101.9)	22.5 (-3.6 to 53.4)	39.8 (-1.5 to 100.1)	25 (-2.3 to 57)	28 (-15.2 to 97.5)	5.6 (-24.2 to 47.2)

Location	Measure	Metric	2010			% Change					
						1990 to 2010			2010 to 2019		
			Both	Female	Male	Both	Female	Male	Both	Female	Male
		Number	157 (106 to 218)	155 (105 to 216)	2 (1 to 3)	226.4 (127.8 to 369.1)	75 (35.8 to 122.6)	227.4 (127.7 to 374.6)	75.4 (35.4 to 123.2)	160.9 (70.4 to 307.8)	48.2 (7.5 to 107.8)
Kermanshah	Incidence	Rate*	11.9 (10.2 to 13.8)	23.4 (20.1 to 27.2)	0.3 (0.2 to 0.3)	58.8 (9 to 136.8)	28.1 (-2 to 65.2)	48.7 (0.9 to 122.9)	27.7 (-2.5 to 64.7)	16.5 (-27.5 to 104.8)	9.8 (-25.2 to 60.6)
		Number†	203 (173 to 237)	201 (171 to 235)	2 (2 to 3)	209 (114 to 367.5)	62.2 (23.5 to 111.1)	210.6 (113.5 to 375.5)	62.5 (23.5 to 112)	106.3 (26.1 to 263.3)	37.4 (-7.4 to 97.8)
	Prevalence	Rate	106.1 (93.5 to 121.8)	208.8 (183.7 to 240.2)	2.2 (1.7 to 2.8)	52.2 (16.7 to 103.9)	29.9 (3.3 to 61.3)	41.2 (7.7 to 89.5)	29.5 (3 to 61.1)	18.9 (-17.7 to 77.7)	17.2 (-13.5 to 61.8)
		Number	1799 (1566 to 2071)	1781 (1551 to 2054)	17 (14 to 22)	202.6 (127 to 318.3)	65.6 (30.8 to 107.9)	203.8 (126.9 to 320.7)	65.8 (30.9 to 108.4)	116.7 (46.2 to 228.7)	44.1 (4.1 to 99.4)
	Deaths	Rate	5.5 (4.8 to 6.2)	10.8 (9.5 to 12.4)	0.2 (0.1 to 0.2)	18 (-19.6 to 76.9)	6.4 (-16.6 to 32.2)	10.8 (-25.3 to 68.8)	5.2 (-17.6 to 30.9)	-7.9 (-39.7 to 65.7)	-5.6 (-32.5 to 31.6)
		Number	86 (76 to 98)	85 (75 to 97)	1 (1 to 1)	132.6 (61.6 to 251.2)	35.1 (4.8 to 68.8)	133.9 (61.3 to 258.2)	35.3 (5 to 69.2)	68.4 (7.4 to 198.9)	20.5 (-13.1 to 68.2)
	DALYs	Rate	171.6 (150 to 196.9)	335.8 (293.6 to 385.4)	4.3 (3.4 to 5.5)	18.4 (-17.6 to 75.4)	5.5 (-18.3 to 31.1)	10.5 (-23.7 to 66.3)	5.5 (-18.5 to 31.3)	-5.3 (-40 to 64.6)	-6.3 (-32.9 to 31.7)
		Number	2977 (2606 to 3410)	2942 (2573 to 3371)	36 (29 to 44)	123.7 (57.1 to 236.3)	33.9 (2.8 to 67)	124.7 (56.7 to 239.8)	34.1 (2.7 to 67.3)	62.4 (3 to 178.8)	15.9 (-16.5 to 61)
	YLLs	Rate	163.8 (142.9 to 187.4)	320.5 (279.4 to 367)	4.1 (3.3 to 5.2)	17 (-19 to 74.6)	4.4 (-20 to 30.2)	9.2 (-24.9 to 65.6)	4.4 (-20.2 to 30.3)	-6.3 (-41.2 to 65.2)	-7.3 (-34.2 to 30.8)
		Number	2843 (2481 to 3252)	2809 (2448 to 3215)	34 (27 to 42)	120.8 (53.4 to 233.6)	32.4 (0.6 to 67)	121.8 (53.5 to 239.7)	32.6 (0.4 to 67.5)	60.6 (1.3 to 179.8)	14.7 (-17.7 to 61.1)
	YLDs	Rate	7.8 (5.2 to 11.1)	15.3 (10.2 to 21.8)	0.2 (0.1 to 0.3)	57.4 (11.2 to 127.5)	30.1 (0 to 67.8)	46.9 (2.6 to 113.5)	29.8 (-0.6 to 67.8)	21.4 (-22.1 to 98.2)	14.7 (-19.6 to 64.1)

Location	Measure	Metric	2010			% Change					
						1990 to 2010			2010 to 2019		
			Both	Female	Male	Both	Female	Male	Both	Female	Male
		Number	134 (90 to 192)	133 (89 to 191)	2 (1 to 2)	207.8 (115.7 to 353.5)	64.5 (25.7 to 116.5)	209.3 (115.8 to 360.4)	64.8 (25.3 to 117.6)	117.1 (36.9 to 263.4)	41.2 (-3.5 to 101.5)
Khorasan-e-Razavi	Incidence	Rate*	10.1 (9 to 11.6)	19.8 (17.6 to 22.7)	0.2 (0.1 to 0.2)	36.7 (-4.4 to 102.7)	48.5 (17.9 to 86.1)	31.4 (-8.7 to 95.4)	49.5 (18.9 to 87.9)	2.1 (-33 to 59.9)	24.8 (-13.2 to 78.2)
		Number†	499 (440 to 573)	495 (437 to 569)	4 (3 to 5)	162 (85.2 to 288.8)	98.1 (56.6 to 149.9)	163 (85 to 292.2)	98.3 (56.6 to 150.6)	76.9 (12.3 to 178.4)	62.5 (12.4 to 132.2)
	Prevalence	Rate	92.2 (82.7 to 104.2)	180.8 (162.2 to 204.5)	1.6 (1.2 to 2)	31.4 (1.6 to 73.2)	47.6 (21.8 to 79.4)	26.2 (-3 to 66.8)	48.5 (22.3 to 81)	1.4 (-24.9 to 38)	31.8 (-2.4 to 75.7)
		Number	4480 (3992 to 5076)	4446 (3962 to 5042)	34 (27 to 43)	158.1 (94.7 to 248.7)	97.8 (61.1 to 144.3)	158.9 (94.8 to 250.7)	98.1 (61.2 to 145.1)	85.2 (30.8 to 158.9)	68.3 (24.5 to 122.3)
	Deaths	Rate	4.8 (4.3 to 5.4)	9.3 (8.3 to 10.4)	0.1 (0.1 to 0.1)	2.9 (-29.1 to 52.8)	18.3 (-4.3 to 44.7)	-1.1 (-32.2 to 48.1)	18.7 (-4.2 to 45.2)	-15 (-43.2 to 29.2)	3.6 (-25.2 to 43.4)
		Number	213 (192 to 241)	211 (189 to 238)	2 (2 to 3)	94.2 (35.6 to 189)	57.6 (27.6 to 93.5)	94.8 (35.4 to 191)	57.8 (27.5 to 94)	49.1 (-4.1 to 132.8)	38.3 (-0.2 to 92)
	DALYs	Rate	150.2 (135 to 169)	294.1 (264.3 to 331)	2.9 (2.3 to 3.4)	3.4 (-27.7 to 53.4)	18.3 (-3.6 to 45)	-0.9 (-30.9 to 47.7)	19.3 (-2.8 to 46.7)	-17.3 (-45.7 to 25.2)	5 (-24.3 to 44.3)
		Number	7518 (6764 to 8465)	7453 (6699 to 8393)	64 (53 to 77)	93.6 (36 to 192.4)	57.2 (26.5 to 93.1)	94.2 (36.1 to 194.8)	57.3 (26.6 to 93.7)	39.7 (-8.2 to 120.3)	34.2 (-1.9 to 84.2)
	YLLs	Rate	143.5 (128.6 to 162.2)	280.9 (251.3 to 317.5)	2.7 (2.2 to 3.3)	2.3 (-28.2 to 52)	16.9 (-5.7 to 44.3)	-2 (-31.8 to 46.2)	17.9 (-5.1 to 45.6)	-18.1 (-46.7 to 25.1)	3.7 (-25.5 to 43.9)
		Number	7183 (6428 to 8113)	7122 (6365 to 8029)	62 (50 to 74)	91.2 (34 to 190.2)	55.3 (24.7 to 91.8)	91.8 (34.5 to 192.5)	55.5 (24.5 to 92.3)	38.2 (-9.6 to 117.1)	32.7 (-4.4 to 83.5)
	YLDs	Rate	6.7 (4.7 to 9.3)	13.2 (9.2 to 18.3)	0.1 (0.1 to 0.2)	36.1 (-1.4 to 93.2)	48.4 (17.4 to 91.2)	30.8 (-5.5 to 87.5)	49.3 (18.2 to 92.7)	5 (-28.5 to 55.1)	30.6 (-7.5 to 83.8)

Location	Measure	Metric	2010			% Change					
						1990 to 2010			2010 to 2019		
			Both	Female	Male	Both	Female	Male	Both	Female	Male
		Number	335 (234 to 464)	332 (232 to 460)	3 (2 to 4)	164.2 (89.2 to 282.5)	97.3 (52.3 to 158.5)	165.1 (89 to 285.8)	97.6 (52.5 to 159.2)	85.6 (22 to 178.5)	67.5 (17.8 to 132.5)
Khuzestan	Incidence	Rate*	12.3 (10.6 to 14)	24.5 (21.2 to 28)	0.2 (0.2 to 0.3)	55.6 (4.9 to 128.1)	40.3 (9.7 to 80.6)	54.5 (3.7 to 128.3)	40.4 (9.4 to 81)	-6.3 (-41.3 to 54.3)	18.2 (-22.1 to 73.2)
		Number†	421 (364 to 482)	417 (361 to 479)	3 (3 to 4)	249.8 (139.1 to 416)	88.1 (46 to 145.4)	252.3 (139.1 to 421.7)	88.4 (46 to 146.1)	86.6 (15.6 to 208)	60.5 (4.7 to 133)
	Prevalence	Rate	111 (97.7 to 125.5)	221.6 (195 to 251.1)	2.1 (1.6 to 2.6)	45.1 (9.8 to 91.7)	40 (13 to 74.3)	44.3 (8 to 92.3)	40 (12.7 to 74.5)	-0.7 (-31.5 to 42.5)	23.2 (-11.1 to 68.1)
		Number	3738 (3278 to 4257)	3708 (3245 to 4226)	30 (23 to 37)	235.1 (147.2 to 352.7)	89 (50.7 to 141.1)	236.9 (147.9 to 354.7)	89.2 (50.6 to 141.7)	102.5 (36 to 197.2)	64.1 (17 to 124.6)
	Deaths	Rate	5.4 (4.8 to 6.1)	10.8 (9.6 to 12.1)	0.1 (0.1 to 0.2)	17.8 (-20.4 to 72)	14.2 (-9.4 to 41)	18.3 (-20.4 to 74)	13.7 (-10 to 40.9)	-22.8 (-50.6 to 23.5)	-0.5 (-28.8 to 42.6)
		Number	165 (145 to 185)	163 (144 to 182)	2 (2 to 2)	158.1 (75.6 to 274.3)	54 (21.6 to 92)	160.1 (75.9 to 280.7)	54.2 (21.2 to 93.3)	55.1 (-1.9 to 152.1)	36.8 (-2.1 to 94.8)
	DALYs	Rate	175.5 (155.5 to 197.2)	349.5 (309.8 to 392.7)	3.8 (3 to 4.7)	20.3 (-17.4 to 71.5)	13.6 (-10.1 to 40.9)	18.4 (-18.7 to 71.1)	13.9 (-9.8 to 41.3)	-24.3 (-51.9 to 22.4)	1.3 (-27.6 to 43.9)
		Number	6113 (5417 to 6859)	6056 (5357 to 6792)	58 (47 to 71)	164.8 (80.9 to 279.1)	51.5 (20 to 88.3)	166.8 (82.1 to 285.1)	51.7 (20.1 to 89.3)	48.4 (-3.7 to 141.3)	35 (-3.1 to 87.7)
	YLLs	Rate	167.3 (147.8 to 187.9)	333.3 (294.2 to 374.4)	3.6 (2.9 to 4.4)	19.1 (-18.7 to 71)	12.3 (-12.1 to 40.7)	17.2 (-20 to 70.9)	12.6 (-12 to 41.5)	-25.2 (-52.6 to 22.7)	0.2 (-28.9 to 43.9)
		Number	5833 (5128 to 6536)	5777 (5074 to 6479)	55 (45 to 68)	161.8 (77.6 to 277.4)	49.8 (16.8 to 87.8)	163.8 (78.5 to 282.9)	49.9 (16.8 to 88.8)	46.7 (-5.2 to 140.8)	33.6 (-4.7 to 88.6)
	YLDs	Rate	8.1 (5.6 to 11.4)	16.2 (11.1 to 22.7)	0.2 (0.1 to 0.3)	52.4 (7.4 to 115.6)	41.1 (9.7 to 79.1)	51.4 (5.9 to 116.8)	41.2 (9.6 to 79.8)	-1 (-35.9 to 55.6)	22.8 (-16.8 to 76.5)

Location	Measure	Metric	2010			% Change					
						1990 to 2010			2010 to 2019		
			Both	Female	Male	Both	Female	Male	Both	Female	Male
		Number	281 (193 to 395)	278 (191 to 392)	3 (2 to 4)	246.1 (138.6 to 406.3)	88.8 (45.3 to 146.8)	248.5 (138.8 to 413.5)	89 (45.5 to 147.6)	98.6 (24.8 to 215.9)	64.5 (10.2 to 134.5)
Kohgiluyeh and Boyer-Ahmad	Incidence	Rate*	8.1 (6.5 to 10)	16.3 (13.1 to 20.3)	0.4 (0.3 to 0.5)	58.8 (0 to 164.5)	45.5 (3.2 to 102.7)	56.7 (-2.6 to 165)	47.9 (4.7 to 107.2)	8.1 (-39.1 to 91.8)	-2 (-38.3 to 51.8)
		Number†	42 (34 to 52)	41 (33 to 51)	1 (1 to 1)	317 (164.4 to 592.8)	84.3 (29.4 to 162.5)	324.5 (164.5 to 636.7)	85.7 (29.9 to 165)	134.2 (29 to 322.9)	22.8 (-22.3 to 89.8)
	Prevalence	Rate	75.8 (63 to 90.1)	152.7 (126.4 to 181.4)	3.4 (2.6 to 4.3)	36 (0.5 to 87.3)	47.3 (10.6 to 95)	34 (-2 to 85.7)	48.9 (11.7 to 98.3)	15.8 (-26.4 to 78.4)	6.2 (-26.4 to 51.3)
		Number	387 (318 to 464)	379 (311 to 456)	8 (6 to 10)	272.4 (167.2 to 433.2)	86.1 (37.3 to 154.3)	276 (167.1 to 447.6)	87.3 (37.7 to 156.6)	159.2 (59.4 to 315.7)	29.7 (-9.9 to 89.2)
	Deaths	Rate	3.4 (2.8 to 4.1)	6.9 (5.6 to 8.5)	0.2 (0.2 to 0.3)	6.8 (-33.8 to 71.8)	20.3 (-11.2 to 63.2)	9.2 (-33.3 to 83.7)	22.6 (-10.1 to 66.6)	-14.3 (-47.8 to 49.5)	-19.4 (-47.8 to 19)
		Number	15 (13 to 19)	15 (12 to 18)	0 (0 to 1)	183.3 (81.1 to 355.1)	49.9 (10.2 to 103.3)	187.9 (80.5 to 379.3)	51.4 (10.8 to 106.5)	90.4 (13.1 to 235.6)	2.9 (-32.6 to 56.1)
	DALYs	Rate	106 (86.1 to 128.5)	210.6 (170.6 to 256.6)	6 (4.7 to 7.7)	16.6 (-24.7 to 84.6)	18.3 (-11.6 to 59.7)	12.9 (-28 to 87.1)	20.3 (-10.5 to 62.5)	-18 (-50.8 to 41.1)	-16.3 (-44.9 to 25.7)
		Number	557 (455 to 682)	542 (441 to 667)	15 (12 to 19)	190.5 (88.1 to 362.3)	49.2 (9.5 to 101)	196.4 (88.6 to 389.4)	50.5 (10.2 to 104.3)	68.2 (0.4 to 196)	4.4 (-31 to 55.6)
	YLLs	Rate	100.4 (81.2 to 123)	199.4 (160.7 to 245)	5.7 (4.4 to 7.3)	15.1 (-26.1 to 85.2)	16.8 (-15.2 to 60.6)	11.4 (-29.7 to 86.9)	18.7 (-13.7 to 63.3)	-19.2 (-51.7 to 40)	-17.3 (-46.4 to 25.5)
		Number	528 (420 to 649)	513 (405 to 634)	14 (11 to 18)	186 (85 to 360.7)	47.3 (6 to 102.4)	191.9 (83.9 to 383.9)	48.5 (6.2 to 105.2)	65.4 (-2.1 to 192.9)	3.3 (-32.8 to 56.5)
	YLDs	Rate	5.6 (3.6 to 7.8)	11.1 (7.1 to 15.6)	0.3 (0.2 to 0.4)	51.9 (2.4 to 131.7)	45.5 (4.9 to 99.6)	49.2 (-0.9 to 130.9)	47.8 (5.9 to 103.3)	15.5 (-32.8 to 95.6)	2.7 (-32.5 to 54.2)

Location	Measure	Metric	2010			% Change					
						1990 to 2010			2010 to 2019		
			Both	Female	Male	Both	Female	Male	Both	Female	Male
		**Number**	29 (18 to 41)	28 (18 to 40)	1 (0 to 1)	303.7 (171.7 to 539.8)	84 (29.1 to 156.9)	309.8 (172.4 to 568.3)	85.5 (29.5 to 160.8)	153.8 (45.1 to 339.3)	26.4 (-17.6 to 90.6)
**Kurdistan**	**Incidence**	**Rate***	10.8 (9.4 to 12.5)	21.5 (18.7 to 24.8)	0.2 (0.2 to 0.3)	47.2 (1.1 to 111.1)	26.4 (-1.4 to 57)	38.2 (-5.4 to 98.9)	26.8 (-1 to 58.2)	13.5 (-30.2 to 86.8)	2.4 (-31.1 to 48.4)
		**Number†**	131 (114 to 150)	129 (113 to 149)	1 (1 to 2)	174.3 (88.4 to 291.1)	76.1 (37.9 to 120.9)	175.4 (88.2 to 297.2)	76.5 (38.2 to 121.9)	98 (21.5 to 227)	38.1 (-8.4 to 98.8)
	**Prevalence**	**Rate**	96.2 (84.9 to 109.3)	190.9 (168.8 to 216.6)	2 (1.6 to 2.5)	39.5 (5.8 to 78.9)	29.8 (5.5 to 56.8)	30 (-2.6 to 67.6)	30.1 (6 to 57.5)	12.9 (-21.1 to 58.8)	12.8 (-17.1 to 50.7)
		**Number**	1151 (1015 to 1309)	1140 (1006 to 1298)	11 (9 to 14)	165.3 (96.5 to 245.4)	81.1 (45.9 to 121.2)	166.1 (96.4 to 248.4)	81.4 (45.8 to 121.6)	103.1 (39.5 to 187.4)	49.5 (9.5 to 101.3)
	**Deaths**	**Rate**	5.3 (4.7 to 6)	10.6 (9.2 to 12)	0.1 (0.1 to 0.2)	16.7 (-19.7 to 64.6)	1.9 (-18.2 to 25.3)	9.2 (-25.4 to 56.4)	1.9 (-18.4 to 25.6)	-7.4 (-39.5 to 50.7)	-13.5 (-40.8 to 19.9)
		**Number**	60 (53 to 67)	59 (52 to 67)	1 (1 to 1)	119.6 (52.6 to 208.4)	39.5 (11.9 to 72.6)	120.5 (52.8 to 211.8)	39.8 (11.6 to 72.9)	67.1 (8.4 to 169)	17.8 (-19.3 to 62.7)
	**DALYs**	**Rate**	159.6 (140.2 to 181.7)	315.4 (276.8 to 359.1)	3.8 (3.1 to 4.8)	13.2 (-20 to 57.5)	1.8 (-18.2 to 26)	6.3 (-25.3 to 48.8)	2.1 (-18.1 to 26.9)	-5.2 (-38.5 to 51.3)	-13.7 (-40.6 to 19.1)
		**Number**	1954 (1710 to 2216)	1931 (1687 to 2191)	22 (18 to 28)	106 (46.4 to 184.4)	41.2 (13 to 75.7)	106.7 (45.8 to 188.1)	41.5 (12.8 to 76.3)	60.1 (6.2 to 152.9)	15.3 (-19.4 to 58.5)
	**YLLs**	**Rate**	152.5 (133.2 to 173.3)	301.4 (262.8 to 342.6)	3.6 (2.9 to 4.6)	12.1 (-21 to 57.6)	0.5 (-20.5 to 25.5)	5.3 (-26.5 to 48.4)	0.9 (-20.3 to 26)	-6.1 (-39.4 to 51)	-14.7 (-41.8 to 18.8)
		**Number**	1868 (1629 to 2128)	1847 (1606 to 2108)	21 (17 to 27)	103.7 (44 to 183.2)	39.5 (10.2 to 73.4)	104.4 (43.6 to 186.6)	39.8 (10.2 to 73.9)	58.5 (4.3 to 152.6)	14 (-21.5 to 57.1)
	**YLDs**	**Rate**	7 (4.8 to 9.7)	14 (9.5 to 19.2)	0.2 (0.1 to 0.3)	44.5 (3.9 to 101.6)	29.4 (0.1 to 62.7)	35.5 (-3 to 91.2)	29.9 (-0.1 to 63.5)	17.5 (-25.3 to 78.3)	8.2 (-24.9 to 53.1)

Location	Measure	Metric	2010			% Change					
						1990 to 2010			2010 to 2019		
			Both	Female	Male	Both	Female	Male	Both	Female	Male
		Number	86 (58 to 119)	85 (57 to 118)	1 (1 to 1)	171.4 (93.1 to 289.7)	79.9 (37.9 to 128)	172.4 (92.9 to 293.2)	80.3 (38 to 129.3)	107.3 (31.2 to 218.6)	44.1 (-0.9 to 104)
Lorestan	Incidence	Rate[*]	10.7 (9.1 to 12.6)	21.1 (17.8 to 24.7)	0.3 (0.2 to 0.4)	48.3 (0.7 to 120.4)	24.1 (-6.5 to 64.1)	38.5 (-6.6 to 106.2)	23.6 (-7.1 to 63.8)	18.3 (-27.8 to 106.9)	-2.8 (-35.3 to 41.9)
		Number[†]	146 (122 to 172)	144 (120 to 170)	2 (1 to 3)	177.4 (88.8 to 310.9)	63.7 (22.6 to 119.9)	179 (88.8 to 317.4)	64.3 (22.6 to 120.8)	93.6 (17.3 to 239.1)	23 (-18.3 to 79.8)
	Prevalence	Rate	98.9 (85 to 113.8)	193.9 (166.4 to 222.6)	2.6 (2 to 3.3)	43.3 (7.2 to 88.2)	27.4 (1.3 to 61)	33.2 (-0.8 to 76.1)	26.7 (0.8 to 60.7)	21.1 (-18.2 to 80.9)	6 (-23.7 to 45.9)
		Number	1326 (1131 to 1537)	1310 (1114 to 1521)	16 (13 to 21)	172.7 (99.8 to 266.5)	68 (32.6 to 116)	173.8 (98.9 to 270.4)	68.4 (32.6 to 117.5)	104.9 (34.3 to 217.2)	31.8 (-5.9 to 82.2)
	Deaths	Rate	4.8 (4.1 to 5.6)	9.5 (8.1 to 11.1)	0.2 (0.1 to 0.2)	8.6 (-27.5 to 62.4)	-1.6 (-26.7 to 29)	2.1 (-32.3 to 53.6)	-3.3 (-28.1 to 27.9)	-5.3 (-40.9 to 56.4)	-19.2 (-45.2 to 17)
		Number	60 (52 to 71)	59 (51 to 69)	1 (1 to 1)	104.8 (38.2 to 203)	29 (-4 to 69.1)	105.8 (37.8 to 207.8)	29.5 (-4.5 to 70.5)	60.8 (-1.4 to 168.6)	1.9 (-30.7 to 47.7)
	DALYs	Rate	146.5 (125.6 to 172.4)	285.1 (243.8 to 335.5)	4.7 (3.7 to 6)	9.4 (-26.6 to 59)	0.1 (-25 to 31.5)	1.3 (-32.1 to 50.1)	0.2 (-25.2 to 32)	-9.4 (-44.1 to 49.9)	-16.9 (-42.8 to 20.3)
		Number	2017 (1707 to 2381)	1986 (1676 to 2348)	30 (24 to 38)	98.3 (34.8 to 190.3)	31.4 (-1.8 to 72.9)	99.5 (34.3 to 195.1)	31.8 (-1.7 to 73.5)	42.4 (-11.3 to 137)	5.2 (-27.9 to 52.7)
	YLLs	Rate	139.3 (119.1 to 164.8)	271.1 (230.9 to 319.9)	4.5 (3.5 to 5.7)	8 (-27.5 to 59.7)	-1.3 (-27.1 to 31.4)	0 (-32.9 to 50)	-1.2 (-27.2 to 32)	-10.7 (-44.8 to 49)	-17.9 (-44.3 to 20.2)
		Number	1919 (1619 to 2276)	1890 (1586 to 2251)	29 (23 to 37)	95.5 (31.8 to 188.5)	29.6 (-4.2 to 72.7)	96.6 (30.7 to 193.8)	30 (-4.1 to 74)	40.2 (-14 to 134.9)	4 (-29.1 to 52.3)
	YLDs	Rate	7.2 (4.9 to 10.1)	14 (9.6 to 19.8)	0.2 (0.1 to 0.3)	47.9 (3.3 to 110)	26.9 (-2.7 to 66.8)	38 (-4.4 to 97.8)	26.5 (-3.3 to 66.1)	24.4 (-21.8 to 104.5)	2.3 (-31.1 to 47.9)

Location	Measure	Metric	2010			% Change					
						1990 to 2010			2010 to 2019		
			Both	Female	Male	Both	Female	Male	Both	Female	Male
		Number	98 (67 to 141)	96 (66 to 139)	1 (1 to 2)	177.9 (93.2 to 300)	66.7 (26.2 to 122.4)	179.3 (92.9 to 304.4)	67.3 (26.5 to 124.1)	106.3 (27.4 to 246.7)	27.9 (-13.9 to 83.6)
Markazi	Incidence	Rate*	11.8 (10.2 to 13.7)	23.2 (20.1 to 27)	0.3 (0.2 to 0.4)	32.9 (-6.1 to 95.5)	30.8 (3.4 to 65.6)	32.5 (-6 to 95.8)	31.9 (3.7 to 67.4)	25.5 (-21.7 to 115.5)	3 (-31.7 to 49.3)
		Number†	155 (135 to 181)	154 (133 to 179)	2 (1 to 2)	152 (77.6 to 268.2)	63.9 (28.7 to 107.7)	152.4 (77.6 to 270)	64.3 (28.5 to 108.3)	121.8 (36.3 to 273.5)	30.3 (-12 to 88.5)
	Prevalence	Rate	109.1 (96.5 to 123.9)	214.4 (189.5 to 243.9)	2.3 (1.9 to 3)	31.3 (1.3 to 73.3)	30.4 (6.9 to 59.5)	30 (-0.2 to 72.9)	31.6 (7.7 to 61.3)	23.4 (-13.9 to 80.4)	11.3 (-19.8 to 52.5)
		Number	1421 (1257 to 1626)	1406 (1242 to 1613)	14 (12 to 18)	150.4 (90.6 to 238.2)	63.9 (33.9 to 101.9)	150.7 (89.9 to 240.5)	64.2 (34 to 102.5)	127.4 (54.7 to 239.2)	38.3 (0.7 to 89.5)
	Deaths	Rate	5.2 (4.6 to 5.9)	10.3 (9 to 11.5)	0.2 (0.1 to 0.2)	-0.1 (-30.7 to 45.3)	8.9 (-12 to 36.6)	-0.8 (-31.5 to 45.5)	9.7 (-11.7 to 38.2)	0.2 (-34.4 to 59.3)	-11.4 (-38.3 to 24)
		Number	65 (57 to 73)	64 (57 to 72)	1 (1 to 1)	93 (36.8 to 180.3)	37.9 (10.9 to 73.1)	93.1 (36.2 to 182.8)	38.3 (10.7 to 74.2)	86 (19.3 to 201.5)	15.6 (-19.4 to 61.2)
	DALYs	Rate	163.2 (142.5 to 184.1)	320.1 (279.7 to 361.4)	4.4 (3.6 to 5.5)	1 (-28.2 to 44.9)	7.3 (-13.8 to 34.8)	0.4 (-28.9 to 44.8)	8.6 (-12.8 to 37)	1.6 (-34.6 to 64.2)	-11.7 (-38.1 to 23.2)
		Number	2153 (1885 to 2429)	2125 (1859 to 2401)	27 (22 to 34)	85 (31.9 to 165.5)	35 (8.2 to 70.8)	85.2 (31.3 to 167.4)	35.3 (8.1 to 71.6)	73.2 (10.9 to 176.5)	10.9 (-22.7 to 54.8)
	YLLs	Rate	155.4 (135.6 to 175.4)	304.6 (265.8 to 344.3)	4.2 (3.4 to 5.3)	-0.2 (-29.3 to 43.8)	6.1 (-15.4 to 34.9)	-0.8 (-30.2 to 43.8)	7.4 (-14.9 to 37.3)	0.6 (-35.4 to 63.3)	-12.6 (-39.5 to 23)
		Number	2049 (1791 to 2312)	2022 (1767 to 2287)	26 (21 to 33)	82.5 (29.5 to 163.1)	33.5 (5.8 to 70.8)	82.7 (29 to 165.5)	33.8 (5.8 to 71.8)	71.1 (8.9 to 177.9)	9.8 (-24.3 to 54.3)
	YLDs	Rate	7.9 (5.2 to 11.2)	15.5 (10.3 to 22)	0.2 (0.1 to 0.3)	33.4 (-4.7 to 95.5)	31.6 (1.4 to 68.1)	32.7 (-5.2 to 96.1)	32.8 (2 to 69.6)	28.8 (-17.5 to 108.6)	7.7 (-25.9 to 55.8)

Location	Measure	Metric	2010			% Change					
						1990 to 2010			2010 to 2019		
			Both	Female	Male	Both	Female	Male	Both	Female	Male
		**Number**	104 (69 to 148)	103 (68 to 146)	1 (1 to 2)	153.2 (78.3 to 273.9)	64.4 (26 to 110.5)	153.5 (78 to 277.3)	64.8 (26.2 to 111.3)	131.4 (46.8 to 271.9)	34.3 (-7.9 to 91.7)
**Mazandaran**	**Incidence**	**Rate***	14.9 (12.7 to 17.2)	29.3 (25 to 33.9)	0.3 (0.2 to 0.3)	51.8 (-0.2 to 130.9)	39.7 (8.4 to 79.4)	50.7 (-1.5 to 131.3)	40.5 (9 to 80.9)	16.5 (-27.1 to 98.1)	12.8 (-26.3 to 65)
		**Number†**	462 (393 to 535)	459 (388 to 532)	4 (3 to 5)	217 (108.7 to 381.6)	90.6 (47.8 to 147.3)	217.8 (108.4 to 386.2)	90.9 (47.7 to 148.1)	133.1 (42.4 to 297.6)	56.3 (0 to 128.3)
	**Prevalence**	**Rate**	140.1 (122.1 to 158.2)	275.8 (239.9 to 311.5)	2.3 (1.8 to 3)	45.7 (5.8 to 98.8)	36.6 (9.6 to 69.9)	44.9 (5.4 to 98.8)	37.2 (9.9 to 71.1)	14.7 (-20.2 to 66.6)	17.3 (-16.3 to 61.7)
		**Number**	4277 (3701 to 4888)	4246 (3672 to 4859)	31 (25 to 40)	211.7 (122.3 to 331.4)	88 (50.4 to 136.9)	212.5 (122.3 to 332.6)	88.2 (50.3 to 137.4)	134.6 (57.7 to 255.9)	60.6 (12.9 to 123.2)
	**Deaths**	**Rate**	5.3 (4.7 to 6)	10.4 (9.1 to 11.7)	0.1 (0.1 to 0.2)	12.2 (-28.4 to 67.7)	16.4 (-6 to 46.3)	12.3 (-28.5 to 69.8)	16.6 (-6.3 to 47.1)	-6.8 (-40.5 to 54.4)	-2 (-33.4 to 40.6)
		**Number**	152 (133 to 171)	150 (131 to 169)	2 (1 to 2)	141.1 (56.2 to 258.4)	62.9 (29.5 to 104.9)	141.7 (55.6 to 261.6)	63.2 (29.3 to 105.8)	98.5 (22.5 to 233.6)	38.6 (-5.5 to 98.7)
	**DALYs**	**Rate**	173.6 (151.3 to 195.8)	340.5 (296.6 to 384.3)	3.5 (2.8 to 4.4)	15.7 (-24 to 70.2)	17.7 (-5.9 to 47.6)	14.1 (-25.6 to 68.2)	18.5 (-5.5 to 48.7)	-6.6 (-40.3 to 54.8)	-2.3 (-32.1 to 37.4)
		**Number**	5409 (4707 to 6114)	5360 (4659 to 6063)	49 (39 to 61)	136.2 (55.4 to 247.6)	61.2 (28 to 102.2)	136.9 (55.2 to 249.6)	61.5 (27.9 to 103.2)	77.9 (12.4 to 197.2)	33.8 (-7.4 to 89.2)
	**YLLs**	**Rate**	163.5 (142.2 to 185.6)	320.8 (279 to 364.1)	3.3 (2.7 to 4.1)	14 (-25.9 to 70.7)	16.4 (-8 to 47.6)	12.5 (-27.5 to 67.9)	17.2 (-7.9 to 48.9)	-7.9 (-41.8 to 54.5)	-3.4 (-33.9 to 36.8)
		**Number**	5097 (4428 to 5803)	5051 (4385 to 5758)	47 (37 to 59)	132.6 (51.3 to 246.6)	59.5 (24.6 to 103.1)	133.3 (51 to 248.4)	59.8 (24.3 to 104)	75.2 (10 to 196.6)	32.3 (-10 to 89.1)
	**YLDs**	**Rate**	10.1 (6.7 to 14.1)	19.8 (13.3 to 27.7)	0.2 (0.1 to 0.3)	50.1 (4.9 to 119.5)	38.7 (6.6 to 81)	49.2 (3.7 to 119.7)	39.5 (6.9 to 82.4)	19.6 (-22.5 to 95.2)	15.7 (-22.4 to 65.7)

Location	Measure	Metric	2010			% Change					
						1990 to 2010			2010 to 2019		
			Both	Female	Male	Both	Female	Male	Both	Female	Male
		Number	312 (207 to 434)	309 (205 to 431)	3 (2 to 4)	214.8 (116.9 to 365.2)	89.3 (44.1 to 150.5)	215.7 (116.7 to 367.3)	89.6 (44 to 151.3)	140.6 (50.6 to 294.5)	58.5 (5.2 to 128.4)
North Khorasan	Incidence	Rate*	9.1 (8 to 10.3)	17.6 (15.4 to 20)	0.4 (0.3 to 0.5)	35.9 (-3 to 90.6)	48.9 (19.7 to 82.8)	30.4 (-7.4 to 86.9)	50.7 (21.1 to 85.6)	5.4 (-34.5 to 80.5)	-4.4 (-34.7 to 35.6)
		Number†	62 (54 to 70)	61 (53 to 69)	1 (1 to 1)	174.2 (98.5 to 279.7)	85 (48.2 to 126.2)	176.3 (98.9 to 287.9)	86.3 (49.4 to 128.4)	94.3 (19.3 to 238.4)	16.6 (-20.2 to 64.1)
	Prevalence	Rate	83.5 (74.1 to 92.9)	161.9 (143.5 to 180.7)	2.9 (2.3 to 3.7)	31.4 (4.2 to 65.5)	47.1 (23.3 to 74.7)	25.4 (-1.1 to 59.7)	48.4 (24 to 76.6)	15.9 (-20.9 to 75.3)	3.3 (-24.9 to 39.2)
		Number	562 (496 to 631)	553 (487 to 622)	9 (7 to 11)	175.5 (114.1 to 254.4)	83.6 (52 to 118.7)	176.7 (113.8 to 258.2)	84.5 (52.3 to 120.4)	118.3 (45.2 to 236.7)	24.5 (-10.2 to 68.7)
	Deaths	Rate	4.3 (3.9 to 4.9)	8.4 (7.5 to 9.5)	0.2 (0.2 to 0.3)	-3.3 (-30.2 to 39.8)	23.7 (0 to 49)	-6.3 (-33.1 to 37.5)	24.6 (1 to 50)	-17.6 (-46.4 to 36.7)	-16.3 (-39.6 to 13.9)
		Number	27 (25 to 31)	27 (24 to 30)	1 (1 to 1)	97.5 (44.1 to 178.4)	53.8 (25 to 85.1)	98.8 (44.2 to 184.2)	55 (25.5 to 87.2)	57.3 (0.9 to 167.4)	3.8 (-24.5 to 39.9)
	DALYs	Rate	136.8 (121.9 to 153.5)	262.6 (233.6 to 294.6)	6.1 (5 to 7.6)	-2.9 (-27.6 to 34.9)	25.5 (2 to 51.5)	-7.4 (-31.6 to 30.8)	27.5 (3.6 to 54.5)	-19 (-48.1 to 36.9)	-15.7 (-38.7 to 14.1)
		Number	949 (844 to 1063)	929 (824 to 1043)	20 (16 to 24)	89.2 (40.2 to 160.5)	55.4 (26.3 to 88)	90.5 (39.8 to 165.8)	56.5 (26.7 to 89.6)	43.6 (-8.1 to 140)	1.9 (-25.5 to 38.6)
	YLLs	Rate	130.7 (116.3 to 146.6)	250.8 (222.9 to 281.8)	5.8 (4.7 to 7.2)	-4.3 (-29.2 to 34)	24.4 (0.8 to 50.9)	-8.7 (-33.1 to 30.5)	26.4 (2.5 to 53.3)	-20.1 (-48.9 to 35.2)	-16.4 (-39.6 to 13.7)
		Number	907 (803 to 1021)	888 (785 to 1001)	19 (15 to 23)	86.4 (37.1 to 157.8)	54 (24.7 to 87.6)	87.6 (36.9 to 164.2)	55.1 (25.5 to 89.3)	41.6 (-9.9 to 137.9)	1 (-26.2 to 38.1)
	YLDs	Rate	6.1 (4.2 to 8.4)	11.8 (8.1 to 16.2)	0.3 (0.2 to 0.4)	37.7 (2 to 84.9)	48.7 (18.3 to 88.4)	31.9 (-3.5 to 80.9)	50.5 (19.6 to 90.9)	14.4 (-28.2 to 86.9)	0.3 (-30.2 to 40.7)

Location	Measure	Metric	2010			% Change					
						1990 to 2010			2010 to 2019		
			Both	Female	Male	Both	Female	Male	Both	Female	Male
		Number	42 (28 to 58)	41 (28 to 57)	1 (1 to 1)	181.3 (107.2 to 284.2)	84.6 (46.1 to 136.2)	183.2 (106.8 to 289.7)	85.8 (46.4 to 137.8)	112.3 (31.4 to 253.4)	21 (-16.7 to 70.2)
Qazvin	Incidence	Rate*	11.9 (10.5 to 13.6)	23.5 (20.6 to 26.8)	0.3 (0.2 to 0.4)	67.5 (14 to 158.9)	29.7 (1.2 to 60.2)	65.4 (12.2 to 157.4)	31 (1.8 to 62.5)	21.7 (-25.2 to 105.5)	3.6 (-29 to 52.7)
		Number†	119 (103 to 136)	118 (102 to 135)	1 (1 to 2)	244.6 (134.3 to 436.5)	86.2 (44.3 to 133.1)	246.6 (135.1 to 446)	86.7 (44.5 to 134.2)	121.8 (36.5 to 273.5)	40 (-5.1 to 104.8)
	Prevalence	Rate	106.3 (93.6 to 119.3)	209.8 (184.5 to 236.3)	2.5 (1.9 to 3.2)	54.3 (18.2 to 105.5)	33.3 (7.7 to 60.8)	52.3 (16.5 to 105.4)	34.5 (8.4 to 62.7)	24 (-15.2 to 81)	10.6 (-18.7 to 52.6)
		Number	1052 (922 to 1189)	1041 (913 to 1178)	11 (9 to 14)	225.2 (143.9 to 349)	90.3 (52.8 to 132.2)	226.6 (144 to 351)	90.8 (52.8 to 133.1)	132.5 (57.4 to 251.6)	47.3 (6.5 to 102.9)
	Deaths	Rate	5.3 (4.7 to 6)	10.4 (9.2 to 11.7)	0.2 (0.1 to 0.2)	19.9 (-22 to 88)	2.2 (-18.4 to 29.3)	18.7 (-22.8 to 88.4)	2.2 (-18.9 to 29.4)	-3.9 (-38.1 to 55.5)	-11.2 (-37.7 to 20.4)
		Number	48 (43 to 54)	48 (42 to 54)	1 (1 to 1)	145.1 (65.5 to 287.1)	45.2 (15.4 to 83.6)	146.5 (65.2 to 291.3)	45.5 (15.2 to 84)	79.5 (15.3 to 193)	19.2 (-15.8 to 63.4)
	DALYs	Rate	161.1 (142.4 to 181.4)	317.8 (279.8 to 358.5)	4.4 (3.5 to 5.5)	20.2 (-16.8 to 86.1)	7.3 (-14.1 to 34.6)	17.9 (-19 to 83.2)	8.7 (-13.2 to 36.2)	-7.7 (-41.3 to 51)	-10.3 (-36.5 to 22.5)
		Number	1628 (1440 to 1835)	1608 (1420 to 1815)	20 (16 to 25)	139.9 (67.4 to 271.3)	53.9 (23 to 92.2)	141.3 (67.3 to 276.9)	54.3 (23.2 to 93)	63.4 (4.1 to 163.6)	21.1 (-14.6 to 67.2)
	YLLs	Rate	153.3 (135.1 to 172.4)	302.4 (266.2 to 340.6)	4.2 (3.4 to 5.2)	18.6 (-18.3 to 85.8)	6 (-16.1 to 34)	16.3 (-20.2 to 82.7)	7.3 (-15.4 to 35.7)	-9 (-42.5 to 48.9)	-11.2 (-37.5 to 22.2)
		Number	1550 (1365 to 1744)	1530 (1347 to 1726)	19 (15 to 24)	136.4 (64.2 to 268.4)	52.1 (19.7 to 91.3)	137.8 (64.7 to 273)	52.5 (19.7 to 92)	60.9 (2.3 to 160.9)	20 (-16.6 to 66.7)
	YLDs	Rate	7.8 (5.3 to 10.8)	15.4 (10.4 to 21.4)	0.2 (0.1 to 0.3)	62.7 (15.4 to 139.3)	33 (3.4 to 67.2)	60.7 (13.5 to 137.5)	34.4 (4 to 69.2)	27.5 (-18.6 to 103.5)	7.9 (-24.8 to 55.6)

Location	Measure	Metric	2010			% Change					
						1990 to 2010			2010 to 2019		
			Both	Female	Male	Both	Female	Male	Both	Female	Male
		**Number**	79 (53 to 110)	78 (52 to 109)	1 (1 to 1)	238 (134.6 to 411.7)	89.8 (46.7 to 139.6)	239.9 (135.2 to 416.2)	90.3 (46.3 to 140.5)	134.3 (48.8 to 277.4)	44.3 (0 to 106.6)
**Qom**	**Incidence**	**Rate***	15.1 (13.2 to 17.3)	30.6 (26.8 to 35.1)	0.5 (0.4 to 0.6)	53.3 (4.4 to 143.7)	21.4 (-1.5 to 52.9)	54.7 (4.9 to 150.1)	22.3 (-0.9 to 54.3)	-6.4 (-43.4 to 59.6)	3.1 (-30.2 to 50.8)
		**Number†**	136 (119 to 157)	134 (117 to 155)	2 (1 to 2)	273.9 (159.7 to 556.8)	76.7 (43.6 to 124.9)	278.1 (160.5 to 567.7)	77 (43.6 to 126.4)	106.7 (24.6 to 261.3)	54 (3.7 to 127)
	**Prevalence**	**Rate**	132.4 (117.4 to 149.2)	268.5 (237.9 to 302.3)	3.8 (3 to 4.8)	50.1 (14.8 to 114.2)	23.6 (3.4 to 50.8)	50.6 (14.3 to 118.3)	24.4 (3.7 to 52.2)	7.3 (-29.3 to 63.3)	8.9 (-21.2 to 52.8)
		**Number**	1189 (1052 to 1349)	1174 (1036 to 1335)	15 (12 to 19)	272.2 (178.2 to 465.2)	80.1 (48.6 to 123.3)	274.9 (178.7 to 476.9)	80.4 (48.3 to 124.1)	137.8 (53.5 to 277.5)	59.8 (15.2 to 123.2)
	**Deaths**	**Rate**	6.9 (6.2 to 7.8)	14.2 (12.7 to 16)	0.3 (0.2 to 0.4)	11 (-24.4 to 83.6)	-0.4 (-19.2 to 23.2)	14.5 (-22.8 to 95.6)	0.8 (-18.3 to 25.1)	-24.9 (-52.6 to 29.6)	-13.8 (-40.1 to 18.1)
		**Number**	55 (49 to 62)	54 (48 to 61)	1 (1 to 1)	166.4 (84 to 370.5)	46.1 (17.6 to 81.7)	169.4 (83.9 to 375.5)	46.4 (17.4 to 82.6)	67.7 (4.5 to 202.8)	31.1 (-7.6 to 78)
	**DALYs**	**Rate**	213.4 (189.5 to 239.9)	431.2 (382.4 to 485)	7.4 (6 to 9)	11.9 (-22.1 to 104.1)	-1.2 (-19.9 to 22)	11.9 (-22.9 to 106)	-0.5 (-19.7 to 23.4)	-27.6 (-54.5 to 29.6)	-11.6 (-37.5 to 21.1)
		**Number**	1964 (1742 to 2217)	1933 (1712 to 2187)	31 (25 to 38)	165.1 (83.8 to 452.1)	42.8 (15.1 to 77.4)	168.1 (85.3 to 467.5)	43 (14.8 to 78.1)	56.4 (-2 to 176.3)	29.2 (-8.2 to 77.7)
	**YLLs**	**Rate**	203.6 (181 to 230.4)	411.4 (365.3 to 465.6)	7 (5.7 to 8.6)	10.5 (-23 to 101.7)	-2.4 (-21.6 to 21.6)	10.4 (-24.2 to 103.6)	-1.7 (-21.3 to 22.8)	-28.7 (-55.3 to 28.6)	-12.6 (-38.6 to 19.8)
		**Number**	1874 (1655 to 2130)	1845 (1627 to 2096)	30 (24 to 36)	161.5 (80.3 to 448.8)	41.1 (13 to 76.9)	164.4 (80.6 to 464)	41.3 (12.8 to 77.8)	54.2 (-2.8 to 174.7)	27.9 (-10.1 to 75.5)
	**YLDs**	**Rate**	9.8 (7 to 13.5)	19.8 (14 to 27.3)	0.3 (0.2 to 0.5)	53.5 (8.8 to 142.8)	23.9 (-0.6 to 55.6)	54.4 (8.3 to 146.2)	24.6 (-0.5 to 56.5)	2.5 (-36.3 to 70.6)	7.5 (-26.3 to 57.1)

Location	Measure	Metric	2010			% Change					
						1990 to 2010			2010 to 2019		
			Both	Female	Male	Both	Female	Male	Both	Female	Male
		Number	89 (63 to 124)	88 (62 to 123)	1 (1 to 2)	275.6 (167.5 to 538)	79.3 (39.9 to 128.7)	279.5 (168.3 to 545.1)	79.6 (39.6 to 129.7)	126.2 (38.4 to 284.6)	58.3 (9.4 to 131.5)
Semnan	Incidence	Rate*	15.3 (13.1 to 17.6)	29.8 (25.5 to 34.3)	0.6 (0.5 to 0.8)	64.4 (10.4 to 154.5)	21.4 (-5.5 to 54.2)	64.3 (9.8 to 156)	22.9 (-4.9 to 56)	76.9 (8.2 to 205.1)	0.9 (-31.6 to 50.4)
		Number†	86 (74 to 99)	85 (72 to 98)	2 (1 to 2)	211.6 (108.9 to 385.8)	71.4 (32.5 to 117.7)	211.7 (108.5 to 391)	71.9 (32.7 to 118.7)	203.8 (85.9 to 424.6)	40.8 (-6 to 111.4)
	Prevalence	Rate	137.4 (119.9 to 155.5)	268.3 (233.8 to 303.6)	5 (3.9 to 6.5)	56.6 (16.4 to 111)	23.5 (-0.8 to 52.7)	57 (15.7 to 114.5)	24.9 (0.1 to 55)	77.5 (18.6 to 164.9)	7 (-25 to 56.2)
		Number	769 (670 to 876)	756 (659 to 863)	13 (10 to 16)	200.4 (120.8 to 316)	74.3 (38.8 to 116.2)	200.2 (120.2 to 317.4)	74.7 (38.6 to 117.6)	209.8 (102.3 to 385.5)	48.3 (3.2 to 115.8)
	Deaths	Rate	6.4 (5.6 to 7.2)	12.2 (10.7 to 13.8)	0.4 (0.3 to 0.4)	17.4 (-25 to 81.4)	-1.5 (-21.4 to 23.8)	17.7 (-25.3 to 84.6)	-0.7 (-21.4 to 24.7)	37.7 (-11.9 to 131.6)	-12.8 (-38.8 to 20.1)
		Number	33 (29 to 38)	32 (28 to 37)	1 (1 to 1)	122.9 (44.4 to 243.6)	37.9 (10.1 to 73)	122.4 (43.6 to 246.2)	38.3 (10.2 to 74.3)	142.3 (53.9 to 303.5)	20.9 (-14.4 to 67.4)
	DALYs	Rate	195.1 (171 to 220.9)	379.6 (331.1 to 431)	9.4 (7.6 to 11.5)	18.3 (-20 to 76.8)	1.6 (-18.6 to 27.1)	17.6 (-21 to 77.1)	2.8 (-17.7 to 29.1)	34.9 (-13.7 to 124.6)	-12.6 (-37.9 to 19.5)
		Number	1109 (970 to 1254)	1084 (946 to 1230)	24 (20 to 29)	119.3 (49.8 to 227.9)	43.2 (14.9 to 79)	119.2 (48.5 to 229.2)	43.7 (15.1 to 80.2)	127.7 (47.2 to 275.8)	21.5 (-14.6 to 66.8)
	YLLs	Rate	184.9 (161.9 to 209.7)	360 (313.6 to 409.6)	8.9 (7.2 to 10.9)	16.5 (-21.7 to 74.5)	0.4 (-20.4 to 26.3)	15.8 (-22.4 to 76)	1.6 (-19.9 to 28.3)	33.1 (-14.9 to 123.3)	-13.4 (-39.3 to 18.9)
		Number	1051 (919 to 1197)	1028 (898 to 1173)	23 (19 to 28)	115.9 (46.4 to 227.1)	41.6 (12.3 to 78.6)	115.7 (45.3 to 228.1)	42 (12.5 to 79.2)	124.6 (43.4 to 273.8)	20.4 (-16.2 to 66.1)

Location	Measure	Metric	2010			% Change					
						1990 to 2010			2010 to 2019		
			Both	Female	Male	Both	Female	Male	Both	Female	Male
	YLDs	Rate	10.1 (7 to 14)	19.7 (13.6 to 27.2)	0.5 (0.3 to 0.7)	62.9 (12.6 to 146.3)	23.2 (-3.9 to 56.4)	62.8 (11.3 to 148.8)	24.7 (-3.2 to 58.8)	82.5 (14.2 to 203.2)	3.2 (-32.2 to 55.6)
		Number	57 (40 to 80)	56 (39 to 78)	1 (1 to 2)	209.2 (111.8 to 371.2)	73.5 (34.2 to 121.3)	209.1 (110.8 to 374.7)	74.1 (33.8 to 122.5)	214.3 (95.9 to 417.4)	43.3 (-4.1 to 113.5)
Sistan and Baluchistan	Incidence	Rate*	7.5 (6.3 to 8.8)	15 (12.5 to 17.5)	0.2 (0.2 to 0.3)	37.3 (-6.3 to 123.5)	33.2 (0.3 to 77)	25.1 (-15.3 to 105.5)	32.8 (-0.4 to 76.1)	-1 (-42.1 to 84.9)	-6.1 (-38.4 to 35.2)
		Number†	100 (83 to 118)	99 (82 to 116)	1 (1 to 2)	184.8 (93.5 to 384.1)	82 (34.4 to 144.9)	187 (93.7 to 392.6)	82.8 (34.2 to 146.1)	76.1 (2.7 to 270.8)	21.3 (-19.7 to 76.2)
	Prevalence	Rate	66.1 (56.6 to 75.4)	132.1 (113.1 to 151)	1.7 (1.3 to 2.2)	25.7 (-4.9 to 74.5)	36.9 (9.6 to 71.9)	14.6 (-14 to 57.7)	36 (8.9 to 71.1)	1.1 (-30.3 to 50.8)	5.8 (-21.6 to 39.5)
		Number	862 (729 to 989)	852 (720 to 977)	10 (8 to 13)	169 (98.9 to 289)	86.6 (43.8 to 140.6)	170.4 (99.1 to 289.9)	87.2 (43.9 to 141.9)	88.8 (27.4 to 196.2)	33.1 (-2 to 76.8)
	Deaths	Rate	4.3 (3.7 to 5.1)	8.6 (7.4 to 10.2)	0.1 (0.1 to 0.2)	7.4 (-28.2 to 67.5)	13.9 (-14 to 49.7)	-1.2 (-33.9 to 56.5)	12.8 (-14.8 to 48.5)	-14.7 (-47.9 to 50.6)	-16.9 (-44 to 19.3)
		Number	52 (44 to 61)	51 (43 to 60)	1 (1 to 1)	118.9 (47.5 to 246.4)	50.8 (13.2 to 100.8)	120.4 (47 to 256.7)	51.5 (13.1 to 101.8)	52.8 (-9.4 to 195.8)	6.7 (-27.4 to 51.9)
	DALYs	Rate	133 (112.8 to 155.6)	263.7 (223.6 to 308.8)	3.8 (3 to 4.9)	10.8 (-25 to 75.9)	14.2 (-14.3 to 51.4)	0.5 (-32.2 to 60.8)	14 (-14.5 to 51.3)	-18.9 (-51.1 to 55.5)	-15.4 (-42.6 to 22.1)
		Number	1826 (1545 to 2132)	1801 (1520 to 2105)	25 (20 to 31)	124 (51.6 to 264.3)	55.2 (16.1 to 106.7)	125.7 (51.5 to 267.4)	55.8 (16 to 108.5)	43.3 (-16.6 to 190.9)	8.8 (-24.9 to 55.2)
	YLLs	Rate	128.2 (108.7 to 150.9)	254 (215.1 to 298.9)	3.6 (2.8 to 4.7)	10 (-25.6 to 74.3)	13.3 (-15.4 to 51.4)	-0.2 (-33.3 to 59.8)	13.2 (-16 to 51.5)	-19.6 (-52.1 to 55.5)	-16 (-43.8 to 22.1)
		Number	1760 (1487 to 2069)	1736 (1465 to 2040)	24 (19 to 30)	122.2 (48.9 to 262.5)	54 (13.9 to 107.3)	123.9 (48.9 to 264.4)	54.7 (13.9 to 108.9)	42.1 (-17.8 to 191.8)	8.1 (-25.8 to 55.4)

Location	Measure	Metric	2010			% Change					
						1990 to 2010			2010 to 2019		
			Both	Female	Male	Both	Female	Male	Both	Female	Male
	YLDs	Rate	4.9 (3.2 to 6.9)	9.7 (6.4 to 13.8)	0.1 (0.1 to 0.2)	35.2 (-6.2 to 110.5)	37 (1.6 to 83.3)	23.1 (-15.2 to 91.9)	36.6 (1.1 to 83.6)	3.1 (-36.1 to 77.4)	0.9 (-30.7 to 41.6)
		Number	66 (43 to 93)	65 (42 to 92)	1 (1 to 1)	186.4 (97.9 to 367.8)	86.6 (34.6 to 155.8)	188.5 (98.4 to 377.3)	87.4 (34.7 to 157.4)	86.5 (16.1 to 245)	28 (-12.5 to 78.2)
South Khorasan	Incidence	Rate*	11.4 (9.9 to 13)	22 (19.1 to 25)	0.3 (0.2 to 0.4)	41.2 (-2.9 to 106)	35.9 (6.9 to 69.2)	33.1 (-9.2 to 96.1)	36.5 (7.3 to 69.5)	4.7 (-34.3 to 79)	-1.8 (-31.5 to 46.4)
		Number†	62 (54 to 71)	62 (54 to 70)	1 (1 to 1)	97.4 (37.2 to 186)	94 (53.3 to 140.8)	98.4 (37 to 191.6)	94.7 (53.3 to 142.1)	34.7 (-18.4 to 129.8)	33.6 (-6.6 to 96.1)
	Prevalence	Rate	103 (91.8 to 116)	198.9 (177.2 to 224.1)	2.3 (1.9 to 3)	38.1 (6.3 to 78.1)	35.5 (10.5 to 63.7)	29.8 (-0.7 to 68.1)	35.8 (10.6 to 64.7)	10.1 (-24.1 to 61.1)	5.7 (-19.8 to 44.8)
		Number	560 (498 to 630)	553 (492 to 624)	6 (5 to 8)	95.2 (48.9 to 154.9)	93.3 (57.3 to 134)	95.9 (48.9 to 156.7)	93.9 (57.3 to 135.2)	46.8 (-3.2 to 122.2)	42.1 (8.1 to 93.7)
	Deaths	Rate	5.4 (4.7 to 6.1)	10.3 (9.1 to 11.8)	0.2 (0.1 to 0.2)	5.4 (-27.8 to 48.9)	13.7 (-8.6 to 38.6)	-0.5 (-32.4 to 42)	13.2 (-9.5 to 38.1)	-14.7 (-45.7 to 39)	-13.4 (-39.3 to 19.3)
		Number	29 (25 to 32)	28 (25 to 32)	0 (0 to 1)	52.1 (4.8 to 115.2)	58.3 (27.5 to 92.6)	52.8 (4.2 to 118.7)	59 (27.7 to 93.7)	16.3 (-28.9 to 91.3)	16 (-18.1 to 60.1)
	DALYs	Rate	163.6 (145.7 to 185.1)	314.6 (279.4 to 356.2)	4.4 (3.5 to 5.5)	6.3 (-25.6 to 47)	12.5 (-9.1 to 37.1)	-0.2 (-30.3 to 38.9)	13.2 (-8.8 to 38.2)	-16.5 (-47.7 to 33.3)	-15.7 (-40.1 to 17.3)
		Number	892 (795 to 1009)	881 (783 to 997)	11 (9 to 14)	43.1 (1.2 to 96.4)	61.7 (29.5 to 97.4)	43.8 (1.4 to 99.4)	62.3 (29.4 to 98.5)	1.8 (-37.4 to 63.8)	16.7 (-16.3 to 61.6)
	YLLs	Rate	156.2 (138.6 to 176.9)	300.2 (266.3 to 340.5)	4.2 (3.4 to 5.3)	5 (-27.1 to 45.9)	11.4 (-10.8 to 36.5)	-1.4 (-31.8 to 38.2)	12.1 (-10.4 to 37.7)	-17.4 (-48.6 to 33.3)	-16.5 (-41.1 to 17.3)
		Number	851 (755 to 965)	840 (745 to 955)	11 (9 to 14)	41.1 (-0.8 to 96)	60.1 (27.5 to 96.9)	41.9 (-0.8 to 98.2)	60.7 (27.8 to 98.2)	0.4 (-38.4 to 62.5)	15.6 (-17.8 to 62.2)

Location	Measure	Metric	2010			% Change					
						1990 to 2010			2010 to 2019		
			Both	Female	Male	Both	Female	Male	Both	Female	Male
	YLDs	Rate	7.5 (5.1 to 10.4)	14.4 (9.9 to 20.1)	0.2 (0.1 to 0.3)	41.8 (0.5 to 95.9)	36 (6.5 to 73.2)	33.6 (-6 to 86.4)	36.6 (6.6 to 74.1)	10.6 (-29.9 to 78.3)	2.8 (-26.4 to 48.8)
		Number	41 (28 to 58)	41 (28 to 57)	1 (0 to 1)	98.9 (41.8 to 177)	94 (52 to 146.5)	99.9 (41.2 to 180.7)	94.7 (52.1 to 148.3)	44.7 (-10.9 to 139.2)	38.6 (0.2 to 98.7)
Tehran	Incidence	Rate[*]	15.8 (13.5 to 18.6)	31.9 (27.1 to 37.4)	0.3 (0.2 to 0.4)	6 (-26.8 to 55)	27.8 (-2.7 to 66.5)	5.3 (-27.3 to 53.3)	27.1 (-3.6 to 65.8)	-13.1 (-50.4 to 59.6)	12.7 (-26.5 to 69.6)
		Number[†]	1795 (1531 to 2113)	1783 (1515 to 2101)	13 (9 to 17)	139.5 (67.7 to 244.3)	78.6 (34.8 to 135.8)	139.9 (67.1 to 245.8)	78.7 (34.7 to 135.9)	89.9 (8 to 247.8)	64.5 (7 to 144.3)
	Prevalence	Rate	150.3 (130.9 to 172.4)	302.5 (263.3 to 347.5)	2.3 (1.8 to 3)	9 (-18.6 to 44.9)	24.3 (-0.7 to 55.6)	8.4 (-19.8 to 44.4)	23.4 (-1.7 to 54.8)	-9.5 (-42.2 to 42.1)	17.2 (-15.6 to 63.4)
		Number	16799 (14604 to 19433)	16684 (14495 to 19319)	114 (88 to 150)	147 (80.2 to 230.8)	75.5 (38.3 to 124.1)	147.5 (79.8 to 231.5)	75.5 (37.9 to 124.5)	98.8 (22.3 to 218)	68 (18.2 to 135.7)
	Deaths	Rate	6.2 (5.3 to 7.1)	12.4 (10.8 to 14.3)	0.1 (0.1 to 0.2)	-14.2 (-42.2 to 26.3)	9.6 (-13.9 to 38.8)	-12.9 (-41.6 to 28.1)	9.2 (-14.6 to 38.5)	-23.2 (-55.1 to 30)	-4.2 (-35.9 to 39)
		Number	633 (545 to 735)	627 (539 to 728)	6 (5 to 9)	102.1 (38.5 to 190.5)	57 (22.2 to 99.9)	102.5 (38.4 to 191.7)	57.1 (21.6 to 100.4)	75.4 (3.5 to 190.3)	46.9 (-2.7 to 111.6)
	DALYs	Rate	185.7 (160.6 to 214.6)	373 (322.7 to 432.2)	3.4 (2.6 to 4.5)	-11.9 (-38.6 to 24.9)	8.2 (-15.4 to 37.2)	-13.3 (-40.1 to 23.1)	7.5 (-16.3 to 36.3)	-26.9 (-56.2 to 19.2)	2 (-31.4 to 50.7)
		Number	21352 (18464 to 24720)	21174 (18273 to 24542)	177 (134 to 231)	95.1 (35.8 to 177)	50.7 (17 to 92)	95.5 (35.5 to 177.8)	50.7 (16.6 to 92.2)	52.9 (-6.2 to 153.2)	44.6 (-2.8 to 113.5)
	YLLs	Rate	175 (150.8 to 203)	351.4 (302.5 to 407.9)	3.2 (2.4 to 4.3)	-12.9 (-40.5 to 24.8)	7.1 (-17.7 to 37.1)	-14.4 (-41.5 to 22.9)	6.3 (-18.4 to 36.4)	-27.7 (-57 to 19.5)	1.2 (-32.6 to 51.9)
		Number	20133 (17313 to 23353)	19966 (17142 to 23184)	168 (126 to 221)	92.7 (32.7 to 177)	49.1 (13.7 to 91.2)	93.2 (32.7 to 178.1)	49.1 (13.4 to 91.5)	50.9 (-8 to 153.6)	43.3 (-4.3 to 114.5)

Location	Measure	Metric	2010			% Change					
						1990 to 2010			2010 to 2019		
			Both	Female	Male	Both	Female	Male	Both	Female	Male
	YLDs	Rate	10.7 (7.3 to 14.6)	21.5 (14.6 to 29.5)	0.2 (0.1 to 0.3)	8.7 (-22.1 to 56)	27 (-2.6 to 66.1)	7.9 (-23.3 to 55.3)	26.2 (-3.3 to 64.4)	-9.3 (-46.3 to 58)	15.6 (-21.7 to 69)
		Number	1218 (825 to 1674)	1208 (819 to 1664)	10 (6 to 15)	144.2 (70.8 to 254.2)	77.7 (33.9 to 135.6)	144.6 (70.4 to 256.9)	77.8 (33.7 to 136.6)	98.3 (16.6 to 245.9)	66.8 (11.2 to 144.4)
West Azarbayejan	Incidence	Rate*	10 (8.8 to 11.3)	19.6 (17.2 to 22.3)	0.2 (0.1 to 0.2)	24.2 (-9.4 to 74.8)	57.2 (24.7 to 96.4)	18.9 (-13.6 to 68.4)	58.1 (24.9 to 97.9)	8.7 (-31.2 to 73.6)	15.5 (-20.9 to 65.5)
		Number†	243 (214 to 278)	241 (212 to 276)	2 (1 to 2)	153.6 (85.5 to 260.4)	111.5 (67.3 to 169.4)	154.1 (85.3 to 261)	111.9 (67.1 to 170.2)	96.9 (20.4 to 220)	56.2 (7.3 to 123.4)
	Prevalence	Rate	91.7 (81.9 to 103)	179.4 (159.9 to 201.4)	1.6 (1.3 to 2)	22.1 (-2.9 to 52.9)	53.7 (27.5 to 86.5)	16.3 (-7.9 to 46.2)	54.7 (27.9 to 88.1)	5.1 (-23 to 44.2)	24.4 (-7.2 to 63.1)
		Number	2209 (1964 to 2496)	2193 (1948 to 2479)	17 (13 to 21)	153 (96.7 to 225.7)	108.9 (71.2 to 157.1)	153.5 (96.6 to 226.5)	109.2 (71.1 to 157.8)	101.3 (40.5 to 180.9)	63.6 (21.4 to 116.2)
	Deaths	Rate	5 (4.4 to 5.6)	9.6 (8.5 to 10.8)	0.1 (0.1 to 0.1)	-4.4 (-34.1 to 34.6)	28.1 (2.9 to 57.9)	-8.9 (-37.3 to 30.2)	28.5 (2.9 to 58.4)	-9.2 (-42.8 to 44.3)	-2.9 (-30.1 to 31.3)
		Number	106 (94 to 118)	105 (93 to 117)	1 (1 to 1)	92.1 (33.9 to 167.7)	74.2 (39.1 to 117.1)	92.4 (34.3 to 169.4)	74.6 (38.7 to 118)	65 (2.3 to 162)	33.9 (-4.4 to 84.6)
	DALYs	Rate	145.3 (129.6 to 162.8)	284.7 (253.4 to 319.1)	2.9 (2.3 to 3.5)	-5.3 (-33 to 29.5)	27.4 (2.7 to 58.9)	-9.6 (-36.3 to 24.7)	28.4 (3.1 to 60.8)	-12 (-44.5 to 36.8)	-1 (-27.8 to 35.4)
		Number	3637 (3228 to 4091)	3605 (3193 to 4061)	32 (26 to 38)	87.3 (31.6 to 154.4)	70.3 (36.9 to 114.8)	87.6 (31.4 to 155.6)	70.7 (37 to 115.7)	54.6 (-2 to 138.1)	30.7 (-4.9 to 79.4)
	YLLs	Rate	138.7 (123.2 to 155.7)	271.7 (241.1 to 305.4)	2.7 (2.2 to 3.3)	-6.4 (-34.2 to 29.6)	26.1 (0.4 to 57.5)	-10.6 (-37.8 to 24.4)	27.1 (0.8 to 59.4)	-12.9 (-45.6 to 37.1)	-2.1 (-29.2 to 35.3)
		Number	3473 (3075 to 3910)	3442 (3043 to 3881)	30 (25 to 36)	84.9 (30.4 to 153.3)	68.5 (33.9 to 115.4)	85.2 (30.3 to 153.6)	68.9 (33.9 to 116.1)	52.8 (-3.9 to 133.9)	29.3 (-6 to 78)

Location	Measure	Metric	2010			% Change					
						1990 to 2010			2010 to 2019		
			Both	Female	Male	Both	Female	Male	Both	Female	Male
Yazd	YLDs	Rate	6.6 (4.7 to 9.4)	13 (9.1 to 18.5)	0.1 (0.1 to 0.2)	25.8 (-6.7 to 71.9)	55.4 (22.8 to 93.9)	20.2 (-11.1 to 64.4)	56.3 (23.3 to 95.6)	11.1 (-27.3 to 69.5)	21.6 (-14.8 to 70)
		Number	164 (114 to 234)	162 (113 to 233)	1 (1 to 2)	158.4 (88.8 to 256.8)	108.4 (62.1 to 162.4)	159 (89 to 259.5)	108.8 (62 to 162.8)	105.9 (32.4 to 213.6)	61.2 (13.3 to 124.3)
	Incidence	Rate*	15.7 (13.3 to 18.4)	31.1 (26.2 to 36.6)	0.8 (0.6 to 1)	58.4 (4.3 to 141.7)	29.1 (-2.8 to 69.4)	61.3 (5.5 to 148.2)	31.3 (-1.7 to 73.6)	74.8 (9.4 to 181.8)	9.5 (-28.3 to 63.8)
		Number†	140 (117 to 165)	137 (114 to 162)	3 (2 to 4)	264.6 (143.5 to 456.1)	67.3 (25.8 to 119)	264.4 (140 to 461.8)	67.9 (24.9 to 121.3)	270.1 (132.3 to 515.5)	43.2 (-7 to 114.8)
	Prevalence	Rate	141.6 (122.1 to 163.2)	281.6 (241 to 326.6)	5.9 (4.6 to 7.6)	51.7 (12 to 103.4)	30 (2.2 to 64.8)	55.5 (13.6 to 111.8)	32.2 (2.9 to 68.1)	73.9 (16.1 to 153.5)	17.6 (-20.2 to 72.6)
		Number	1254 (1065 to 1458)	1230 (1041 to 1434)	24 (18 to 30)	253.4 (157.3 to 386.5)	68.6 (30.4 to 115.4)	253 (155.1 to 389)	69 (29.4 to 116.8)	277.7 (152.3 to 479.3)	49.9 (0.5 to 121.2)
	Deaths	Rate	6.5 (5.7 to 7.6)	12.5 (10.8 to 14.7)	0.5 (0.4 to 0.6)	11.7 (-27.8 to 72.8)	2 (-20.5 to 28.3)	13.8 (-26.4 to 77.2)	4.4 (-19.3 to 32.7)	38 (-10.7 to 129.7)	-11 (-39.1 to 25.2)
		Number	53 (45 to 62)	51 (44 to 60)	2 (1 to 2)	152 (64.2 to 284.1)	30.8 (1.6 to 65.7)	151 (62.1 to 287)	31.1 (0.1 to 67)	189.5 (80.3 to 387.7)	20.2 (-18.8 to 71)
	DALYs	Rate	198.2 (169.1 to 229.9)	393.4 (333.9 to 458)	11.2 (8.7 to 13.9)	11.8 (-24.5 to 66.7)	2.9 (-20.1 to 29.2)	12.8 (-24.1 to 69.2)	4.4 (-19.3 to 31.7)	31.8 (-15.9 to 119.1)	-6.9 (-36 to 32.7)
		Number	1787 (1519 to 2080)	1740 (1471 to 2034)	46 (36 to 57)	150.5 (70.2 to 271.2)	32.9 (2.6 to 68.8)	149.9 (68.2 to 275.7)	33.3 (1.6 to 69.9)	175.4 (72.1 to 356)	18.7 (-19.8 to 69.9)
	YLLs	Rate	187.8 (160.1 to 220.1)	372.8 (316.5 to 438.1)	10.7 (8.3 to 13.3)	10 (-26.1 to 66.3)	1.4 (-22.5 to 29.4)	10.9 (-26.2 to 69.8)	2.8 (-22.4 to 31.7)	30 (-18.6 to 118.5)	-8 (-37.1 to 32.2)
		Number	1693 (1437 to 1979)	1649 (1394 to 1937)	44 (34 to 55)	146.2 (65.3 to 271.4)	30.9 (-0.6 to 66.6)	145.6 (64 to 273.8)	31.3 (-0.7 to 68.3)	171.6 (68.9 to 353.6)	17.4 (-21.2 to 70.4)

Location	Measure	Metric	2010			% Change					
						1990 to 2010			2010 to 2019		
			Both	Female	Male	Both	Female	Male	Both	Female	Male
	YLDs	Rate	10.4 (7.1 to 14.6)	20.6 (14 to 29.1)	0.6 (0.4 to 0.8)	57.9 (6.2 to 130.7)	30.7 (-2.9 to 69)	60.8 (6.9 to 140.5)	32.8 (-1.9 to 73.6)	78.3 (10.5 to 178.7)	13.4 (-27.2 to 74.8)
		Number	94 (63 to 132)	91 (62 to 129)	2 (1 to 3)	265 (146.6 to 445.4)	68.9 (24.7 to 123.4)	264.6 (143.5 to 448.9)	69.4 (24.6 to 124.8)	283 (139.1 to 520.7)	45.8 (-7 to 124.1)
Zanjan	Incidence	Rate[*]	9.6 (8.5 to 10.8)	18.6 (16.5 to 21)	0.2 (0.2 to 0.3)	69.3 (14.4 to 154.3)	31.1 (8.2 to 59.1)	63.3 (10.1 to 148.1)	31.6 (8.4 to 60.2)	49.1 (-3.3 to 141.4)	3.2 (-28.9 to 46.8)
		Number[†]	79 (70 to 89)	78 (69 to 88)	1 (1 to 1)	209.7 (115.2 to 360)	75 (43.4 to 113.7)	210.5 (114.7 to 365)	75.5 (43.4 to 114.6)	148.9 (55 to 303.1)	31.9 (-10.1 to 87.5)
	Prevalence	Rate	84.8 (75.8 to 94.8)	164.2 (146.8 to 183.6)	1.9 (1.5 to 2.3)	44 (12.2 to 88.5)	36 (14.8 to 59.4)	38.9 (7.4 to 83.9)	36.6 (14.8 to 60.3)	29.3 (-4.2 to 85.5)	14.3 (-16 to 52)
		Number	693 (616 to 775)	686 (610 to 768)	7 (6 to 9)	170.9 (109.7 to 259.4)	81.6 (52.2 to 114.2)	171.5 (109.8 to 261.3)	82 (52.4 to 114.8)	129.4 (63.1 to 234.5)	44 (8.3 to 93.2)
	Deaths	Rate	4.7 (4.2 to 5.2)	9 (8 to 10)	0.1 (0.1 to 0.2)	28.4 (-16.4 to 97.7)	7.3 (-10.9 to 28.7)	24.8 (-19 to 93.8)	6.7 (-11.6 to 28.1)	21.9 (-17.3 to 99.2)	-12.6 (-35.7 to 19)
		Number	36 (32 to 40)	36 (32 to 40)	0 (0 to 1)	143.6 (62.4 to 266.6)	42 (18.3 to 70.2)	144 (62.3 to 269.6)	42.4 (18.4 to 71.1)	116.8 (42 to 243.4)	12.3 (-17 to 50.4)
	DALYs	Rate	135.3 (122 to 150.7)	261.6 (235.9 to 290.5)	3.4 (2.8 to 4.1)	28.9 (-13.1 to 87.6)	4.9 (-12.2 to 25.5)	23.2 (-17.7 to 81.4)	5.7 (-11.5 to 27.2)	19.5 (-18.8 to 90.3)	-13.7 (-36.3 to 14.8)
		Number	1122 (1014 to 1244)	1109 (1001 to 1229)	14 (11 to 16)	126.3 (53 to 224.9)	40 (17.5 to 68)	126.9 (52.6 to 227.7)	40.3 (17.6 to 68.7)	87.7 (27.3 to 192.4)	10.1 (-18.8 to 46.6)
	YLLs	Rate	129.1 (115.9 to 143.7)	249.6 (224 to 277.2)	3.3 (2.7 to 3.9)	27.8 (-14.3 to 87.6)	3.5 (-14.1 to 24.9)	22.1 (-18.9 to 80.6)	4.3 (-13.4 to 26)	18.4 (-20.1 to 91)	-14.8 (-37.4 to 14.4)
		Number	1071 (965 to 1185)	1058 (953 to 1171)	13 (11 to 16)	123.9 (49.5 to 224.2)	38.1 (15.1 to 67.3)	124.5 (49 to 227.4)	38.4 (15.1 to 68.2)	85.5 (25.1 to 190.9)	8.7 (-20 to 44.7)

Location	Measure	Metric	2010			% Change					
						1990 to 2010			2010 to 2019		
			Both	Female	Male	Both	Female	Male	Both	Female	Male
	YLDs	Rate	6.2 (4.3 to 8.7)	12 (8.3 to 16.8)	0.2 (0.1 to 0.3)	57.5 (13.9 to 123.3)	34.5 (7.8 to 65.3)	51.9 (8.9 to 116.9)	35.2 (7.8 to 66.6)	45.5 (-0.3 to 125.5)	9.5 (-22.6 to 53)
		Number	52 (36 to 72)	51 (35 to 71)	1 (0 to 1)	191.3 (112.6 to 314.7)	79 (43.2 to 121.1)	191.9 (111.8 to 319.4)	79.5 (43.5 to 122.1)	148.3 (63.9 to 279.6)	38.4 (-2.1 to 93.3)

Data in parentheses are 95% uncertainty intervals; DALYs= Disability-Adjusted Life Years; YLLs= Years of Life Lost; YLDs= Years Lived with Disability

* Age-standardized rate (per 100,000) † All ages
