# Supplementary material for: National and subnational burden of female and male breast cancer and risk factors in Iran from 1990 to 2019: results from the Global Burden of Disease study 2019
Source: Breast Cancer Res. 2023 Apr 26;25:47. doi: 10.1186/s13058-023-01633-4 (PMC10131337; doi:10.1186/s13058-023-01633-4)
Supplement: Supplementary file 13 — Additional file 13. Table 5 Pearson’s correlation coefficient (r) between measures of burden and socio-demographic index (SDI) [file 13058_2023_1633_MOESM13_ESM.pdf]

Pearson's correlation coefficient (*r*) between measures of burden and socio-demographic index (SDI)

| Measure | Both | Female | Male |
|---|---|---|---|
| **Incidence** | 0.8282 | 0.8111 | 0.2021 |
| **Prevalence** | 0.8290 | 0.8117 | 0.3251 |
| **Deaths** | 0.5751 | 0.5287 | -0.0469 |
| **DALYs** | 0.5956 | 0.5393 | -0.0556 |
| **YLLs** | 0.5627 | 0.5032 | -0.0708 |
| **YLDs** | 0.8339 | 0.8175 | 0.2807 |

DALYs= Disability-Adjusted Life Years; YLLs= Years of Life Lost; YLDs= Years Lived with Disability
