# Supplementary material for: National and subnational burden of female and male breast cancer and risk factors in Iran from 1990 to 2019: results from the Global Burden of Disease study 2019
Source: Breast Cancer Res. 2023 Apr 26;25:47. doi: 10.1186/s13058-023-01633-4 (PMC10131337; doi:10.1186/s13058-023-01633-4)
Supplement: Supplementary file 14 — Additional file 14. Table 6 Breast cancer deaths and disability-adjusted life years (DALYs) rate (per 100,000 population), numbers and percent changes attributable to risk factors in all 31 provinces of Iran between 1990 and 2019 for both sexes, females, and males (provinces have been sorted alphabetically). [file 13058_2023_1633_MOESM14_ESM.pdf]

Province	Measure	Metric	Risk factors	1990			2019			% Change (1990 to 2019)		
				Both	Female	Male	Both	Female	Male	Both	Female	Male
Alborz	Deaths	Rate	Behavioral	0.51 (0.26 to 0.83)	1.07 (0.54 to 1.73)	0.01 (0.01 to 0.02)	0.52 (0.33 to 0.74)	1.07 (0.67 to 1.53)	0.01 (0.01 to 0.02)	2.18 (-36.9 to 73.48)	0.74 (-38.68 to 72.93)	-0.97 (-46.23 to 93.24)
			Metabolic	0.49 (0.1 to 1.13)	1.02 (0.21 to 2.33)		0.93 (0.3 to 1.78)	1.94 (0.62 to 3.7)		90.18 (1.09 to 313.21)	90.7 (1.78 to 322.27)	
		Number	Behavioral	3 (2 to 5)	3 (2 to 5)	0 (0 to 0)	14 (9 to 20)	14 (9 to 20)	0 (0 to 0)	344.88 (179.27 to 678.6)	345.03 (178.69 to 685.73)	330.75 (134.44 to 782.03)
			Metabolic	2 (0 to 5)	2 (0 to 5)		20 (5 to 41)	20 (5 to 41)		895.21 (-893.07 to 3085.91)	895.21 (-893.07 to 3085.91)	
	DALYs	Rate	Behavioral	15.24 (7.55 to 24.29)	32.38 (15.94 to 51.98)	0.3 (0.14 to 0.55)	15.51 (9.72 to 21.92)	31.38 (19.68 to 44.5)	0.32 (0.18 to 0.5)	1.76 (-35.49 to 73.89)	-3.08 (-38.47 to 66.18)	4.75 (-44.1 to 111.59)
			Metabolic	8.78 (-1.42 to 23.28)	19.02 (-2.69 to 49.58)		18.95 (3.04 to 39.16)	38.46 (5.87 to 79.98)		115.81 (-283.94 to 653.07)	102.24 (-215.95 to 565.35)	
		Number	Behavioral	114 (52 to 184)	113 (51 to 182)	1 (1 to 2)	486 (303 to 693)	481 (299 to 687)	5 (3 to 8)	325.19 (175.01 to 631.68)	325.19 (172.38 to 639.96)	324.66 (128.93 to 753.72)
			Metabolic	30 (-43 to 119)	30 (-43 to 119)		409 (-63 to 986)	409 (-63 to 986)		1246.17 (-10110.62 to 7874.88)	1246.17 (-10110.62 to 7874.88)	
Ardebil	Deaths	Rate	Behavioral	0.39 (0.22 to 0.63)	0.83 (0.46 to 1.34)	0.01 (0 to 0.01)	0.42 (0.26 to 0.59)	0.82 (0.51 to 1.14)	0.01 (0 to 0.01)	7.26 (-31.65 to 66.83)	-1.37 (-37.53 to 54.14)	-1.01 (-42.36 to 86)
			Metabolic	0.34 (0.07 to 0.79)	0.77 (0.18 to 1.75)		0.68 (0.23 to 1.31)	1.31 (0.42 to 2.53)		100.41 (9.7 to 328.64)	70.55 (-8.22 to 233.91)	
		Number	Behavioral	2 (1 to 3)	2 (1 to 3)	0 (0 to 0)	5 (3 to 7)	5 (3 to 7)	0 (0 to 0)	143.32 (57.76 to 272.53)	143.63 (57.78 to 273.11)	105.3 (16.09 to 293.53)

Province	Measure	Metric	Risk factors	1990			2019			% Change (1990 to 2019)		
				Both	Female	Male	Both	Female	Male	Both	Female	Male
			Metabolic	2 (0 to 4)	2 (0 to 4)		7 (2 to 14)	7 (2 to 14)		339.07 (117.13 to 841.55)	339.07 (117.13 to 841.55)	
	DALYs	Rate	Behavioral	11.96 (6.65 to 18.56)	25.15 (14.07 to 39.06)	0.17 (0.08 to 0.3)	12.46 (7.66 to 17.59)	24.6 (15.05 to 34.92)	0.17 (0.09 to 0.26)	4.14 (-31.28 to 56.53)	-2.15 (-35.79 to 47.83)	-2.76 (-43.91 to 81.78)
			Metabolic	6.37 (-0.81 to 16.5)	15.2 (-0.23 to 37.25)		13.56 (1.49 to 29.75)	25.79 (2.07 to 57.67)		113 (-326.94 to 616.6)	69.59 (-68.84 to 423.67)	
		Number	Behavioral	72 (40 to 111)	72 (39 to 111)	1 (0 to 1)	167 (101 to 239)	166 (101 to 238)	1 (1 to 2)	131.26 (54.03 to 247.82)	131.55 (53.79 to 249.64)	92.68 (7 to 270.19)
			Metabolic	33 (-8 to 91)	33 (-8 to 91)		130 (-25 to 330)	130 (-25 to 330)		290 (-364.5 to 1089.83)	290 (-364.5 to 1089.83)	
Bushehr	Deaths	Rate	Behavioral	0.4 (0.21 to 0.7)	0.81 (0.42 to 1.42)	0.01 (0 to 0.01)	0.47 (0.3 to 0.67)	0.95 (0.6 to 1.35)	0.01 (0 to 0.01)	18.13 (-28.26 to 94.96)	18.08 (-28.62 to 96.62)	4.74 (-40.36 to 87.21)
			Metabolic	0.36 (0.07 to 0.88)	0.74 (0.15 to 1.79)		0.75 (0.22 to 1.49)	1.48 (0.41 to 2.95)		110.11 (7.23 to 375.98)	100.19 (-1.15 to 335.75)	
		Number	Behavioral	1 (1 to 2)	1 (1 to 2)	0 (0 to 0)	5 (3 to 7)	5 (3 to 6)	0 (0 to 0)	283.27 (140.75 to 521.25)	283.68 (140.71 to 523.68)	219.38 (75.6 to 488.38)
			Metabolic	1 (0 to 2)	1 (0 to 2)		5 (1 to 12)	5 (1 to 12)		561.4 (91.37 to 1851.51)	561.4 (91.37 to 1851.51)	
	DALYs	Rate	Behavioral	12.1 (6.33 to 20.16)	24.71 (12.93 to 41.45)	0.14 (0.06 to 0.25)	14.53 (8.98 to 20.81)	30 (18.46 to 43.07)	0.15 (0.08 to 0.23)	20.09 (-23.02 to 90.23)	21.41 (-22.49 to 92.22)	5.21 (-41.65 to 93.11)
			Metabolic	6.59 (-0.76 to 18.42)	14.04 (-1.11 to 38.47)	0 (0 to 0)	15.13 (0.99 to 35.09)	29.3 (0.24 to 70.08)	0 (0 to 0)	129.59 (-299.83 to 1017.46)	108.67 (-181.41 to 689.83)	0 (0 to 0)
		Number	Behavioral	42 (21 to 68)	42 (21 to 68)	0 (0 to 0)	162 (98 to 232)	161 (98 to 231)	1 (0 to 1)	287.06 (153.55 to 501.99)	287.46 (153.29 to 504.62)	217.41 (73.34 to 493.51)

Province	Measure	Metric	Risk factors	1990			2019			% Change (1990 to 2019)		
				Both	Female	Male	Both	Female	Male	Both	Female	Male
			Metabolic	13 (-11 to 49)	13 (-11 to 49)	0 (0 to 0)	93 (-60 to 288)	93 (-60 to 288)	0 (0 to 0)	587.27 (-2813.52 to 3508.57)	587.27 (-2813.52 to 3508.57)	0 (0 to 0)
Chahar Mahaal and Bakhtiari	Deaths	Rate	Behavioral	0.34 (0.18 to 0.56)	0.7 (0.37 to 1.16)	0.01 (0 to 0.01)	0.29 (0.18 to 0.43)	0.58 (0.35 to 0.85)	0 (0 to 0.01)	-13.28 (-44.29 to 36.28)	-18.18 (-47.86 to 29.3)	-15.23 (-53.45 to 52.1)
			Metabolic	0.26 (0.06 to 0.64)	0.55 (0.13 to 1.34)	0 (0 to 0)	0.38 (0.12 to 0.79)	0.74 (0.22 to 1.54)	0 (0 to 0)	47.01 (-21.05 to 192.87)	32.71 (-29.11 to 152.25)	0 (0 to 0)
		Number	Behavioral	1 (1 to 2)	1 (1 to 2)	0 (0 to 0)	3 (2 to 4)	3 (2 to 4)	0 (0 to 0)	145.5 (62.48 to 274.67)	145.7 (62.04 to 276.55)	119.11 (17.96 to 318.89)
			Metabolic	1 (0 to 2)	1 (0 to 2)	0 (0 to 0)	3 (1 to 6)	3 (1 to 6)	0 (0 to 0)	301.33 (79.5 to 708.98)	301.33 (79.5 to 708.98)	0 (0 to 0)
	DALYs	Rate	Behavioral	9.98 (5.44 to 15.64)	20.95 (11.39 to 32.8)	0.13 (0.06 to 0.22)	9 (5.41 to 13.05)	17.92 (10.73 to 26.07)	0.11 (0.06 to 0.18)	-9.87 (-39.32 to 34.16)	-14.43 (-42.97 to 27.91)	-12.93 (-52.49 to 66.32)
			Metabolic	4.97 (-0.13 to 13.42)	11.25 (0.24 to 29.54)	0 (0 to 0)	7.82 (0.64 to 18.12)	15.16 (0.95 to 35.66)	0 (0 to 0)	57.37 (-85.85 to 321.24)	34.76 (-54.82 to 222.75)	0 (0 to 0)
		Number	Behavioral	35 (19 to 55)	35 (19 to 55)	0 (0 to 0)	87 (51 to 127)	86 (51 to 127)	1 (0 to 1)	148.55 (65.97 to 264.59)	148.77 (65.51 to 266.84)	115.43 (18.26 to 315.02)
			Metabolic	14 (-3 to 40)	14 (-3 to 40)	0 (0 to 0)	54 (-12 to 146)	54 (-12 to 146)	0 (0 to 0)	278.65 (-232.08 to 1067.57)	278.65 (-232.08 to 1067.57)	0 (0 to 0)
East Azarbayejan	Deaths	Rate	Behavioral	0.36 (0.2 to 0.59)	0.74 (0.41 to 1.23)	0.01 (0 to 0.01)	0.48 (0.3 to 0.69)	0.95 (0.59 to 1.37)	0.01 (0 to 0.01)	33.48 (-16.84 to 108.23)	28.27 (-20.59 to 101.98)	27.24 (-28.46 to 128.02)
			Metabolic	0.26 (0.03 to 0.64)	0.56 (0.08 to 1.35)	0 (0 to 0)	0.69 (0.18 to 1.42)	1.36 (0.35 to 2.82)	0 (0 to 0)	163.61 (27.47 to 559.65)	141.53 (17.2 to 483.49)	0 (0 to 0)
		Number	Behavioral	7 (4 to 11)	6 (3 to 11)	0 (0 to 0)	20 (12 to 29)	20 (12 to 29)	0 (0 to 0)	206.35 (95.41 to 376.99)	206.57 (94.83 to 379.61)	182.59 (53.66 to 422.67)

Province	Measure	Metric	Risk factors	1990			2019			% Change (1990 to 2019)		
				Both	Female	Male	Both	Female	Male	Both	Female	Male
			Metabolic	4 (0 to 9)	4 (0 to 9)	0 (0 to 0)	23 (4 to 51)	23 (4 to 51)	0 (0 to 0)	519.4 (0.84 to 1789.9)	519.4 (0.84 to 1789.9)	0 (0 to 0)
	DALYs	Rate	Behavioral	11.34 (6.02 to 18.06)	23.35 (12.44 to 37.43)	0.18 (0.08 to 0.32)	15.05 (9.21 to 21.89)	29.99 (18.34 to 43.59)	0.24 (0.13 to 0.38)	32.77 (-14.31 to 105.47)	28.44 (-17.31 to 98.62)	31.1 (-25.71 to 141.99)
			Metabolic	4.08 (-2.31 to 12.8)	9.36 (-3.95 to 27.66)	0 (0 to 0)	12.36 (-1.4 to 31.1)	24.11 (-3.52 to 61.49)	0 (0 to 0)	203.27 (-1554.66 to 1784.33)	157.72 (-729.27 to 1492.7)	0 (0 to 0)
		Number	Behavioral	234 (122 to 370)	232 (121 to 367)	2 (1 to 3)	694 (419 to 1011)	689 (416 to 1004)	5 (3 to 8)	196.33 (93.83 to 360.36)	196.49 (93.37 to 361.2)	176.37 (51.79 to 423.05)
			Metabolic	64 (-66 to 220)	64 (-66 to 220)	0 (0 to 0)	400 (-213 to 1187)	400 (-213 to 1187)	0 (0 to 0)	529.2 (-3408.68 to 2821.07)	529.2 (-3408.68 to 2821.07)	0 (0 to 0)
Fars	Deaths	Rate	Behavioral	0.3 (0.15 to 0.52)	0.61 (0.3 to 1.05)	0.01 (0 to 0.01)	0.42 (0.24 to 0.6)	0.83 (0.49 to 1.21)	0.01 (0 to 0.01)	36.41 (-17.5 to 127.7)	36.1 (-18.1 to 128.32)	20.45 (-33.12 to 136.25)
			Metabolic	0.25 (0.04 to 0.6)	0.5 (0.08 to 1.21)	0 (0 to 0)	0.61 (0.15 to 1.25)	1.22 (0.3 to 2.5)	0 (0 to 0)	147.52 (12.54 to 474.35)	141.45 (6.79 to 426.32)	0 (0 to 0)
		Number	Behavioral	5 (3 to 9)	5 (3 to 9)	0 (0 to 0)	21 (12 to 30)	20 (12 to 30)	0 (0 to 0)	293.15 (141.39 to 562.77)	293.73 (140.44 to 566.54)	235.67 (80.12 to 580.93)
			Metabolic	3 (0 to 8)	3 (0 to 8)	0 (0 to 0)	23 (0 to 51)	23 (0 to 51)	0 (0 to 0)	587.32 (-138.4 to 1656.96)	587.32 (-138.4 to 1656.96)	0 (0 to 0)
	DALYs	Rate	Behavioral	9.49 (4.7 to 15.63)	19.35 (9.53 to 31.94)	0.17 (0.07 to 0.3)	13.39 (7.75 to 19.89)	26.79 (15.41 to 39.89)	0.21 (0.12 to 0.34)	41.02 (-12 to 131.45)	38.41 (-13.72 to 127.72)	26.81 (-30.31 to 159.92)
			Metabolic	4.03 (-1.78 to 12.5)	8.86 (-3.05 to 26.39)	0 (0 to 0)	10.78 (-2.81 to 27.53)	21.25 (-5.99 to 54.89)	0 (0 to 0)	167.3 (-504.1 to 1385.29)	139.85 (-433.52 to 985.54)	0 (0 to 0)
		Number	Behavioral	186 (91 to 308)	185 (89 to 307)	2 (1 to 3)	737 (422 to 1108)	732 (418 to 1102)	6 (3 to 9)	295.95 (148.75 to 554.29)	296.51 (148.16 to 557.89)	233.56 (79.35 to 588.72)

Province	Measure	Metric	Risk factors	1990			2019			% Change (1990 to 2019)		
				Both	Female	Male	Both	Female	Male	Both	Female	Male
			**Metabolic**	51 (-59 to 200)	51 (-59 to 200)	0 (0 to 0)	332 (-388 to 1179)	332 (-388 to 1179)	0 (0 to 0)	556.12 (-2424.64 to 3188.03)	556.12 (-2424.64 to 3188.03)	0 (0 to 0)
**Gilan**	**Deaths**	**Rate**	**Behavioral**	0.38 (0.2 to 0.62)	0.73 (0.39 to 1.19)	0.01 (0 to 0.02)	0.49 (0.31 to 0.7)	0.97 (0.62 to 1.37)	0.01 (0 to 0.01)	29.16 (-17.44 to 108.18)	31.65 (-15.91 to 114.3)	0.18 (-42.96 to 89.04)
			**Metabolic**	0.28 (0.04 to 0.67)	0.54 (0.08 to 1.27)	0 (0 to 0)	0.74 (0.21 to 1.49)	1.46 (0.42 to 2.95)	0 (0 to 0)	161.45 (25.8 to 491.53)	171.37 (29.28 to 533.41)	0 (0 to 0)
		**Number**	**Behavioral**	5 (3 to 8)	5 (3 to 8)	0 (0 to 0)	16 (10 to 23)	16 (10 to 23)	0 (0 to 0)	214.03 (100.63 to 410.98)	214.71 (100.38 to 413.9)	150.93 (40.79 to 369.29)
			**Metabolic**	3 (0 to 8)	3 (0 to 8)	0 (0 to 0)	23 (6 to 46)	23 (6 to 46)	0 (0 to 0)	617.15 (180.91 to 1957.51)	617.15 (180.91 to 1957.51)	0 (0 to 0)
	**DALYs**	**Rate**	**Behavioral**	11.91 (6.4 to 18.81)	23.57 (12.55 to 37.26)	0.24 (0.11 to 0.4)	15.65 (9.84 to 22.25)	30.8 (19.36 to 43.77)	0.25 (0.14 to 0.39)	31.4 (-14.09 to 107.87)	30.67 (-14.56 to 107.23)	4.54 (-39.4 to 96.89)
			**Metabolic**	4.59 (-2.15 to 13.74)	9.19 (-4.29 to 27.2)	0 (0 to 0)	14.72 (-0.05 to 34.01)	28.73 (-0.59 to 66.79)	0 (0 to 0)	220.4 (-1087.16 to 1571.78)	212.74 (-1056.5 to 1527.83)	0 (0 to 0)
		**Number**	**Behavioral**	178 (95 to 279)	176 (94 to 277)	2 (1 to 3)	534 (333 to 756)	529 (330 to 750)	4 (2 to 6)	199.66 (96.34 to 372.16)	200.3 (96.65 to 376.41)	134.63 (32.03 to 351.23)
			**Metabolic**	56 (-42 to 183)	56 (-42 to 183)	0 (0 to 0)	478 (-21 to 1130)	478 (-21 to 1130)	0 (0 to 0)	750.56 (-2897.12 to 4872.05)	750.56 (-2897.12 to 4872.05)	0 (0 to 0)
**Golestan**	**Deaths**	**Rate**	**Behavioral**	0.34 (0.18 to 0.55)	0.69 (0.37 to 1.11)	0.01 (0 to 0.01)	0.47 (0.29 to 0.67)	0.91 (0.56 to 1.3)	0.01 (0 to 0.01)	37.94 (-18.22 to 123.92)	32.57 (-21.89 to 116.95)	12.93 (-34.15 to 112.71)
			**Metabolic**	0.28 (0.03 to 0.68)	0.58 (0.08 to 1.41)	0 (0 to 0)	0.66 (0.17 to 1.36)	1.28 (0.32 to 2.66)	0 (0 to 0)	140.14 (11.17 to 491.31)	120.71 (-0.59 to 385.41)	0 (0 to 0)
		**Number**	**Behavioral**	2 (1 to 3)	2 (1 to 3)	0 (0 to 0)	8 (5 to 12)	8 (5 to 12)	0 (0 to 0)	296.37 (136.67 to 533.05)	297.13 (136.16 to 537.06)	205.05 (71.35 to 490.76)

Province	Measure	Metric	Risk factors	1990			2019			% Change (1990 to 2019)		
				Both	Female	Male	Both	Female	Male	Both	Female	Male
			**Metabolic**	1 (0 to 3)	1 (0 to 3)	0 (0 to 0)	9 (1 to 20)	9 (1 to 20)	0 (0 to 0)	620.35 (-759.09 to 2518.82)	620.35 (-759.09 to 2518.82)	0 (0 to 0)
	**DALYs**	**Rate**	**Behavioral**	10.77 (5.7 to 16.88)	22.01 (11.67 to 34.65)	0.16 (0.07 to 0.28)	15.42 (9.36 to 22.3)	30.17 (18.3 to 43.63)	0.19 (0.1 to 0.3)	43.09 (-12.96 to 127.23)	37.11 (-16.58 to 119.21)	18.33 (-33.15 to 124.92)
			**Metabolic**	4.59 (-2.31 to 14.63)	10.51 (-3.59 to 31.56)	0 (0 to 0)	13.84 (-1.15 to 34.5)	26.58 (-2.92 to 67.04)	0 (0 to 0)	201.46 (-1163.98 to 1228.52)	152.82 (-579.17 to 1099.36)	0 (0 to 0)
		**Number**	**Behavioral**	75 (39 to 118)	75 (39 to 117)	1 (0 to 1)	301 (181 to 439)	299 (180 to 437)	2 (1 to 3)	300.66 (144.46 to 526.45)	301.35 (144.52 to 529.03)	204.53 (66.99 to 471.85)
			**Metabolic**	18 (-28 to 82)	18 (-28 to 82)	0 (0 to 0)	173 (-115 to 549)	173 (-115 to 549)	0 (0 to 0)	850.32 (-4395.46 to 3962.26)	850.32 (-4395.46 to 3962.26)	0 (0 to 0)
**Hamadan**	**Deaths**	**Rate**	**Behavioral**	0.37 (0.2 to 0.6)	0.75 (0.42 to 1.25)	0.01 (0 to 0.01)	0.41 (0.26 to 0.59)	0.82 (0.51 to 1.18)	0 (0 to 0.01)	12.47 (-29.5 to 74.22)	8.59 (-32.35 to 69.21)	-4.8 (-45.82 to 72.33)
			**Metabolic**	0.27 (0.06 to 0.63)	0.58 (0.13 to 1.32)	0 (0 to 0)	0.58 (0.17 to 1.16)	1.14 (0.34 to 2.3)	0 (0 to 0)	111.05 (9.53 to 327.54)	95.64 (2.48 to 297.81)	0 (0 to 0)
		**Number**	**Behavioral**	3 (2 to 5)	3 (2 to 5)	0 (0 to 0)	8 (5 to 11)	8 (5 to 11)	0 (0 to 0)	138.21 (50.6 to 263.69)	138.48 (49.92 to 265.24)	99.66 (10.84 to 256.6)
			**Metabolic**	2 (0 to 5)	2 (0 to 5)	0 (0 to 0)	9 (2 to 20)	9 (2 to 20)	0 (0 to 0)	356.76 (106.06 to 893)	356.76 (106.06 to 893)	0 (0 to 0)
	**DALYs**	**Rate**	**Behavioral**	11.45 (6.23 to 18.27)	23.5 (12.79 to 37.65)	0.14 (0.06 to 0.25)	12.85 (7.82 to 18.82)	25.54 (15.54 to 37.08)	0.14 (0.07 to 0.23)	12.19 (-26.16 to 67.14)	8.71 (-28.94 to 62.37)	-2.3 (-46.92 to 80.11)
			**Metabolic**	5.06 (-1.04 to 13.6)	11.25 (-1.36 to 29.54)	0 (0 to 0)	11.38 (0.45 to 26.77)	22.23 (0.46 to 52.83)	0 (0 to 0)	124.98 (-353.37 to 734.9)	97.62 (-90.46 to 563.7)	0 (0 to 0)
		**Number**	**Behavioral**	110 (60 to 175)	109 (60 to 175)	1 (0 to 1)	252 (151 to 369)	250 (151 to 364)	1 (1 to 2)	128.97 (50.96 to 241.65)	129.22 (50.62 to 243.78)	88.74 (1.39 to 249.8)

Province	Measure	Metric	Risk factors	1990			2019			% Change (1990 to 2019)		
				Both	Female	Male	Both	Female	Male	Both	Female	Male
			Metabolic	40 (-16 to 115)	40 (-16 to 115)	0 (0 to 0)	172 (-37 to 457)	172 (-37 to 457)	0 (0 to 0)	334.31 (-891.56 to 1632.94)	334.31 (-891.56 to 1632.94)	0 (0 to 0)
Hormozgan	Deaths	Rate	Behavioral	0.31 (0.17 to 0.5)	0.65 (0.35 to 1.04)	0.01 (0.01 to 0.02)	0.39 (0.24 to 0.55)	0.77 (0.48 to 1.1)	0.01 (0.01 to 0.02)	23.18 (-22.15 to 102.6)	17.67 (-26.31 to 94.5)	-22.02 (-56.7 to 53.74)
			Metabolic	0.24 (0.04 to 0.56)	0.53 (0.1 to 1.19)	0 (0 to 0)	0.58 (0.18 to 1.15)	1.14 (0.34 to 2.27)	0 (0 to 0)	138.37 (23.26 to 398.13)	117.16 (11.63 to 332.84)	0 (0 to 0)
		Number	Behavioral	1 (1 to 2)	1 (1 to 2)	0 (0 to 0)	5 (3 to 7)	5 (3 to 7)	0 (0 to 0)	295.08 (151.5 to 558.19)	298.75 (151.26 to 564.58)	125.85 (24.72 to 364.79)
			Metabolic	1 (0 to 2)	1 (0 to 2)	0 (0 to 0)	5 (1 to 12)	5 (1 to 12)	0 (0 to 0)	537.89 (86.97 to 1289.62)	537.89 (86.97 to 1289.62)	0 (0 to 0)
	DALYs	Rate	Behavioral	9.49 (5.05 to 14.83)	20.03 (10.61 to 31.49)	0.35 (0.15 to 0.62)	11.76 (7.26 to 16.93)	23.44 (14.43 to 33.72)	0.28 (0.15 to 0.44)	23.94 (-19.82 to 114.57)	16.98 (-25.39 to 101.3)	-18.27 (-54.97 to 69.16)
			Metabolic	4.22 (-1 to 11.18)	9.81 (-1.52 to 25.01)	0 (0 to 0)	11.47 (0.88 to 26.41)	21.87 (0.39 to 51.23)	0 (0 to 0)	171.78 (-357.65 to 980.22)	123.05 (-284.08 to 546.66)	0 (0 to 0)
		Number	Behavioral	43 (23 to 68)	43 (22 to 68)	1 (0 to 2)	179 (110 to 261)	177 (108 to 259)	2 (1 to 3)	311.82 (166.13 to 663)	315.22 (166.09 to 679.3)	141.6 (30.05 to 409.2)
			Metabolic	13 (-10 to 42)	13 (-10 to 42)	0 (0 to 0)	79 (-79 to 267)	79 (-79 to 267)	0 (0 to 0)	498.25 (-1636.81 to 3915.13)	498.25 (-1636.81 to 3915.13)	0 (0 to 0)
Ilam	Deaths	Rate	Behavioral	0.28 (0.14 to 0.5)	0.63 (0.31 to 1.14)	0.01 (0 to 0.01)	0.44 (0.28 to 0.62)	0.88 (0.55 to 1.24)	0.01 (0 to 0.01)	58.57 (-10.54 to 172.46)	39.94 (-22.34 to 142.44)	26.38 (-29.3 to 149.55)
			Metabolic	0.21 (0.04 to 0.55)	0.52 (0.1 to 1.31)	0 (0 to 0)	0.69 (0.22 to 1.36)	1.38 (0.45 to 2.76)	0 (0 to 0)	222.18 (44.64 to 703.43)	166.17 (19.88 to 499.15)	0 (0 to 0)
		Number	Behavioral	0 (0 to 1)	0 (0 to 1)	0 (0 to 0)	2 (1 to 3)	2 (1 to 3)	0 (0 to 0)	370.92 (176.48 to 700.55)	372.73 (176.01 to 705.12)	232.43 (85.51 to 570.1)

Province	Measure	Metric	Risk factors	1990			2019			% Change (1990 to 2019)		
				Both	Female	Male	Both	Female	Male	Both	Female	Male
			Metabolic	0 (0 to 1)	0 (0 to 1)	0 (0 to 0)	3 (0 to 6)	3 (0 to 6)	0 (0 to 0)	821.4 (189.59 to 2738.96)	821.4 (189.59 to 2738.96)	0 (0 to 0)
	DALYs	Rate	Behavioral	8.36 (4.34 to 14.33)	18.71 (9.47 to 32.47)	0.18 (0.08 to 0.31)	13.47 (8.21 to 19.15)	26.67 (16.27 to 37.94)	0.23 (0.13 to 0.36)	61.1 (-3.41 to 166.88)	42.54 (-15.43 to 140.28)	32.66 (-27.7 to 171.71)
			Metabolic	3.93 (-0.58 to 11.19)	10.34 (0 to 28.32)	0 (0 to 0)	14.4 (1.67 to 31.44)	28.31 (2.78 to 62.28)	0 (0 to 0)	266.03 (-559.89 to 1728.63)	173.91 (-42.83 to 866.34)	0 (0 to 0)
		Number	Behavioral	17 (8 to 28)	16 (8 to 28)	0 (0 to 0)	79 (47 to 112)	78 (47 to 111)	1 (0 to 1)	376.77 (188.43 to 683.77)	378.27 (188.7 to 688.59)	246.24 (87.89 to 614.65)
			Metabolic	5 (-3 to 17)	5 (-3 to 17)	0 (0 to 0)	50 (-23 to 138)	50 (-23 to 138)	0 (0 to 0)	902.91 (-3030.49 to 5098.72)	902.91 (-3030.49 to 5098.72)	0 (0 to 0)
Isfahan	Deaths	Rate	Behavioral	0.36 (0.19 to 0.6)	0.71 (0.37 to 1.2)	0.01 (0 to 0.01)	0.45 (0.29 to 0.64)	0.92 (0.59 to 1.3)	0.01 (0 to 0.01)	26.44 (-23.17 to 118.6)	28.03 (-22.81 to 123.9)	23.49 (-28.83 to 135.2)
			Metabolic	0.32 (0.07 to 0.77)	0.63 (0.14 to 1.5)	0 (0 to 0)	0.7 (0.22 to 1.4)	1.44 (0.45 to 2.84)	0 (0 to 0)	118.65 (11.31 to 348.44)	127.1 (15.9 to 374.26)	0 (0 to 0)
		Number	Behavioral	7 (4 to 12)	7 (4 to 11)	0 (0 to 0)	25 (16 to 36)	25 (16 to 36)	0 (0 to 0)	263.48 (119.83 to 535.02)	263.48 (118.75 to 540.62)	263.31 (102.01 to 616.96)
			Metabolic	5 (1 to 12)	5 (1 to 12)	0 (0 to 0)	33 (8 to 69)	33 (8 to 69)	0 (0 to 0)	549.69 (206.88 to 1373.74)	549.69 (206.88 to 1373.74)	0 (0 to 0)
	DALYs	Rate	Behavioral	10.8 (5.61 to 17.48)	22.06 (11.48 to 35.79)	0.17 (0.08 to 0.3)	14.01 (8.88 to 19.98)	28.23 (17.89 to 40.35)	0.22 (0.12 to 0.34)	29.81 (-17.85 to 124.56)	27.98 (-19.39 to 122.01)	30.16 (-24.92 to 152.72)
			Metabolic	5.98 (-0.11 to 15.45)	12.1 (-0.35 to 31.53)	0 (0 to 0)	13.66 (0.65 to 31.48)	27.6 (1.21 to 63.67)	0 (0 to 0)	128.43 (-103.12 to 724.99)	128.13 (-118.03 to 777.73)	0 (0 to 0)
		Number	Behavioral	237 (121 to 381)	235 (120 to 379)	2 (1 to 3)	854 (535 to 1227)	847 (531 to 1219)	6 (4 to 10)	260.53 (127.4 to 544.99)	260.61 (125.91 to 547.75)	250.72 (96.83 to 606.76)

Province	Measure	Metric	Risk factors	1990			2019			% Change (1990 to 2019)		
				Both	Female	Male	Both	Female	Male	Both	Female	Male
			Metabolic	98 (-36 to 289)	98 (-36 to 289)	0 (0 to 0)	622 (-132 to 1640)	622 (-132 to 1640)	0 (0 to 0)	535 (-1106.66 to 2815.3)	535 (-1106.66 to 2815.3)	0 (0 to 0)
Kerman	Deaths	Rate	Behavioral	0.35 (0.19 to 0.58)	0.72 (0.38 to 1.18)	0.01 (0 to 0.01)	0.39 (0.25 to 0.54)	0.77 (0.49 to 1.09)	0.01 (0 to 0.01)	9.87 (-30.63 to 71.31)	8.21 (-32.28 to 70.19)	-1.46 (-42.61 to 79.43)
			Metabolic	0.28 (0.05 to 0.68)	0.58 (0.11 to 1.39)	0 (0 to 0)	0.56 (0.17 to 1.14)	1.12 (0.32 to 2.27)	0 (0 to 0)	101.28 (2.3 to 314.94)	92.3 (-1.49 to 291.44)	0 (0 to 0)
		Number	Behavioral	3 (2 to 5)	3 (2 to 5)	0 (0 to 0)	11 (7 to 15)	11 (7 to 15)	0 (0 to 0)	237.45 (116.91 to 428.13)	237.95 (116.05 to 431.57)	187.09 (63.81 to 444.3)
			Metabolic	2 (0 to 5)	2 (0 to 5)	0 (0 to 0)	11 (1 to 25)	11 (1 to 25)	0 (0 to 0)	450.45 (102.93 to 1077.31)	450.45 (102.93 to 1077.31)	0 (0 to 0)
	DALYs	Rate	Behavioral	10.86 (5.98 to 17.37)	22.32 (12.22 to 35.71)	0.19 (0.09 to 0.32)	11.97 (7.5 to 16.97)	24.11 (15.04 to 34.23)	0.19 (0.11 to 0.3)	10.21 (-27.45 to 67.29)	8.01 (-29.47 to 64.88)	1.03 (-43.03 to 87.29)
			Metabolic	4.88 (-1.67 to 14.17)	10.7 (-2.76 to 29.97)	0 (0 to 0)	10.77 (0.05 to 25.76)	20.96 (-0.87 to 51.05)	0 (0 to 0)	120.78 (-557.91 to 615.85)	95.79 (-226.37 to 489.39)	0 (0 to 0)
		Number	Behavioral	108 (59 to 173)	107 (57 to 172)	1 (0 to 2)	372 (228 to 527)	369 (226 to 524)	3 (2 to 4)	244.57 (127.38 to 422.65)	245.03 (126.14 to 426.12)	192.23 (61.68 to 454.55)
			Metabolic	37 (-26 to 120)	37 (-26 to 120)	0 (0 to 0)	187 (-142 to 598)	187 (-142 to 598)	0 (0 to 0)	406.64 (-1029.47 to 2553.99)	406.64 (-1029.47 to 2553.99)	0 (0 to 0)
Kermanshah	Deaths	Rate	Behavioral	0.35 (0.19 to 0.56)	0.75 (0.41 to 1.21)	0.01 (0 to 0.01)	0.45 (0.28 to 0.63)	0.88 (0.55 to 1.24)	0.01 (0 to 0.01)	28.02 (-18.84 to 104.5)	17.48 (-26.37 to 89.08)	-1.59 (-44.43 to 89.79)
			Metabolic	0.25 (0.04 to 0.62)	0.59 (0.12 to 1.42)	0 (0 to 0)	0.64 (0.18 to 1.28)	1.26 (0.34 to 2.52)	0 (0 to 0)	153.47 (24.36 to 464.26)	114.83 (7.25 to 331.14)	0 (0 to 0)
		Number	Behavioral	3 (2 to 4)	3 (2 to 4)	0 (0 to 0)	9 (6 to 13)	9 (5 to 13)	0 (0 to 0)	219.49 (105.34 to 407.48)	220.45 (104.54 to 411.04)	130.64 (26.03 to 351.02)

Province	Measure	Metric	Risk factors	1990			2019			% Change (1990 to 2019)		
				Both	Female	Male	Both	Female	Male	Both	Female	Male
			Metabolic	2 (0 to 4)	2 (0 to 4)	0 (0 to 0)	11 (2 to 22)	11 (2 to 22)	0 (0 to 0)	592.76 (-533.27 to 1915.37)	592.76 (-533.27 to 1915.37)	0 (0 to 0)
	DALYs	Rate	Behavioral	10.85 (5.92 to 17)	23.06 (12.64 to 36.54)	0.2 (0.09 to 0.36)	13.78 (8.36 to 19.87)	27.01 (16.32 to 38.99)	0.2 (0.11 to 0.32)	26.97 (-16.42 to 99.73)	17.12 (-23.59 to 86.41)	3.43 (-42.64 to 103.42)
			Metabolic	4.08 (-1.98 to 12.16)	10.5 (-2.46 to 28.95)	0 (0 to 0)	11.98 (-0.88 to 28.51)	23.05 (-2.22 to 55.42)	0 (0 to 0)	193.52 (-496.58 to 1340.18)	119.51 (-231.94 to 634.35)	0 (0 to 0)
		Number	Behavioral	98 (54 to 152)	97 (53 to 152)	1 (0 to 2)	302 (183 to 435)	299 (181 to 433)	2 (1 to 3)	206.46 (101.9 to 386.16)	207.2 (101.93 to 388.6)	128.56 (23.7 to 353.95)
			Metabolic	22 (-29 to 86)	22 (-29 to 86)	0 (0 to 0)	186 (-88 to 521)	186 (-88 to 521)	0 (0 to 0)	736.68 (-3863.2 to 6891.85)	736.68 (-3863.2 to 6891.85)	0 (0 to 0)
Khorasan-e-Razavi	Deaths	Rate	Behavioral	0.35 (0.2 to 0.56)	0.72 (0.4 to 1.15)	0.01 (0 to 0.01)	0.43 (0.26 to 0.6)	0.84 (0.51 to 1.19)	0.01 (0 to 0.01)	22.14 (-21.08 to 91.8)	16.8 (-24.93 to 84.76)	-2.45 (-42.45 to 72.1)
			Metabolic	0.34 (0.07 to 0.77)	0.72 (0.16 to 1.62)	0 (0 to 0)	0.73 (0.22 to 1.48)	1.43 (0.42 to 2.9)	0 (0 to 0)	114.52 (21.16 to 307.06)	97.91 (10.86 to 262.44)	0 (0 to 0)
		Number	Behavioral	8 (5 to 13)	8 (5 to 13)	0 (0 to 0)	25 (15 to 36)	25 (15 to 36)	0 (0 to 0)	202.74 (98.92 to 372.89)	203.3 (98.76 to 376.12)	129.21 (30.56 to 311.14)
			Metabolic	7 (1 to 16)	7 (1 to 16)	0 (0 to 0)	35 (7 to 76)	35 (7 to 76)	0 (0 to 0)	396.1 (136.02 to 827.75)	396.1 (136.02 to 827.75)	0 (0 to 0)
	DALYs	Rate	Behavioral	10.94 (6.07 to 17.01)	22.54 (12.47 to 35.07)	0.15 (0.07 to 0.25)	13.38 (7.88 to 19.08)	26.45 (15.58 to 37.84)	0.15 (0.08 to 0.23)	22.26 (-19.38 to 91.01)	17.31 (-23.02 to 83.81)	-0.63 (-43.55 to 78.06)
			Metabolic	6.9 (-0.35 to 17.38)	15.15 (0.13 to 37.45)	0 (0 to 0)	15.53 (1.38 to 35.26)	30.13 (2.16 to 68.84)	0 (0 to 0)	124.99 (-60.69 to 487.71)	98.79 (-72.97 to 346.01)	0 (0 to 0)
		Number	Behavioral	291 (155 to 455)	289 (154 to 452)	2 (1 to 3)	879 (508 to 1269)	874 (505 to 1265)	4 (2 to 7)	202.18 (96.77 to 367.6)	202.71 (96.9 to 369.37)	122.87 (26.16 to 305.22)

Province	Measure	Metric	Risk factors	1990			2019			% Change (1990 to 2019)		
				Both	Female	Male	Both	Female	Male	Both	Female	Male
			Metabolic	158 (-29 to 419)	158 (-29 to 419)	0 (0 to 0)	711 (-202 to 1903)	711 (-202 to 1903)	0 (0 to 0)	350.2 (-216.49 to 1196.26)	350.2 (-216.49 to 1196.26)	0 (0 to 0)
Khuzestan	Deaths	Rate	Behavioral	0.35 (0.2 to 0.56)	0.71 (0.4 to 1.13)	0.01 (0 to 0.01)	0.48 (0.29 to 0.67)	0.95 (0.58 to 1.33)	0.01 (0 to 0.01)	35.91 (-15.91 to 109.96)	34.52 (-17.14 to 109.33)	-14.24 (-51.71 to 56.71)
			Metabolic	0.3 (0.05 to 0.72)	0.61 (0.1 to 1.44)	0 (0 to 0)	0.81 (0.23 to 1.69)	1.61 (0.45 to 3.35)	0 (0 to 0)	170.69 (41.27 to 506.69)	163.79 (36.2 to 474.89)	0 (0 to 0)
		Number	Behavioral	5 (3 to 8)	5 (3 to 8)	0 (0 to 0)	19 (12 to 28)	19 (12 to 27)	0 (0 to 0)	300.57 (152.28 to 513.18)	302.33 (152.32 to 519.27)	142.36 (34.48 to 357.52)
			Metabolic	3 (0 to 8)	3 (0 to 8)	0 (0 to 0)	25 (3 to 55)	25 (3 to 55)	0 (0 to 0)	706.3 (-158.53 to 2135.69)	706.3 (-158.53 to 2135.69)	0 (0 to 0)
	DALYs	Rate	Behavioral	11.08 (6.29 to 17.65)	22.66 (12.86 to 36.18)	0.22 (0.1 to 0.39)	15.25 (9.18 to 21.5)	30.53 (18.36 to 43.11)	0.19 (0.11 to 0.32)	37.63 (-11.43 to 107.21)	34.7 (-13.91 to 103.67)	-12.18 (-51.01 to 62.63)
			Metabolic	5.25 (-1.72 to 15.13)	11.33 (-2.91 to 31.73)	0 (0 to 0)	16.93 (0.41 to 40.52)	33.46 (0.4 to 80.55)	0 (0 to 0)	222.59 (-519.62 to 1596.24)	195.41 (-549.92 to 1005.36)	0 (0 to 0)
		Number	Behavioral	172 (97 to 275)	170 (96 to 273)	2 (1 to 3)	699 (417 to 1004)	695 (414 to 999)	4 (2 to 6)	305.97 (159.09 to 504.49)	307.59 (159.1 to 508.87)	142.34 (34.32 to 375.66)
			Metabolic	45 (-64 to 181)	45 (-64 to 181)	0 (0 to 0)	468 (-245 to 1396)	468 (-245 to 1396)	0 (0 to 0)	929.72 (-3023.7 to 6453.33)	929.72 (-3023.7 to 6453.33)	0 (0 to 0)
Kohgiluyeh and Boyer-Ahmad	Deaths	Rate	Behavioral	0.23 (0.13 to 0.43)	0.48 (0.25 to 0.86)	0.01 (0.01 to 0.02)	0.31 (0.19 to 0.46)	0.65 (0.41 to 0.95)	0.01 (0 to 0.01)	32.6 (-26.71 to 133.28)	35.41 (-25.61 to 139.22)	-21.02 (-57.35 to 59.57)
			Metabolic	0.2 (0.04 to 0.48)	0.4 (0.09 to 0.96)	0 (0 to 0)	0.44 (0.13 to 0.87)	0.95 (0.29 to 1.89)	0 (0 to 0)	123.96 (9.09 to 400.44)	133.88 (16.41 to 406.04)	0 (0 to 0)
		Number	Behavioral	0 (0 to 1)	0 (0 to 1)	0 (0 to 0)	2 (1 to 3)	2 (1 to 3)	0 (0 to 0)	332.12 (150.03 to 642.26)	337.73 (149.91 to 663.08)	127.43 (19.47 to 384.53)

Province	Measure	Metric	Risk factors	1990			2019			% Change (1990 to 2019)		
				Both	Female	Male	Both	Female	Male	Both	Female	Male
			Metabolic	0 (0 to 1)	0 (0 to 1)	0 (0 to 0)	2 (0 to 4)	2 (0 to 4)	0 (0 to 0)	565.39 (35.49 to 1441.34)	565.39 (35.49 to 1441.34)	0 (0 to 0)
	DALYs	Rate	Behavioral	6.66 (3.62 to 11.38)	14 (7.56 to 24.21)	0.31 (0.14 to 0.56)	9.42 (5.74 to 14.02)	19.13 (11.7 to 28.5)	0.25 (0.14 to 0.39)	41.43 (-16.77 to 140.26)	36.63 (-20.07 to 134.82)	-18.75 (-57.19 to 68.36)
			Metabolic	3.46 (-0.08 to 9.17)	7.76 (0.21 to 19.88)	0 (0 to 0)	8.21 (0.12 to 19.21)	17.2 (0.47 to 39.96)	0 (0 to 0)	137.16 (-111.58 to 529.4)	121.61 (-92.32 to 449.55)	0 (0 to 0)
		Number	Behavioral	14 (7 to 24)	13 (7 to 23)	0 (0 to 1)	61 (36 to 92)	60 (36 to 91)	1 (0 to 1)	343.61 (168.7 to 655.61)	349.64 (169.82 to 674.12)	121.32 (15.93 to 366.34)
			Metabolic	4 (-2 to 14)	4 (-2 to 14)	0 (0 to 0)	22 (-30 to 80)	22 (-30 to 80)	0 (0 to 0)	380.76 (-1988.48 to 3173.98)	380.76 (-1988.48 to 3173.98)	0 (0 to 0)
Kurdistan	Deaths	Rate	Behavioral	0.34 (0.2 to 0.55)	0.74 (0.42 to 1.19)	0.01 (0 to 0.01)	0.41 (0.27 to 0.58)	0.83 (0.53 to 1.15)	0.01 (0 to 0.01)	20.22 (-23 to 82.67)	11.42 (-29.79 to 70.6)	-9.51 (-48.21 to 61.07)
			Metabolic	0.25 (0.05 to 0.58)	0.59 (0.12 to 1.32)	0 (0 to 0)	0.63 (0.21 to 1.23)	1.27 (0.42 to 2.46)	0 (0 to 0)	149.04 (31.38 to 431.74)	116.01 (14.23 to 353.06)	0 (0 to 0)
		Number	Behavioral	2 (1 to 3)	2 (1 to 3)	0 (0 to 0)	6 (4 to 9)	6 (4 to 9)	0 (0 to 0)	207.68 (98.55 to 365.15)	208.49 (99.51 to 367.63)	123.27 (25.96 to 309.09)
			Metabolic	1 (0 to 3)	1 (0 to 3)	0 (0 to 0)	8 (2 to 16)	8 (2 to 16)	0 (0 to 0)	532.69 (167.74 to 1486.22)	532.69 (167.74 to 1486.22)	0 (0 to 0)
	DALYs	Rate	Behavioral	10.57 (6.04 to 16.27)	22.5 (12.79 to 34.94)	0.17 (0.08 to 0.3)	12.33 (7.73 to 17.31)	24.52 (15.32 to 34.44)	0.16 (0.09 to 0.26)	16.73 (-22 to 72.88)	8.99 (-27.17 to 62.94)	-4.98 (-46.9 to 75.44)
			Metabolic	4.67 (-0.99 to 12.54)	11.34 (-0.81 to 28.68)	0 (0 to 0)	12.79 (1.77 to 28.03)	24.92 (2.84 to 55.19)	0 (0 to 0)	173.78 (-282.92 to 910.06)	119.74 (-107.77 to 505.99)	0 (0 to 0)
		Number	Behavioral	70 (40 to 108)	70 (39 to 107)	1 (0 to 1)	208 (128 to 291)	207 (127 to 290)	1 (1 to 2)	194.82 (96.09 to 338.24)	195.41 (96.02 to 340.42)	123.54 (24.54 to 320.81)

Province	Measure	Metric	Risk factors	1990			2019			% Change (1990 to 2019)		
				Both	Female	Male	Both	Female	Male	Both	Female	Male
			Metabolic	23 (-13 to 69)	23 (-13 to 69)	0 (0 to 0)	148 (-34 to 381)	148 (-34 to 381)	0 (0 to 0)	536.5 (-1651.58 to 3017.36)	536.5 (-1651.58 to 3017.36)	0 (0 to 0)
Lorestan	Deaths	Rate	Behavioral	0.33 (0.18 to 0.55)	0.71 (0.38 to 1.17)	0.01 (0 to 0.01)	0.36 (0.22 to 0.53)	0.71 (0.42 to 1.03)	0.01 (0 to 0.01)	9.26 (-34.73 to 80.6)	-0.32 (-40.89 to 64.51)	-13.89 (-53.27 to 63.17)
			Metabolic	0.29 (0.06 to 0.67)	0.65 (0.14 to 1.48)	0 (0 to 0)	0.52 (0.15 to 1.06)	1.01 (0.29 to 2.07)	0 (0 to 0)	80.3 (-10.02 to 274.5)	55.63 (-21.94 to 201.87)	0 (0 to 0)
		Number	Behavioral	2 (1 to 4)	2 (1 to 4)	0 (0 to 0)	6 (4 to 9)	6 (3 to 9)	0 (0 to 0)	167.69 (63.77 to 330.19)	168.77 (63.83 to 334.26)	85.4 (-4.23 to 254.03)
			Metabolic	2 (0 to 4)	2 (0 to 4)	0 (0 to 0)	7 (1 to 15)	7 (1 to 15)	0 (0 to 0)	333.28 (68.7 to 896.35)	333.28 (68.7 to 896.35)	0 (0 to 0)
	DALYs	Rate	Behavioral	10.02 (5.46 to 15.89)	21.37 (11.67 to 34.16)	0.22 (0.11 to 0.39)	11.21 (6.7 to 16.42)	21.9 (13.06 to 32.15)	0.19 (0.11 to 0.3)	11.91 (-30.17 to 77.79)	2.46 (-36.48 to 64.47)	-12.57 (-53.5 to 68.97)
			Metabolic	5.35 (-0.46 to 14.01)	12.88 (0.08 to 31.86)	0 (0 to 0)	10.49 (0.44 to 24.85)	20.07 (0.41 to 47.95)	0 (0 to 0)	96.21 (-140.88 to 591.48)	55.87 (-73.49 to 348.91)	0 (0 to 0)
		Number	Behavioral	75 (42 to 120)	75 (41 to 119)	1 (0 to 2)	201 (120 to 296)	199 (119 to 294)	2 (1 to 3)	166.35 (69.47 to 320.14)	167.37 (69.26 to 323.54)	81.24 (-5.52 to 254.46)
			Metabolic	30 (-11 to 88)	30 (-11 to 88)	0 (0 to 0)	123 (-50 to 347)	123 (-50 to 347)	0 (0 to 0)	316.62 (-783.93 to 1605.32)	316.62 (-783.93 to 1605.32)	0 (0 to 0)
Markazi	Deaths	Rate	Behavioral	0.4 (0.21 to 0.64)	0.79 (0.43 to 1.27)	0.01 (0 to 0.01)	0.44 (0.28 to 0.63)	0.87 (0.55 to 1.26)	0.01 (0 to 0.01)	11.08 (-28.12 to 67.03)	10.3 (-28.74 to 66)	-0.19 (-42.79 to 78.84)
			Metabolic	0.32 (0.06 to 0.74)	0.67 (0.14 to 1.52)	0 (0 to 0)	0.67 (0.2 to 1.31)	1.31 (0.38 to 2.59)	0 (0 to 0)	108.09 (10.78 to 324.51)	97.4 (2.97 to 282.08)	0 (0 to 0)
		Number	Behavioral	3 (1 to 4)	3 (1 to 4)	0 (0 to 0)	7 (4 to 10)	7 (4 to 10)	0 (0 to 0)	167.67 (75.33 to 301.68)	167.91 (74.89 to 301.97)	138.96 (34.86 to 330.78)

Province	Measure	Metric	Risk factors	1990			2019			% Change (1990 to 2019)		
				Both	Female	Male	Both	Female	Male	Both	Female	Male
			Metabolic	2 (0 to 4)	2 (0 to 4)	0 (0 to 0)	9 (2 to 19)	9 (2 to 19)	0 (0 to 0)	397.71 (130.3 to 974.18)	397.71 (130.3 to 974.18)	0 (0 to 0)
	DALYs	Rate	Behavioral	12.15 (6.51 to 19.16)	24.17 (12.95 to 38.17)	0.19 (0.09 to 0.33)	13.4 (8.16 to 19.08)	26.66 (16.18 to 38.02)	0.2 (0.11 to 0.31)	10.29 (-25.84 to 61.54)	10.29 (-26.35 to 61.82)	4.04 (-41.11 to 91.45)
			Metabolic	5.68 (-1.12 to 15.31)	12.28 (-1.31 to 31.95)	0 (0 to 0)	12.83 (0.12 to 29.58)	24.94 (-0.49 to 58.34)	0 (0 to 0)	125.84 (-572.44 to 661.33)	103.2 (-181.97 to 702.33)	0 (0 to 0)
		Number	Behavioral	87 (46 to 137)	86 (45 to 136)	1 (0 to 1)	221 (133 to 318)	219 (132 to 317)	2 (1 to 2)	153.31 (69.83 to 268.85)	153.49 (69.45 to 270.26)	130.15 (30.12 to 324.56)
			Metabolic	37 (-10 to 103)	37 (-10 to 103)	0 (0 to 0)	164 (-41 to 428)	164 (-41 to 428)	0 (0 to 0)	343.86 (-765.63 to 1278.28)	343.86 (-765.63 to 1278.28)	0 (0 to 0)
Mazandaran	Deaths	Rate	Behavioral	0.36 (0.21 to 0.61)	0.72 (0.41 to 1.21)	0.01 (0 to 0.01)	0.49 (0.3 to 0.7)	0.97 (0.6 to 1.38)	0.01 (0 to 0.01)	34.77 (-18.6 to 115.84)	34.03 (-18.99 to 114.65)	2.41 (-41.07 to 89.95)
			Metabolic	0.34 (0.07 to 0.81)	0.68 (0.13 to 1.6)	0 (0 to 0)	0.75 (0.19 to 1.49)	1.46 (0.36 to 2.91)	0 (0 to 0)	118.62 (13.33 to 322.33)	113.67 (9.07 to 305.18)	0 (0 to 0)
		Number	Behavioral	5 (3 to 8)	5 (3 to 8)	0 (0 to 0)	20 (12 to 28)	20 (12 to 28)	0 (0 to 0)	304.1 (146.04 to 544.01)	304.84 (145.68 to 547.1)	210.3 (71.63 to 489.39)
			Metabolic	4 (0 to 9)	4 (0 to 9)	0 (0 to 0)	26 (5 to 54)	26 (5 to 54)	0 (0 to 0)	644.12 (241.52 to 1783.73)	644.12 (241.52 to 1783.73)	0 (0 to 0)
	DALYs	Rate	Behavioral	11.5 (6.42 to 19.09)	23.05 (12.91 to 38.23)	0.17 (0.08 to 0.3)	16.04 (9.72 to 23.03)	31.74 (19.22 to 45.71)	0.17 (0.1 to 0.28)	39.49 (-13.6 to 116.87)	37.72 (-14.96 to 114.74)	5.88 (-40.74 to 92.86)
			Metabolic	6.46 (-1.08 to 18.12)	13.46 (-1.84 to 37.06)	0 (0 to 0)	15.14 (-1.11 to 34.68)	29.21 (-3.04 to 67.88)	0 (0 to 0)	134.26 (-190.85 to 596.75)	117.09 (-172.26 to 458.76)	0 (0 to 0)
		Number	Behavioral	173 (97 to 287)	172 (96 to 286)	1 (1 to 2)	684 (411 to 995)	680 (408 to 990)	3 (2 to 6)	294.08 (146 to 507.57)	294.8 (145.73 to 510.43)	190.33 (59.83 to 447.4)

Province	Measure	Metric	Risk factors	1990			2019			% Change (1990 to 2019)		
				Both	Female	Male	Both	Female	Male	Both	Female	Male
			Metabolic	68 (-41 to 218)	68 (-41 to 218)	0 (0 to 0)	538 (-148 to 1337)	538 (-148 to 1337)	0 (0 to 0)	697.6 (-2544.05 to 3060.94)	697.6 (-2544.05 to 3060.94)	0 (0 to 0)
North Khorasan	Deaths	Rate	Behavioral	0.34 (0.19 to 0.54)	0.69 (0.39 to 1.1)	0.01 (0.01 to 0.02)	0.41 (0.25 to 0.58)	0.8 (0.49 to 1.15)	0.01 (0 to 0.01)	20.43 (-23.99 to 85.69)	15.67 (-27.81 to 79.67)	-22.47 (-56.26 to 44.08)
			Metabolic	0.36 (0.07 to 0.86)	0.77 (0.17 to 1.85)	0 (0 to 0)	0.67 (0.18 to 1.35)	1.33 (0.36 to 2.69)	0 (0 to 0)	86.87 (3.97 to 265.4)	72.47 (-3.19 to 221.15)	0 (0 to 0)
		Number	Behavioral	1 (1 to 2)	1 (1 to 2)	0 (0 to 0)	3 (2 to 5)	3 (2 to 5)	0 (0 to 0)	202.79 (96.24 to 356.69)	204.91 (96.07 to 365.28)	82.46 (0.08 to 244.7)
			Metabolic	1 (0 to 2)	1 (0 to 2)	0 (0 to 0)	4 (1 to 9)	4 (1 to 9)	0 (0 to 0)	378.52 (135.47 to 990.76)	378.52 (135.47 to 990.76)	0 (0 to 0)
	DALYs	Rate	Behavioral	10.52 (6.01 to 16.38)	21.54 (12.44 to 33.8)	0.33 (0.15 to 0.56)	12.86 (7.64 to 18.54)	25.15 (14.93 to 36.38)	0.26 (0.14 to 0.39)	22.22 (-19.75 to 82.11)	16.77 (-23.85 to 76.59)	-21.04 (-55.56 to 47.79)
			Metabolic	7.49 (-0.37 to 19.74)	16.86 (0.72 to 43.04)	0 (0 to 0)	14.08 (0.41 to 32.67)	27.41 (0.38 to 63.93)	0 (0 to 0)	87.91 (-78.76 to 453)	62.57 (-72.47 to 276.38)	0 (0 to 0)
		Number	Behavioral	37 (21 to 58)	37 (20 to 57)	1 (0 to 1)	109 (65 to 159)	108 (64 to 158)	1 (1 to 2)	194.93 (97.06 to 333.49)	196.86 (98.01 to 337.95)	73.98 (-3.41 to 228.42)
			Metabolic	21 (-5 to 59)	21 (-5 to 59)	0 (0 to 0)	89 (-23 to 239)	89 (-23 to 239)	0 (0 to 0)	321.36 (-306.28 to 1358.09)	321.36 (-306.28 to 1358.09)	0 (0 to 0)
Qazvin	Deaths	Rate	Behavioral	0.34 (0.18 to 0.57)	0.67 (0.37 to 1.14)	0.01 (0 to 0.01)	0.42 (0.26 to 0.6)	0.83 (0.51 to 1.18)	0.01 (0 to 0.01)	25.69 (-23.86 to 111.02)	23.21 (-26.05 to 107.16)	-4.94 (-47.4 to 71.53)
			Metabolic	0.27 (0.05 to 0.64)	0.54 (0.1 to 1.29)	0 (0 to 0)	0.56 (0.13 to 1.15)	1.06 (0.24 to 2.22)	0 (0 to 0)	108.85 (-4.5 to 354.99)	94.56 (-12.16 to 311.04)	0 (0 to 0)
		Number	Behavioral	2 (1 to 2)	1 (1 to 2)	0 (0 to 0)	5 (3 to 8)	5 (3 to 8)	0 (0 to 0)	260.49 (123.09 to 503.71)	261.85 (123.02 to 508.18)	142.36 (34.2 to 352.81)

Province	Measure	Metric	Risk factors	1990			2019			% Change (1990 to 2019)		
				Both	Female	Male	Both	Female	Male	Both	Female	Male
			Metabolic	1 (0 to 2)	1 (0 to 2)	0 (0 to 0)	5 (0 to 12)	5 (0 to 12)	0 (0 to 0)	421.58 (18.76 to 1141.81)	421.58 (18.76 to 1141.81)	0 (0 to 0)
	DALYs	Rate	Behavioral	10.09 (5.54 to 16.27)	20.53 (11.24 to 33.33)	0.21 (0.1 to 0.35)	13.3 (7.99 to 19.1)	26.64 (15.96 to 38.34)	0.2 (0.11 to 0.31)	31.81 (-14.46 to 116.15)	29.78 (-16.73 to 114.87)	-4.99 (-48.11 to 77.32)
			Metabolic	4.56 (-1 to 12.68)	9.88 (-1.59 to 26.73)	0 (0 to 0)	10.07 (-1.25 to 25.49)	18.96 (-3.62 to 49.07)	0 (0 to 0)	120.62 (-327.19 to 739.3)	91.84 (-252.19 to 570.66)	0 (0 to 0)
		Number	Behavioral	51 (28 to 81)	50 (28 to 80)	1 (0 to 1)	191 (113 to 277)	190 (112 to 275)	1 (1 to 2)	276.52 (145.56 to 521.1)	277.97 (145.22 to 529.61)	138.6 (28.89 to 358)
			Metabolic	18 (-9 to 55)	18 (-9 to 55)	0 (0 to 0)	76 (-81 to 269)	76 (-81 to 269)	0 (0 to 0)	330.83 (-1422.07 to 2164.23)	330.83 (-1422.07 to 2164.23)	0 (0 to 0)
Qom	Deaths	Rate	Behavioral	0.47 (0.26 to 0.78)	0.95 (0.51 to 1.59)	0.02 (0.01 to 0.03)	0.54 (0.34 to 0.77)	1.12 (0.7 to 1.61)	0.01 (0.01 to 0.02)	14.32 (-27.61 to 91.84)	18.01 (-25.9 to 100.62)	-27.09 (-58.82 to 32.42)
			Metabolic	0.43 (0.08 to 1.01)	0.87 (0.16 to 2.05)	0 (0 to 0)	0.87 (0.27 to 1.7)	1.85 (0.58 to 3.6)	0 (0 to 0)	102.87 (11.36 to 341.66)	113.11 (17.38 to 376.07)	0 (0 to 0)
		Number	Behavioral	2 (1 to 3)	2 (1 to 3)	0 (0 to 0)	6 (4 to 9)	6 (4 to 9)	0 (0 to 0)	300.09 (156.97 to 620.07)	302.68 (157.71 to 631.25)	150.92 (37.66 to 378.2)
			Metabolic	1 (0 to 3)	1 (0 to 3)	0 (0 to 0)	8 (2 to 16)	8 (2 to 16)	0 (0 to 0)	620.34 (188.95 to 1940.46)	620.34 (188.95 to 1940.46)	0 (0 to 0)
	DALYs	Rate	Behavioral	14.35 (7.31 to 23.3)	29.39 (14.91 to 47.76)	0.45 (0.19 to 0.75)	16.38 (10 to 23.58)	33.51 (20.44 to 48.28)	0.33 (0.18 to 0.51)	14.16 (-25.65 to 105.27)	14 (-26.17 to 105.98)	-25.77 (-59.29 to 42.77)
			Metabolic	7.79 (-1.37 to 21.11)	16.13 (-2.81 to 43.5)	0 (0 to 0)	17.85 (2.42 to 39.24)	36.9 (4.97 to 81.25)	0 (0 to 0)	129.06 (-454.71 to 644.81)	128.73 (-442.84 to 647.7)	0 (0 to 0)
		Number	Behavioral	55 (27 to 89)	54 (27 to 87)	1 (0 to 1)	215 (129 to 315)	213 (128 to 313)	2 (1 to 3)	292.42 (154.61 to 654.61)	294.76 (154.28 to 661.08)	144.96 (30.24 to 382.58)

Province	Measure	Metric	Risk factors	1990			2019			% Change (1990 to 2019)		
				Both	Female	Male	Both	Female	Male	Both	Female	Male
			Metabolic	19 (-15 to 64)	19 (-15 to 64)	0 (0 to 0)	147 (-45 to 393)	147 (-45 to 393)	0 (0 to 0)	687.45 (-2789.02 to 3874.08)	687.45 (-2789.02 to 3874.08)	0 (0 to 0)
Semnan	Deaths	Rate	Behavioral	0.41 (0.21 to 0.74)	0.8 (0.41 to 1.42)	0.01 (0.01 to 0.02)	0.48 (0.31 to 0.71)	0.94 (0.59 to 1.37)	0.02 (0.01 to 0.02)	17.42 (-29.98 to 97.73)	18.06 (-29.91 to 101.16)	34.54 (-27.71 to 151.92)
			Metabolic	0.4 (0.07 to 1.01)	0.77 (0.13 to 1.93)	0 (0 to 0)	0.78 (0.22 to 1.59)	1.49 (0.41 to 3.07)	0 (0 to 0)	93.67 (-4.43 to 325.88)	93.57 (-6.81 to 322.54)	0 (0 to 0)
		Number	Behavioral	1 (1 to 2)	1 (1 to 2)	0 (0 to 0)	4 (2 to 5)	3 (2 to 5)	0 (0 to 0)	207.95 (85.16 to 419)	207.65 (83.99 to 423.77)	230.05 (77.19 to 525.89)
			Metabolic	1 (0 to 3)	1 (0 to 3)	0 (0 to 0)	5 (1 to 10)	5 (1 to 10)	0 (0 to 0)	376.39 (104.99 to 969.45)	376.39 (104.99 to 969.45)	0 (0 to 0)
	DALYs	Rate	Behavioral	12.47 (6.41 to 20.85)	24.68 (12.75 to 41.33)	0.3 (0.14 to 0.52)	15.16 (9.05 to 21.91)	30.05 (17.88 to 43.57)	0.41 (0.24 to 0.63)	21.53 (-24.38 to 100.93)	21.73 (-24.88 to 102.55)	36.38 (-26.94 to 163.35)
			Metabolic	7.52 (-0.86 to 21.31)	15 (-1.78 to 42.47)	0 (0 to 0)	15.22 (0.01 to 36.62)	29.61 (-0.9 to 72.1)	0 (0 to 0)	102.46 (-111.93 to 765.22)	97.37 (-120.66 to 675.24)	0 (0 to 0)
		Number	Behavioral	38 (20 to 63)	38 (19 to 62)	0 (0 to 1)	120 (71 to 175)	119 (70 to 173)	2 (1 to 2)	216.21 (99.04 to 412.49)	216.06 (97.59 to 415.14)	228.81 (76.33 to 541.16)
			Metabolic	20 (-5 to 60)	20 (-5 to 60)	0 (0 to 0)	89 (-30 to 247)	89 (-30 to 247)	0 (0 to 0)	337.44 (-516.18 to 1389.35)	337.44 (-516.18 to 1389.35)	0 (0 to 0)
Sistan and Baluchistan	Deaths	Rate	Behavioral	0.3 (0.16 to 0.48)	0.67 (0.35 to 1.07)	0.01 (0 to 0.01)	0.36 (0.22 to 0.52)	0.71 (0.43 to 1.04)	0.01 (0 to 0.01)	18.37 (-27.7 to 97.61)	6.39 (-35.43 to 78.85)	-22.19 (-56.96 to 48.2)
			Metabolic	0.23 (0.05 to 0.51)	0.53 (0.12 to 1.15)	0 (0 to 0)	0.48 (0.15 to 0.97)	0.96 (0.29 to 1.95)	0 (0 to 0)	111.03 (13.46 to 322.27)	81.96 (-2.98 to 248.7)	0 (0 to 0)
		Number	Behavioral	2 (1 to 3)	2 (1 to 3)	0 (0 to 0)	6 (3 to 8)	6 (3 to 8)	0 (0 to 0)	212.58 (91.16 to 432.01)	214.28 (91.43 to 435.35)	79.2 (-2.82 to 264.79)

Province	Measure	Metric	Risk factors	1990			2019			% Change (1990 to 2019)		
				Both	Female	Male	Both	Female	Male	Both	Female	Male
			Metabolic	1 (0 to 2)	1 (0 to 2)	0 (0 to 0)	5 (0 to 11)	5 (0 to 11)	0 (0 to 0)	352.64 (19.84 to 784.11)	352.64 (19.84 to 784.11)	0 (0 to 0)
	DALYs	Rate	Behavioral	9.03 (4.69 to 14.36)	20.02 (10.39 to 31.97)	0.2 (0.08 to 0.36)	10.98 (6.42 to 15.98)	21.8 (12.75 to 31.72)	0.15 (0.08 to 0.24)	21.62 (-24.99 to 100.98)	8.9 (-33.29 to 82.1)	-22.59 (-57.67 to 57.02)
			Metabolic	4.41 (0.01 to 10.88)	10.8 (0.75 to 25.53)	0 (0 to 0)	9.94 (1.12 to 22.48)	19.69 (1.97 to 44.61)	0 (0 to 0)	125.5 (-19.01 to 519.99)	82.28 (-38.16 to 331.64)	0 (0 to 0)
		Number	Behavioral	61 (31 to 98)	60 (30 to 97)	1 (0 to 1)	200 (114 to 298)	198 (113 to 297)	1 (1 to 2)	228.59 (101.91 to 446.38)	230.38 (101.5 to 456.35)	79.58 (-2.9 to 278.98)
			Metabolic	22 (-7 to 61)	22 (-7 to 61)	0 (0 to 0)	76 (-73 to 271)	76 (-73 to 271)	0 (0 to 0)	250.6 (-1023.87 to 1824.3)	250.6 (-1023.87 to 1824.3)	0 (0 to 0)
South Khorasan	Deaths	Rate	Behavioral	0.39 (0.22 to 0.61)	0.8 (0.46 to 1.27)	0.01 (0 to 0.01)	0.46 (0.28 to 0.66)	0.88 (0.53 to 1.27)	0.01 (0 to 0.01)	19.06 (-26.9 to 82.36)	10.67 (-31.95 to 71.1)	-17.72 (-55.09 to 48.86)
			Metabolic	0.5 (0.12 to 1.17)	1.06 (0.27 to 2.46)	0 (0 to 0)	0.96 (0.32 to 1.92)	1.85 (0.6 to 3.69)	0 (0 to 0)	93.24 (12.86 to 234.72)	74.46 (1.92 to 196.77)	0 (0 to 0)
		Number	Behavioral	1 (1 to 2)	1 (1 to 2)	0 (0 to 0)	3 (2 to 5)	3 (2 to 5)	0 (0 to 0)	134.36 (44.09 to 256.34)	135.32 (44.12 to 260.37)	47.43 (-20.54 to 173.93)
			Metabolic	2 (0 to 4)	2 (0 to 4)	0 (0 to 0)	6 (2 to 13)	6 (2 to 13)	0 (0 to 0)	265.71 (106.65 to 516.63)	265.71 (106.65 to 516.63)	0 (0 to 0)
	DALYs	Rate	Behavioral	11.58 (6.74 to 17.71)	24 (13.92 to 36.82)	0.22 (0.1 to 0.41)	13.75 (8.22 to 19.97)	26.63 (15.88 to 38.74)	0.18 (0.1 to 0.29)	18.76 (-24.64 to 77.93)	10.96 (-29.92 to 67.23)	-18.6 (-55.86 to 50.82)
			Metabolic	10.88 (1.09 to 26.79)	23.73 (2.97 to 57.45)	0 (0 to 0)	21.09 (4.02 to 46.68)	40.28 (7.05 to 90)	0 (0 to 0)	93.8 (0.21 to 300.18)	69.78 (-16.4 to 223.94)	0 (0 to 0)
		Number	Behavioral	47 (27 to 72)	47 (27 to 72)	0 (0 to 1)	107 (64 to 155)	106 (63 to 154)	1 (0 to 1)	126.82 (44.1 to 241.42)	127.73 (43.8 to 243.24)	38.44 (-25.26 to 162.75)

Province	Measure	Metric	Risk factors	1990			2019			% Change (1990 to 2019)		
				Both	Female	Male	Both	Female	Male	Both	Female	Male
			**Metabolic**	43 (5 to 104)	43 (5 to 104)	0 (0 to 0)	136 (8 to 324)	136 (8 to 324)	0 (0 to 0)	214.07 (2.5 to 464.17)	214.07 (2.5 to 464.17)	0 (0 to 0)
**Tehran**	**Deaths**	**Rate**	**Behavioral**	0.57 (0.33 to 0.94)	1.15 (0.67 to 1.89)	0.01 (0 to 0.02)	0.55 (0.34 to 0.79)	1.1 (0.68 to 1.59)	0.01 (0 to 0.01)	-4.88 (-39.36 to 52.27)	-4.44 (-38.68 to 53.41)	-17.68 (-57.62 to 54.91)
			**Metabolic**	0.73 (0.18 to 1.62)	1.43 (0.35 to 3.17)	0 (0 to 0)	1.01 (0.33 to 1.97)	2.04 (0.67 to 3.99)	0 (0 to 0)	38.34 (-19.42 to 157.42)	42.62 (-15.9 to 164.82)	0 (0 to 0)
		**Number**	**Behavioral**	25 (14 to 41)	25 (14 to 41)	0 (0 to 0)	80 (49 to 116)	80 (49 to 115)	0 (0 to 1)	219.96 (107.15 to 407.2)	220.18 (106.68 to 411.47)	186.46 (44.26 to 463.59)
			**Metabolic**	24 (4 to 56)	24 (4 to 56)	0 (0 to 0)	128 (36 to 258)	128 (36 to 258)	0 (0 to 0)	431.19 (189.27 to 1001.28)	431.19 (189.27 to 1001.28)	0 (0 to 0)
	**DALYs**	**Rate**	**Behavioral**	16.75 (9.66 to 27.37)	34.42 (19.72 to 56.4)	0.21 (0.1 to 0.38)	16.09 (9.94 to 23.52)	32.18 (19.81 to 47.03)	0.18 (0.1 to 0.27)	-3.89 (-36.89 to 49.62)	-6.5 (-38.93 to 45.27)	-14.05 (-56.55 to 64.69)
			**Metabolic**	14.39 (1.22 to 34.4)	29.08 (2.25 to 69.41)	0 (0 to 0)	20.41 (3.72 to 44.08)	40.78 (7.29 to 88.45)	0 (0 to 0)	41.85 (-27.08 to 211.29)	40.24 (-27.46 to 209.22)	0 (0 to 0)
		**Number**	**Behavioral**	856 (474 to 1380)	851 (472 to 1373)	5 (2 to 9)	2562 (1553 to 3752)	2548 (1543 to 3734)	14 (8 to 21)	199.35 (95.35 to 361.19)	199.57 (94.65 to 362.57)	163.69 (36.13 to 422.6)
			**Metabolic**	483 (-111 to 1340)	483 (-111 to 1340)	0 (0 to 0)	2557 (73 to 6066)	2557 (73 to 6066)	0 (0 to 0)	429.39 (-879.32 to 2136.19)	429.39 (-879.32 to 2136.19)	0 (0 to 0)
**West Azarbayejan**	**Deaths**	**Rate**	**Behavioral**	0.39 (0.23 to 0.63)	0.81 (0.47 to 1.3)	0.01 (0 to 0.01)	0.48 (0.3 to 0.68)	0.94 (0.59 to 1.33)	0.01 (0 to 0.01)	22.28 (-23.52 to 87.08)	15.95 (-27.74 to 78.36)	-4.14 (-42.89 to 65.73)
			**Metabolic**	0.33 (0.07 to 0.77)	0.69 (0.15 to 1.6)	0 (0 to 0)	0.8 (0.26 to 1.57)	1.53 (0.49 to 3.02)	0 (0 to 0)	143.92 (34.73 to 380.92)	121.33 (21.5 to 329.1)	0 (0 to 0)
		**Number**	**Behavioral**	4 (2 to 7)	4 (2 to 7)	0 (0 to 0)	14 (9 to 20)	14 (9 to 20)	0 (0 to 0)	230.72 (110.08 to 404.74)	231.29 (109.85 to 408.27)	145.16 (38.6 to 330.8)

Province	Measure	Metric	Risk factors	1990			2019			% Change (1990 to 2019)		
				Both	Female	Male	Both	Female	Male	Both	Female	Male
			Metabolic	3 (0 to 6)	3 (0 to 6)	0 (0 to 0)	18 (4 to 39)	18 (4 to 39)	0 (0 to 0)	580.48 (216.72 to 1457.06)	580.48 (216.72 to 1457.06)	0 (0 to 0)
	DALYs	Rate	Behavioral	11.59 (6.69 to 18.14)	23.99 (13.9 to 37.81)	0.14 (0.06 to 0.26)	13.92 (8.5 to 20.05)	27.51 (16.79 to 39.73)	0.13 (0.07 to 0.2)	20.09 (-21.83 to 76.99)	14.71 (-25.65 to 69.75)	-2.34 (-42.99 to 67.35)
			Metabolic	5.69 (-0.67 to 14.73)	12.73 (-0.81 to 31.73)	0 (0 to 0)	14.99 (1.62 to 33.71)	28.73 (2.48 to 65.13)	0 (0 to 0)	163.46 (-210.64 to 857.8)	125.69 (-131.68 to 498.32)	0 (0 to 0)
		Number	Behavioral	145 (84 to 226)	145 (83 to 225)	1 (0 to 2)	462 (280 to 669)	460 (279 to 666)	2 (1 to 3)	217.56 (110.2 to 365.4)	218.01 (109.72 to 366.96)	140.85 (36.67 to 321.46)
			Metabolic	52 (-22 to 149)	52 (-22 to 149)	0 (0 to 0)	346 (-72 to 894)	346 (-72 to 894)	0 (0 to 0)	571.43 (-710.49 to 3562.68)	571.43 (-710.49 to 3562.68)	0 (0 to 0)
Yazd	Deaths	Rate	Behavioral	0.45 (0.23 to 0.78)	0.86 (0.44 to 1.5)	0.01 (0.01 to 0.02)	0.51 (0.33 to 0.73)	1.02 (0.65 to 1.46)	0.02 (0.01 to 0.03)	14.94 (-31 to 88.86)	19.42 (-27.88 to 97.58)	37.4 (-20.94 to 152.63)
			Metabolic	0.39 (0.07 to 0.99)	0.72 (0.11 to 1.82)	0 (0 to 0)	0.86 (0.26 to 1.71)	1.69 (0.51 to 3.41)	0 (0 to 0)	116.9 (7.94 to 400.4)	134.03 (16.47 to 471.89)	0 (0 to 0)
		Number	Behavioral	2 (1 to 3)	2 (1 to 3)	0 (0 to 0)	5 (3 to 8)	5 (3 to 7)	0 (0 to 0)	227.06 (104.39 to 432.09)	226.09 (101.83 to 434.23)	290.14 (108.17 to 655.06)
			Metabolic	1 (0 to 3)	1 (0 to 3)	0 (0 to 0)	7 (1 to 15)	7 (1 to 15)	0 (0 to 0)	452.92 (124.2 to 1246.17)	452.92 (124.2 to 1246.17)	0 (0 to 0)
	DALYs	Rate	Behavioral	13.51 (7.1 to 22.63)	26.86 (14.07 to 44.67)	0.38 (0.18 to 0.63)	15.59 (9.86 to 22.39)	31.68 (19.97 to 45.51)	0.53 (0.29 to 0.84)	15.41 (-26.72 to 82.14)	17.93 (-25.22 to 85.99)	41.45 (-23.47 to 166.38)
			Metabolic	6.75 (-1.55 to 19.91)	12.64 (-3.83 to 38.47)	0 (0 to 0)	15.36 (-0.11 to 36.96)	31 (-0.82 to 75.55)	0 (0 to 0)	127.7 (-309.39 to 770.69)	145.19 (-367.91 to 1169.1)	0 (0 to 0)
		Number	Behavioral	54 (28 to 88)	53 (28 to 88)	1 (0 to 1)	179 (110 to 257)	176 (108 to 254)	3 (2 to 5)	231.3 (113.82 to 426.05)	230.52 (111.42 to 425.86)	287.76 (111.58 to 633.23)

Province	Measure	Metric	Risk factors	1990			2019			% Change (1990 to 2019)		
				Both	Female	Male	Both	Female	Male	Both	Female	Male
			Metabolic	22 (-10 to 71)	22 (-10 to 71)	0 (0 to 0)	102 (-72 to 319)	102 (-72 to 319)	0 (0 to 0)	356.05 (-1155.57 to 2152.57)	356.05 (-1155.57 to 2152.57)	0 (0 to 0)
Zanjan	Deaths	Rate	Behavioral	0.27 (0.15 to 0.48)	0.55 (0.3 to 0.98)	0 (0 to 0.01)	0.39 (0.25 to 0.54)	0.74 (0.48 to 1.04)	0.01 (0 to 0.01)	40.21 (-15.91 to 123.74)	34.11 (-20.47 to 114.28)	18.14 (-27.55 to 117.55)
			Metabolic	0.22 (0.04 to 0.53)	0.46 (0.1 to 1.08)	0 (0 to 0)	0.6 (0.21 to 1.15)	1.13 (0.39 to 2.19)	0 (0 to 0)	171.61 (43.57 to 531.88)	147.44 (30.97 to 418.9)	0 (0 to 0)
		Number	Behavioral	1 (1 to 2)	1 (1 to 2)	0 (0 to 0)	4 (3 to 6)	4 (3 to 6)	0 (0 to 0)	245.91 (114.72 to 436.32)	246.57 (114.12 to 438.58)	168.97 (54.49 to 390.68)
			Metabolic	1 (0 to 2)	1 (0 to 2)	0 (0 to 0)	5 (2 to 10)	5 (2 to 10)	0 (0 to 0)	569.47 (253.98 to 1690.45)	569.47 (253.98 to 1690.45)	0 (0 to 0)
	DALYs	Rate	Behavioral	7.94 (4.4 to 13.22)	16.26 (8.92 to 27.23)	0.12 (0.06 to 0.21)	10.94 (7 to 15.6)	21.36 (13.62 to 30.52)	0.14 (0.08 to 0.22)	37.75 (-11.45 to 109.84)	31.38 (-16.62 to 101.09)	18.14 (-29.93 to 112.84)
			Metabolic	3.85 (-0.52 to 10.32)	8.78 (-0.4 to 22.58)	0 (0 to 0)	12.14 (2.43 to 25.3)	23.03 (4.16 to 48.47)	0 (0 to 0)	215.55 (-430.75 to 1465.12)	162.19 (-174.72 to 687.06)	0 (0 to 0)
		Number	Behavioral	38 (21 to 61)	37 (21 to 61)	0 (0 to 1)	120 (76 to 171)	119 (75 to 171)	1 (0 to 1)	220.37 (108.98 to 379.64)	220.95 (108.72 to 383)	148.04 (41.85 to 343.68)
			Metabolic	16 (-4 to 43)	16 (-4 to 43)	0 (0 to 0)	102 (1 to 237)	102 (1 to 237)	0 (0 to 0)	533.02 (-520.58 to 2121.47)	533.02 (-520.58 to 2121.47)	0 (0 to 0)

Data in parentheses are 95% uncertainty intervals

* Age-standardized rate (per 100,000) † All ages
