# Supplementary material for: National and subnational burden of female and male breast cancer and risk factors in Iran from 1990 to 2019: results from the Global Burden of Disease study 2019
Source: Breast Cancer Res. 2023 Apr 26;25:47. doi: 10.1186/s13058-023-01633-4 (PMC10131337; doi:10.1186/s13058-023-01633-4)
Supplement: Supplementary file 15 — Additional file 15. Contributions by the GBD Iran Breast Cancer Collaborators. [file 13058_2023_1633_MOESM15_ESM.docx]

## GBD 2019 Iran Breast Cancer Authors (core team and collaborators)

| **First Names** | **Last Name** |
| --- | --- |
| Armin | Aryannejad |
| Sahar | Saeedi Moghaddam |
| Baharnaz | Mashinchi |
| Mohammadreza | Tabary |
| Negar | Rezaei |
| Sarvenaz | Shahin |
| Nazila | Rezaei |
| Mohsen | Abbasi-Kangevari |
| Zeinab | Abbasi-Kangevari |
| Hedayat | Abbastabar |
| Hassan | Abidi |
| Hassan | Abolhassani |
| Mohammad | Aghaali |
| Bahman | Ahadinezhad |
| Ali | Ahmadi |
| Sepideh | Ahmadi |
| Marjan | Ajami |
| Mohammad Esmaeil | Akbari |
| Yousef | Alimohamadi |
| Sadaf | Alipour |
| Vahid | Alipour |
| Saeed | Amini |
| Ali Arash | Anoushirvani |
| Jalal | Arabloo |
| Morteza | Arab-Zozani |
| Bahar | Ataeinia |
| Seyyed Shamsadin | Athari |
| Abbas | Azadmehr |
| Sina | Azadnajafabad |
| Mohammadreza | Azangou-Khyavy |
| Amirhossein | Azari Jafari |
| Nader | Bagheri |
| Sara | Bagherieh |
| Saeed | Bahadory |
| Sima | Besharat |
| Somayeh | Bohlouli |
| Natália | Cruz-Martins |
| Mostafa | Dianatinasab |
| Mojtaba | Didehdar |
| Shirin | Djalalinia |
| Fariba | Dorostkar |
| Sharareh | Eskandarieh |
| Bita | Eslami |
| Shahab | Falahi |
| Mohammad | Farahmand |
| Ali | Fatehizadeh |
| Masood | Fereidoonnezhad |
| Nasrin | Galehdar |
| Seyyed-Hadi | Ghamari |
| Ahmad | Ghashghaee |
| Maryam | Gholamalizadeh |
| Ali | Gholami |
| Pouya | Goleij |
| Mohamad | Golitaleb |
| Nima | Hafezi-Nejad |
| Arvin | Haj-Mirzaian |
| Aram | Halimi |
| Soheil | Hassanipour |
| Mohammad | Heidari |
| Zahra | Heidarymeybodi |
| Keyvan | Heydari |
| Mohammad-Salar | Hosseini |
| Elham | Jamshidi |
| Roksana | Janghorban |
| Ali | Kabir |
| Leila R | Kalankesh |
| Taras | Kavetskyy |
| Leila | Keikavoosi-Arani |
| Mohammad | Keykhaei |
| Rovshan | Khalilov |
| Javad | Khanali |
| Mahmoud | Khodadost |
| Ali-Asghar | Kolahi |
| Farzad | Kompani |
| Hamid Reza | Koohestani |
| Mozhgan | Letafat-nezhad |
| Somayeh | Livani |
| Amirhosein | Maali |
| Farzan | Madadizadeh |
| Soleiman | Mahjoub |
| Ata | Mahmoodpoor |
| Mohammad-Reza | Malekpour |
| Reza | Malekzadeh |
| Mohammad Ali | Mansournia |
| Sahar | Masoudi |
| Seyedeh Zahra | Masoumi |
| Entezar | Mehrabi Nasab |
| Seyyedmohammadsadeq | Mirmoeeni |
| Esmaeil | Mohammadi |
| Abdollah | Mohammadian-Hafshejani |
| Mohammad | Mohseni |
| Sara | Momtazmanesh |
| Abdolvahab | Moradi |
| Maryam | Moradi |
| Yousef | Moradi |
| Farhad | Moradpour |
| Rahmatollah | Moradzadeh |
| Abbas | Mosapour |
| Mozhgan | Moshtagh |
| Haleh | Mousavi Isfahani |
| Christopher J L | Murray |
| Javad | Nazari |
| Seyed Aria | Nejadghaderi |
| Maryam | Noori |
| Hassan | Okati-Aliabad |
| Morteza | Oladnabi |
| Babak | Pakbin |
| Fatemeh | Pashazadeh Kan |
| Hamidreza | Pazoki Toroudi |
| Naeimeh | Pourtaheri |
| Navid | Rabiee |
| Sima | Rafiei |
| Fakher | Rahim |
| Vahid | Rahmanian |
| Samira | Raoofi |
| Mahsa | Rashidi |
| Mohammad-Mahdi | Rashidi |
| Mohammad Sadegh | Razeghinia |
| Nima | Rezaei |
| Saeid | Rezaei |
| Aziz | Rezapour |
| Gholamreza | Roshandel |
| Siamak | Sabour |
| Maryam | Sahebazzamani |
| Amirhossein | Sahebkar |
| Soraya | Sajadimajd |
| Sadaf G | Sepanlou |
| Saeed | Shahabi |
| Fariba | Shahraki-Sanavi |
| Javad | Sharifi-Rad |
| Reza | Shirkoohi |
| Parnian | Shobeiri |
| Mohammad Sadegh | Soltani-Zangbar |
| Elnaz | Tabibian |
| Majid | Taheri |
| Yasaman | Taheri Abkenar |
| Ahmad | Tavakoli |
| Amir | Tiyuri |
| Seyed Abolfazl | Tohidast |
| Sahel | Valadan Tahbaz |
| Rohollah | Valizadeh |
| Seyed Hossein | Yahyazadeh Jabbari |
| Leila | Zaki |
| Maryam | Zamanian |
| Iman | Zare |
| Mohammad | Zoladl |
| Mohsen | Naghavi |
| Bagher | Larijani |
| Farshad | Farzadfar |
